# High yielding synthesis of 2,2′-bipyridine macrocycles, versatile intermediates in the synthesis of rotaxanes†
†Electronic supplementary information (ESI) available: Full synthetic procedures and characterisation of all novel compounds. CCDC 1442682–1442684. For ESI and crystallographic data in CIF or other electronic format see DOI: 10.1039/c6sc00011h


**DOI:** 10.1039/c6sc00011h

**Published:** 2016-01-27

**Authors:** J. E. M. Lewis, R. J. Bordoli, M. Denis, C. J. Fletcher, M. Galli, E. A. Neal, E. M. Rochette, S. M. Goldup

**Affiliations:** a Chemistry , University of Southampton , Highfield , Southampton , SO17 1BJ , UK . Email: s.goldup@soton.ac.uk; b School of Biological and Chemical Sciences , Queen Mary University of London , Mile End Road , London , E1 4NS , UK

## Abstract

We present an operationally simple approach to 2,2′-bipyridine macrocycles. Our method uses simple starting materials to produce these previously hard to access rotaxane precursors in remarkable yields (typically >65%) across a range of scales (0.1–5 mmol). All of the macrocycles reported are efficiently converted (>90%) to rotaxanes under AT-CuAAC conditions. With the requisite macrocycles finally available in sufficient quantities, we further demonstrate their long term utility through the first gram-scale synthesis of an AT-CuAAC [2]rotaxane and extend this powerful methodology to produce novel Sauvage-type molecular shuttles.

## Abstract 2

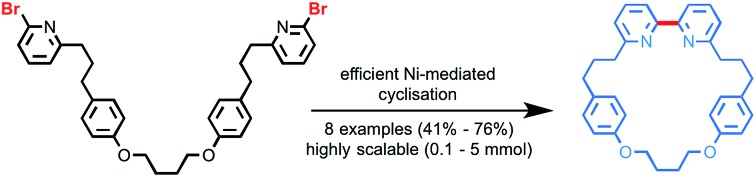
We present a simple approach to bipyridine macrocycles in remarkable yields (typically >65%) and demonstrate their application in efficient rotaxane synthesis.

## Introduction

The synthesis of mechanically interlocked molecules has progressed significantly since early reports.^1^,^2^ This was largely made possible by the development of passive template methodologies, inspired by Sauvage and co-workers' seminal work,^3^ in which non-covalent interactions direct mechanical bond formation.^4^ Thus, there exists a range of methodologies for the synthesis of complex interlocked structures in excellent yield in the mechanical bond-forming step.

Despite innovations in the preparation of interlocked molecules,^5^ the synthesis of the requisite functionalised macrocyclic components is often a challenge that can in turn limit the scalability of their synthesis, hindering their investigation for applications such as molecular machines,^6^ materials,^7^ drug delivery agents,^8^ and catalysts.^9^ Indeed the synthesis of macrocyclic molecules more generally remains an important area of investigation due to their unusual properties^10^ combined with the inherent problem of selecting the desired ring closure event over oligomerisation.^11^ A number of strategies have been developed to overcome this including the use of templating interactions to pre-organise the acyclic precursor for ring closure, the preparation of rigid acyclic precursors with restricted rotational degrees of freedom, and expansion of smaller rings.^12^ Where this is not possible, the most general approach is to carry out the ring-closing step under high dilution, conditions with significant consequences for the scalability of the process.^13^

The contrast in efficiency between the synthesis of the macrocycle and the formation of the mechanical bond is perhaps nowhere more acute than in our small bipyridine macrocycle modification^14^ of Leigh's active template Cu-mediated alkyne-azide cycloaddition^15^ (AT-CuAAC)^16^ reaction.^17^,^18^ Although AT-CuAAC reactions with small bipyridine macrocycles have been described as “amongst the highest yielding and readily accessible routes to stable interlocked structures”,4*c* the synthesis of the macrocyclic bipyridine precursor is extremely inefficient, proceeding in just 4–11% yield. Given the proven utility of the AT-CuAAC reaction for the synthesis of functionalised rotaxanes,14a,^19^ including examples with multiple mechanical bonds,14d,^20^ stabilised organometallic species,14*b* stereochemically complex interlocked molecules,14*c* interlocked catalysts,9*g* and molecular machines,6*a* the limitations placed on the application of this powerful methodology by the poor availability of the most efficacious macrocyclic precursors^19^ is a significant barrier to further developments.

Here we report an operationally simple method for the scalable synthesis of bipyridine macrocycles from readily available substrates that effectively removes this limitation. All of the bipyridine macrocycles reported are efficiently converted to [2]rotaxanes under standard conditions. We demonstrate the long term potential of the AT-CuAAC approach with these now readily available substrates through the gram-scale synthesis of a [2]rotaxane catalyst precursor, and a novel synthesis of Sauvage-type molecular shuttles.

## Results and discussion

### A new Ni-mediated macrocyclisation

Bipyridine macrocycles suitable for AT-CuAAC reactions such as **2a** are typically synthesised *via* a double Williamson cyclisation of a pre-formed bipyridine precursor.^14^,16b,^21^ However, the preferred *trans*-rotamer of the bipyridine unit^22^ disfavours the desired cyclisation, leading to low yields, particularly in the case of smaller macrocycles.^23^ To overcome this, we resolved to form the ligand during the macrocyclisation reaction.

Previous routes to bipyridine macrocycles have made use of the Ni-mediated reductive coupling^24^ of 2-halo pyridines^25^ to produce the bipyridine moiety prior to the macrocyclisation step.^14^,16b Although Ni-mediated couplings have not been employed as a macrocyclisation reaction for the preparation of bipyridine macrocycles for incorporation into interlocked architectures,^26^ similar reactions have been successful in the key cyclisation step towards cyclic and bicyclic oligoparaphenylenes,^27^,^28^ as well as limited examples of bi-phenyl and bi-benzyl natural products.^29^ Accordingly, dibromo precursor **1a** was synthesised from known intermediates in a succinct manner (see ESI†) and, under high dilution, subjected to conditions based on those developed by the groups of Tiecco and Iyoda for the Ni-mediated intermolecular homo-coupling of 2-halo-pyridines.25a,c No reaction was observed (Table 1, entry 1).

**Table 1:** Optimisation of the Ni-mediated synthesis of macrocycle **2a**^*a*^

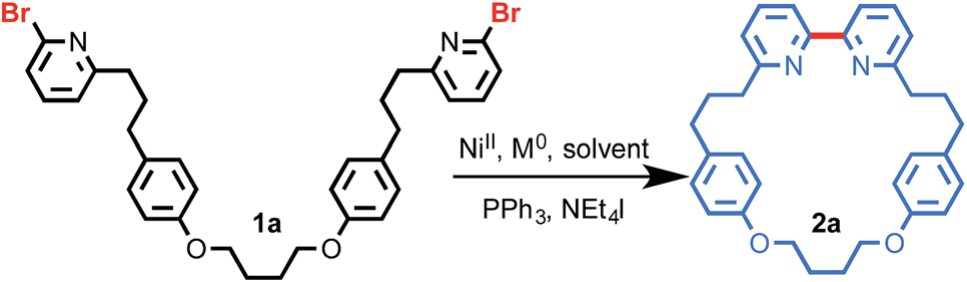				
Entry	Ni^II^ source	M^0^ (equiv.)		Yield^*b*^
1^*c*^	NiCl_2_·6H_2_O	Zn (2)	n.r.	
2^*d*^	NiCl_2_·6H_2_O	Zn (2)	7%	
3^*e*^	NiCl_2_·6H_2_O	Zn (2)	37%	
4^*e*^	[Ni(PPh_3_)_2_Br_2_]	Zn (2)	60%	
5^*e*^ ^,^^*f*^	[Ni(PPh_3_)_2_Br_2_]	Zn (2)	47%	
6^*e*^	[Ni(PPh_3_)_2_Br_2_]	Zn (4)	50%	
7^*e*^	[Ni(PPh_3_)_2_Br_2_]	Mn (2)	63%	
8^*e*^	[Ni(PPh_3_)_2_Br_2_]	Mn (10)	70%	

^*a*^Reagents and conditions: 0.1 mmol each **1a**, Ni^II^, NEt_4_I, 0.4 mmol PPh_3_ in total, DMF, 50 °C, 4 h.

^*b*^Determined by ^1^H NMR analysis of the crude product.

^*c*^5 mM conc. of **1a**.

^*d*^0.05 M conc. of **1a**.

^*e*^Pseudo-high dilution (4 h addition, 0.05 M final conc. of **1a**).

^*f*^THF as solvent. n.r. = no reaction.

Repeating the same reaction at higher concentration (entry 2) led to consumption of **1a** to give **2a**, albeit in low yield. Combining these observations, slow addition of **1a** to the preformed Ni^0^ species under otherwise identical conditions gave macrocycle **2a** in 37% yield (entry 3). A brief screen of reaction conditions revealed that replacing NiCl_2_·6H_2_O with NiBr_2_(PPh_3_)_2_ resulted in an increased yield of **2a** (entry 4), whereas conducting the reaction in THF, commonly employed in such couplings,^25^^*a*^ led to a less efficient reaction (entry 5). Increasing the equivalents of Zn reduced the yield of product, with significant proto-dehalogenation observed in the crude mixture (entry 6). On the hypothesis that this was due to halogen–zinc exchange competing with the desired Ni-mediated process, Mn was substituted for Zn leading to an improved yield (entry 7).^30^ Finally, increasing the equivalents of Mn (entry 8) to improve the efficiency of reductive steps during the catalytic cycle,^24^ gave **2a** in 70% yield.

### Preparative scale synthesis of bipyridine macrocycles

**Fig. 1:**
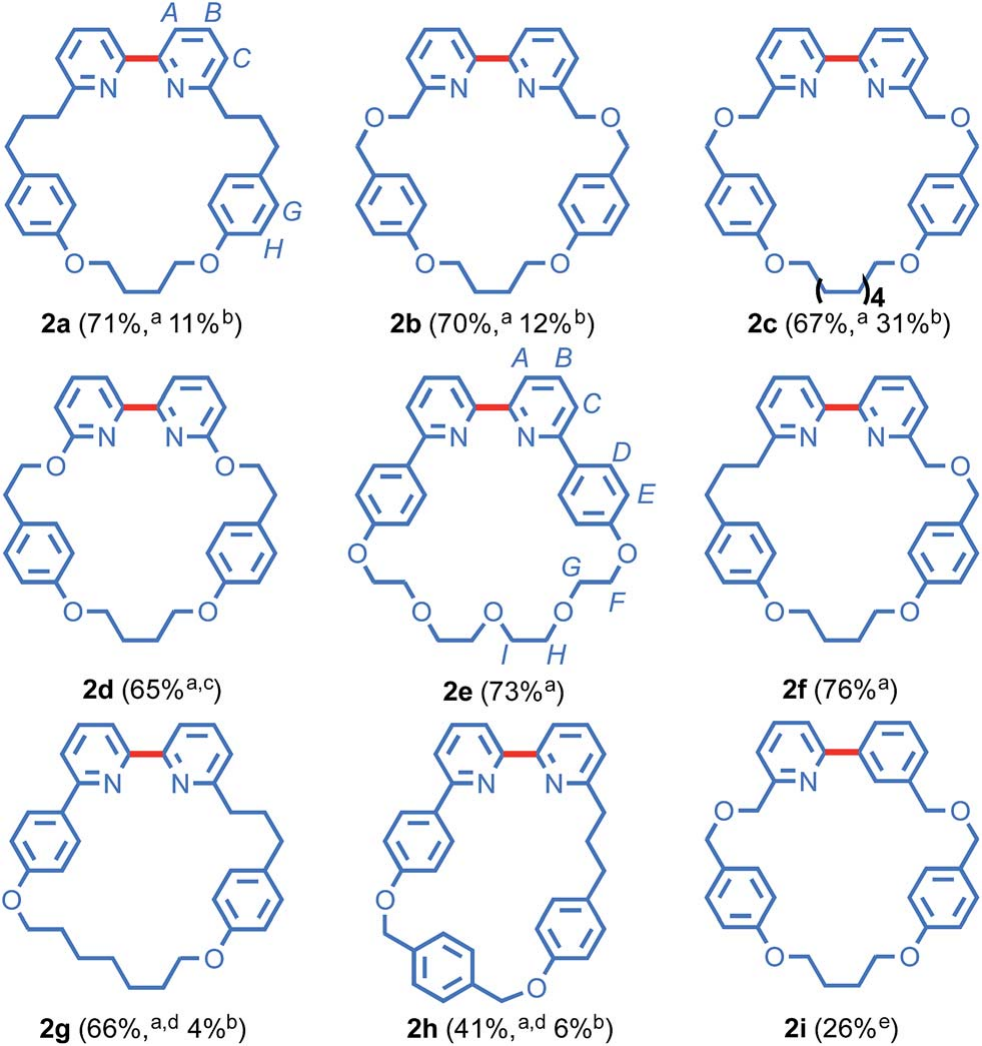
Synthesis of macrocycles **2** at 2 mmol scale. ^*a*^Reaction conditions as in Table 1, entry 8. ^*b*^Macrocyclisation yield of the best previous synthesis. ^*c*^*T* = 70 °C. ^*d*^Solvent = DMF–THF (3 : 1). ^*e*^Yield from Br/I precursor.

Under our optimised conditions, the preparative scale (2 mmol) reaction of **1a** provided macrocycle **2a** in an excellent 71% isolated yield (Fig. 1) and so we extended our investigation to a selection of previously reported and novel macrocycles. An ether link proximal to the pyridine unit did not affect the outcome; previously disclosed macrocycles **2b** and **2c** were produced in excellent yields, demonstrating that, unlike Williamson ether methods, the Ni-mediated cyclisation is relatively insensitive to ring size. Furthermore, when the dichloro analogue of **1b** was employed, macrocycle **2b** was produced in 56% yield, indicating that 2-chloro pyridines are also viable substrates. The reaction also tolerates heteroatom substitution on the pyridine ring (**2d**) although in this case an elevated temperature was required due to the lower reactivity of the more electron-rich substrate.^31^ Similarly, the reaction tolerates 6-aryl-substitution; sterically hindered macrocycle **2e**, a smaller analogue of Sauvage's iconic phenanthroline macrocycle,^3^ was produced in excellent 73% yield.

We then extended the Ni-mediated coupling to the synthesis of unsymmetrical bipyridine macrocycles, important intermediates in the synthesis of mechanically chiral molecules.14*c* Macrocycle **2f**, a hybrid of macrocycles **2a** and **2b** in which the two pyridine moieties are differentiated by a proximal ether unit, was produced in excellent 76% yield. Initial attempts to extend our approach to previously disclosed macrocycle **2g** were hampered by the poor solubility of **1g** in DMF. This was readily overcome by employing a DMF–THF solvent mixture, leading to **2g** in 66% yield, an order of magnitude improvement over the previous synthesis (4%). When the flexible alkyl chain of **2g** was replaced by a *p*-xylyl moiety to give **2h**, the yield of the macrocyclisation reaction fell to 41%, presumably due to the increased strain of the smaller, more rigid macrocycle. However, the yield of **2h** remains significantly higher than previous syntheses (6%; see ESI†) and demonstrates that the Ni-coupling can be used to generate more challenging structures.

Finally, we extended our approach to an aryl-pyridine cross-coupling. Initial attempts with the corresponding dibromo precursor delivered **2i** in a low 16% yield. However, replacing the bromo-benzene moiety with an iodo-benzene starting material raised the yield to 24%, suggesting that there is potential to extend our efficient Ni-mediated ring closure to other biaryl macrocyclic motifs in future.

The Ni-mediated synthesis of bipyridine macrocycles is obviously a dramatic improvement over established procedures in terms of simple reaction yield, by as much as an order of magnitude in some cases. Other key benefits of this methodology include: (i) the Ni-mediated coupling appears relatively insensitive to substrate structure (ring size, pyridine substituents) and tolerates simple modifications (temperature, solvent) where required; (ii) the reaction is capable of producing unsymmetrical bipyridine macrocycles such as **2f–i** through a formal intramolecular cross-coupling reaction; (iii) bis-halopyridine precursors **1** are readily available on scale using simple chemistry and commercially available precursors; (iv) isolation of the product is simplified both by the improved impurity profile of the crude product and by the use of pseudo-high dilution conditions.

**Scheme 1:**
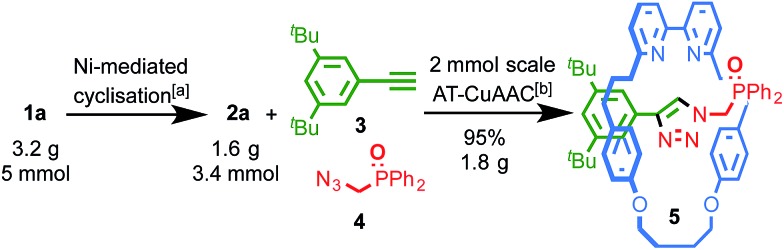
Large scale macrocyclisation and AT-CuAAC reactions. ^*a*^Conditions as Table 1, entry 8. ^*b*^[Cu(MeCN)_4_](PF_6_), N^*i*^Pr_2_Et, CH_2_Cl_2_–EtOH (1 : 1), 80 °C, 18 h.

This latter point renders the Ni-mediated macrocyclisation highly scalable; whereas previous syntheses of small bipyridine macrocycles **2a**, **2b**, **2g** and **2h** required between 3 and 9 litres of DMF per mmol of product, only ∼30 ml of DMF is required to produce each mmol of macrocycle using the method presented here. Using James' EMAC measure of reaction efficiency,^13^ a logarithmic scale taking into account the yield and reaction concentration, this corresponds to an increase in reaction efficiency from EMAC = 3 to 7. Furthermore, the excellent reaction yield is maintained across a range of scales; repeating the synthesis of **2a** (Scheme 1) with 5 mmol of **1a** delivered 1.6 g of macrocycle **2a** (69% isolated yield).

### Bipyridine macrocycles for the scalable synthesis of [2]rotaxanes in excellent yield

Macrocycles **2a–c** and **2g** are well precedented in the high yielding AT-CuAAC synthesis of interlocked molecules.9g,^14^,16b Pleasingly, preliminary investigations confirmed that macrocycles **2d–f** and **2h** are also excellent rotaxane precursors; under AT-CuAAC conditions with simple alkyne and azide coupling partners, **2d–f** and **2h** are effectively converted (>90% in all cases) to interlocked products (see ESI†).^32^

Furthermore, with significant quantities of macrocycles **2** in hand we were able for the first time to demonstrate the scalability of the AT-CuAAC reaction. Previous reports have been limited to small scales (typically 0.025 mmol) by the availability of the macrocycle component. Pleasingly, when the synthesis of interlocked phosphine oxide **5**, an advanced precursor of a stimuli-responsive interlocked Au catalyst,9*g* was carried out at an 80-fold larger scale using 0.96 g of macrocycle **2a** (2 mmol) the isolated yield of the interlocked product was increased relative to our previous report, delivering 1.8 g (95%) of **5** in a single synthetic operation.

### An efficient AT-CuAAC approach to Sauvage-type molecular shuttles

**Scheme 2:**
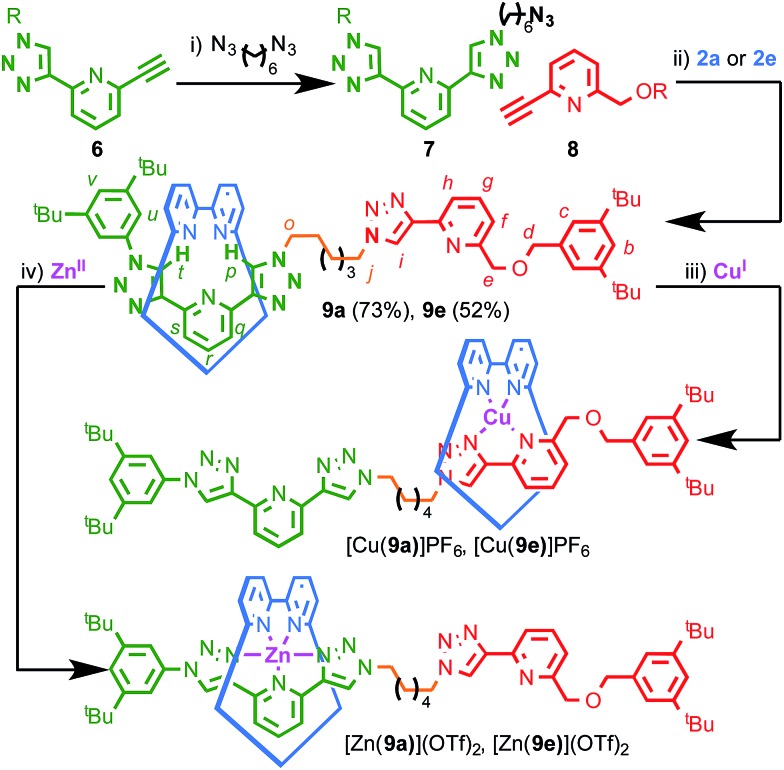
The AT-CuAAC synthesis and operation of bi-stable molecular shuttles **9**. Reagents and conditions: (i) [Cu(MeCN)_4_](PF_6_), CH_2_Cl_2_, rt; (ii) **2a** or **2e** [Cu(MeCN)_4_](PF_6_), N^i^Pr_2_Et, CH_2_Cl_2_, 80 °C; (iii) [Cu(MeCN)_4_](PF_6_), CDCl_3_; (iv) Zn(OTf)_2_, CDCl_3_. R = 3,5-di-^*t*^Bu-C_6_H_3_.

**Fig. 2:**
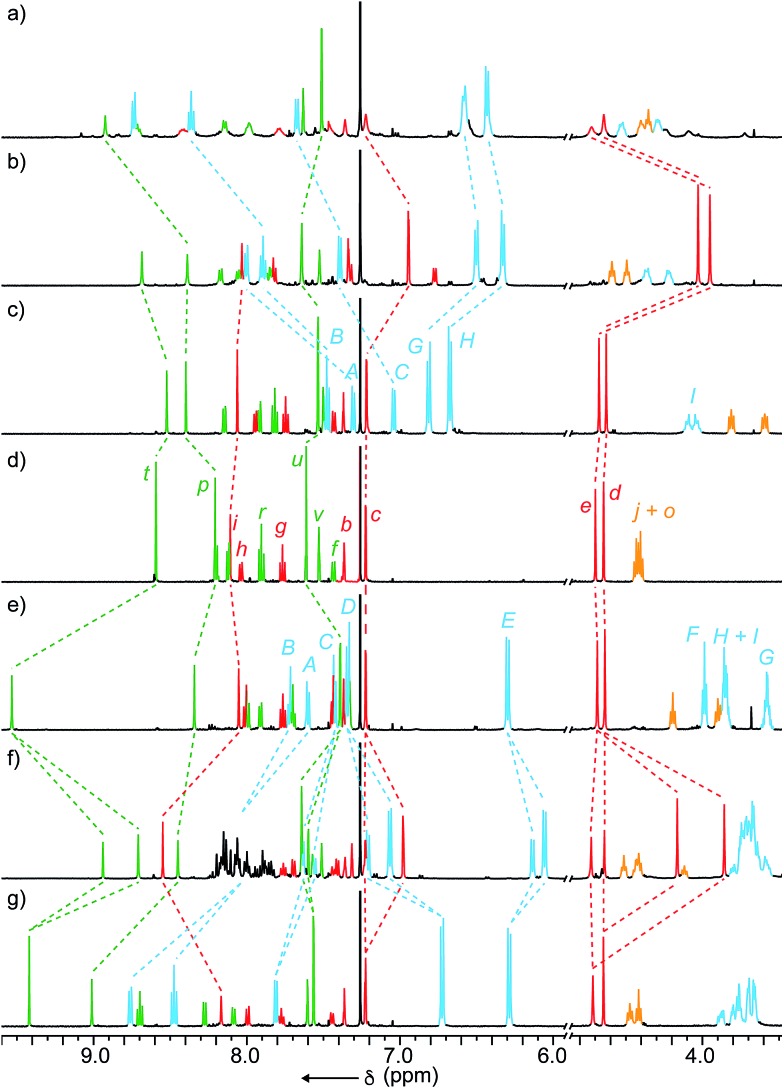
Partial ^1^H NMR (500 MHz, CDCl_3_) of (a) **9a** + Zn(OTf)_2_; (b) **9a** + [Cu(MeCN)_4_](PF_6_); (c) **9a**; (d) non-interlocked thread; (e) **9e**; (f) **9e** + [Cu(MeCN)_4_](PF_6_); (g) **9e** + Zn(OTf)_2_. Selected signals assigned with labels as in Fig. 1 (macrocycle) and Scheme 2 (thread).

Now that bipyridine macrocycles are far more readily available their application in more complex mechanically interlocked molecules and molecular machines is a far more attractive proposition. To capitalise on this, we developed a streamlined synthesis of two Sauvage-type molecular shuttles using macrocycles **2a** and **2e** and compared their behaviour. Such shuttles are usually based on phenanthroline macrocycles and a thread containing a terpyridine and a bipyridine station, assembled using a passive template threading approach.3*c* Replacing the phenanthroline macrocycle with its bipyridine analogue and the terpyridine and bipyridine stations with bistriazolylpyridine (btp) and monotriazolylpyridine (mtp) units respectively,^33^,^34^ greatly facilitates shuttle synthesis using an AT-CuAAC approach. Thus, shuttles **9a** and **9e** were synthesised in a concise manner from readily available ethynyl pyridine derivatives **6** and **8** through sequential CuAAC couplings of 1,6 bis-azido hexane, delivering **9a** and **9e** in 73% and 52% isolated yields respectively in the mechanical bond-forming step (Scheme 2).^35^ The corresponding non-interlocked thread was also synthesised for comparison. With shuttles **9** in hand, we examined their co-conformational behaviour by ^1^H and ROESY NMR (Fig. 2 and ESI respectively†).

Analysis of shuttles **9a** and **9e** by ^1^H NMR (Fig. 2c and e respectively) suggests that in both cases the macrocycle predominantly occupies the tridentate btp station; significant shifts of thread triazole protons H_*p*_ and H_*t*_ were observed relative to the non-interlocked thread (Fig. 2d), whereas protons associate with the bidentate mtp unit remain largely unaffected by the formation of the mechanical bond. ROESY NMR analysis supports this assignment with cross-peaks observed between macrocycle protons and H_*q*_, H_*r*_ and H_*s*_ of the thread. Given the significant shifts of protons H_*p*_ and H_*t*_ and our previous observation of C–H···N hydrogen bonding in AT-CuAAC derived rotaxanes,14*a* the localisation of the macrocycle over the btp station in rotaxanes **9** is tentatively attributed to the presence of two C–H···N hydrogen-bonding interactions compared with only one in the case of the mtp station.

Next we investigated shuttles **9** in the presence of diamagnetic metal ions Cu^I^ and Zn^II^ in order to monitor their co-conformational behaviour by NMR, as previously reported by Sauvage in the case of phenanthroline derived shuttles.3c,^36^ Addition of [Cu(MeCN)_4_](PF_6_) to **9a** led to large changes in the ^1^H NMR (Fig. 2b) consistent with the expected shuttling of the macrocycle to the bidentate mtp station to form the preferred tetradentate coordination site for Cu^I^; protons H_*c*_, H_*d*_ and H_*e*_ are shielded significantly and ROESY cross-peaks are observed between mtp triazole proton H_*i*_ of the thread and alkyl proton H_*I*_ of the macrocycle. Replacing Cu^I^ with Zn^II^ resulted in a new species consistent with the expected complex in which the macrocycle occupies the btp station, providing a pentadentate binding site for Zn; signals associated with the mtp station return to values similar to that of the thread and cross-peaks are observed between the triazole protons H_*p*_ and H_*t*_ of the btp station and H_*I*_ of the macrocycle. Thus it appears that shuttle **9a** behaves as a simple bipyridine–mtp/btp analogue of Sauvage's phenanthroline–bipyridine/terpyridine shuttle.3*c*

The behaviour of shuttle **9e** proved to be more complicated. Surprisingly, addition of Cu^I^ led to the formation of two new species in an approximate 2 : 1 ratio (Fig. 2f). Two-dimensional exchange spectroscopy (2D-EXSY)^37^ confirmed that these co-conformational isomers are in slow exchange on the NMR timescale with a unimolecular rate constant at room temperature of the order of 10^–3^ s^–1^ corresponding to an activation barrier of ∼21 kcal mol^–1^. The major co-conformation was assigned as that in which the macrocycle, as initially expected, is localised over the bidentate mtp station, based on the shielding of signals corresponding to H_*e*_, H_*d*_ and H_*c*_, and ROESY cross-peaks between H_*D*_ and H_*E*_ of the macrocycle and H_*e*_ and H_*d*_ of the thread.

Similar analysis confirmed that the minor co-conformation is that in which the macrocycle unexpectedly coordinates the Cu^I^ ion at the nominally tridentate btp station, initially suggestive of a five-coordinate Cu centre. However, Schmittel and co-workers have previously reported the formation of a heteroleptic Cu^I^ complex derived from one tridentate and one bidentate ligand in which the metal ion adopts the expected four-coordinate geometry, with the fifth donor not involved in binding to the metal ion.^38^ Although this phenomenon requires further investigation, we tentatively suggest that similar behaviour may account for the minor co-conformation of [Cu(**9a**)](PF_6_), with subtle differences in the secondary interactions (C–H···π, π···π) between macrocycles **2a** or **2e** and the thread accounting for the differences observed.

Finally, replacing Cu^I^ with Zn^II^ led to a much simpler outcome; a single new species was observed with ^1^H and ROESY NMR confirming that, in the case of the Zn^II^ complex [Zn(**9e**)](OTf)_2_, the macrocycle was located predominantly on the tridentate btp station, as expected (Fig. 2g).

## Conclusions

In conclusion we have presented an extremely efficient, scalable and general Ni-mediated method for the synthesis of small bipyridine macrocycles for the AT-CuAAC reaction in high yield from readily available precursors under pseudo high dilution conditions. Although this preliminary study has focussed on macrocycles similar to those used previously in the AT-CuAAC reaction, Ni-mediated couplings are typically tolerant to a wide range of functional groups24*b* and investigations are currently underway to determine the wider substrate scope. These now readily available macrocycles are proven versatile intermediates for the synthesis of interlocked molecules in excellent yield using both active^14^,16b,18a–e and passive^39^ template methods. Given the scalability of both the macrocycle synthesis and the mechanical bond forming step demonstrated here, and the clear potential to extend the approach to more complex molecules, the AT-CuAAC reaction mediated by bipyridine macrocycles clearly has a bright future in the synthesis of interlocked architectures for a variety of applications.

## Supplementary Material

Supplementary information

Crystal structure data
