# Correction: High yielding synthesis of 2,2′-bipyridine macrocycles, versatile intermediates in the synthesis of rotaxanes

**DOI:** 10.1039/c6sc90027e

**Published:** 2016-04-21

**Authors:** J. E. M. Lewis, R. J. Bordoli, M. Denis, C. J. Fletcher, M. Galli, E. A. Neal, E. M. Rochette, S. M. Goldup

**Affiliations:** a Chemistry , University of Southampton , Highfield , Southampton , SO17 1BJ , UK . Email: s.goldup@soton.ac.uk; b School of Biological and Chemical Sciences , Queen Mary University of London , Mile End Road , London , E1 4NS , UK

## Abstract

Correction for ‘High yielding synthesis of 2,2′-bipyridine macrocycles, versatile intermediates in the synthesis of rotaxanes’ by J. E. M. Lewis *et al.*, *Chem. Sci.*, 2016, DOI: 10.1039/c6sc00011h.



## 

The support of the Leverhulme Trust (RPG-2014-084) was regrettably omitted by the authors in the published manuscript. The updated acknowledgements section is as follows:

The Royal Society of Chemistry apologises for this error and any consequent inconvenience to authors and readers.

## Acknowledgements

We thank Fluorochem for the gift of reagents, the EPSRC National Mass Spectrometry Service for HRMS analysis and the EPSRC (EP/L016621/1), Leverhulme Trust (RPG-2014-084), Queen Mary, University of London and the University of Southampton for funding. JEML is a European Commission Marie Skłodowska-Curie Fellow. SMG is a Royal Society Research Fellow. This project has received funding from the European Union's Horizon 2020 research and innovation programme under the Marie Sklodowska-Curie grant agreement No. 660731.

