# Supplementary material for: High yielding synthesis of 2,2′-bipyridine macrocycles, versatile intermediates in the synthesis of rotaxanes
Source: Chem Sci. 2016 Jan 27;7(5):3154–61. doi: 10.1039/c6sc00011h (PMC6005271; doi:10.1039/c6sc00011h)
Supplement: Supplementary file 1 [file SC-007-C6SC00011H-s001.pdf]

Electronic Supplementary Material (ESI) for Chemical Science.
This journal is © The Royal Society of Chemistry 2016

Electronic Supporting Information

High Yielding Synthesis of 2,2`-Bipyridine Macrocycles, Versatile Intermediates in the Synthesis of Rotaxanes

James E. M. Lewis,[1] Robert J. Bordoli,[2] Mathieu Denis,[1] Catherine J. Fletcher,[1] Marzia Galli,[1] Edward A. Neal,[2] Elise M. Rochette,[1] and Stephen M. Goldup[1]*

[1]Chemistry, University of Southampton, Highfield, Southampton, SO17 1BJ, UK

[2]School of Biological and Chemical Science, Queen Mary University of London, Mile End Road, London, E1 4NS, UK

Email: s.goldup@soton.ac.uk

Table of Contents

1. General Experimental 3

2. Experimental Procedures 4

3. Synthesis and shuttling experiments of rotaxanes 9a and 9e 32

4. Exchange Spectroscopy (EXSY) Investigation of Shuttling Process of [Cu(9e)](PF_6) 40

5. Graphical NMR Data including 2D Spectra 44

Figure S186 ^{1}H ROESY NMR ($CDCl_3$, 400 MHz) of 9a. 141

Figure S192 ^{1}H ROESY NMR ($CDCl_3$, 500 MHz) of [Cu(9a)](PF_6). 145

Figure S198 ^{1}H ROESY NMR ($CDCl_3$, 500 MHz) of [Zn(9a)]$(OTf)_2$. 149

Figure S204 ^{1}H ROESY NMR ($CDCl_3$, 500 MHz) of 9e. 152

Figure S210 ^{1}H ROESY NMR ($CDCl_3$, 500 MHz) of [Cu(9e)](PF_6). 155

Figure S216 ^{1}H ROESY NMR ($CDCl_3$, 500 MHz) of [Zn(9e)]$(OTf)_2$. 158

6. Crystalographic Data 159

7. References 166

1. General Experimental

Unless otherwise stated, all reagents were purchased from commercial sources and used without further purification. All reactions were carried out under an atmosphere of N_2 using anhydrous solvents, purchased from commercial sources, unless otherwise stated. Petrol refers to the fraction of petroleum ether boiling in the range 40-60 °C. IPA refers to isopropyl alcohol. EDTA-NH_3 solution refers to an aqueous solution of NH_3 (17% w/w) saturated with sodium-ethylenediaminetetraacetate. Flash column chromatography was performed using Biotage Isolera-4 automated chromatography system, employing Biotage SNAP or ZIP cartridges. Analytical TLC was performed on precoated silica gel plates (0.25 mm thick, 60F254, Merck, Germany) and observed under UV light. NMR spectra were recorded on Bruker AV400 or AV500 instruments, at a constant temperature of 298 K. Chemical shifts are reported in parts per million from low to high field and referenced to residual solvent. Coupling constants (*J*) are reported in Hertz (Hz). Standard abbreviations indicating multiplicity were used as follows: m = multiplet, quint = quintet, q = quartet, t = triplet, d = doublet, s = singlet, app. = apparent, br = broad. All melting points were determined using a Griffin melting point apparatus and are uncorrected. Low resolution mass spectrometry was carried out using a Waters TQD mass spectrometer equipped with a triple quadrupole analyser with UHPLC injection [BEH C18 column; MeCN-hexane gradient {0.2% formic acid}]. High resolution mass spectrometry was carried out by the mass spectrometry services at the University of Southampton with samples were analysed using a MaXis (Bruker Daltonics, Bremen, Germany) mass spectrometer equipped with a Time of Flight (TOF) analyser. Samples were introduced to the mass spectrometer *via* a Dionex Ultimate 3000 autosampler and uHPLC pump. Gradient 20% acetonitrile (0.2% formic acid) to 100% acetonitrile (0.2% formic acid) in five minutes at 0.6 mL min. Column, Acquity UPLC BEH C18 (Waters) 1.7 micron 50 × 2.1mm.

2-Bromo-6-(3-(4-methoxyphenyl)propyl)pyridine[1] and 2-bromo-6-(4-methoxyphenyl)pyridine[2] were made according to our previously reported methods. 3,5-Di-*tert*-butylethynylbenzene (**3**)[3], (azidomethyl)diphenylphosphine oxide (**4**)[4], 3,5-di-*tert*-butylazidobenzene (**S15**)[5] and 3,5-di-*tert*-butylbenzylazide (**S16**)[6] were made according to literature procedures. Spectra for previously reported macrocycles **2a**,[1] **2b**,[7] **2c**,[8] and **2g**[2] and rotaxane **5**[9] were consistent with previous literature reports.

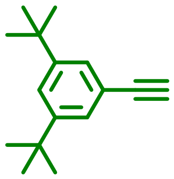

3

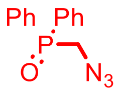

4

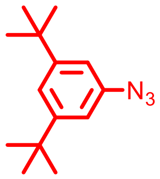

S15

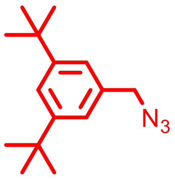

S16

2. Experimental Procedures

General Macrocyclisation Procedure:

[$Ni(PPh_3)_2Br_2$] (1.49 g, 2.00 mmol, 1 eq.), PPh_3 (1.05 g, 4.00 mmol, 2 eq.), Mn (1.10 g, 2.00 mmol, 10 eq.) and NEt_4I (0.514 g, 2.00 mmol, 1 eq.) in DMF (20 mL) were sonicated for 10 min, followed by stirring at 50 °C for 1 h. To this catalyst mixture was added **1** (2.00 mmol, 1 eq.) in DMF (20 mL) *via* syringe over 4 h (except where noted below), followed by additional stirring of the reaction for 1 h. To the cooled reaction was added CH_2Cl_2 (100 mL) and EDTA-NH_3 solution (100 mL). After filtering through a pad of Celite the organic phase was washed with water (2 × 100 mL) and brine (100 mL), and the combined aqueous phases extracted with CH_2Cl_2 (50 mL). The combined organic phases were dried ($MgSO_4$), filtered and the solvent removed *in vacuo*. The crude product was immediately loaded onto a pad of silica and eluted with 1:1 petrol/CH_2Cl_2 to remove PPh_3, followed by 10-20% MeCN in 1:1 petrol/CH_2Cl_2 to elute the product. Subsequent purification by column chromatography yielded **2** as white to yellow solids.

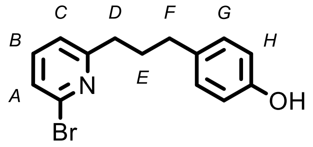

2-Bromo-6-(3-(4-hydroxyphenyl)propyl)pyridine (S1):

A solution of 2-bromo-6-(3-(4-methoxyphenyl)propyl)pyridine (12.25 g, 40.00 mmol, 1 eq.) in 48% aqueous HBr (40 mL) was stirred at reflux overnight. The cooled reaction mixture was diluted with H_2O (40 mL) and neutralized using 10 M $NaOH_{(aq)}$ solution. The product was extracted with CH_2Cl_2 (3 × 50 mL) and the combined organic layers were dried ($MgSO_4$), filtered and the solvent removed *in vacuo.* After purification by column chromatography (CH_2Cl_2 with 0% to 5% Et_2O gradient), **S1** (11.22 g, 96%) was obtained as an oil that solidified on standing. M.p. 83-85 °C. ^{1}H NMR ($CDCl_3$, 400 MHz) δ: 7.44 (app. t, J = 7.7, 1H, H_B), 7.30 (dd, J = 7.9, 0.8, 1H, H_A), 7.08 (dd, J = 7.6, 0.8, 1H, H_C), 7.03 (d, J = 8.5, 2H, H_G), 6.75 (d, J = 8.5, 2H, H_H), 5.13 (s, 1H, H_{OH}), 2.83-2.72 (m, 2H, H_D), 2.59 (t, J = 7.7, 2H, H_F), 2.07-1.92 (m, 2H, H_E). ^{13}C NMR ($CDCl_3$, 100 MHz) δ: 164.0, 153.9, 141.7, 138.8, 134.1, 129.6, 125.5, 121.7, 115.4, 37.5, 34.7, 31.7. HR-ESI-MS (MeCN): m/z = 292.0332 $[M+H]^+$ calc. 292.0338.

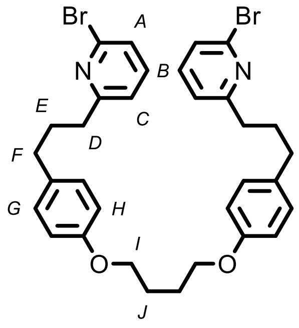

Macrocycle 2a Precursor (1a):

To a solution of compound **S1** (4.83 g, 16.6 mmol, 2 eq.) in DMF (83 mL) was added K_2CO_3 (9.18 g, 66.4 mmol, 8 eq.) and 1,4-dibromobutane (1.79 g, 8.30 mmol, 1 eq.) and the reaction mixture stirred overnight at 80 °C. The suspension was diluted with of CH_2Cl_2 (500 mL) and washed with H_2O (500 mL) and brine (500 mL). The aqueous extracts were extracted with CH_2Cl_2 (200 mL) and the combined organic extracts were dried (Na_2SO_4) and the solvent removed *in vacuo*. After purification by column chromatography (1:1 petrol/DCM with a 0 to 10% gradient of MeCN) gave the product as an off-white solid (3.71 g, 70%). M.p. 87-89 °C. ^{1}H NMR ($CDCl_3$, 400 MHz) δ: 7.43 (app. t, J = 7.7, 2H, H_B), 7.29 (d, J = 7.8, 2H, H_A), 7.10-7.06 (m, 6H, H_C, H_G), 6.82 (d, J = 8.6, 4H, H_H), 4.01 (t, J = 5.2, 4H, H_I), 2.78 (t, J = 7.8, 4H, H_D), 2.61 (t, J = 7.6, 4H, H_F), 2.05-1.95 (m, 8H, H_E, H_J). ^{13}C NMR ($CDCl_3$, 100 MHz) δ: 163.9, 157.3, 141.7, 138.7, 134.0, 129.5, 125.4, 121.6, 114.5, 67.6, 37.6, 34.7, 31.7, 26.2. HR-ESI-MS (CH_3CN): m/z = 637.1074 $[M+H]^+$ calc. 637.1060.

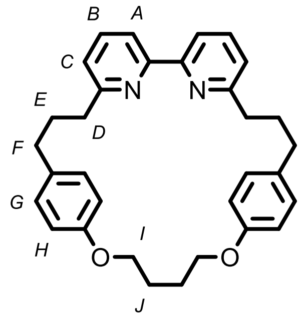

Macrocycle 2a:

Prepared according to general procedure with **1a** (1.28 g, 2.00 mmol). Purification by column chromatography (1:1 petrol/CH_2Cl_2 with a 0 to 5% gradient of EtOAc) afforded **2a** (0.683 g, 71%) as a white solid. ^{1}H NMR ($CDCl_3$, 400 MHz) δ: 7.64-7.57 (m, 4H, H_A, H_B), 7.12 (dd, J = 1.4, 7.2, 2H, H_C), 7.02 (d, J = 8.6, 4H, H_G), 6.68 (d, J = 8.6, 4H, H_H), 4.03 (t, J = 5.8, 4H, H_I), 2.91-2.87 (m, 4H, H_D), 2.66 (t, J = 7.0, 4H, H_F), 2.17-2.09 (m, 4H, H_E), 1.94 (t, J = 5.8, 4H, H_J). ^{13}C NMR ($CDCl_3$, 100 MHz) δ: 162.5, 157.0, 157.0, 136.7, 134.2, 129.7, 122.1, 119.6, 114.8, 67.5, 37.7, 34.8, 31.0, 25.4.

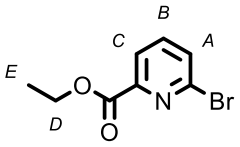

Ethyl-6-bromopicolinate (S2):

A stirring solution of 6-bromo-2-picolinic acid (101 g, 0.500 mol) and H_2SO_4 (4 mL) in EtOH (500 mL) was heated at reflux for 1 h. The cooled reaction solution was reduced *in vacuo*, dissolved in $CHCl_3$ (400 mL), washed with 1M $NaOH_{(aq)}$ (400 mL), dried ($MgSO_4$) and concentrated *in vacuo* to afford **S2** (92.8 g, 81%) as a white, low-melting solid that was used without further purification. ^1H NMR (400 MHz, $CDCl_3$) δ: 8.06 (d, J = 7.3, 1H, H_A), 7.63-7.71 (m, 2H, H_B and H_C), 4.46 (q, J = 7.2, 2H, H_D), 1.42 (t, J = 7.2, 3H, H_E). ^{13}C NMR (100 MHz, $CDCl_3$) δ 164.0, 149.3, 142.3, 139.2, 131.8, 124.1, 62.4, 14.4. HR-ESI-MS (MeCN): m/z = 251.9632 $[M+Na]^+$ calc. 251.9631.

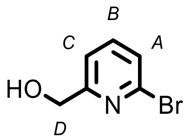

2-Bromo-6-hydroxymethylpyridine (S3):

To a solution of **S2** (92.8 g, 0.405 mol) in EtOH (500 mL) in a water bath was added $NaBH_4$ (18.9 g, 0.500 mol, 1.2 eq.) portionwise over 1 h. The solution was stirred for 8 h then H_2O (500 mL) was added slowly to quench the reaction before the EtOH was removed *in vacuo*. The suspension was extracted with CH_2Cl_2 (3 × 500 mL), dried ($MgSO_4$) and concentrated *in vacuo* to afford **S3** (72.2 g, 95%) as a white, low-melting solid that was used without further purification. ^1H NMR ($CDCl_3$, 400 MHz) δ: 7.55 (app. t, J = 7.7, 1H, H_B), 7.39 (dd, J = 7.8, 0.5, 1H, H_A), 7.28 (dd, J = 7.6, 0.6, 1H, H_C), 4.74 (s, 2H, H_D), 3.12 (s, 1H, H_{OH}). ^{13}C NMR ($CDCl_3$, 100 MHz) δ: 161.3, 141.6, 139.2, 126.8, 119.4, 64.3. HR-ESI-MS (CH_3CN): m/z = 209.9524 $[M+Na]^+$ calc. 209.9525.

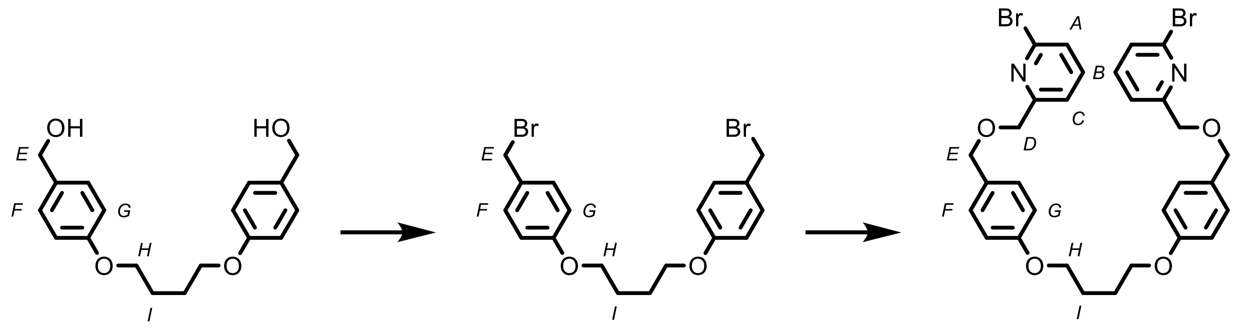

Macrocycle 2b Precursor (1b):

A solution of 4-hydroxybenzyl alcohol (25.0 g, 0.201 mol, 2 eq.), 1,4-dibromobutane (21.7 g, 0.101 mol, 1 eq.) and K_2CO_3 (55.7 g, 0.403 mol, 4 eq.) in MeCN (1000 mL) was stirred at reflux for 18 h. The solution was reduced to ~250 mL *in vacuo* and H_2O (1250 mL) added resulting in a beige precipitate, which was collected by filtration to afford wet crude 1,4-di(4-(hydroxymethyl)phenoxy)butane. ^{1}H NMR (400 MHz, $CDCl_3$) δ: 7.26-7.32 (m, 4H, H_F), 6.89 (d, J = 8.5, 4H, H_G), 4.62 (d, J = 5.7, 4H, H_E), 3.96-4.11 (m, 4H, H_H), 1.91-2.02 (m, 4H, H_I).

The crude 1,4-di(4-(hydroxymethyl)phenoxy)butane was then suspended in CH_2Cl_2 (250 mL) and HBr (48% w/w aq., 250 mL) and stirred at RT for 3 h until the solid had dissolved. The solution was then diluted with H_2O (600 mL) and extracted with $CHCl_3$ (3 × 300 mL). The combined organic layers were dried ($MgSO_4$), filtered and concentrated *in vacuo* to afford crude 1,4-di(4-(bromomethyl)phenoxy)butane as a pale yellow wax. ^{1}H NMR (400 MHz, $CDCl_3$) δ: 7.31 (d, J = 8.6, 4H, H_F), 6.85 (d, J = 8.6, 4H, H_G), 4.50 (s, 4H, H_E), 3.98-4.09 (m, 4H, H_H), 1.92-2.02 (m, 4H, H_I).

To a solution of 2-bromo-6-hydroxymethylpyridine (41.7 g, 0.222 mol, 2.2 eq.) in DMF (500 mL) at 0 °C was added 60% NaH (13.3 g, 0.332 mol, 3.3 eq.) portionwise over 15 min with stirring. The crude 1,4-di(4-(bromomethyl)phenoxy)butane was added in one portion and the solution stirred for 1 h, allowing to warm to RT. H_2O (500 mL) was added cautiously to quench the reaction, followed by addition of further H_2O (1500 mL) resulting in a yellow precipitate which was collected by filtration. This was dissolved in $CHCl_3$ (500 mL), washed with brine (500 mL), dried ($MgSO_4$), filtered and the solvent removed *in vacuo*. This was purified by column chromatography on silica (2:11:11 EtOAc:Petrol:CH_2Cl_2) to give **1b** (45.6 g, 70% over 3 steps based on 4-hydroxybenzyl alcohol) as an off-white solid. M.p. 58-60 °C. ^{1}H NMR (400 MHz, $CDCl_3$) δ: 7.54 (dd, J = 7.8, 7.6, 2H, H_B), 7.46 (d, J = 7.6, 2H, H_A), 7.36 (d, J = 7.8, 2H, H_C), 7.29 (d, J = 8.0, 4H, H_F), 6.89 (d, J = 8.0, 4H, H_G), 4.62 (s, 4H, H_D), 4.57 (s, 4H, H_E), 3.95-4.10 (m, 4H, H_H), 1.89-2.03 (m, 4H, H_I), 1.45-1.60 (m, 4H, H_J). ^{13}C NMR (100 MHz, $CDCl_3$) δ: 160.7, 159.0, 141.4, 139.9, 129.9, 129.7, 126.7, 120.1, 114.7, 73.0, 72.1, 67.7, 26.2. HR-ESI-MS (CH_3CN) m/z = 663.0460 $[M+Na]^+$ calc. 663.0465.

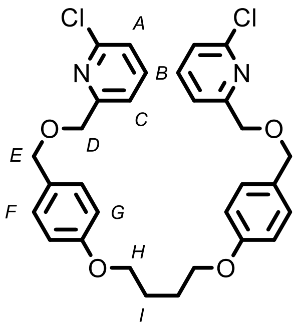

Macrocycle 2b Dichloro-precursor (S4):

S4 was prepared as for **1b**: 2-(6-chloropyridine)methanol (1.58 g, 11.0 mmol, 2.2 eq.), 60% NaH (0.660 g, 16.5 mmol, 3.3 eq.) and 1,4-di(4-(bromomethyl)phenoxy)butane (2.14 g, 5.00 mmol, 1 eq.) in DMF (25 mL). After purification by column chromatography on silica (1:1 petrol/CH_2Cl_2 with a 0 to 10% gradient of EtOAc) the product was obtained as a white solid (1.19 g, 43%). M.p. 84-86 °C. ^{1}H NMR ($CDCl_3$, 400 MHz) δ: 7.65 (app. t, J = 7.8, 2H, H_B), 7.43 (d, J = 7.6, 2H, H_A), 7.29 (d, J = 8.6, 4H, H_F), 7.21 (d, J = 7.9, 2H, H_C), 6.89 (d, J = 8.6, 4H, H_G), 4.62 (s, 4H, H_D), 4.57 (s, 4H, H_E), 4.05-4.02 (m, 4H, H_H), 1.99-1.96 (m, 4H, H_I). ^{13}C NMR ($CDCl_3$, 100 MHz) δ: 160.0, 158.9, 150.7, 139.4, 129.9, 129.7, 122.9, 119.7, 114.6, 72.9, 72.2, 67.6, 26.1. HR-ESI-MS (MeCN): m/z = 575.1481 $[M+Na]^+$ calc. 575.1475.

Macrocycle 2b:

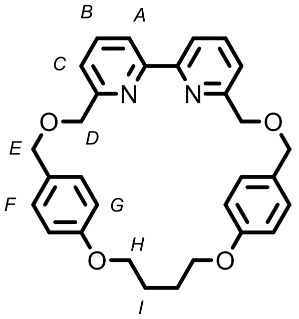

Prepared according to general procedure with **1b** (1.28 g, 2.00 mmol). Purification by column chromatography (petrol with a 0 to 50% gradient of Et_2O) afforded **2b** (0.685 g, 70%) as a white solid. ^{1}H NMR ($CDCl_3$, 400 MHz) δ: 7.75-7.70 (m, 4H, H_A, H_B), 7.32 (dd, J = 6.1, 2.6, 2H, H_C), 7.24 (d, J = 8.7, 4H, H_F), 6.77 (d, J = 8.7, 4H, H_G), 4.65 (s, 2H, H_D), 4.54 (s, 2H, H_E), 4.03 (t, J = 6.0, 4H, H_H), 1.98-1.95 (m, 4H, H_I). ^{13}C NMR ($CDCl_3$, 100 MHz) δ: 159.2, 158.8, 156.0, 137.2, 130.7, 129.7, 121.5, 120.6, 114.8, 72.8, 72.0, 67.4, 25.4.

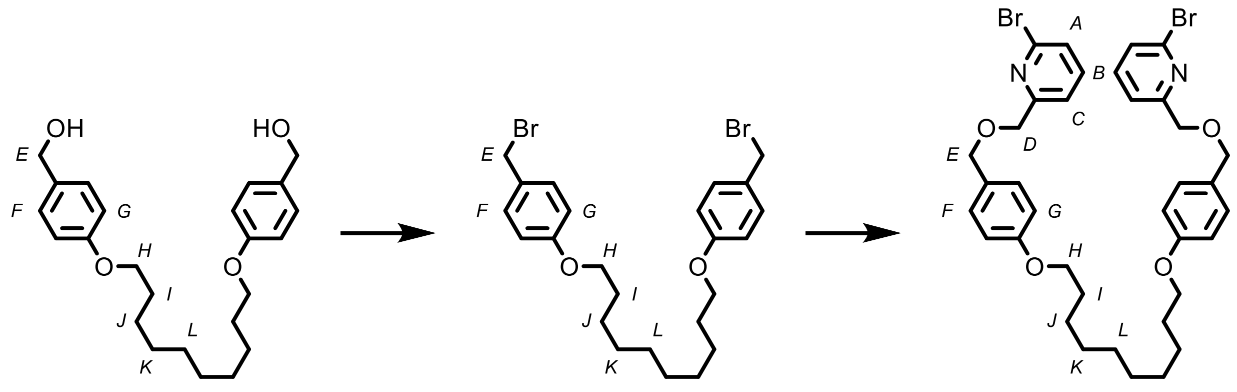

Macrocycle 2c Precursor (1c):

A solution of 4-hydroxybenzyl alcohol (10.00 g, 80.55 mmol, 2 eq.), 1,10-dibromodecane (12.09 g, 40.27 mmol, 1 eq.) and K_2CO_3 (22.26 g, 101.1 mmol, 4 eq.) in MeCN (400 mL) was stirred at reflux for 18 h. The solution was reduced to ~100 mL *in vacuo* and H_2O (500 mL) added resulting in a beige precipitate, which was collected by filtration to afford wet crude 1,10-di(4-(hydroxymethyl)phenoxy)decane. ^1H NMR (400 MHz, $CDCl_3$) δ: 7.24-7.30 (m, 4H, H_F), 6.91 (d, J = 8.4, 4H, H_G), 4.64 (d, J = 5.6, 4H, H_E), 3.98 (t, J = 6.6, 4H, H_H), 1.80 (tt, J = 7.2, 6.6, 4H, H_I), 1.28-1.60 (m, 12H, H_J, H_K and H_L).

The crude 1,10-di(4-(hydroxymethyl)phenoxy)decane was then suspended in CH_2Cl_2 (100 mL) and HBr (48% w/w aq., 100 mL) and stirred at RT for 3 h until the solid had dissolved. The solution was then diluted with H_2O (300 mL) and extracted twice with $CHCl_3$ (2 × 300 mL). The combined organic layers were dried ($MgSO_4$), filtered and concentrated *in vacuo* to afford crude 1,10-di(4-(bromomethyl)phenoxy)decane as a pale yellow wax. ^1H NMR (400 MHz, $CDCl_3$) δ: 7.31 (d, J = 8.6, 4H, H_F), 6.86 (d, J = 8.6, 4H, H_G), 4.51 (s, 4H, H_E), 3.96 (t, J = 6.6, 4H, H_H), 1.78 (tt, J = 7.4, 6.6, 4H, H_I), 1.24-1.55 (m, 12H, H_J, H_K and H_L).

To a solution of 2-bromo-6-hydroxymethylpyridine (16.65 g, 88.60 mmol, 2.2 eq.) in DMF (200 mL) at 0 °C was added 60% NaH (5.32 g, 133 mmol, 3.3 eq.) portionwise over 15 min with stirring. The crude 1,10-di(4-(bromomethyl)phenoxy)decane was added in one portion and the solution stirred for 1 h, allowing to warm to RT. H_2O (200 mL) was added cautiously to quench the reaction, followed by addition of further H_2O (600 mL) resulting in a yellow precipitate which was collected by filtration. This was dissolved in $CHCl_3$ (250 mL), washed with brine (250 mL), dried ($MgSO_4$), filtered and the solvent removed *in vacuo*. This was purified by column chromatography on silica (2:11:11 EtOAc:Petrol:CH_2Cl_2) to give **1c** (6.60 g, 23%) as an off-white solid. M.p. 65-67 °C. ^1H NMR (400 MHz, $CDCl_3$) δ: 7.54 (dd, J = 7.8, 7.6, 2H, H_B), 7.46 (d, J = 7.6, 2H, H_A), 7.36 (d, J = 7.8, 2H, H_C), 7.28 (d, J = 8.2, 4H, H_F), 6.88 (d, J = 8.2, 4H, H_G), 4.62 (s, 4H, H_D), 4.56 (s, 4H, H_E), 3.95 (t, 4H, J = 6.5, H_H), 1.77 (tt, 4H, J = 7.0, 6.5, H_I), 1.23-1.52 (m, 12H, H_J, H_K and H_L). ^{13}C NMR (100 MHz,

$CDCl_3$) δ: 160.7, 159.1, 141.4, 139.1, 129.7, 129.6, 126.6, 120.1, 114.6, 73.0, 72.1, 68.2, 29.6, 29.5, 29.4, 26.2. HR-ESI-MS (CH_3CN) *m/z* = 747.1400 $[M+Na]^+$ calc. 747.1404.

Macrocycle 2c:

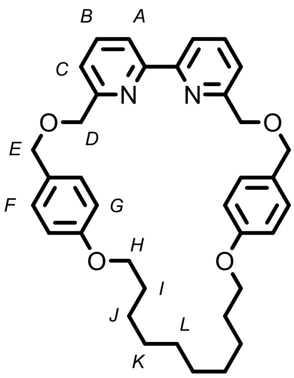

Prepared according to general procedure with **1c** (1.45 g, 2.00 mmol). Purification by column chromatography (1:1 petrol/CH_2Cl_2 with a 0 to 15% gradient of EtOAc) afforded **2c** (0.756 g, 67%) as an off-white solid. ^{1}H NMR ($CDCl_3$, 400 MHz) δ: 8.04 (d, J = 7.5, 2H, H_A), 7.72 (app. t, J = 7.8, 2H, H_B), 7.40 (d, J = 7.6, 2H, H_C), 7.19 (d, J = 8.6, 4H, H_F), 6.76 (d, J = 8.7, 4H, H_G), 4.66 (s, 2H, H_D), 4.65 (s, 4H, H_E), 3.91 (t, J = 6.5, 4H, H_H), 1.73-1.66 (m, 4H, H_I), 1.40-1.37 (m, 4H, H_J), 1.29-1.26 (m, 8H, H_K, H_L). ^{13}C NMR ($CDCl_3$, 100 MHz) δ: 158.8, 158.7, 155.9, 137.3, 130.0, 129.9, 121.5, 120.1, 114.6, 72.7, 72.4, 67.9, 29.4, 29.1, 28.9, 25.9.

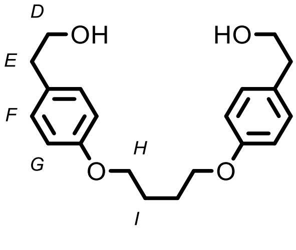

1,4-bis(4-(2-hydroxyethyl)phenoxy)butane (S5):

2-(4-Hydroxyphenyl)ethanol (3.04 g, 22.0 mmol, 2.2 eq.), 1,4-dibromobutane (2.16 g, 10.0 mmol, 1 eq.) and K_2CO_3 (11.1 g, 80.0 mmol, 8 eq.) were stirred in DMF (50 mL) at 80 °C for 18 h. CH_2Cl_2 (250 mL) added and washed with H_2O (200 mL) and brine (200 mL). Aqueous layers extracted with CH_2Cl_2 (100 mL) and combined organic layers dried ($MgSO_4$), filtered and solvent removed *in vacuo*. The resultant solid was suspended in acetone (30 mL), filtered and dried *in vacuo* to give **S4** (2.50 g, 76%) as a white solid. M.p. 140-142 °C. ^{1}H NMR ($CDCl_3$, 400 MHz) δ: 7.14 (d, J = 8.6, 4H, H_F), 6.85 (d, J = 8.6, 4H, H_G), 4.03-4.00 (m, 4H, H_H), 3.82 (app. q, J = 6.4, 4H, H_D), 2.81 (t, J = 6.5, 4H, H_E), 1.98-1.95 (m, 4H, H_I), 1.38 (t, J = 5.9, 2H, H_{OH}). ^{13}C NMR ($CDCl_3$, 100 MHz) δ: 157.9, 130.5, 130.1, 114.8, 67.6, 64.0, 38.4, 26.2. HR-ESI-MS (CH_3CN) *m/z* = 353.1725 $[M+Na]^+$ calc. 353.1723.

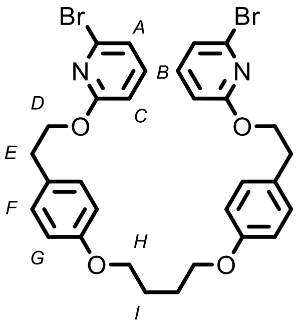

Macrocycle 2d Precursor (1d):

S5 (2.31 g, 7.00 mmol, 1 eq.), 2,6-dibromopyridine (4.97 g, 21.0 mmol, 3 eq.) and 60% NaH (0.840 g, 21.0 mmol, 3 eq.) were stirred at 80 °C in DMF (70 mL) for 18 h. H_2O (200 mL) was added to the cooled reaction. The resultant precipitate was isolated by filtration, washed with H_2O (100 mL), dissolved in CH_2Cl_2 (200 mL), dried ($MgSO_4$), filtered and the solvent removed *in vacuo*. After purification by column chromatography (1:1 petrol/CH_2Cl_2 with a 0 to 10% gradient of EtOAc), **1d** (3.50 g, 78%) was obtained as a crystalline white solid. M.p. 100-102 °C. ^{1}H NMR ($CDCl_3$, 400 MHz) δ: 7.39 (app. t, J = 7.8, 2H, H_B), 7.19 (d, J = 7.9, 4H, H_F), 7.03 (d, J = 7.5, 2H, H_A), 6.85 (d, J = 7.9, 4H, H_G), 6.66 (d, J = 8.1, 2H, H_C), 4.47 (t, J = 7.1, 4H, H_D), 4.02 (s, 4H, H_H), 3.01 (t, J = 7.1, 4H, H_E), 1.97 (s, 4H, H_I). ^{13}C NMR ($CDCl_3$, 100 MHz) δ: 163.5, 157.8, 140.5, 138.7, 130.4, 130.1, 120.3, 114.7, 109.7, 67.7, 67.6, 34.6, 26.2. HR-ESI-MS (CH_3CN) *m/z* = 641.0647 $[M+H]^+$ calc. 641.0645.

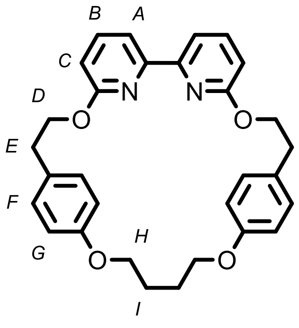

Macrocycle 2d:

Prepared according to general procedure with **1d** (1.28 g, 2.00 mmol) at 70 °C. Purification by column chromatography (petrol with a 0 to 50% gradient of Et_2O) afforded **2d** (0.623 g, 65%) as a colourless oil that solidified on standing. M.p. 107-109 °C. ^{1}H NMR ($CDCl_3$, 400 MHz) δ: 7.60 (app. t, J = 7.8, 2H, H_B), 7.39 (d, J = 7.4, 2H, H_A), 7.09 (d, J = 8.5, 4H, H_F), 6.70 (d, J = 8.2, 2H, H_C), 6.57 (d, J = 8.6, 4H, H_G), 4.71 (t, J = 7.3, 4H, H_D), 4.00 (br. m, 4H, H_H), 3.04 (t, J = 7.3, 4H, H_E), 1.89 (br. m, 4H, H_I). ^{13}C NMR ($CDCl_3$, 100 MHz) δ: 163.8, 157.3, 154.3, 139.1, 130.3, 129.9, 114.9, 114.8, 110.9, 67.3, 66.2, 35.4, 35.4. HR-ESI-MS (CH_3CN) *m/z* = 483.2290 $[M+H]^+$ calc. 483.2278.

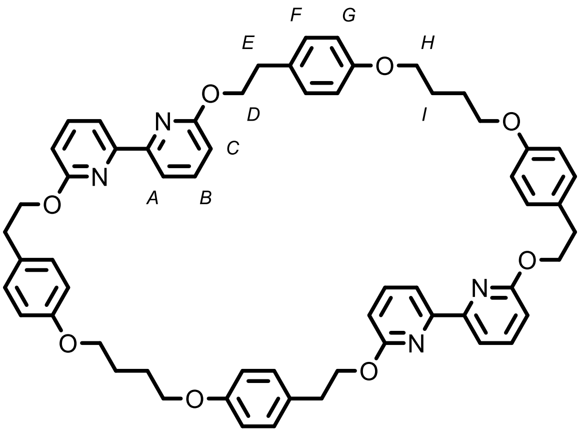

Macrocycle 2d Dimer:

^{1}H NMR ($CDCl_3$, 400 MHz) δ: 7.93 (d, J = 7.5, 4H, H_A), 7.63 (app. t, J = 7.8, 4H, H_B), 7.21 (d, J = 8.3, 8H, H_F), 6.84 (d, J = 8.5, 8H, H_G), 6.70 (d, J = 8.0, 4H, H_C), 4.59 (t, J = 7.0, 8H, H_D), 4.01 (br. m, 8H, H_H), 3.07 (t, J = 6.9, 8H, H_E), 1.95 (br. m, 8H, H_I).

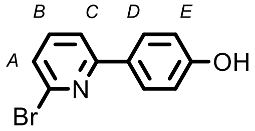

2-Bromo-6-(4-hydroxyphenyl)pyridine (S6):

A solution of 2-bromo-6-(4-methoxyphenyl)pyridine (11.89 g, 45.00 mmol, 1 eq.) in 48% aqueous HBr (45 mL) was stirred at reflux overnight. The cooled reaction mixture was diluted with H_2O (45 mL) and neutralized using a 10 M $NaOH_{(aq)}$ solution. The precipitate was recovered *via* filtration, washed with H_2O and dissolved in CH_2Cl_2. The organic layer was dried ($MgSO_4$), filtered and the solvent removed *in vacuo* affording **S5** (10 g, 88%) as a white solid. M.p. 132-134 °C. ^{1}H NMR ($CDCl_3$, 400 MHz) δ: 7.87 (d, J = 8.7, 2H, H_D), 7.59 (dd, J = 7.7, 1.2, 1H, H_C), 7.55 (app. t, J = 7.6, 1H, H_B), 7.35 (dd, J = 7.4, 1.2, 1H, H_A), 6.89 (d, J = 8.7, 2H, H_E), 5.44 (s, 1H, H_{OH}). ^{13}C NMR ($CDCl_3$, 100 MHz) δ: 158.5, 157.3, 142.1, 139.1, 130.5, 128.8, 125.7, 118.4, 115.9. HR-ESI-MS (MeCN): m/z = 249.9862 $[M+H]^+$ calc. 249.9865.

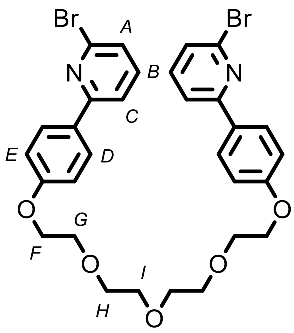

Macrocycle 2e Precursor (1e):

Tetraethylene glycol ditosylate (5.03 g, 10.0 mmol, 1 eq.), 2-bromo-6-(4-hydroxyphenyl)pyridine (6.25 g, 25.0 mmol, 2.5 eq.) and K_2CO_3 (6.91 g, 50.0 mmol, 5 eq.) were stirred in DMF (50 mL) at 80 °C for 16 h. H_2O (200 mL) was added to the cooled reaction mixture. The resultant precipitate was isolated by filtration, washed with H_2O (50 mL), dissolved in CH_2Cl_2 (250 mL), dried ($MgSO_4$), filtered and the solvent removed *in vacuo*. After purification by column chromatography (1:1 petrol/CH_2Cl_2 with a 0 to 25% gradient of EtOAc), **1e** (4.10 g, 62%) was isolated as a white solid. M.p. 98-100 °C. ^{1}H NMR ($CDCl_3$, 400 MHz) δ: 7.91 (d, J = 8.8, 4H, H_D), 7.57, (d, J = 7.7, 2H, H_A), 7.52 (app. t, J = 7.7, 2H, H_B), 7.32 (d, J = 7.6, 2H, H_C), 6.96 (d, J = 8.8, 4H, H_E), 4.16 (t, J = 4.8, 4H, H_F), 3.87 (t, J = 4.8, 4H, H_G), 3.75-3.69 (m, 8H, H_H, H_I). ^{13}C NMR ($CDCl_3$, 100 MHz) δ: 160.3, 158.3, 142.1, 139.0, 130.4, 128.4, 125.6, 118.2, 114.9, 71.0, 70.8, 69.8, 67.7. HR-ESI-MS (CH_3CN): m/z = 679.0414 $[M+Na]^+$ calc. 679.0414.

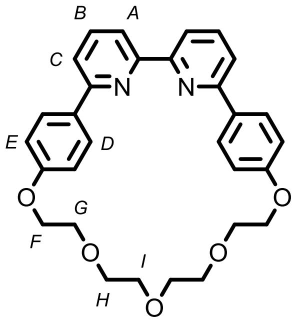

Macrocycle 2e:

Prepared according to general procedure with **1e** (1.32 g, 2.00 mmol). Purification by column chromatography (1:1 petrol/CH_2Cl_2 with a 0 to 20% gradient of MeCN) afforded **2e** (0.724 g, 73%) as a yellow solid. M.p. 135-137 °C. ^{1}H NMR ($CDCl_3$, 400 MHz) δ: 8.24 (d, J = 8.9, 4H, H_D), 7.84 (app. t, J = 7.7, 2H, H_B), 7.76-7.72 (m, 4H, H_A, H_C), 7.21 (d, J = 8.9, 4H, H_E), 4.38 (t, J = 4.9, 4H, H_F), 3.79 (t, J = 4.9, 4H, H_G), 3.67 (s, 8H, H_H, H_I). ^{13}C NMR ($CDCl_3$, 100 MHz) δ: 160.3, 157.3, 155.6, 137.6, 132.6, 128.5, 119.1, 118.8, 116.2, 71.2, 71.0, 70.7, 68.7. HR-ESI-MS (CH_3CN): m/z = 499.2237 $[M+H]^+$ calc. 499.2227.

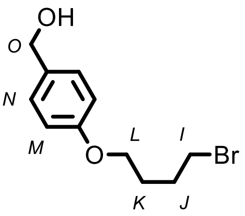

4-(4-bromobutoxy)benzyl alcohol (S7)

Prepared according to literature procedure.[1] ^{1}H NMR ($CDCl_3$, 400 MHz) δ: 7.28 (d, J = 8.7, 2H, H$_N$), 6.88 (d, J = 8.7, 2H, H$_M$), 4.61 (s, 2H, H$_O$), 3.99 (t, J = 6.0, 2H, H$_L$), 3.49 (t, J = 6.6, 2H, H$_I$), 2.11-2.04 (m, 2H, H$_J$), 1.98-1.91 (m, 2H, H$_K$), 1.64 (s, 1H, H$_{OH}$). ^{13}C NMR ($CDCl_3$, 100 MHz) δ: 158.6, 133.3, 128.8, 114.7, 67.0, 65.2, 33.6, 29.6, 28.0.

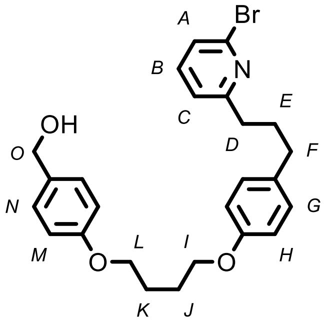

4-(4-(4-(3-(2-bromopyridin-6-yl)prop-1-yl)phenoxy)butoxy)benzyl alcohol (S8):

S7 (2.59 g, 10.0 mmol, 1 eq.), **S1** (3.51 g, 12.0 mmol, 1.2 eq.) and K_2CO_3 (4.15 g, 30.0 mmol, 3 eq.) were stirred in DMF (40 mL) at 80 °C for 18 h. To the cooled reaction was added H_2O (150 mL) and the resultant precipitate isolated by filtration, washed with H_2O (100 mL), dissolved in CH_2Cl_2 (150 mL), dried ($MgSO_4$), filtered and the solvent removed *in vacuo*. After purification by column chromatography (1:1 petrol/CH_2Cl_2 with 0% to 10% EtOAc gradient), **S8** (3.54 g, 75%) was obtained as a white solid. M.p. 82-84 ° C. ^{1}H NMR ($CDCl_3$, 400 MHz) δ: 7.43 (app. t, J = 7.7, 1H, H$_B$), 7.30-7.26 (m, 3H, H$_A$, H$_N$), 7.10-7.06 (m, 3H, H$_C$, H$_G$), 6.89 (d, J = 8.6, 2H, H$_M$), 6.82 (d, J = 8.5, 2H, H$_H$), 4.61 (s, 2H, H$_O$), 4.05-4.00 (m, 4H, H$_I$, H$_L$), 2.78 (t, J = 7.8, 2H, H$_D$), 2.61 (t, J = 7.6, 2H, H$_F$), 2.05-1.93 (m, 6H, H$_E$, H$_J$, H$_K$), 1.65 (s, 1H, H$_{OH}$). ^{13}C NMR ($CDCl_3$, 100 MHz) δ: 163.9, 158.8, 157.3, 141.7, 138.7, 134.1, 133.2, 129.5, 128.8, 125.4, 121.6, 114.7, 114.5, 67.7, 67.6, 65.2, 37.6, 34.7, 31.6, 26.2, 26.2. HR-ESI-MS (CH_3CN) m/z = 492.1138 $[M+Na]^+$ calc. 492.1145.

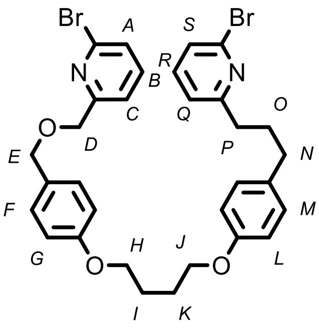

Macrocycle 2f Precursor (1f):

To a stirring solution of **S7** (2.35 g, 5.00 mmol, 1 eq.) in DMF (25 mL) at 0 ºC was added 60% NaH (0.300 g, 7.50 mmol, 1.5 eq.) as a solid. After 30 mins 2-bromo-6-(bromomethyl)pyridine (1.51 g, 6.00 mmol, 1.2 eq.) was added as a solid and the reaction mixture allowed to warm to RT and stirred for 19 h. MeOH (5 mL) followed by H_2O (100 mL) were added and the resultant precipitate isolated by filtration, washed with H_2O, dissolved in CH_2Cl_2 (150 mL), dried ($MgSO_4$), filtered and the solvent removed *in vacuo*. After purification by column chromatography (1:1 petrol/CH_2Cl_2 with a 0 to 10% gradient of EtOAc) the product was obtained as a white solid (2.94 g, 92%). M.p. 74-76 °C. ^{1}H NMR ($CDCl_3$, 400 MHz) δ: 7.55 (app. t, J = 7.7 Hz, 1H, H_B), 7.47-7.42 (m, 2H, H_A, H_R), 7.36 (d, J = 7.8, 1H. H_C), 7.30-7.27 (m, 3H, H_F, H_S), 7.10-7.06 (m, 3H, H_M, H_Q), 6.88 (d, J = 8.6, 2H, H_G), 6.82 (d, J = 8.6, 2H, H_L), 4.62 (s, 2H, H_D), 4.57 (s, 2H, H_E), 4.05-3.99 (m, 4H, H_H, H_J), 2.81-2.77 (m, 2H, H_P), 2.61 (t, J = 7.6, 2H, H_N), 2.05-1.96 (m, 6H, H_I, H_K, H_O). ^{13}C NMR ($CDCl_3$, 100 MHz) δ: 163.9, 160.6, 158.9, 157.3, 141.5, 141.3, 139.1, 138.8, 134.0, 129.8, 129.7, 129.5, 126.7, 125.5, 121.7, 120.1, 114.6, 114.5, 72.9, 72.1, 67.7, 67.6, 37.5, 34.7, 31.7, 26.2, 26.1. HR-ESI-MS (MeCN): m/z = 638.0878 $[M]^+$ calc. 638.0774.

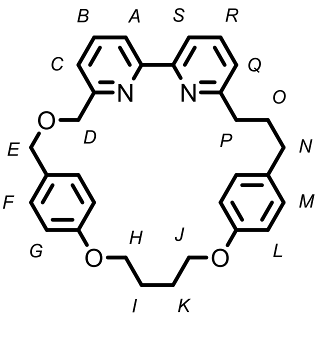

Macrocycle 2f:

Prepared according to general procedure with **1f** (1.28 g, 2.00 mmol). Purification by column chromatography (1:1 petrol/CH_2Cl_2 with a 0 to 10% gradient of EtOAc) afforded **2f** (0.730 g, 76%) as a colourless oil that solidified on standing. M.p. 77-80 °C. ^{1}H NMR ($CDCl_3$, 400 MHz) δ: 7.74-7.60 (m, 4H, H_A, H_B, H_R, H_S), 7.45 (d, J = 6.8 Hz, 1H, H_C), 7.23 (d, J = 8.6 Hz, 2H, H_F), 7.13 (dd, J = 6.6, 2.0 Hz, 1H, H_Q), 7.05 (d, J = 8.5 Hz, 2H, H_M), 6.80 (d, J = 8.6 Hz, 2H, H_G), 6.66 (d, J = 8.6 Hz, 2H,

H_L), 4.68 (s, 2H, H_E), 4.58 (s, 2H, H_D), 4.06 (t, J = 5.9 Hz, 2H, H_H), 4.00 (t, J = 6.0 Hz, 2H, H_J), 2.87-2.83 (m, 2H, H_P), 2.64 (t, J = 6.8 Hz, 1H, H_N), 2.12 (app. quint, J = 7.1 Hz, 2H, H_O), 1.96 (br. m, 4H, H_I, H_K). ^{13}C NMR ($CDCl_3$, 100 MHz) δ: 162.5, 159.5, 158.8, 157.1, 156.5, 156.3, 137.1, 136.8, 134.2, 130.5, 129.9, 129.7, 122.4, 120.9, 120.6, 119.4, 114.9, 114.6, 72.8, 71.9, 67.5, 67.4, 37.4, 34.5, 30.9, 25.6, 25.3. HR-ESI-MS (MeCN): m/z = 481.2492 $[M+H]^+$ calc. 481.2486.

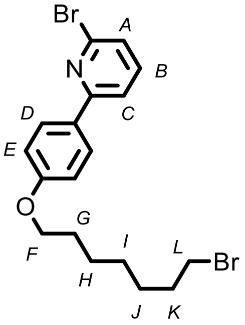

2-Bromo-6-(4-(7-bromohept-1-yl)phenyl)pyridine (S9):

To a solution of **S6** (2.00 g, 7.80 mmol, 1 eq.) in MeCN (40 mL) was added K_2CO_3 (4.44 g, 32.1 mmol, 4 eq.) as a solid. After stirring for 30 min, 1,7 dibromoheptane (5.19 g, 3.43 mL, 20.0 mmol, 2.5 eq.) was added *via* syringe and the reaction stirred at reflux for 18 h. The solvent was removed *in vacuo* and the resultant solid dissolved in CH_2Cl_2 (200 mL), washed with H_2O (50 mL) and brine (50 mL), dried ($MgSO_4$), filtered and the solvent removed *in vacuo*. After purification by column chromatography (hexane with 0% to 40% CH_2Cl_2 gradient), **S9** (2.50 g, 75%) was obtained as a white solid. M.p. 58-60 °C. ^{1}H NMR ($CDCl_3$, 400 MHz) δ: 7.93 (d, J = 8.0, 2H, H_D), 7.60 (d, J = 8.0, 1H, H_C), 7.53 (app. t, J = 8.0, 1H, H_B), 7.33 (d, J = 7.6, 1H, H_A), 6.96 (d, J = 8.8, 2H, H_E), 4.00 (t, J = 6.4, 2H, H_F), 3.42 (t, J = 6.8, 2H, H_L), 1.95-1.73 (m, 4H, H_G, H_K), 1.56-1.30 (m, 6H, H_H, H_I, H_J). ^{13}C NMR ($CDCl_3$, 100 MHz) δ: 160.6, 158.4, 142.1, 139.0, 130.1, 128.4, 125.5, 118.2, 114.8, 68.1, 34.0, 32.8, 29.2. 28.6, 28.2, 26.0. HR-ESI-MS (MeCN): m/z = 426.0074 $[M+H]^+$ calc. 426.0063.

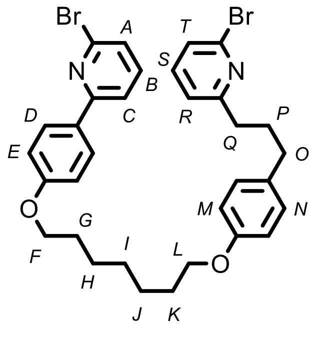

Macrocycle 2g Precursor (1g):

To a solution of **S1** (1.55 g, 5.38 mmol, 1.2 eq.) in MeCN (40 mL) was added K_2CO_3 (2.93 g, 21.2 mmol, 4 eq.) as a solid. After stirring for 30 min, **S9** (2.00 g, 4.69 mmol, 1 eq.) was added as a

solid and the reaction stirred at reflux for 18 h. The solvent was removed *in vacuo* and the resultant solid dissolved in CH_2Cl_2 (200 mL), washed with H_2O (50 mL) and brine (50 mL), dried ($MgSO_4$), filtered and the solvent removed *in vacuo*. After purification by column chromatography (7:3 petrol/CH_2Cl_2 with 0% to 5% EtOAc gradient), **1g** (2.91 g, 85%) was obtained as a white solid. M.p. 83-85 °C. ^{1}H NMR ($CDCl_3$, 400 MHz) δ: 7.93 (d, J = 8.8, 2H, H_D), 7.60 (d, J = 7.6, 1H, H_C), 7.53 (app. t, J = 7.8, 1H, H_B), 7.42 (app. t, J = 7.8, 1H, H_S), 7.33 (d, J = 7.6, 1H, H_A), 7.29 (d, J = 7.6, 2H, H_R), 7.10-7.05 (m, 3H, H_T, H_N), 6.96 (d, J = 9.6, 2H, H_E), 6.81 (d, J = 8.8, 2H, H_M), 4.01 (t, J = 4.8, 2H, H_F), 4.94 (t, J = 6.4, 2H, H_L), 2.78 (t, J = 7.6, 2H, H_Q), 2.61 (t, J = 7.6, 2H, H_O), 2.01 (q, J = 7.6, 2H, H_P), 1.89-1.77 (m, 4H, H_G, H_K), 1.59-1.41 (m, 6H, H_H, H_I, H_J). ^{13}C NMR ($CDCl_3$, 100 MHz) δ: 163.8, 160.6, 158.3, 157.4, 142.0, 141.6, 138.9, 138.6, 133.8, 130.0, 129.3, 128.3, 125.4, 125.3, 121.6, 118.1, 114.7, 114.4, 68.1, 67.9, 37.4, 34.6, 31.5, 29.3, 29.2, 29.2, 26.1, 26.0. HR-ESI-MS (MeCN): m/z = 637.1058 $[MH]^+$ calc. 637.1060.

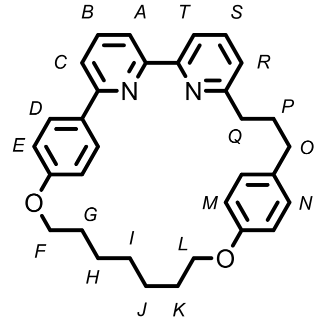

Macrocycle 2g:

Prepared according to general procedure with **1g** (1.28 g, 2.00 mmol) added as a solution in 1:1 THF/DMF (20 mL). Purification by column chromatography (1:1 petrol/CH_2Cl_2 with a 0 to 2% gradient of acetone) afforded **2g** (0.631 g, 66%) as an off-white solid. ^{1}H NMR ($CDCl_3$, 400 MHz) δ: 8.14 (d, J = 8.8, 2H, H_D), 7.80 (app. t, J = 8.0, 1H, H_B), 7.74-7.60 (m, 4H, H_C, H_S, H_A, H_R), 7.29 (d, J = 10.4, 2H, H_N), 7.21 (d, J = 7.3, 1H, H_T), 6.91 (d, J = 8.9, 2H, H_E), 6.80 (d, J = 8.5, 2H, H_M), 4.08 (t, J = 6.9, 2H, H_F), 3.95 (t, J = 6.5, 2H, H_L), 3.01 (t, J = 7.3, 2H, H_Q), 2.70 (t, J = 7.3, 2H, H_O), 2.34 (quint., J = 8.1, 2H, H_P), 1.91-1.78 (m, 4H, H_G, H_K), 1.64-1.45 (m, 6H, H_H, H_I, H_J). ^{13}C NMR ($CDCl_3$, 100 MHz) δ: 162.4, 160.0, 157.3, 157.2, 156.7, 156.5, 137.4, 136.8, 135.0, 132.0, 129.9, 128.6, 122.8, 119.6, 119.4, 118.6, 115.0, 114.5, 68.0, 67.5, 37.1, 34.3, 31.2, 28.1, 27.5, 26.9, 25.5, 25.4.

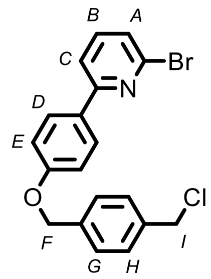

2-Bromo-6-(4-((4-(chloromethyl)benzyl)oxy)phenyl)pyridine (S10):

A solution of **S6** (5.00 g, 20.0 mmol, 1 eq.) α,α-dichloro-*p*-xylene (7.00 g, 40.0 mmol, 2 eq) and K_2CO_3 (11.0 g, 79.6 mmol, 4 eq.) in DMF (100 mL) was stirred at RT for 18 h. H_2O (500 mL) and $CHCl_3$ (500 mL) were added, the layers separated and the aqueous layer extracted with $CHCl_3$ (500 mL). The combined organics were dried ($MgSO_4$), filtered through celite and concentrated at 100 mbar in a 40 °C water bath to distil off the $CHCl_3$. MeOH (100 mL) was added and the resultant precipitate isolated by filtration, washed with MeOH and dried *in vacuo* to give the product (4.38 g, 56%) as a colourless solid. M.p. 159-161 °C. ^{1}H NMR ($CDCl_3$, 500 MHz) δ: 7.96 (d, J = 9.0 Hz, 2H, H_D), 7.61 (dd, J = 7.7, 0.9 Hz, 1H, H_C), 7.55 (app. t, J = 7.6 Hz, 1H, H_B), 7.45 (d, J = 8.5 Hz, 2H, $H_{G/H}$), 7.43 (d, J = 8.5 Hz, 1H, $H_{G/H}$), 7.35 (dd, J = 7.7, 0.9 Hz, 1H, H_A), 7.05 (d, J = 8.3 Hz, 2H, H_E), 5.14 (s, 2H, H_F), 4.61 (s, 2H, H_I). ^{13}C NMR ($CDCl_3$, 125 MHz) δ: 159.9, 158.1, 142.0, 138.9, 137.3, 137.0, 130.6, 128.9, 128.4, 127.8, 125.5, 118.1, 115.1, 69.6, 45.9. HR-ESI-MS (MeCN): m/z = 388.0094 $[M+H]^+$ calc. 388.0098.

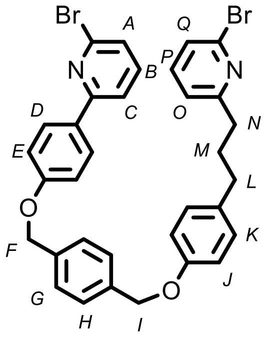

Macrocycle 2h Precursor (1h)

A solution of **S1** (3.88 g, 13.0 mmol, 1.2 eq.) **S10** (4.31 g, 11.1 mmol, 1 eq.) and K_2CO_3 (6.11 g, 44.2 mmol, 4 eq.) in DMF (55 mL) was stirred at RT for 18 h. H_2O (275 mL) and $CHCl_3$ (275 mL) were added, the layers separated and the aqueous layer extracted with $CHCl_3$ (275 mL). The combined organics were dried ($MgSO_4$) and concentrated at 100 mbar in a 40 °C water bath to distil off the $CHCl_3$. MeOH (55 mL) was added and the resultant precipitate isolated by filtration, washed with MeOH and dried *in vacuo*. After purification by column chromatography (1:1 petrol/CH_2Cl_2 to CH_2Cl_2), **1h** (4.51 g, 65%) was obtained as a colourless solid. M.p. 142-144 °C. ^{1}H NMR ($CDCl_3$, 400

MHz) δ: 7.95 (d, J = 8.9, 2H, H_D), 7.61 (dd, J = 7.8, 0.9, 1H, H_C), 7.54 (app. t, J = 7.7, 1H, H_B), 7.46 (app. br. s, 4H, H_G, H_H), 7.43 (t, J = 7.7, 1H, H_P), 7.34 (dd, J = 7.7, 0.9, 1H, H_A), 7.29 (dd, J = 7.8, 0.8, 1H, H_Q), 7.11-7.04 (m, 5H, H_K, H_O, H_E), 6.89 (d, J = 8.6, 2H, H_J), 5.13 (s, 2H, H_F), 5.06 (s, 2H, H_I), 2.78 (t, J = 7.8, 2H, H_N), 2.62 (t, J = 7.6, 2H, H_L), 2.01 (app. quint, J = 7.7, 2H, H_M). ^{13}C NMR ($CDCl_3$, 100 MHz, 298 K) δ: 163.9, 160.2, 158.3, 157.1, 142.2, 141.7, 139.0, 138.7, 137.3, 136.5, 134.5, 130.7, 129.5, 128.5, 127.8 (× 2), 125.7, 125.4, 121.7, 118.3, 115.2, 114.9, 70.0, 69.9, 37.6, 34.7, 31.6. HR-ESI-MS (MeCN): m/z = 643.0584 $[M+H]^+$ calc. 643.0590.

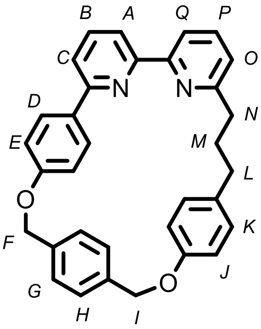

Macrocycle 2h – Williamson Synthesis:

Prepared according to general procedure with **1h** (1.28 g, 2.00 mmol) at 70 °C. Purification by column chromatography (petrol with a 0 to 50% gradient of Et_2O) afforded **2h** (0.623 g, 65%) as a colourless oil that solidified on standing. M.p. 177-179 °C. ^{1}H NMR ($CDCl_3$, 400 MHz) δ: 7.87 (d, J = 8.9, 2H, H_D), 7.78 (t, J = 7.8, 1H, H_B), 7.69-7.64 (m, 2H, H_A, H_P), 7.58 (d, J = 7.6, 1H, H_C), 7.53 (d, J = 7.5, 1H, H_Q), 7.35 (d, J = 8.1, 2H, H_H), 7.27 (d, J = 9.0, 2H, H_G), 7.18 (d, J = 7.5, 1H, H_O), 7.04 (d, J = 8.6, 2H, H_K), 6.74 (d, J = 8.9, 2H, H_E), 6.67 (d, J = 8.6, 2H, H_T), 5.24 (s, 2H, H_F), 5.19 (s, 2H, H_I), 2.95-2.91 (m, 2H, H_N), 2.64-2.60 (m, 2H, H_L), 2.19-2.11 (m, 2H, H_M). ^{13}C NMR ($CDCl_3$, 100 MHz) δ: 162.7, 159.1, 157.8, 157.0, 156.8, 155.6, 137.4, 137.3, 137.1, 136.8, 135.0, 132.8, 129.4, 128.5, 127.6, 126.7, 122.5, 119.9, 119.8, 119.1, 116.6, 115.9, 70.8, 69.7, 38.2, 35.0, 32.1. HR-ESI-MS (MeCN): m/z = 485.2222 $[M+H]^+$ calc. 485.2224.

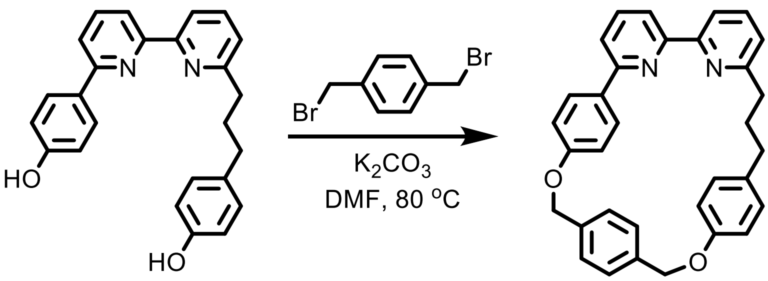

Williamson Synthesis of Macrocycle 2h:

A dry 2 L round bottomed flask was charged with bis-phenol[2] (2.01 g, 5.23 mmol), K_2CO_3 (7.23 g, 52.3 mmol), DMF (1.9 L) and 1,4-bis(bromomethyl)benzene (1.38 g, 5.23 mmol). The reaction

mixture was stirred at 80 °C for 72 h after which time the solvent was removed *in vacuo*. The residue was extracted with refluxing Et_2O (3 × 200 mL), the extracts combined and reduced *in vacuo*. The residue was purified by column chromatography (1:1 petrol/CH_2Cl_2 with a 0 to 10% gradient of MeCN) and the fractions containing the product further purified by GPC (Bio Rad Bio-Beads^{TM} S-X1 Support 200-400 mesh, eluting in DCM) to yield macrocycle **2h** (152 mg, 6%) as a white solid. All data were in accordance with those reported above.

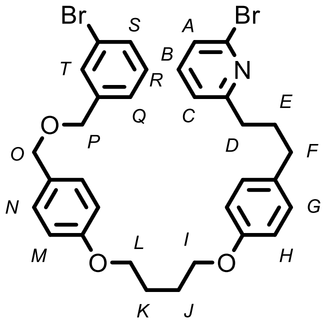

Macrocycle 2i Dibromo-precursor (1i^{Br}):

To a solution of **S7** (2.35 g, 5.00 mmol, 1 eq.) in DMF (25 mL) at 0 °C was added 60% NaH (0.300 g, 7.50 mmol, 1.5 eq.) as a solid. After stirring for 30 min, 3-bromobenzyl bromide (1.87 g, 7.50 mmol, 1.5 eq.) was added as a solid and the reaction stirred at RT for 22 h. H_2O (400 mL) was added and the resultant precipitate isolated by filtration, washed with H_2O (100 mL), dissolved in CH_2Cl_2 (400 mL), dried ($MgSO_4$), filtered and the solvent removed *in vacuo*. After purification by column chromatography (1:1 petrol/CH_2Cl_2 with 0% to 5% EtOAc gradient), **1i^{Br}** (3.08 g, 96%) was obtained as a colourless oil that solidified on standing. M.p. 42-44 °C. ^{1}H NMR ($CDCl_3$, 400 MHz) δ: 7.51 (s, 1H, H_T), 7.46-7.40 (m, 2H, H_B, H_Q), 7.31-7.26 (m, 4H, H_A, H_N, H_S), 7.21 (app. t, J = 7.7, 1H, H_R), 7.10-7.07 (m, 3H, H_C, H_G), 6.89 (d, J = 8.6, 2H, H_M), 6.82 (d, J = 8.6, 2H, H_H), 4.49 (s, 2H, $H_{O/P}$), 4.48 (s, 2H, $H_{O/P}$), 4.05-4.00 (m, 4H, H_I, H_L), 2.80 (t, J = 7.8, 2H, H_D), 2.62 (t, J = 7.6, 2H, H_F), 2.05-1.96 (m, 6h, H_E, H_J, H_K). ^{13}C NMR ($CDCl_3$, 100 MHz) δ: 163.9, 158.9, 157.3, 141.5, 140.9, 138.8, 134.0, 130.8, 130.7, 130.1, 130.0, 129.6, 129.5, 126.3, 125.5, 122.7, 121.7, 114.6, 114.5, 72.2, 71.0, 67.7, 67.6, 37.5, 34.7, 31.7, 26.2, 26.2. HR-ESI-MS (CH_3CN) m/z = 638.0909 $[M+H]^+$ calc. 638.0900.

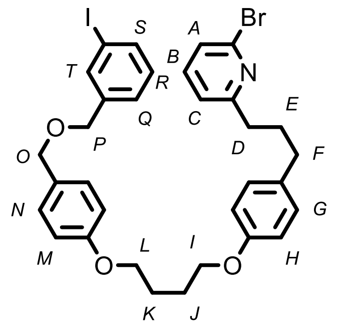

Macrocycle 2i Iodobromo-precursor ($1i^I$):

To a solution of **S7** (1.41 g, 3.00 mmol, 1 eq.) in DMF (15 mL) at 0 °C was added 60% NaH (0.180 g, 4.50 mmol, 1.5 eq.) as a solid. After stirring for 30 min, 3-iodobenzyl bromide (1.34 g, 4.50 mmol, 1.5 eq.) was added as a solid and the reaction stirred at RT for 21 h. H_2O (100 mL) was added and the resultant precipitate isolated by filtration, washed with H_2O (100 mL), dissolved in CH_2Cl_2 (200 mL), dried ($MgSO_4$), filtered and the solvent removed *in vacuo*. After purification by column chromatography (1:1 petrol/CH_2Cl_2 with 0% to 5% EtOAc gradient), $\mathbf{1i^I}$ (1.58 g, 77%) was obtained as a white solid. M.p. 41-43 °C. ^{1}H NMR ($CDCl_3$, 400 MHz) δ: 7.72 (s, 1H, H_T), 7.62 (d, J = 7.9, 1H, H_S), 7.43 (app. t, J = 7.7, 1H, H_B), 7.33-7.26 (m, 4H, H_A, H_N, H_Q), 7.11-7.06 (m, 4H, H_C, H_G, H_R), 6.90 (d, J = 8.6, 2H, H_M), 6.83 (d, J = 8.6, 2H, H_H), 4.49 (s, 2H, H_P), 4.46 (s, 2H, H_O), 4.06-4.01 (m, 4H, H_I, H_L), 2.79 (t, J = 7.8, 2H, H_D), 2.62 (t, J = 7.6, 2H, H_F), 2.06-1.97 (m, 6H, H_E, H_J, H_K). ^{13}C NMR ($CDCl_3$, 100 MHz) δ: 163.9, 158.8, 157.3, 141.6, 140.9, 138.7, 136.7, 136.7, 134.0, 130.2, 130.0, 129.6, 129.5, 126.9, 125.4, 121.6, 114.6, 114.5, 94.5, 72.2, 70.9, 67.6, 67.6, 37.5, 34.6, 31.6, 26.2, 26.1. HR-ESI-MS (CH_3CN) m/z = 686.0770 $[M+H]^+$ calc. 686.0761.

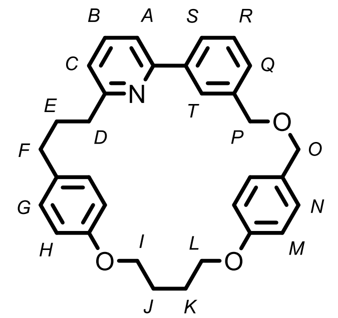

Macrocycle 2i:

Prepared according to general procedure with $\mathbf{1i^I}$ (1.37 g, 2.00 mmol). Purification by column chromatography (1:1 petrol/CH_2Cl_2 with a 0 to 5% gradient of EtOAc) afforded **2i** (0.231 g, 24%) as a colourless oil. ^{1}H NMR ($CDCl_3$, 400 MHz) δ: 8.21 (s, 1H, H_T), 7.70 (d, J = 6.2, 1H, H_S), 7.65 (app. t, J = 7.7, 1H, H_B), 7.53 (d, J = 7.8, 1H, H_A), 7.44-7.43 (m, 2H, H_Q, H_R), 7.28-7.26 (m, 2H, H_N), 7.14-7.10 (m, 3H, H_C, H_G), 6.75-6.70 (m, 4H, H_H, H_M), 4.56 (s, 2H, H_O), 4.51 (s, 2H, H_P), 4.10-4.08 (m,

4H, H_I, H_L), 2.87 (t, J = 7.6, 2H, H_D), 2.65 (t, J = 7.5, 2H, H_F), 2.15 (app. quint, J = 7.6, 2H, H_E), 1.98 (br, 4H, H_J, H_K). ^{13}C NMR ($CDCl_3$, 100 MHz) δ: 162.0, 158.6, 157.1, 156.7, 140.0, 139.3, 137.0, 134.5, 130.1, 129.7, 129.6, 128.7, 128.2, 126.8, 125.7, 121.3, 117.9, 114.8, 114.8, 71.8, 71.1, 67.5, 67.5, 37.7, 34.8, 31.6, 25.7, 25.6. HR-ESI-MS (CH_3CN) m/z = 480.2533 $[M+H]^+$ calc. 480.2533.

General Rotaxination Procedure

To macrocycle **2** (1 eq.), azide half-thread, alkyne half-thread and $[Cu(CH_3CN)_4]PF_6$ (0.96 eq.) in 1:1 EtOH/CH_2Cl_2 (0.01 M based on macrocycle) was added $^iPr_2NEt$ (2 eq.) and the reaction stirred at 80 °C for 16 h. After removal of the solvent *in vacuo*, the crude reaction mixture was dissolved in CH_2Cl_2 (5 mL) and treated with F_3CCOOH (5 drops). After stirring at RT for several hours the reaction mixture was washed with EDTA-NH_3 solution (50 mL), further extracted with CH_2Cl_2 (2 × 10 mL), dried ($MgSO_4$), filtered and the solvent removed *in vacuo*. Purification by column chromatography yielded the products as off-white solids/foams.

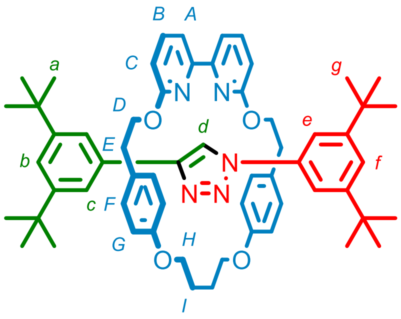

Rotaxane S11

Prepared according to general procedure with **2d** (24.1 mg, 0.0500 mmol, 1 eq.), alkyne **3** (107 mg, 0.500 mmol, 10 eq.), 3,5-di-*tert*-butylphenyl azide (**S15**) (116 mg, 0.500 mmol, 10 eq.), $[Cu(CH_3CN)_4]PF_6$ (17.9 mg, 0.048 mmol, 0.96 eq) and $^iPr_2NEt$ (17 µL, 0.10 mmol, 2 eq.). Purification by column chromatography (petrol with a 0 to 50% gradient of Et_2O) afforded the product (35.3 mg, 76%) as a white foam. ^{1}H NMR ($CDCl_3$, 400 MHz) δ: 9.83 (s, 1H, H_d), 7.68-7.64 (m, 4H, H_B, H_e), 7.61 (d, J = 1.8, 2H, H_c), 7.29 (d, J = 7.0, 2H, H_A), 7.25-7.23 (m, 2H, H_b, H_f), 6.71 (d, J = 7.8, 2H, H_c), 6.54 (d, J = 8.5, 4H, $H_{F/G}$), 6.34 (d, J = 8.5, 4H, $H_{F/G}$), 4.58-4.52 (m, 2H, H_H), 4.36-4.30 (m, 2H, $H_{H'}$), 3.84 (t, J = 7.7, 4H, H_D), 2.60-2.48 (m, 4H, H_E), 2.32-2.14 (m, 4H, H_I), 1.24 (s, 18H, H_f), 1.22 (s, 18H, H_a). ^{13}C NMR ($CDCl_3$, 100 MHz) δ: 164.8, 158.0, 156.7, 151.2, 150.3, 145.7, 139.2, 137.3, 131.5, 128.4, 128.3, 120.4, 120.4, 120.3, 117.2, 116.4, 114.4, 109.9, 68.4, 67.8, 35.2, 34.9, 34.7, 31.6, 31.5, 25.4. LR-ESI-MS (CH_3CN) m/z = 928.61 $[M+H]^+$ calc. 928.57

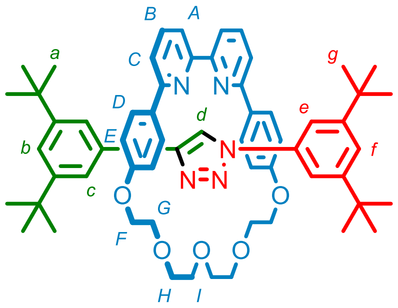

Rotaxane S12

Prepared according to general procedure with **2e** (12.5 mg, 0.0250 mmol, 1 eq.), alkyne **3** (8.1 mg, 0.038 mmol, 1.5 eq.), 3,5-di-*tert*-butylphenyl azide (**S15**) (8.8 mg, 0.038 mmol, 1.5 eq.), $[Cu(CH_3CN)_4]PF_6$ (8.9 mg, 0.024 mmol, 0.96 eq) and $^iPr_2NEt$ (9 µL, 0.01 mmol, 2 eq.). Purification by column chromatography (1:1 petrol/CH_2Cl_2 with a 0 to 10% gradient of MeCN) afforded the product (21.3 mg, 90%) as a white foam. ^{1}H NMR ($CDCl_3$, 400 MHz) δ: 10.30 (s, 1H, H_d), 7.87 (app. t, J = 7.7, 2H, H_B), 7.81 (dd, J = 0.7, 7.7, 2H, H_A), 7.52 (m, 4H, H_c, H_e), 7.50 (dd, J = 0.8, 7.7, 2H, H_C), 7.25 (t, J = 1.6, 1H, $H_{b/f}$), 7.22 (t, J = 1.8, 1H, $H_{b/f}$), 7.13 (d, J = 8.7, 4H, H_D), 6.24 (d, J = 8.7, 4H, H_E), 4.06-3.87 (m, 12H, H_F, H_H, H_I), 3.62-3.59 (m, 4H, H_G), 1.15 (s, 18H, $H_{a/g}$), 1.14 (m, 18H, $H_{a/g}$). ^{13}C NMR ($CDCl_3$, 100 MHz) δ: 160.2, 158.5, 157.5, 151.6, 150.4, 147.0, 137.5, 137.1, 132.5, 131.1, 129.0, 123.9, 121.3, 121.0, 120.7, 120.1, 119.7, 114.4, 114.2, 69.7, 69.6, 68.4, 66.3, 35.0, 34.8, 31.5, 31.4. HR-ESI-MS (CH_3CN) m/z = 944.5703 $[M+H]^+$ calc. 944.5684.

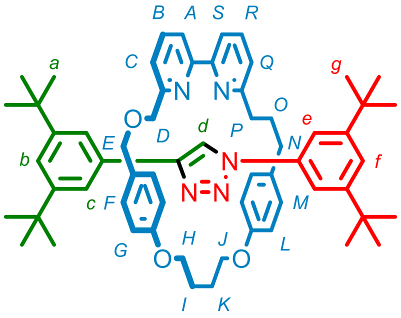

Rotaxane S13

To a solution of **2f** (24.0 mg, 0.0500 mmol, 1 eq.), 3,5-di-*tert*-butylphenyl azide (**S15**) (17.4 mg, 0.0750 mmol, 1.5 eq.), alkyne **3** (16.1 mg, 0.0750 mmol, 1.5 eq.) and $[Cu(CH_3CN)_4]PF_6$ (17.9 mg, 0.0480 mmol, 0.96 eq.) in 1:1 EtOH/CH_2Cl_2 (5 mL) was added $^iPr_2EtN$ (17 µL, 0.10 mmol, 2 eq.) and the reaction stirred at 80 °C for 16 h. After removal of the solvent *in vacuo* the crude mixture was dissolved in CH_2Cl_2 (10 mL) and washed with EDTA-NH_3 solution (50 mL). After further extraction with CH_2Cl_2 (2 × 10 mL) the combined organic extracts were dried ($MgSO_4$), filtered and the solvent removed *in vacuo*. After purification by column chromatography (petrol with a 0 to 50% gradient of Et_2O), **S13** was obtained as a white foam (42 mg, 91%). ^{1}H NMR ($CDCl_3$, 400 MHz) δ: 10.49 (s, 1H,

H_d), 7.71 (app. t, J = 7.8, 2H, H_B, H_R), 7.68 (d, J = 1.7, 2H, H_e), 7.64-7.62 (m, 2H, H_A, H_S), 7.61 (d, J = 1.8, 2H, H_c), 7.38 (dd, J = 0.7, 7.6, 2H, H_C), 7.21 (t, J = 1.7, 1H, H_f), 7.18-7.15 (m, 2H, H_b, H_Q), 6.71-6.65 (m, 4H, H_F, H_G), 6.38-6.31 (m, 4H, H_L, H_M), 4.86 (app. q, J = 7.1, 1H, H_H), 4.67 (app. q, J = 7.3, 1H, H_K), 4.48-4.43 (m, 1H, H_H), 4.38-4.31 (m, 2H, H_E), 4.28-4.23 (m, 1H, H_K), 3.64-3.56 (m, 2H, H_D), 2.50-2.18 (m, 8H, H_I, H_J, H_N, H_P), 1.64-1.56 (m, 2H, H_O), 1.18 (s, 18H, H_g), 1.15 (s, 18H, H_a). ^{13}C NMR ($CDCl_3$, 100 MHz) δ: 163.7, 160.3, 159.7, 157.2, 156.5, 156.0, 151.2, 150.1, 146.4, 137.5, 137.1, 137.1, 131.8, 131.7, 128.7, 128.3, 127.9, 122.6, 121.7, 120.4, 120.2, 120.2, 120.0, 119.7, 119.4, 115.7, 114.5, 114.3, 73.0, 69.4, 67.0, 66.1, 37.5, 35.5, 35.2, 34.9, 32.6, 31.5, 31.5, 25.2, 25.0. HR-ESI-MS (MeCN): m/z = 926.5962 $[M+H]^+$ calc. 926.5943.

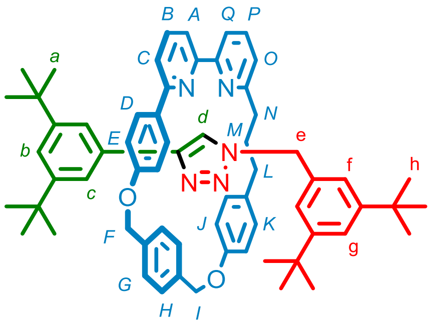

Rotaxane S14

Prepared according to general procedure with **2h** (24.2 mg, 0.0500 mmol), alkyne **3** (107 mg, 0.500 mmol, 10 eq.), 3,5-di-*tert*-butylbenzyl azide (**S16**) (123 mg, 0.500 mmol, 10 eq.), $[Cu(CH_3CN)_4]PF_6$ (17.9 mg, 0.048 mmol, 0.96 eq) and ^{i}Pr$_2$NEt (17 µL, 0.10 mmol, 2 eq.). Purification by column chromatography (petrol with a 0 to 100% gradient of Et_2O) afforded the product (23.2 mg, 50%) as a white foam. ^{1}H NMR ($CDCl_3$, 400 MHz) δ: 10.70 (s, 1H, H_d), 7.80 (d, J = 8.0, 2H, $H_{G/H}$), 7.75 (app. t, J = 7.7, 1H, H_P), 7.68-7.63 (m, 3H, $H_{G/H}$, H_Q), 7.59 (app. t, J = 7.7, 1H, H_B), 7.52 (d, J = 7.3, 1H, H_A), 7.28 (t, J = 1.7, 1H, H_g), 7.20-7.18 (4H, H_C, H_O, H_f), 7.12 (d, J = 1.8, 2H, H_c), 7.00 (t, J = 1.8, 1H, H_b), 6.51 (d, J = 8.6, 2H, H_D), 6.48 (d, J = 8.6, 2H, H_K), 6.27 (d, J = 8.7, 2H, H_E), 6.11 (d, J = 8.5, 2H, H_J), 5.32 (d, J = 13.9, 1H, $H_{F/I}$), 5.24-5.14 (m, 3H, $H_{F/I}$), 3.97 (d, J = 14.3, 1H, H_e), 3.86 (d, J = 14.3, 1H, H_e), 2.77-2.60 (m, 2H, H_N), 2.36-2.28 (m, 1H, H_M), 2.16-2.09 (m, 1H, H_M), 1.89-1.66 (m, 2H, H_L), 1.14 (s, 18H, $H_{a/h}$), 1.12 (s, 18H, $H_{a/h}$). ^{13}C NMR ($CDCl_3$, 100 MHz) δ: 162.9, 159.4, 158.1, 157.7, 156.9, 156.1, 150.6, 149.0, 145.5, 138.8, 137.8, 137.3, 136.4, 134.9, 132.3, 131.9, 131.1, 128.4, 128.1, 126.0, 124.3, 123.0, 122.4, 120.3, 120.3, 119.9, 119.9, 119.6, 114.8, 114.1, 69.1, 68.9, 52.8, 38.4, 35.2, 34.7, 34.6, 31.7, 31.4, 31.3. LR-ESI-MS (MeCN): m/z = 944.61 $[M+H]^+$ calc. 944.58.

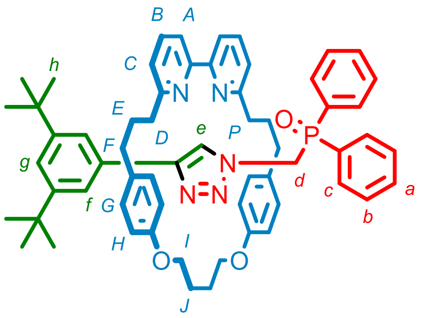

Rotaxane 5

To a solution of **2a** (0.957 g, 2.00 mmol, 1 eq.), **3** (0.644 g, 3.00 mmol, 1.5 eq.), **4** (0.772 g, 3.00 mmol, 1.5 eq.) and $[Cu(CH_3CN)_4]PF_6$ (0.717 g, 1.92 mmol, 0.96 eq.) in 1:1 EtOH:CH_2Cl_2 (20 mL) was added ^{i}Pr_2EtN (0.52 mL, 4.0 mmol, 2 eq.) and the reaction stirred at 80 °C for 18 h. After evaporation of the solvent, EtOH (10 mL) was added to the crude mixture. The suspension was sonicated and left to stand for 30 min. After this time, the ethanolic phase was decanted and the remaining solid washed with EtOH (2 × 10 mL). The solid was then dissolved in CH_2Cl_2 (500 mL), washed with EDTA-NH_3 solution (500 mL) and brine (500 mL), dried ($MgSO_4$) and the solvent removed *in vacuo* to give **5** (1.495 g). The ethanolic phase was then diluted with CH_2Cl_2 (500 mL), washed with EDTA-NH_3 solution (500 mL) and brine (500 mL), dried ($MgSO_4$) and the solvent removed *in vacuo*. After purification by column chromatography (1:1 petrol/CH_2Cl_2 with a 0 to 40% gradient of MeCN) additional **5** (0.303 g) of was obtained. These two were combined to give **5** as a white foam (1.798 g, 95%). ^{1}H NMR ($CDCl_3$, 500 MHz) δ: 9.72 (s, 1H, H_e), 7.77-7.71 (m, 4H, H_c), 7.68 (app t, J = 7.8, 2H, H_B), 7.56-7.49 (m, 4H, $H_{A/a}$), 7.47 (d, J = 2.1, 2H, H_f), 7.45-7.40 (m, 4H, H_b), 7.18 (t, J = 1.5, 1H, H_g), 7.14 (d, J = 7.8, 2H, H_C), 6.33 (d, 4H, J = 8.4, H_H), 6.26 (d, 4H, J = 8.4, H_G), 4.59 (d, 2H, J = 4.4, H_d), 4.27-4.17 (m, 4H, H_I), 2.48-2.29 (m, 8H, $H_{D/F}$), 2.27-2.18 (m, 2H, 2 of H_J), 2.06-1.95 (m, 2H, 2 of H_J), 1.70-1.56 (m, 4H, H_E), 1.20 (s, 18H, H_h). ^{13}C NMR ($CDCl_3$,125 MHz) δ: 163.5, 157.7, 157.4, 149.7, 145.9, 136.7, 132.1 (d, J_{CP}= 2.9), 132.0, 131.7 (d, J_{CP}= 9.5), 131.3, 131.1, 128.6 (d, J_{CP}= 12.1), 128.5, 125.4, 121.8, 120.7, 120.4, 119.9, 115,3, 67.0, 48.5 (d, J_{CP}= 74.0), 37.0, 35.1, 34.9, 31.6, 31.1, 25.1. ^{31}P NMR ($CDCl_3$, 202 MHz) δ: 24.4.

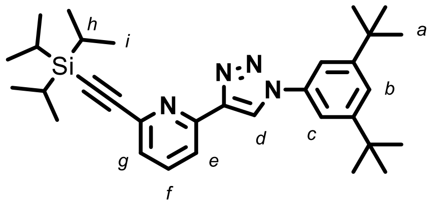

2-(Triisopropylsilylethynyl)-6-(1-(3,5-di-*tert*-butylphen-1-yl)-1,2,3-triazol-4-yl)pyridine (S17):

2,6-Dibromopyridine (7.0 g, 30 mmol), [Pd(PPh_3)$_2$$Cl_2$] (0.52 g, 0.74 mmol) and CuI (0.14 g, 0.74 mmol) were dissolved in Et_2NH (70 mL) and the mixture cooled to 0 ºC. (Triisopropylsilyl)acetylene (6.6 mL, 45 mmol) was added and the mixture was allowed to warm to RT overnight. Brine (30 mL) and CH_2Cl_2 (50 mL) were added and the phases separated. The aqueous phase was extracted with CH_2Cl_2 (3 × 50 mL). The combined organic phases were dried over $MgSO_4$ and the solvent removed *in vacuo*. The mixture was purified by filtration through a short pad of silica gel (100% petrol) to afford crude 2-(triisopropylsilylethynyl)-6-bromopyridine (9.2 g, 92%).

The crude 2-(triisopropylsilylethynyl)-6-bromopyridine, [Pd(PPh_3)$_2$$Cl_2$] (0.29 g, 0.41 mmol) and CuI (0.13 g, 0.68 mmol) were dissolved in Et_3N (90 mL) and trimethylsilylacetylene (4.3 mL, 30 mmol) was added. The mixture was stirred at 35 ºC for 17 h and cooled to RT. H_2O (100 mL) was added followed by CH_2Cl_2 (100 mL) and the phases were separated. The aqueous phase was extracted with CH_2Cl_2 (3 × 100 mL). The combined organic phases were washed with brine (100 mL) and dried over $MgSO_4$. The solvent was removed *in vacuo* and purified by filtration through a short pad of silica gel (100% petrol) to afford crude 2-(triisopropylsilylethynyl)-6-(trimethylsilylethynyl)pyridine (6.8 g, 71%).

The crude 2-(triisopropylsilylethynyl)-6-(trimethylsilylethynyl)pyridine was dissolved in CH_2Cl_2 (40 mL) and MeOH (135 mL) and KOH (2.2 g, 38 mmol) was added. The mixture was stirred at RT overnight and the solvent removed *in vacuo*. H_2O (100 mL) and CH_2Cl_2 (100 mL) were added and the phases separated. The aqueous phase was extracted with CH_2Cl_2 (3 × 100 mL). The combined organic phases were dried over $MgSO_4$ and the solvent removed *in vacuo* to afford the crude 2-(triisopropylsilylethynyl)-6-ethynylpyridine (4.9 g, 90%).

Crude 2-(triisopropylsilylethynyl)-6-ethynylpyridine (3.0 g, 11 mmol) and 1-azido-3,5-di-tert-butylbenzene (2.4 g, 11 mmol) were dissolved in DMF (53 mL). Sodium ascorbate (2.6 g, 11 mmol) and $CuSO_4 \cdot 5H_2O$ (2.6 g, 11 mmol) were added and the reaction mixture stirred at RT for 4 h. EDTA-NH_3 solution (50 mL) was added followed by Et_2O (100 mL). The phases were separated and the organic phase washed with H_2O (3 × 50 mL) and brine (50 mL) and dried over $MgSO_4$. The crude mixture was purified by flash column chromatography on silica gel (0% to 5% EtOAc in petroleum ether) to give the product as an off-white solid (2.6 g, 49%). ^1H NMR (400 MHz, $CDCl_3$) δ 8.66 (s,

1H, H$_d$), 8.22 (d, J = 7.9, 1H, H$_e$), 7.76 (app. t, J = 7.9, 1H, H$_f$), 7.62 (d, J = 1.6, 2H, H$_c$), 7.52 (d, J = 1.6, 1H, H$_b$), 7.45 (d, J = 7.9, 1H, H$_g$), 1.39 (d, J = 0.9, 18H, H$_i$), 1.18 (s, 18H, H$_a$), 1.17 (s, 3H, H$_h$). ^{13}C NMR (100 MHz, $CDCl_3$) δ 152.8, 150.5, 148.2, 143.1, 136.9, 136.7, 127.4, 123.1, 121.1, 119.8, 115.4, 106.0, 92.0, 35.2, 31.4, 18.7, 11.3. HR-ESI-MS (CH_3CN): m/z = 515.3571 $[M+H]^+$ calc. 515.3565.

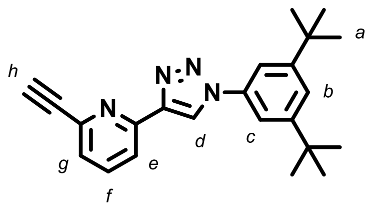

2-(Ethynyl)-6-(1-(3,5-di-*tert*-butylphen-1-yl)-1,2,3-triazol-4-yl)pyridine (6):

S17 (2.6 g, 5.2 mmol) was dissolved in THF (dry, 330 mL) and MeOH (dry, 0.5 mL). nBu$_4$NH$_4$F (1 M in THF, 6.2 mL, 6.2 mmol) was added and the reaction mixture was stirred at RT for 1.5 h. $CaCO_3$ (1.2 g, 12 mmol, 1.9 eq.) was added followed by DOWEX 50WX-8-400 (3.7 g) and MeOH (9 mL) and the mixture was stirred at RT for 1.5 h. The reaction mixture was filtered through a short pad of celite and washed with MeOH. The solvent was removed *in vacuo* to give the product as an off-white solid (1.8 g, 98%). The product can be used crude or purified by flash column chromatography on silica gel (0% to 20% EtOAc in petrol). M.p. 109-112 °C. ^{1}H NMR (400 MHz, $CDCl_3$) δ 8.70 (s, 1H, H$_d$), 8.28 (d, J = 7.8, 1H, H$_e$), 7.80 (app. t, J = 7.8, 1H, H$_f$), 7.52 (br. s, 1H, H$_b$), 7.46 (d, J = 7.8, 1H, H$_g$), 7.63 (d, J = 1.6, 2H, H$_c$), 3.22 (s, 1H, H$_h$), 1.39 (s, 18H, H$_a$). ^{13}C NMR (100 MHz, $CDCl_3$) δ 152.9, 150.8, 148.0, 142.0, 137.2, 136.6, 126.7, 123.1, 120.8, 120.4, 115.1, 82.8, 77.3, 31.4, 17.7. HR-ESI-MS (CH_3CN): m/z = 359.2240 $[M+H]^+$ calc. 359.2230.

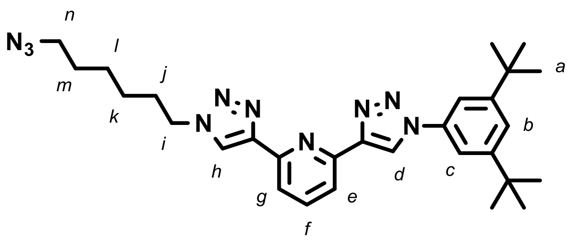

2-(1-(6-azidohex-1-yl)-1,2,3-triazol-4-yl)-6-(1-(3,5-di-*tert*-butylphen-1-yl)-1,2,3-triazol-4-yl)pyridine (7):

NaN_3 (2.9 g, 45 mmol) and 1,6-dibromohexane (0.86 mL, 5.6 mmol) were dissolved in DMF (dry, 12 mL) and the mixture was heated to 80 ºC for 16 h. **6** (0.4 g, 1 mmol), sodium ascorbate (0.22 g, 1.1 mmol) and $CuSO_4 \cdot 5H_2O$ (0.14 g, 0.55 mmol) were added and the mixture was stirred at 60 ºC for 16 h. To the cooled reaction mixture was added Et_2O (20 mL) and H_2O (20 mL). The phases were separated and the organic phase washed with H_2O (3 × 20 mL) and brine (20 mL). The organic phases were dried ($MgSO_4$), filtered and the solvent was removed *in vaccuo*. The crude was purified by flash column chromatography on silica gel (0% to 30% EtOAc in petroleum ether) to give the product as a pale yellow solid (0.18 g, 30%). M.p. 142-143 °C. ^{1}H NMR (400 MHz, $CDCl_3$) δ 8.59 (s, 1H, H_d), 8.22 (s, 1H, H_h), 8.20 (dd, J = 7.8, 1.1 Hz, 1H, H_g), 8.13 (dd, J = 7.8, 1.1 Hz, 1H, H_e), 7.91 (app. t, J = 7.8 Hz, 1H, H_f), 7.61 (d, J = 1.7 Hz, 2H, H_c), 7.53 (t, J = 1.7 Hz, 1H, H_b), 4.44 (t, J = 7.1 Hz, 2H, H_i), 3.26 (t, J = 6.7 Hz, 2H, H_n), 2.06 – 1.92 (m, 2H, H_j), 1.67 – 1.53 (m, 2H, H_m), 1.40 (br, 22H, H_a, H_k, H_l). ^{13}C NMR (100 MHz, $CDCl_3$) δ 152.88, 150.2, 150.0, 148.6, 148.4, 137.9, 136.7, 123.1, 121.9, 120.4, 119.7, 119.6, 115.5, 51.2, 50.3, 35.2, 31.4, 30.2, 28.6, 26.2, 26.1. HR-ESI-MS (CH_3CN): *m/z* = 527.3367 $[M+H]^+$ calc. 527.3354.

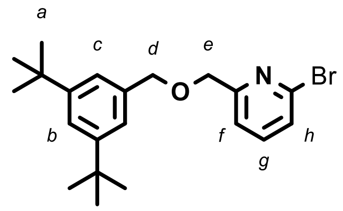

2-Bromo-6-(3,5-di-*tert*-butylbenzyloxymethyl)pyridine (S18):

To a suspension of 60% NaH (0.280 g, 7.00 mmol, 1.4 eq.) in DMF (17 mL) at 0 °C was added dropwise a solution of 2-(6-bromopyridine)methanol (1.18 g, 6.25 mmol, 1.25 eq.) in DMF (8 mL). After stirring for 30 minutes, 3,5-di-*tert*-butylbenzyl bromide (1.42 g, 5.00 mmol, 1 eq.) was added as a solid in one portion. The reaction was stirred for 5 h, allowing to warm up to RT. H_2O (80 mL) was added to the reaction, resulting in a yellow precipitate that was dissolved in CH_2Cl_2 (100 mL), dried ($MgSO_4$), filtered and the solvent removed *in vacuo*. The material was purified by loading onto a plug of silica, eluting with petrol to remove impurities, followed by CH_2Cl_2 to elute the product. After

removal of solvent *in vacuo* the product was obtained as white solid (1.46 g, 75%). M.p. 71-73 °C. ^{1}H NMR ($CDCl_3$, 400 MHz) δ: 7.56 (app. t, J = 7.6, 1H, H$_g$), 7.50 (d, J = 7.3, 1H, H$_h$), 7.38-7.37 (m, 2H, H$_b$, H$_f$), 7.23 (d, J = 1.7, 2H, H$_c$), 4.68 (s, 2H, H$_e$), 4.64 (s, 2H, H$_d$), 1.34 (s, 18H, H$_a$). ^{13}C NMR ($CDCl_3$, 100 MHz) δ: 160.8, 151.1, 141.4, 139.0, 136.9, 126.6, 122.3, 122.1, 120.1, 74.0, 72.5, 35.0, 31.6. HR-ESI-MS (CH_3CN): m/z = 412.1237 $[M+Na]^+$ calc. 412.1246.

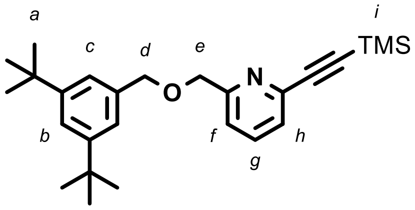

2-(Trimethylsilylethynyl)-6-(3,5-di-*tert*-butylbenzyloxymethyl)pyridine (S19):

To a stirring solution of **S18** (2.63 g, 6.74 mmol, 1 eq.), $Pd(PPh_3)_2Cl_2$ (0.118 g, 0.168 mmol, 2.5 mol%) and CuI (0.032 g, 0.17 mmol, 2.5 mol%) in Et_3N (25 mL) at 0 °C was added ethynyltrimethylsilane (1.14 mL, 8.08 mmol, 1.2 eq.) dropwise. The reaction was then allowed to stir at RT for 22 h. CH_2Cl_2 (25 mL) was added and the reaction washed with EDTA-NH_3 solution (50 mL). After further extraction with CH_2Cl_2 (50 mL), the combined organic phases were washed with brine (50 mL), dried ($MgSO_4$), filtered and the solvent removed *in vacuo*. After filtration through a plug of silica (CH_2Cl_2) the product was obtained as a brown oil (2.69 g, 98%). ^{1}H NMR ($CDCl_3$, 400 MHz) δ: 7.59 (app. t, J = 7.8, 1H, H$_g$), 7.43 (d, J = 7.8, 1H, H$_h$), 7.31-7.29 (m, 2H, H$_b$, H$_f$), 7.15 (d, J = 1.8, 2H, H$_c$), 4.64 (s, 2H, H$_e$), 4.56 (s, 2H, H$_d$), 1.27 (s, 18H, H$_a$), 0.21 (s, 9H, H$_i$). ^{13}C NMR ($CDCl_3$, 100 MHz) δ: 159.7, 151.0, 142.3, 137.0, 136.8, 126.2, 122.3, 122.1, 120.8, 103.8, 94.8, 73.9, 73.1, 35.0, 31.6, -0.1. HR-ESI-MS (CH_3CN): m/z = 408.2727 $[M+H]^+$ calc. 408.2717.

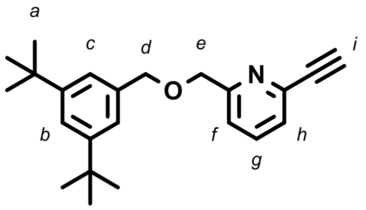

2-Ethynyl-6-(3,5-di-*tert*-butylbenzyloxymethyl)pyridine (8):

S19 (2.67 g, 6.55 mmol, 1 eq.) and K_2CO_3 (4.53 g, 32.7 mmol, 5 eq.) were stirred in MeOH (65 mL) at RT for 90 min. H_2O (200 mL) was added and the reaction extracted with CH_2Cl_2 (4 × 50 mL). The combined organic phases were washed with brine (50 mL), dried ($MgSO_4$), filtered and the solvent removed *in vacuo*. After filtration through a plug of silica (CH_2Cl_2) the product was obtained as a yellow oil (1.73 g, 79%). ^{1}H NMR ($CDCl_3$, 400 MHz) δ: 7.68 (app. t, J = 7.8, 1H, H$_g$), 7.53 (d, J = 7.8, 1H, H$_h$), 7.40-7.37 (m, 2H, H$_b$, H$_f$), 7.22 (d, J = 1.6, 2H, H$_c$), 4.71 (s, 2H, H$_e$), 4.64 (s, 2H, H$_d$),

3.14 (s, 1H, H$_i$), 1.33 (s, 18H, H$_a$). ^{13}C NMR ($CDCl_3$, 100 MHz) δ: 159.8, 151.0, 141.5, 137.0, 136.9, 126.1, 122.3, 122.1, 121.3, 82.9, 74.0, 73.0, 34.9, 31.6. HR-ESI-MS (CH_3CN): *m/z* = 336.2322 $[M+H]^+$ calc. 336.2322.

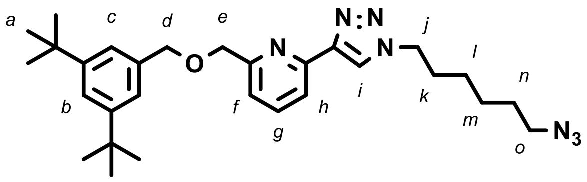

2-(1-(6-azidohex-1-yl)-1,2,3-triazol-4-yl)-6-(3,5-di-*tert*-butylbenzyloxymethyl)pyridine (S20):

NaN_3 (1.56 g, 23.8 mmol) and 1,6-dibromohexane (0.72 mL, 4.8 mmol) were dissolved in DMF (12 mL) and the mixture heated at 60 ºC for 16 h. Alkyne **8** (0.4 g, 1 mmol), sodium ascorbate (0.24 g, 1.2 mmol) and $CuSO_4 \cdot 5H_2O$ (0.15 g, 0.60 mmol) were added and the mixture stirred at 60 ºC for 16 h. To the cool reaction mixture was added Et_2O (20 mL) and EDTA-NH_3 solution (20 mL). The phases were separated and the organic phase washed with H_2O (3 × 20 mL) and brine (20 mL). The organic phases were dried ($MgSO_4$) and the solvent removed *in vacuo*. The material was purified by flash column chromatography on silica gel (0% to 40% EtOAc in petrol) to give the product as a pale yellow oil (0.17 g, 29%). ^{1}H NMR ($CDCl_3$, 400 MHz) δ: 8.12 (s, 1H, H$_i$), 8.06 (d, J = 7.7, 1H, H$_h$), 7.79 (app. t, J = 7.8, 1H, H$_g$), 7.45 (d, J = 7.7, 1H, H$_f$), 7.37 (t, J = 1.8, 1H, H$_b$), 7.24 (d, J = 1.8, 2H, H$_c$), 4.71 (s, 2H, H$_e$), 4.66 (s, 2H, H$_d$), 4.42 (t, J = 7.1, 2H, H$_j$), 3.26 (t, J = 6.8, 2H, H$_o$), 2.01-1.94 (m, 2H, H$_k$), 1.63-1.56 (m, 2H, H$_n$), 1.59-1.33 (m, 4H, H$_l$, H$_m$), 1.33 (s, 18H, H$_a$). ^{13}C NMR ($CDCl_3$, 100 MHz) δ: 158.8, 151.0, 149.8, 148.7, 137.6, 137.2, 122.2, 122.0, 122.0, 120.7, 118.9, 73.8, 73.3, 51.4, 50.4, 35.0, 31.6, 30.3, 28.8, 26.3, 26.2. HR-ESI-MS (CH_3CN): *m/z* = 504.3446 $[M+H]^+$ calc. 504.3445.

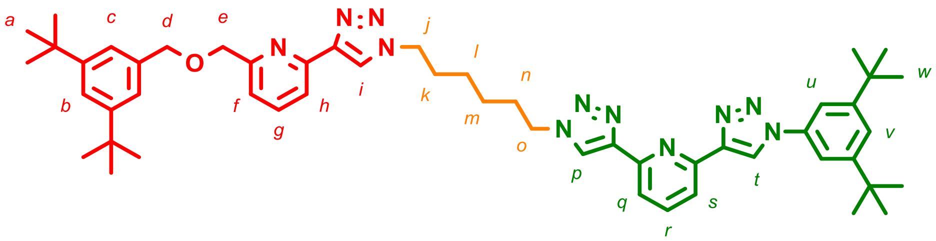

Shuttle Thread (S19):

Azide **S20** (0.126 g, 0.250 mmol, 1 eq.), **4** (0.090 g, 0.25 mmol, 1 eq.), sodium ascorbate (0.047 g, 0.25 mmol, 1 eq.) and $CuSO_4 \cdot 5H_2O$ (0.031 g, 0.13 mmol, 0.5 eq.) were stirred in DMF (1.25 mL) for 20 h. EDTA-NH_3 solution (8 mL) was added. The aqueous phase was extracted with EtOAc (2 × 20 mL), washed with H_2O (4 × 10mL), brine (10 mL), dried ($MgSO_4$), filtered and the solvent removed *in vacuo*. After purification by column chromatography (1:1 petrol/CH_2Cl_2 with a 0 to 25% gradient

of MeCN) the product was obtained as a light yellow foam (0.084 g, 39%). ^{1}H NMR ($CDCl_3$, 400 MHz) δ: 8.60 (s, 1H, H_t), 8.21-8.19 (m, 2H, H_p, $H_{s/q}$), 8.13-8.11 (m 2H, H_i, $H_{s/q}$), 8.04 (d, *J* = 7.6, 1H, H_h), 7.90 (app. t, *J* = 7.8, 1H, H_r), 7.77 (app. t, *J* = 7.8, 1H, H_g), 7.61 (d, *J* = 1.7, 2H, H_u), 7.53 (t, *J* = 1.7, 1H, H_v), 7.43 (d, *J* = 7.6, 1H, H_f), 7.36 (t, *J* = 1.9, 1H, H_b), 7.23 (d, *J* = 1.8, 2H, H_c), 4.70 (s, 2H, H_e), 4.64 (s, 2H, H_d), 4.45-4.39 (m, 4H, H_j, H_o), 2.00-1.95 (m, 4H, H_k, H_n), 1.43-1.39 (m, 4H, H_l, H_m), 1.39 (s, 18H, H_w), 1.32 (s, 18H, H_a). ^{13}C NMR ($CDCl_3$, 100 MHz) δ: 159.8, 153.0, 151.0, 150.3, 150.1, 149.7, 148.8, 148.7, 148.5, 138.0, 137.5, 137.2, 136.9, 123.3, 122.2, 122.1, 122.1, 122.0, 120.7, 120.6, 119.8, 119.7, 118.9, 115.7, 73.8, 73.3, 50.4, 50.3, 35.4, 35.0, 31.6, 31.5, 30.3, 30.1, 26.0, 26.0. HR-ESI-MS (CH_3CN): *m/z* = 884.5408 $[M+Na]^+$ calc. 884.5422.

3. Synthesis and shuttling experiments of rotaxanes 9a and 9e

General procedure for complexation experiments: Zn(II) and Cu(I) complexes of the shuttles (**9a** and **9e**) were prepared by titration of $Zn(OTf)_2$ and $[Cu(CH_3CN)_4](PF_6)$, respectively, into an approximately 10 mM solution of each shuttle.

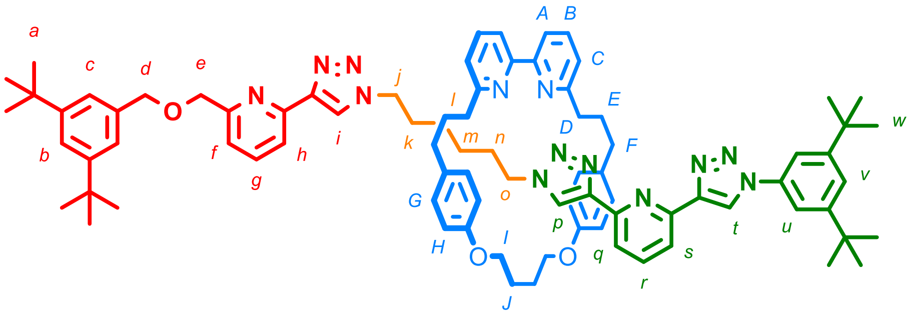

Shuttle 9a: Azide **7** (50 mg, 0.095 mmol, 1.0 eq), alkyne **8** (32 mg, 0.095 mmol, 1.0 eq.), $[Cu(MeCN)_4](PF_6)$ (34 mg, 0.090 mmol, 0.95 eq.) and **2a** (45 mg, 0.095 mmol, 1.0 eq.) were dissolved in CH_2Cl_2 (5 mL) and $^{i}Pr_2EtN$ (0.17 mL, 0.95 mmol) was added. The reaction mixture was stirred at 80 ºC for 15 h. The cooled reaction solution was washed with EDTA-NH_3 solution (20 mL) and the aqueous phase extracted with CH_2Cl_2 (3 × 10 mL). The combined organic phases were washed with brine (20 mL) and dried over $MgSO_4$. The crude was purified by flash column chromatography on silica gel (0% to 60% EtOAc in petrol) to give the product as a pale yellow foam (93 mg, 75%) in approximately 95% purity (^{1}H NMR). For the shuttling experiments, an analytical sample (29 mg) was prepared through a second round of chromatography. ^{1}H NMR ($CDCl_3$, 500 MHz) δ: 8.52 (s, 1H, H$_{t}$), 8.40 (s, 1H, H$_{p}$), 8.15 (d, J = 7.7, 1H, H$_{q/s}$), 8.06 (s, 1H, H$_{i}$), 7.96-7.91 (m, 2H, H$_{h}$, H$_{q/s}$), 7.82 (app. t, J = 7.8, 1H, H$_{r}$), 7.75 (app. t, J = 7.8, 1H, H$_{g}$), 7.54 (d, J = 1.4, 2H, H$_{u}$), 7.51-7.46 (m, 3H, H$_{v}$, H$_{B}$), 7.43 (d, J = 7.7, 1H, H$_{f}$), 7.37 (s, 1H, H$_{b}$), 7.31 (d, J = 7.7, 2H, H$_{A}$), 7.22 (d, J = 1.4, 2H, H$_{c}$), 7.04 (d, J = 7.7, 2H, H$_{C}$), 6.81 (d, J = 8.4, 4H, H$_{G}$), 6.68 (d, J = 8.5, 4H, H$_{H}$), 4.68 (s, 2H, H$_{e}$), 4.63 (s, 2H, H$_{d}$), 4.12-4.01 (m, 4H, H$_{I}$), 3.81 (t, J = 7.6, 2H, H$_{j}$), 3.59 (t, J = 7.8, 2H, H$_{o}$), 2.60-2.48 (m, 8H, H$_{F}$, H$_{D}$), 1.94 (br, 4H, H$_{J}$), 1.83-1.73 (m, 4H, H$_{E}$), 1.35 (s, 18H, H$_{w}$), 1.32 (s, 18H, H$_{a}$), 1.21-1.14 (m, 2H, H$_{k}$), 1.01-0.93 (m, 2H, H$_{n}$), 0.64-0.63 (m, 4H, H$_{l}$, H$_{m}$). ^{13}C NMR ($CDCl_3$, 125 MHz) δ: 162.6, 158.8, 157.9, 157.5, 152.9, 151.0, 150.6, 150.0, 149.9, 148.9, 148.0, 147.7, 137.6, 137.4, 137.2, 136.9, 136.9, 133.6, 129.6, 123.3, 123.1, 122.6, 122.2, 122.0, 121.6, 120.8, 120.3, 120.2, 119.4, 119.2, 118.7, 115.6, 114.9, 73.8, 73.4, 66.5, 50.0, 49.9, 37.0, 35.3, 35.0, 34.7, 32.0, 31.6, 31.5, 29.4, 29.1, 25.7, 25.6, 24.8. LR-ESI-MS (CH_3CN) m/z = 1340.82 $[M+H]^+$calc. 1340.82.

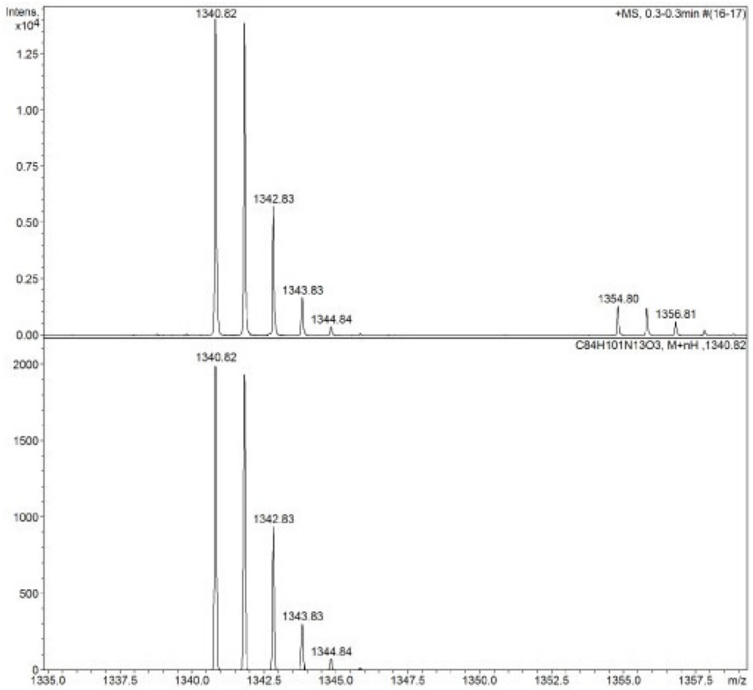

Figure S1 Experimental (top) and calculated (bottom) LR-ESI-MS for 9a.

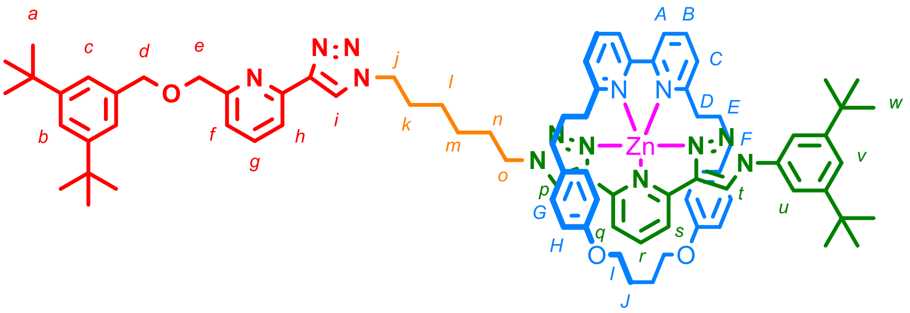

[Zn(9a)](OTf)$_2$:

^{1}H NMR ($CDCl_3$, 500 MHz) δ: 8.92 (s, 1H, H_p), 8.74 (d, J = 8.1, 2H, H_A), 8.73-8.69 (m, 1H, H_r), 8.45-8.39 (m, 1H, H_h), 8.36 (app. t, J = 8.0, 2H, H_B), 8.19-8.10 (m, 1H, H_s), 8.02-7.96 (m, 1H, H_q), 7.82-7.76 (m, 1H, H_g), 7.67 (d, J = 7.9, 2H, H_C), 7.63 (t, J = 1.6, 1H, H_v), 7.51 (d, J = 1.7, 2H, H_u), 7.46 (br, 1H, H_f), 7.36 (br, 1H, H_b), 7.22 (br, 2H, H_c), 6.59-6.55 (m, $H_{G/H}$), 6.43 (d, J = 8.6, 4H, $H_{G/H}$), 4.72 (br, 2H, H_e), 4.64 (br, 2H, H_d), 4.55-4.51 (m, 2H, H_I), 4.42-4.39 (m, 2H, H_o), 4.37-4.34 (m, 2H, H_j), 4.31-4.26 (m, 2H, H_I), 2.53-2.23 (m, 16H, H_D, H_E, H_F, H_J), 1.89 (br, 4H, H_k, H_n), 1.40 (s, 18H, H_w), 1.31 (br, 22H, H_a, H_l, H_m). ^{13}C NMR ($CDCl_3$, 125 MHz) δ: 161.4, 157.5, 154.0, 152.0, 151.0, 149.4, 144.6, 143.8, 143.7, 141.3, 141.2, 135.6, 132.6, 129.7, 129.3, 129.1, 126.8, 125.2, 124.2, 124.1, 123.5, 122.5, 122.2, 122.0, 115.8, 115.5, 115.1, 114.9, 114.8, 73.9, 73.3, 66.7, 52.0, 50.2, 37.8, 35.6, 34.7, 33.7, 31.6, 31.6, 31.5, 31.4, 29.5, 25.5, 25.5, 25.3.

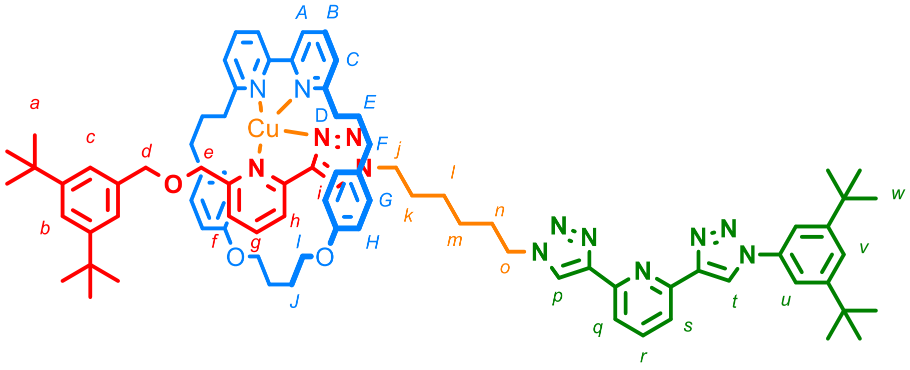

[Cu(9a)](PF$_6$):

^{1}H NMR (CDCl$_3$, 500 MHz) δ: 8.68 (s, 1H, H$_t$), 8.39 (s, 1H, H$_p$), 8.17 (d, J = 7.8, 2H, H$_{s/r}$), 8.07-8.00 (m, 4H, H$_{s/r}$, H$_i$, H$_A$), 7.91-7.81 (m, 4H, H$_B$, H$_g$, H$_r$), 7.64 (d, J = 1.5, 2H, H$_u$), 7.53 (t, J = 1.4, 1H, H$_v$), 7.40 (d, J = 7.7, 2H, H$_C$), 7.34-7.32 (m, 2H, H$_b$, H$_f$), 6.95 (d, J = 1.8, 2H, H$_c$), 6.78 (d, J = 7.9, 1H, H$_h$), 6.50 (d, J = 8.3, 4H, H$_G$), 6.33 (d, J = 8.4, 4H, H$_H$), 4.59 (t, J = 7.4, 2H, H$_j$), 4.49 (t, J = 6.8, 2H, H$_o$), 4.39-4.34 (m, 2H, H$_I$), 4.24-4.20 (m, 2H, H$_I$), 4.03 (s, 2H H$_d$), 3.95 (s, 2H, H$_e$), 2.47-2.36 (m, 6H, H$_D$, H$_F$), 2.27-2.14 (m, 6H, H$_F$, H$_J$), 2.09-2.05 (m, 4H, H$_k$, H$_n$), 1.53 (br. m, 8H, H$_E$, H$_l$, H$_m$), 1.40 (s, 18H, H$_w$), 1.28 (s, 18H, H$_a$). ^{13}C NMR (CDCl$_3$, 125 MHz) δ: 161.9, 157.6, 155.1, 153.0, 151.8, 151.2, 150.4, 150.2, 148.8, 148.3, 144.9, 144.0, 138.2, 137.7, 137.6, 136.9, 136.2, 132.4, 128.8, 125.6, 124.2, 123.2, 123.0, 122.3, 122.2, 122.0, 121.5, 120.8, 119.5, 119.2, 115.7, 114.9, 114.7, 73.7, 72.5, 66.1, 51.1, 50.3, 39.3, 35.4, 35.2, 34.9, 31.6, 31.5, 30.9, 30.1, 30.1, 25.6, 25.5, 24.6.

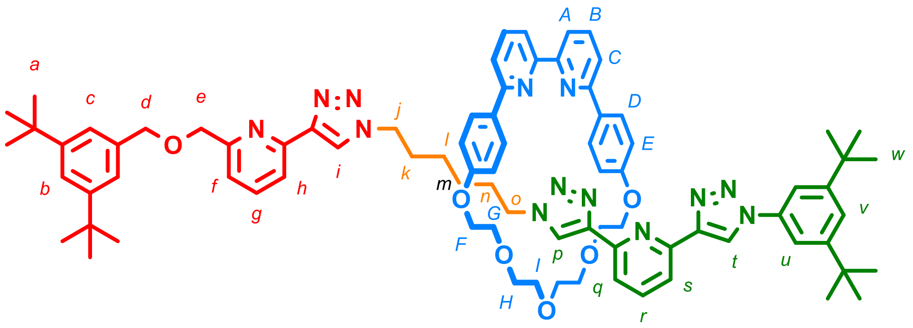

Shuttle 9e:

Azide **7** (50 mg, 0.095 mmol, 1.0 eq.), alkyne **8** (32 mg, 0.095 mmol, 1.0 eq.), $[Cu(MeCN)_4]PF_6$ (34 mg, 0.090 mmol, 0.95 eq.) and **2e** (47 mg, 0.095 mmol) were dissolved in CH_2Cl_2 (5 mL) and $^iPr_2EtN$ (0.17 mL, 0.95 mmol, 10 eq.) was added. The reaction mixture was stirred at 80 ºC for 18 h. To the cooled reaction was added MeOH (10mL) followed by a solution of KCN (38 mg, 0.58 mmol, 6.1 eq.) in MeOH (5 mL) and the mixture was stirred vigorously for 1 h. The solvent was removed *in vacuo*, the residue dissolved in CH_2Cl_2 (10 mL) and washed with brine (10 mL). The phases were separated and the aqueous phase was extracted with CH_2Cl_2 (3 × 10 mL). The combined organic phases were dried over $MgSO_4$. The crude was purified by flash column chromatography on silica gel (0% to 50% MeCN in 1:1 petrol/CH_2Cl_2 then 0% to 10% MeOH in 1:1 petrol/CH_2Cl_2) to give the product as a pale yellow foam (57 mg, 55 %). ^{1}H NMR ($CDCl_3$, 500 MHz) δ: 9.53 (s, 1H, H_t), 8.34 (s, 1H, H_p), 8.05 (s, 1H, H_i), 8.02-7.98 (m, 2H, H_h, H_s), 7.91 (dd, 1H, H_q), 7.76 (app. t, 1H, H_g), 7.73-7.69 (m, 3H, H_r, H_B), 7.60 (d, 2H, H_A), 7.45-7.42 (m, 3H, H_f, H_C), 7.39 (d, 2H, H_u), 7.37 (t, 1H, H_b), 7.35-7.33 (m, 5H, H_v, H_D), 7.23 (d, 2H, H_c), 6.30 (d, 4H, H_E), 4.69 (s, 2H, H_e), 4.64 (s, 2H, H_d), 4.19 (t, J = 7.1, 2H, H_j), 3.99 (t, J = 6.0, 4H, H_F), 3.91-3.82 (m, 10H, H_o, H_H, H_I), 3.60-3.55 (m, 4H, H_G), 1.73-1.65 (m, 2H, H_k), 1.51-1.45 (m, 2H, H_l), 1.32 (s, 18H, H_a), 1.19 (s, 18H, H_w), 1.14-1.08 (m, 4H, H_m, H_n). ^{13}C NMR ($CDCl_3$, 125 MHz) δ: 159.8, 159.0, 158.9, 157.4, 152.2, 151.0, 150.1, 149.8, 149.6, 148.6, 148.5, 148.1, 137.5, 137.4, 137.2, 137.1, 136.9, 132.6, 129.2, 124.4, 124.1, 122.3, 122.2, 122.2, 122.0, 120.5, 120.1, 119.3, 119.2, 118.8, 118.3, 115.0, 114.4, 73.8, 73.4, 70.2, 70.1, 68.6, 66.7, 50.2, 49.8, 35.1, 35.0, 31.6, 31.4, 29.9, 29.5, 26.0, 25.9. LR-ESI-MS (CH_3CN) m/z = 1360.78 $[M+H]^+$ calc. 1360.78.

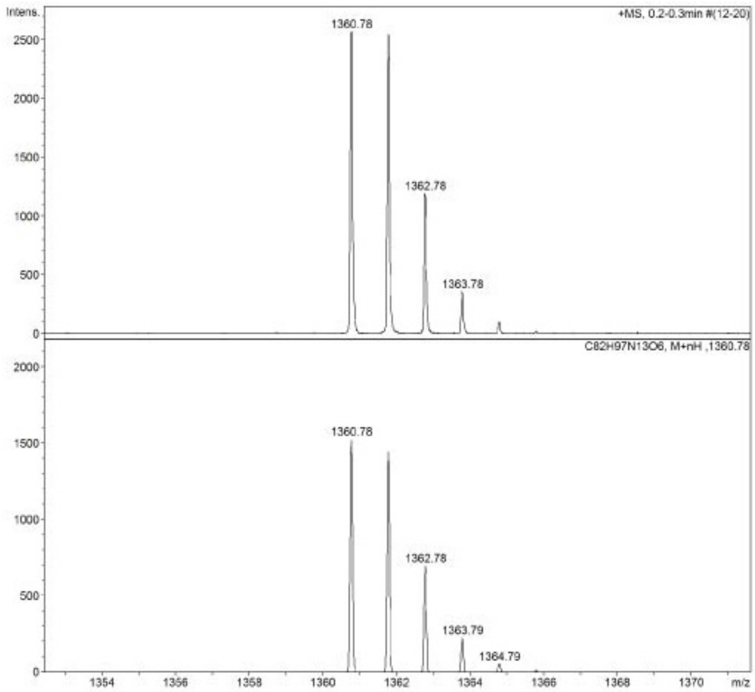

Figure S2 Experimental (top) and calculated (bottom) LR-ESI-MS for 9e.

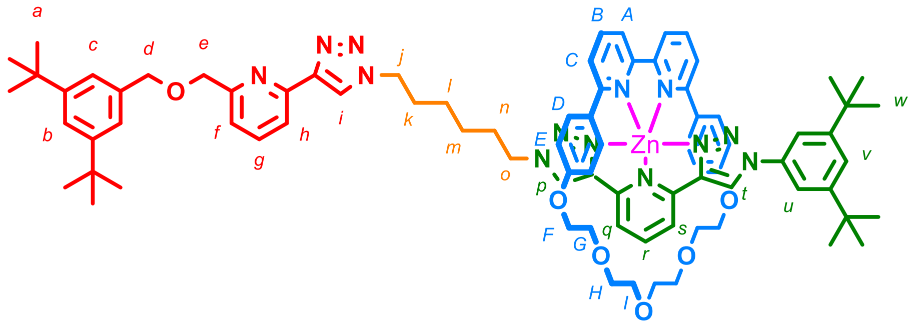

[Zn(9e)](OTf)$_2$:

^{1}H NMR (CDCl$_3$, 500 MHz) δ: 9.42 (s, 1H, H$_t$), 9.01 (s, 1H, H$_p$), 8.76 (dd, J = 8.2, 0.9, 2H, H$_A$), 8.70 (app. t, J = 7.9, 1H, H$_r$), 8.47 (app. t, J = 7.9, 2H, H$_B$), 8.28 (dd, J = 7.9, 0.8, 1H, H$_s$), 8.17 (s, 1H, H$_i$), 8.09 (d, J = 7.9, 1H, H$_q$), 8.00 (d, J = 7.9, 1H, H$_h$), 7.81 (dd, J = 7.8, 0.8, 2H, H$_C$), 7.77 (app. t, J = 7.8, 1H, H$_g$), 7.60 (t, J = 1.7, 1H, H$_v$), 7.57 (d, J = 1.7, 2H, H$_u$), 7.45 (d, J = 7.7, 1H, H$_f$), 7.36 (t, J = 1.9, 1H, H$_b$), 7.23 (d, J = 1.9, 2H, H$_c$), 6.73 (d, J = 8.6, 4H, H$_D$), 6.29 (d, J = 8.6, 4H, H$_E$), 4.71 (s, 2H, H$_e$), 4.65 (s, 2H, H$_d$), 4.47 (t, J = 7.4, 2H, H$_o$), 4.42 (t, J = 6.9, 2H, H$_j$), 3.90-3.74 (m, 8H, H$_H$, H$_I$), 3.71-3.65 (m, 8H, H$_F$, H$_G$), 1.99-1.91 (m, 4H, H$_k$, H$_n$), 1.42-1.39 (m, 4H, H$_m$, H$_l$), 1.39 (s, 18H, H$_w$), 1.32 (s, 18H, H$_a$). ^{13}C NMR (CDCl$_3$, 125 MHz) δ: 160.4, 160.0, 158.9, 153.9, 151.0, 149.8, 149.7, 148.5, 148.0, 143.7, 142.7, 142.5, 142.4, 141.6, 137.6, 137.2, 135.7, 131.6, 128.6, 125.9, 125.5, 125.0, 123.3, 123.2, 122.8, 122.5, 122.4, 122.2, 122.0, 120.6, 118.9, 115.4, 114.8, 73.9, 73.3, 71.3, 71.0, 69.4, 68.5, 52.0, 50.2, 35.5, 35.0, 31.6, 31.4, 30.0, 29.8, 25.6, 25.6.

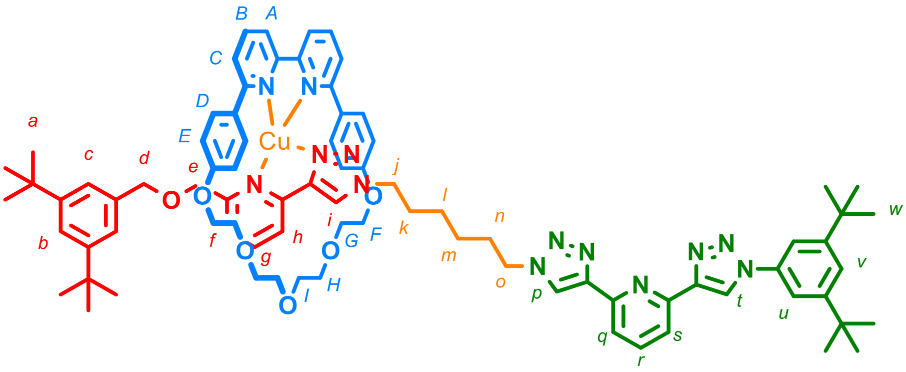

[Cu(9e)](PF_6):

Two species were observed in an approximately 1:1.7 ratio. As much as has been possible the ^{1}H NMR signals belonging to both the major and minor species have been assigned separately.

Major species:

^{1}H NMR ($CDCl_3$, 500 MHz) δ: 8.71 (s, 1H, H_t), 8.55 (s, 1H, H_i), 8.45 (s, 1H, H_p), 8.18-7.82 (m, H_A, H_B, H_q, H_r, H_s, H_g), 7.70 (d, J = 7.5, 1H, H_h), 7.64-7.63 (m, 4H, H_C, H_u), 7.51 (t, J = 1.6, 1H, H_v), 7.41 (d, J = 7.7, 1H, H_f), 7.31 (t, J = 1.8, 1H, H_b), 7.07 (d, J = 8.5, 4H, H_D), 6.98 (d, J = 1.8, 2H, H_c), 6.06 (d, J = 8.6, 4H, H_E), 4.51 (t, J = 6.8, 2H, H_o), 4.45-4.40 (m, H_j), 4.16 (s, 2H, H_d), 3.86 (s, 2H, H_e), 3.82-3.56 (m, H_F, H_G, H_H, H_I), 2.09-2.04 (m, 2H, H_n), 2.00-1.95 (m, H_k), 1.55-1.46 (m, H_m), 1.42-1.26 (m, H_l), 1.39 (s, 18H, H_w), 1.26 (s, 18H, H_a).

Minor species:

^{1}H NMR ($CDCl_3$, 500 MHz) δ: 8.94 (s, 1H, $H_{t'}$), 8.20 (s, 1H, $H_{i'/p'}$), 8.18-7.82 (m, $H_{A'}$, $H_{i'/p'}$, $H_{B'}$, $H_{q'}$, $H_{r'}$, $H_{s'}$), 7.76 (app. t, J = 7.8, 1H, $H_{g'}$), 7.60 (d, J = 1.7, 2H, $H_{u'}$), 7.58-7.55 (m, 3H, $H_{v'}$, $H_{C'}$), 7.44 (d, J = 7.6, 1H, $H_{f'}$), 7.36 (t, J = 1.8, 1H, $H_{b'}$), 7.23 (d, J = 1.8, 2H, $H_{c'}$), 7.21 (d, J = 8.5, 4H, $H_{D'}$), 6.14 (d, J = 8.6, 4H, $H_{E'}$), 6.06 (d, J = 8.6, 4H, H_E), 4.73 (s, 2H, $H_{e'}$), 4.64 (s, 2H, $H_{d'}$), 4.45-4.40 (m, $H_{j'/o'}$), 4.12 (t, J = 7.2, 2H, $H_{j'/o'}$), 3.82-3.56 (m, $H_{F'}$, $H_{G'}$, $H_{H'}$, $H_{I'}$), 2.00-1.95 (m, $H_{k'/n'}$), 1.77-1.72 (m, $H_{k'/n'}$), 1.55-1.46 (m, $H_{l'/m'}$), 1.42-1.26 (m, $H_{l'/m'}$), 1.42 (s, 18H, $H_{w'}$), 1.32 (s, 18H, $H_{a'}$).

^{13}C NMR ($CDCl_3$, 125 MHz) δ: 159.3, 159.1, 158.9, 158.9, 158.8, 155.7, 153.3, 152.9, 152.5, 152.4, 151.1, 151.0, 151.00, 150.4, 150.2, 149.8, 148.8, 148.5, 148.3, 148.2, 146.7, 146.1, 145.7, 145.4, 144.4, 138.7, 138.5, 138.4, 138.3, 137.7, 137.5, 137.2, 136.9, 136.6, 136.3, 134.0, 133.6, 129.5, 129.1, 124.0, 123.8, 123.6, 123.5, 123.2, 123.2, 123.1, 122.5, 122.2, 122.2, 122.0, 122.0, 121.8, 121.5, 121.5, 121.4, 120.9, 120.7, 120.4, 120.1, 119.4, 119.4, 118.9, 116.2, 115.6, 115.3, 113.4, 113.3, 74.0, 73.8, 73.8, 73.4, 72.2, 71.6, 71.1, 71.1, 70.7, 69.9, 69.3, 68.3, 68.1, 51.1, 50.3, 50.3, 50.3, 35.5, 35.3, 35.0, 34.9, 31.6, 31.6, 31.5, 30.1, 30.1, 30.0, 29.8, 25.9, 25.8.

4. Exchange Spectroscopy (EXSY) Investigation of Shuttling Process of [Cu(9e)](PF_6)

The ROESY NMR cross-peaks of the methylene signals H_d and H_e of the thread were used to evaluate the shuttling of the macrocycle between the two stations as these peaks were significantly shifted between the major and minor species and did not overlap with other peaks. ROESY NMR spectra were run at several mixing times and the integrations of the diagonal- and cross-peaks from each of these spectra used to obtain values for $\Delta G^{\ddagger}$ which were found to be consistent (*vide infra*).

$$r = \frac{(I_{AA} + I_{BB})}{(I_{AB} + I_{BA})}$$

$$k = \frac{1}{\tau_m} \times \ln\frac{r+1}{r-1}$$

$$\Delta G^{\ddagger} = -RT \ln\frac{kh}{k_B T}$$

Where R is the gas constant (1.987 cal K^{-1} mol^{-1}); k_B is the Boltzmann constant (3.30×10^{-24} cal K^{-1}) and h is Planck's constant (1.58×10^{-34} cal s).

τ_m (s)	I_{AA}	I_{AB}	I_{BB}	I_{BA}	k (s^{-1})	$\Delta G^{\ddagger}$ (kcal mol^{-1})
0.10	2.2×10^3	1.0	2.4×10^3	0.61	5.6×10^{-3}	21
0.20	8.3×10^2	1.0	1.4×10^3	0.70	7.7×10^{-3}	20
0.30	7.3×10^2	1.0	1.2×10^3	0.74	6.2×10^{-3}	21

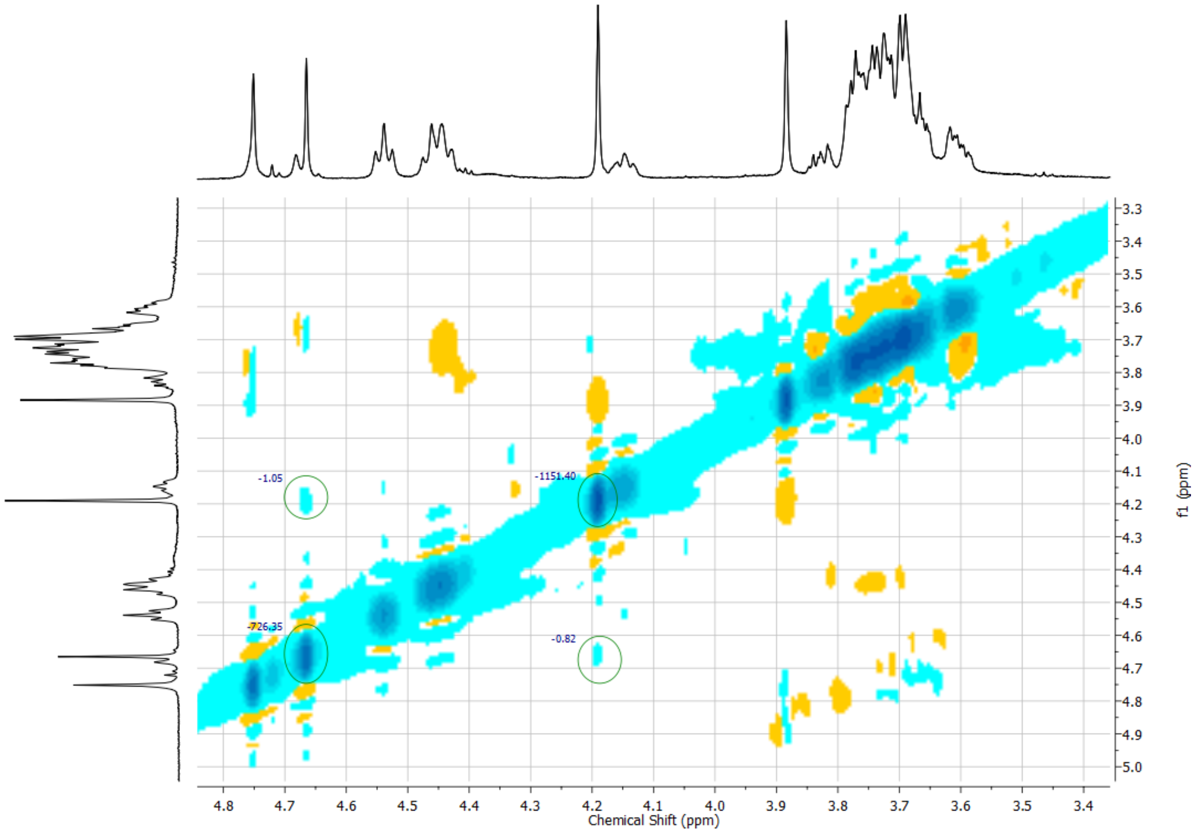

Figure S3 Example ^{1}H ROESY NMR ($CDCl_3$, 500 MHz) with τ_m = 300 ms.

Rotaxane Stack Plots Demonstrating Conversion of Macrocycles 2

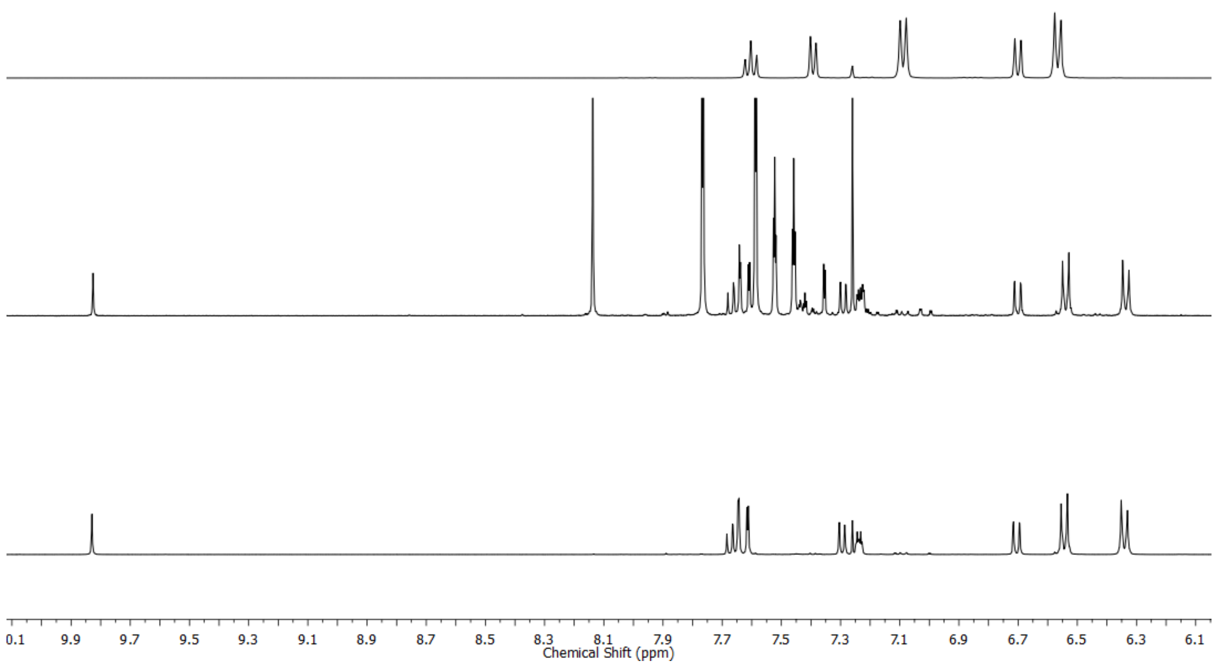

Figure S4 Partial ^{1}H NMR ($CDCl_3$, 400 MHz) spectra of **2d** (top), crude **S11** (middle) and purified **S11** (bottom). Quantitative conversion of **2d** is observed.

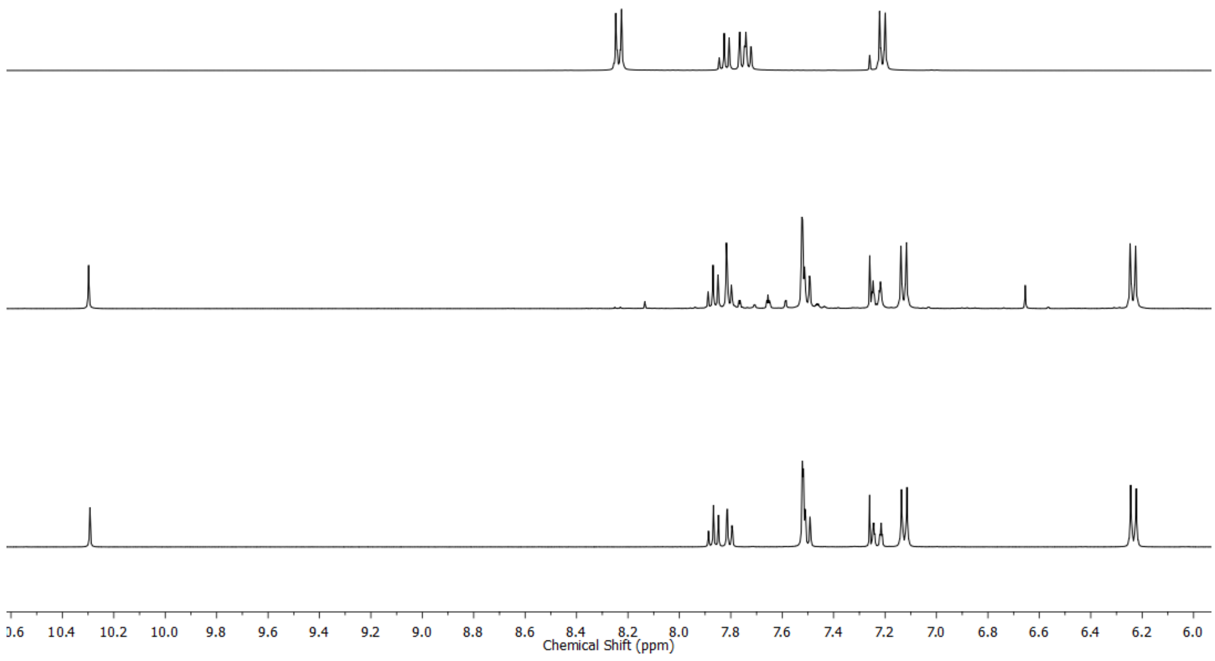

Figure S5 Partial ^{1}H NMR ($CDCl_3$, 400 MHz) spectra of **2e** (top), crude **S12** (middle) and purified **S12** (bottom). Quantitative conversion of **2e** is observed.

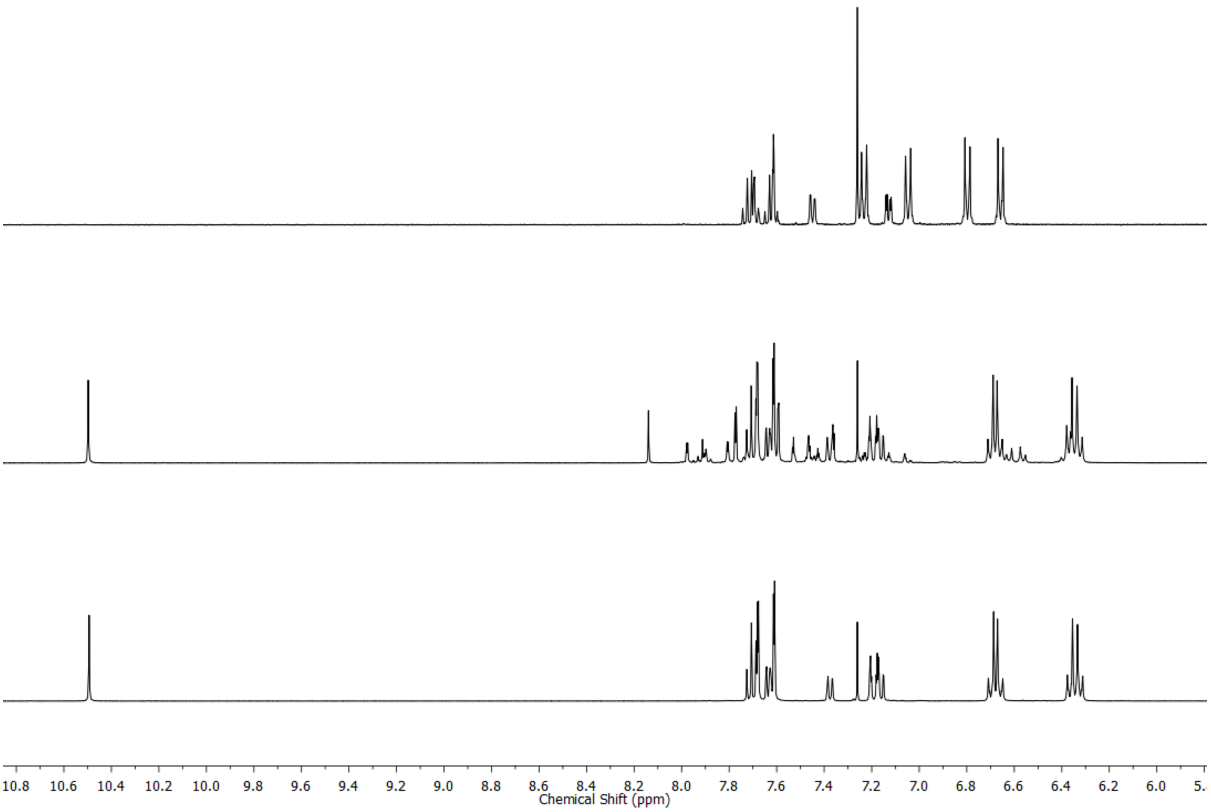

Figure S6 Partial ^{1}H NMR ($CDCl_3$, 400 MHz) spectra of **2f** (top), crude **S13** (middle) and purified **S13** (bottom). Quantitative conversion of **2f** is observed**.**

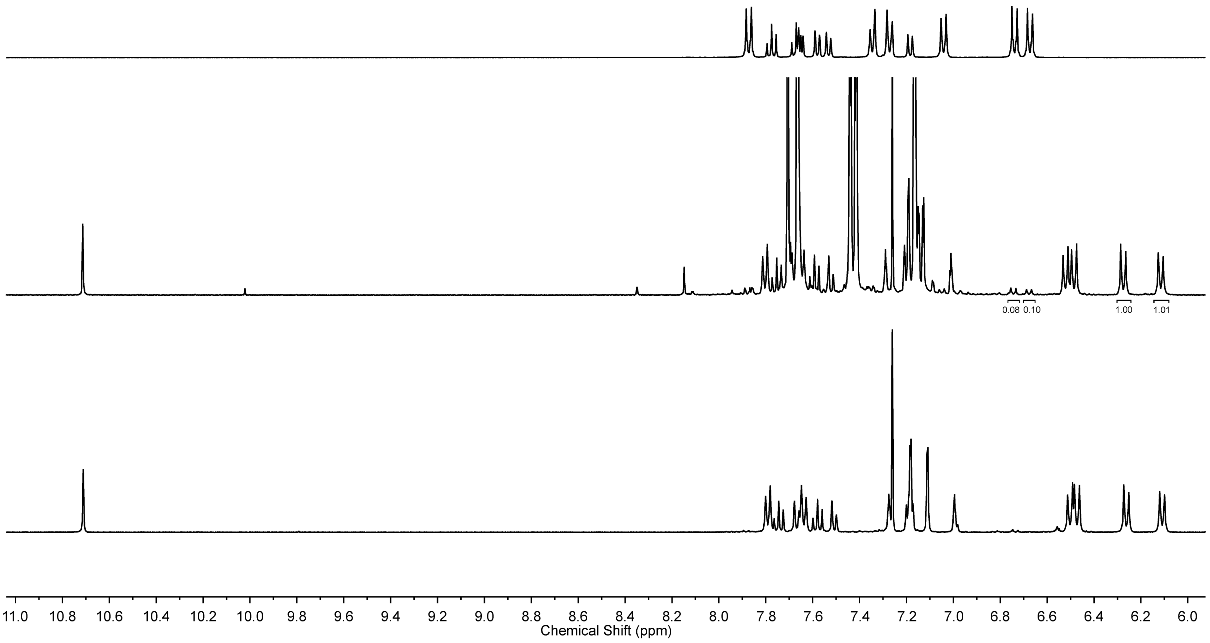

Figure S7 Partial ^{1}H NMR ($CDCl_3$, 400 MHz) spectra of **2h** (top), crude **S14** (middle) and purified **S14** (bottom). Conversion of **2h** to rotaxane **S14** was 93% (calculated using the integrals of the flanking aromatic units as indicated).

5. Graphical NMR Data including 2D Spectra

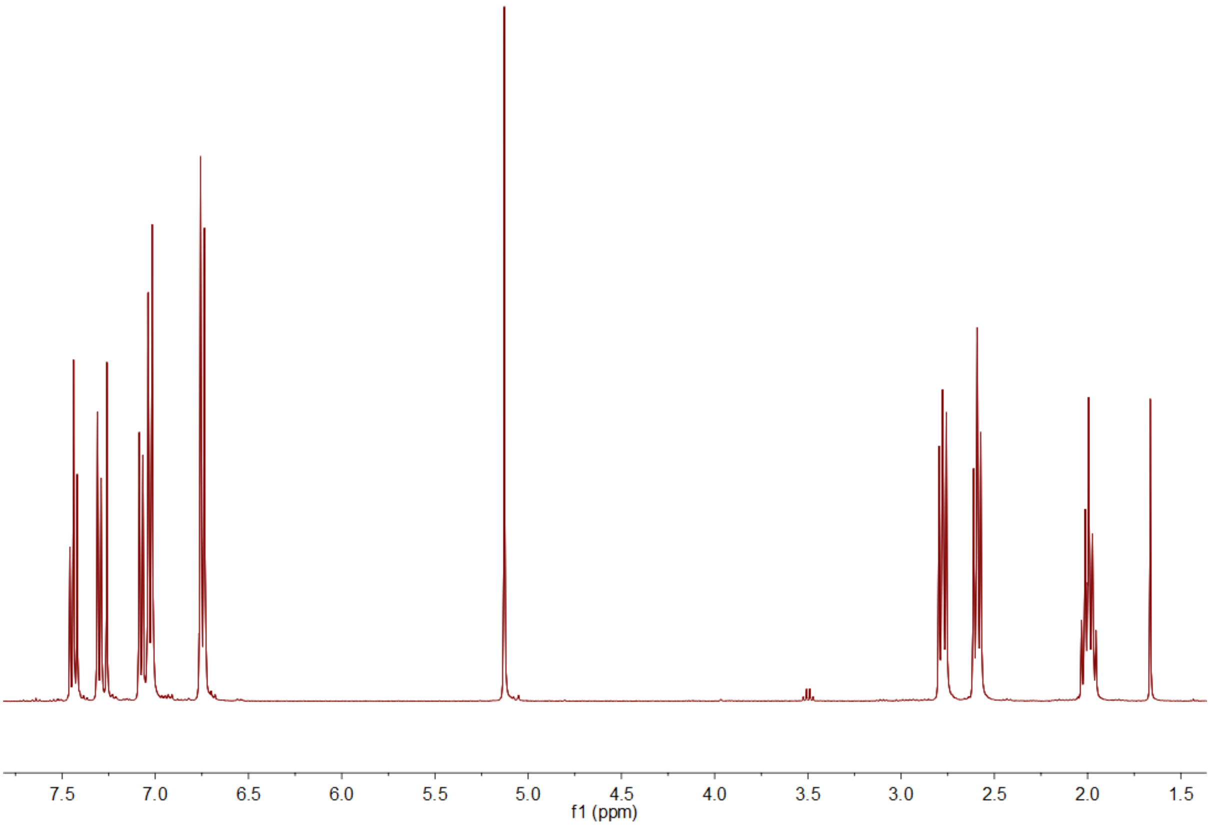

Figure S8 ^{1}H NMR ($CDCl_3$, 400 MHz) of S1.

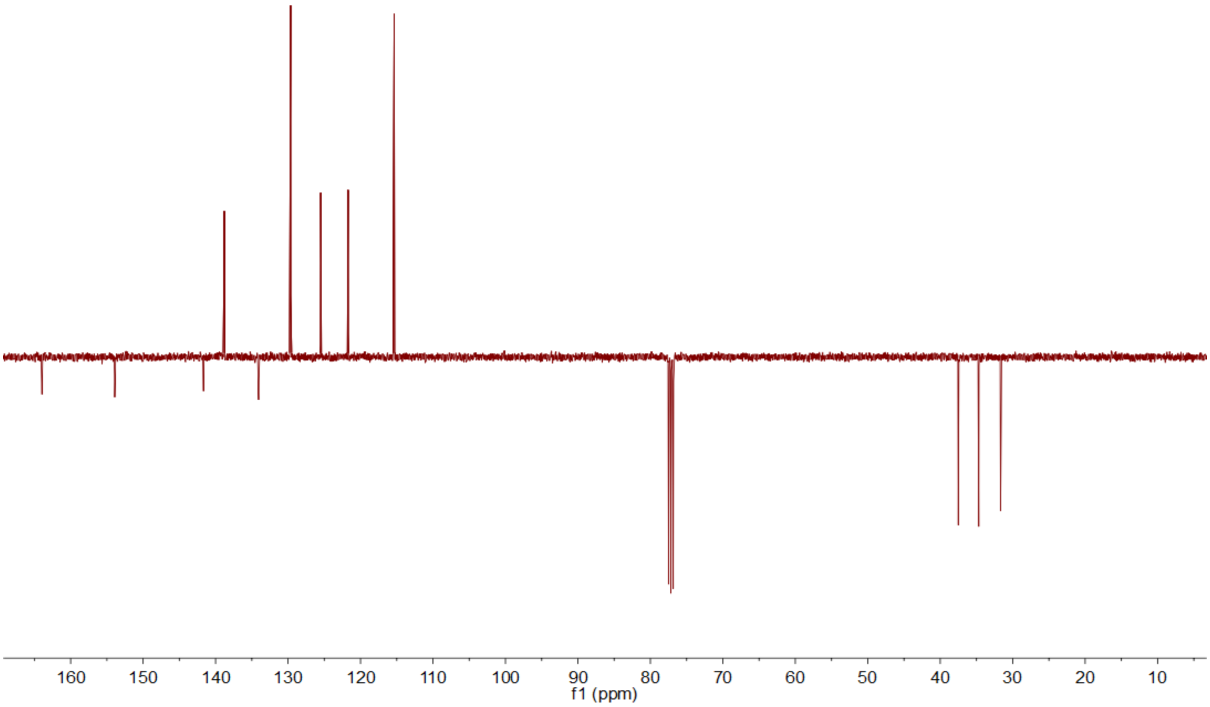

Figure S9 JMOD NMR ($CDCl_3$, 100 MHz) of S1.

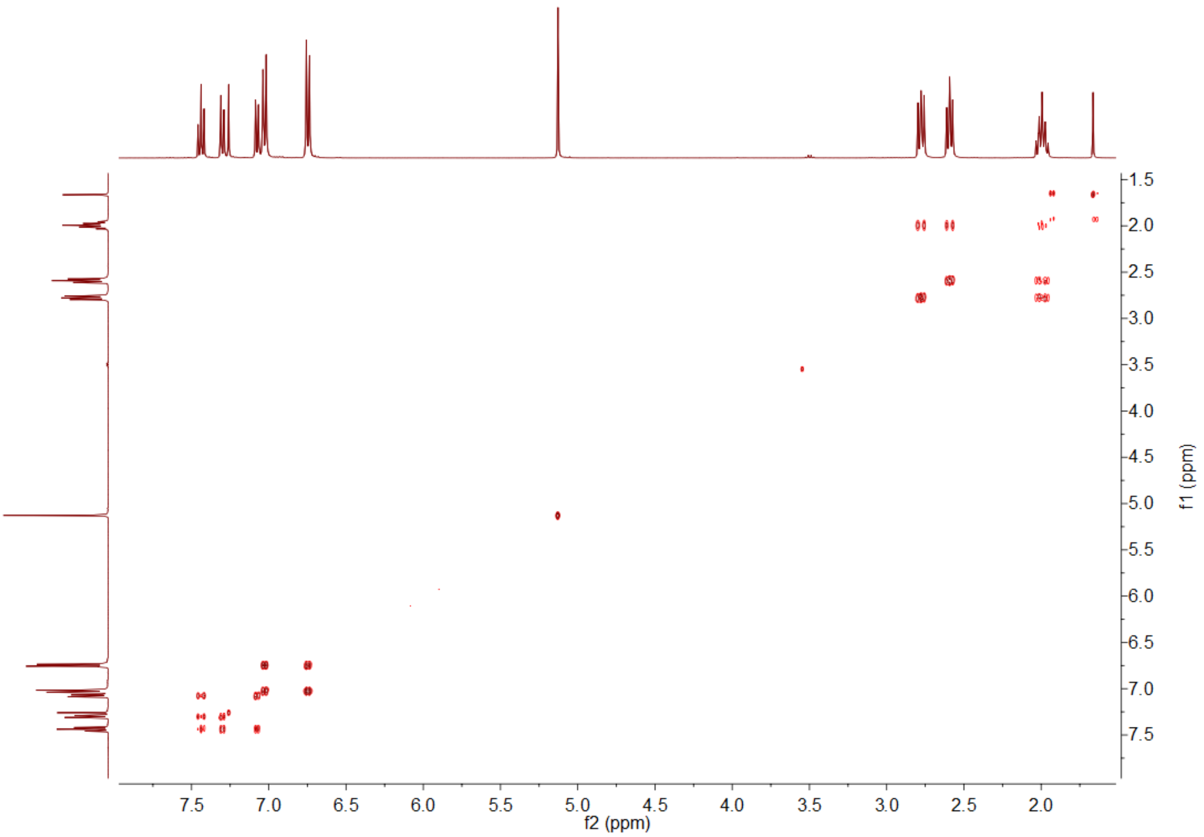

Figure S10 ^{1}H COSY NMR ($CDCl_3$, 400 MHz) of S1.

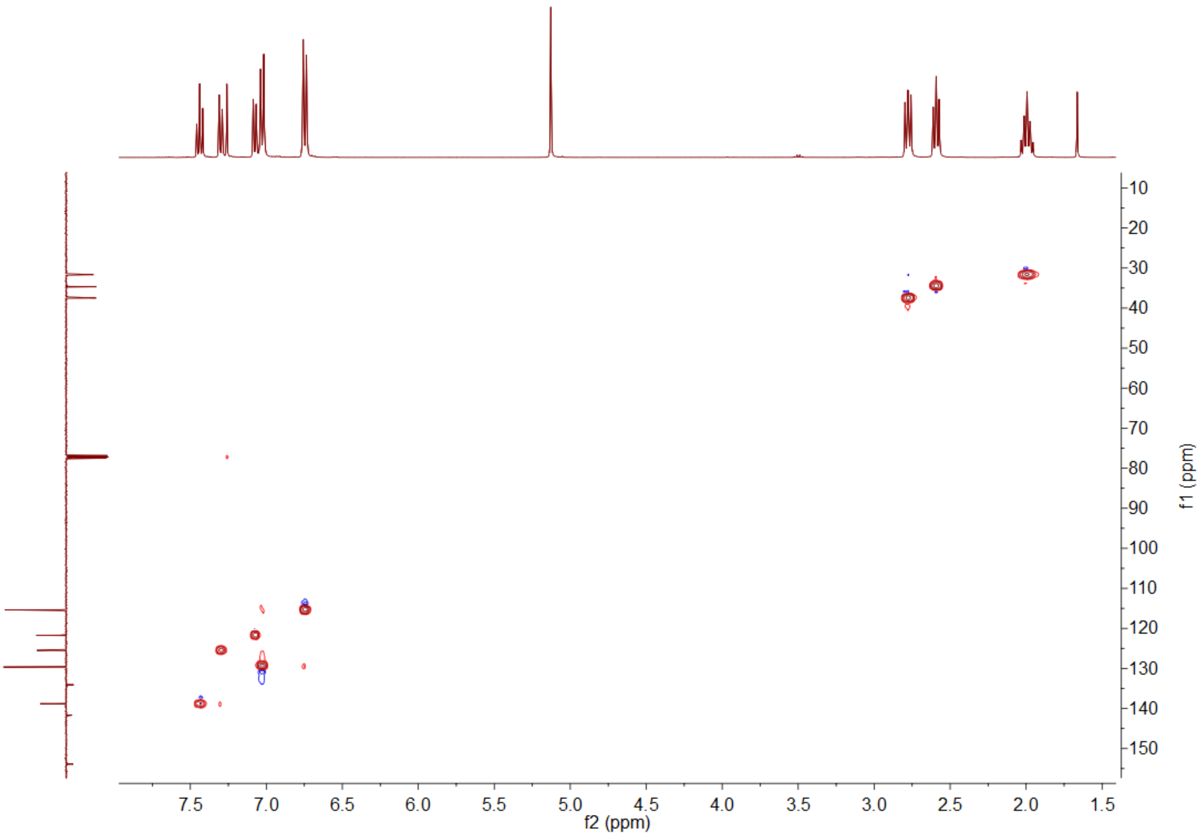

Figure S11 HSQC NMR ($CDCl_3$, 400 MHz) of S1.

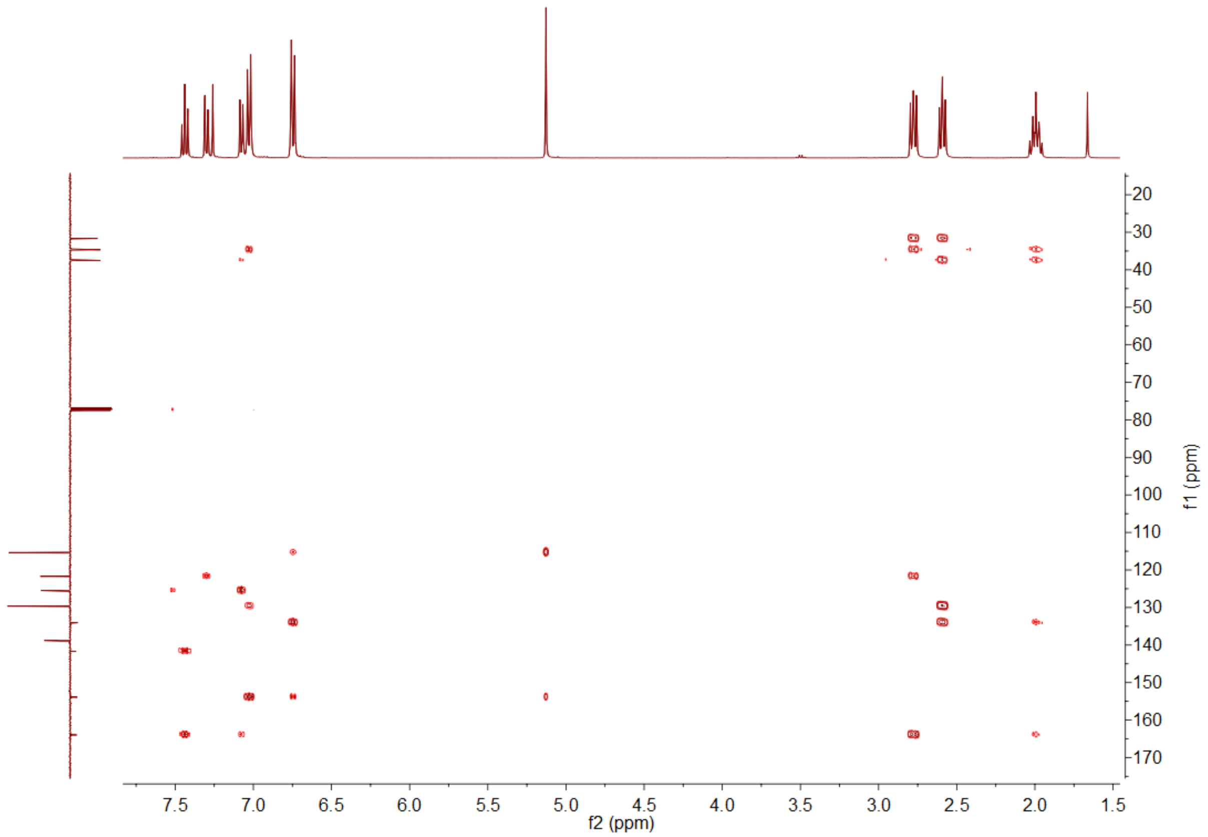

Figure S12 HMBC NMR ($CDCl_3$, 400 MHz) of S1.

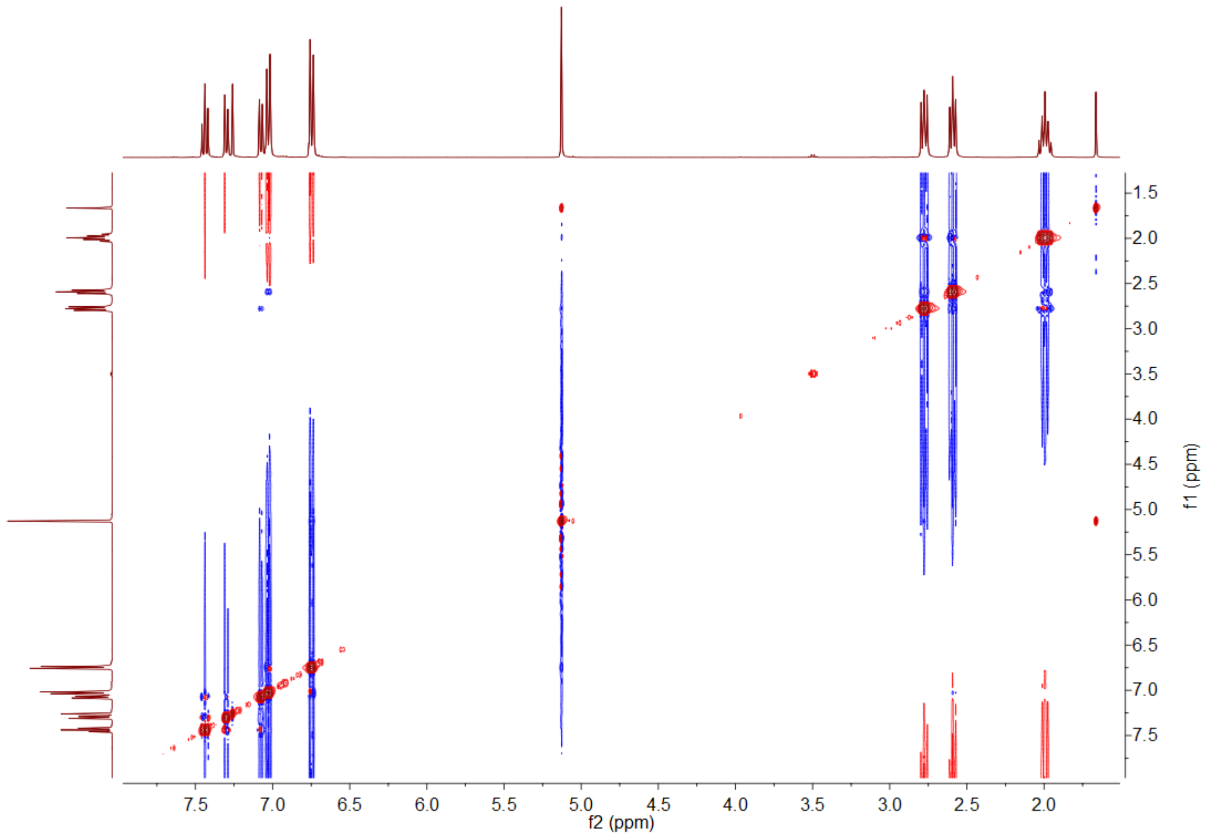

Figure S13 ^{1}H NOESY NMR ($CDCl_3$, 400 MHz) of S1.

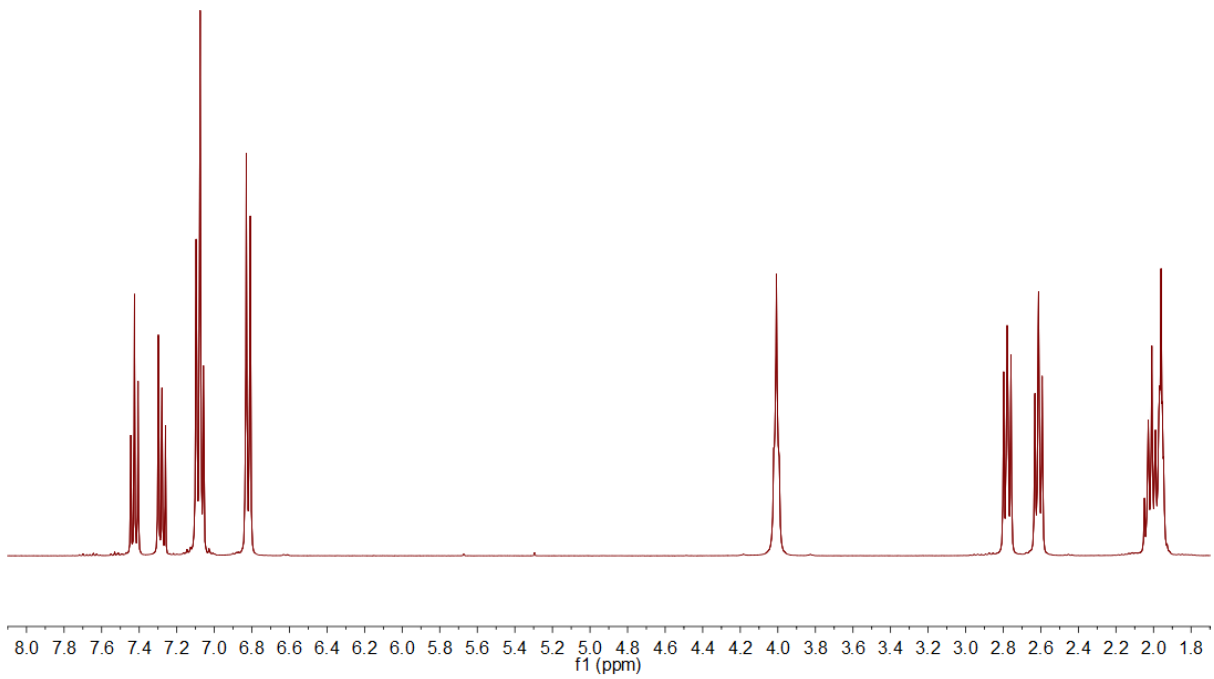

Figure S14 ^{1}H NMR ($CDCl_3$, 400 MHz) of 1a.

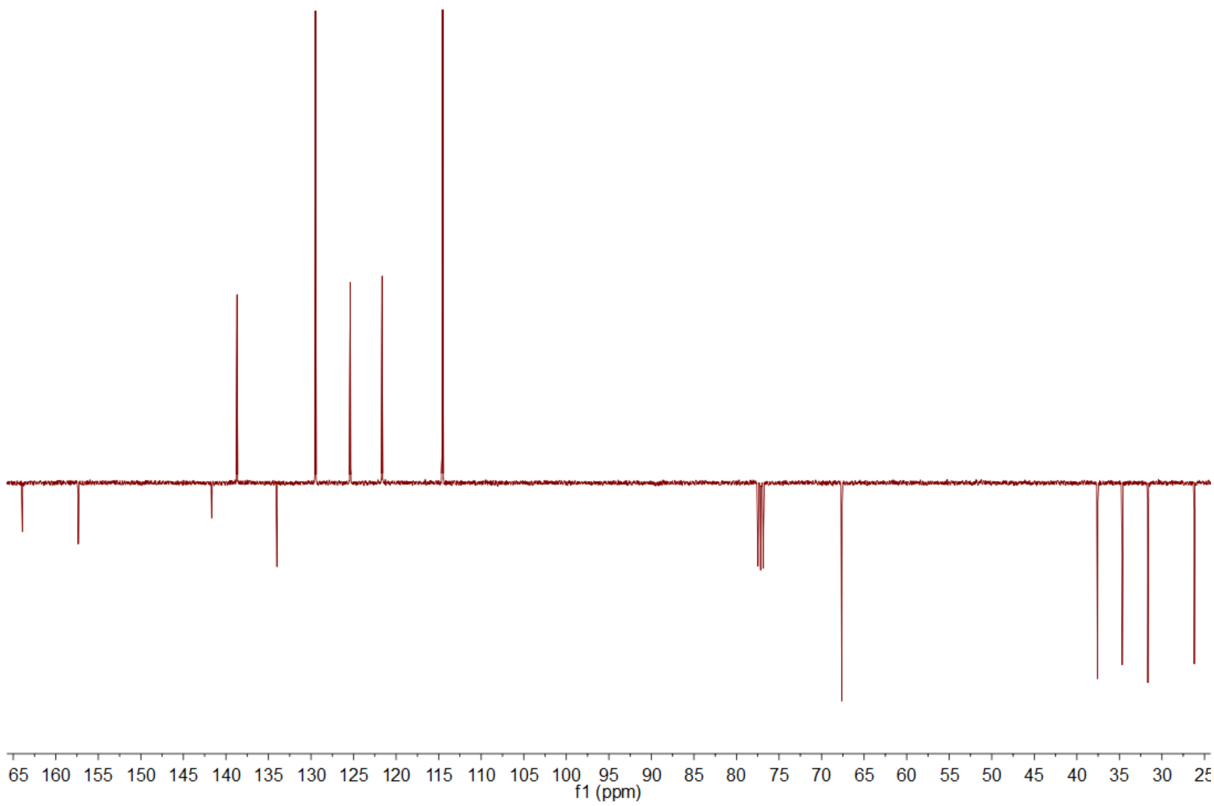

Figure S15 JMOD NMR ($CDCl_3$, 100 MHz) of 1a.

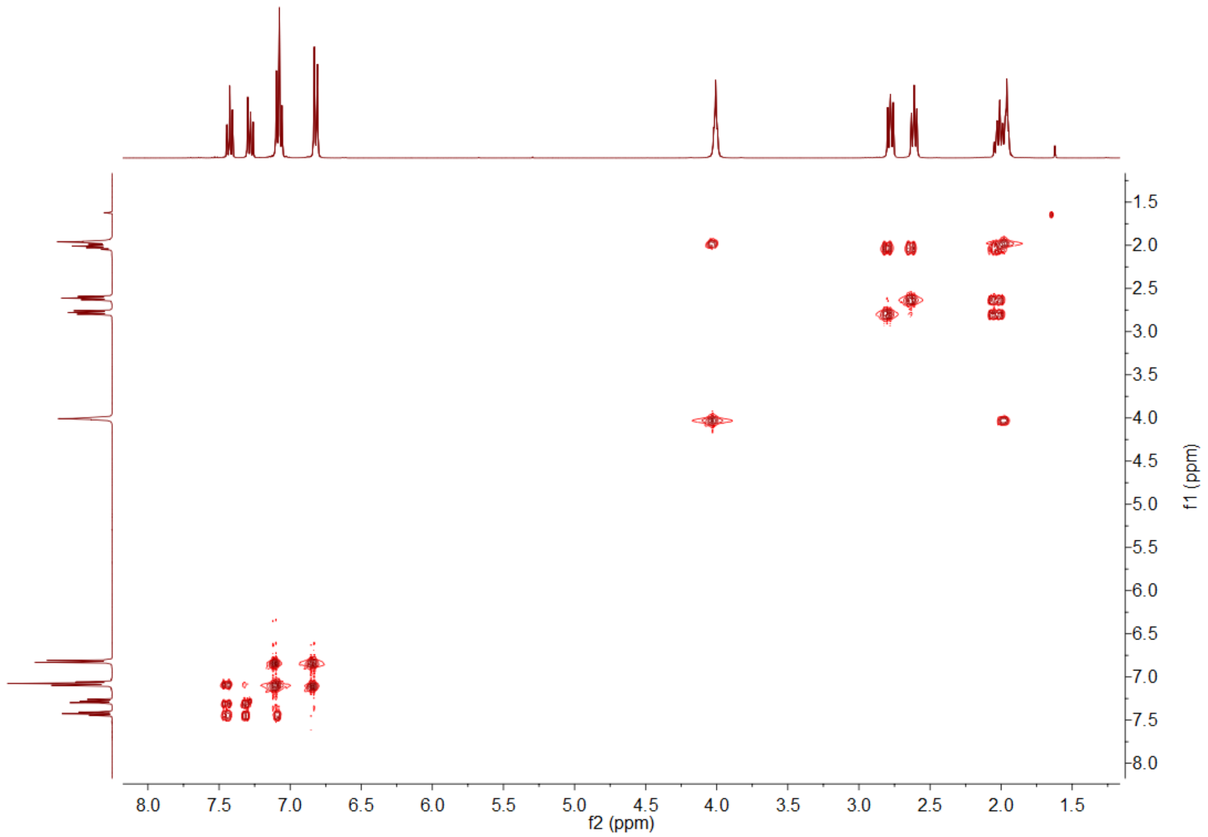

Figure S16 ^{1}H COSY NMR ($CDCl_3$, 400 MHz) of 1a.

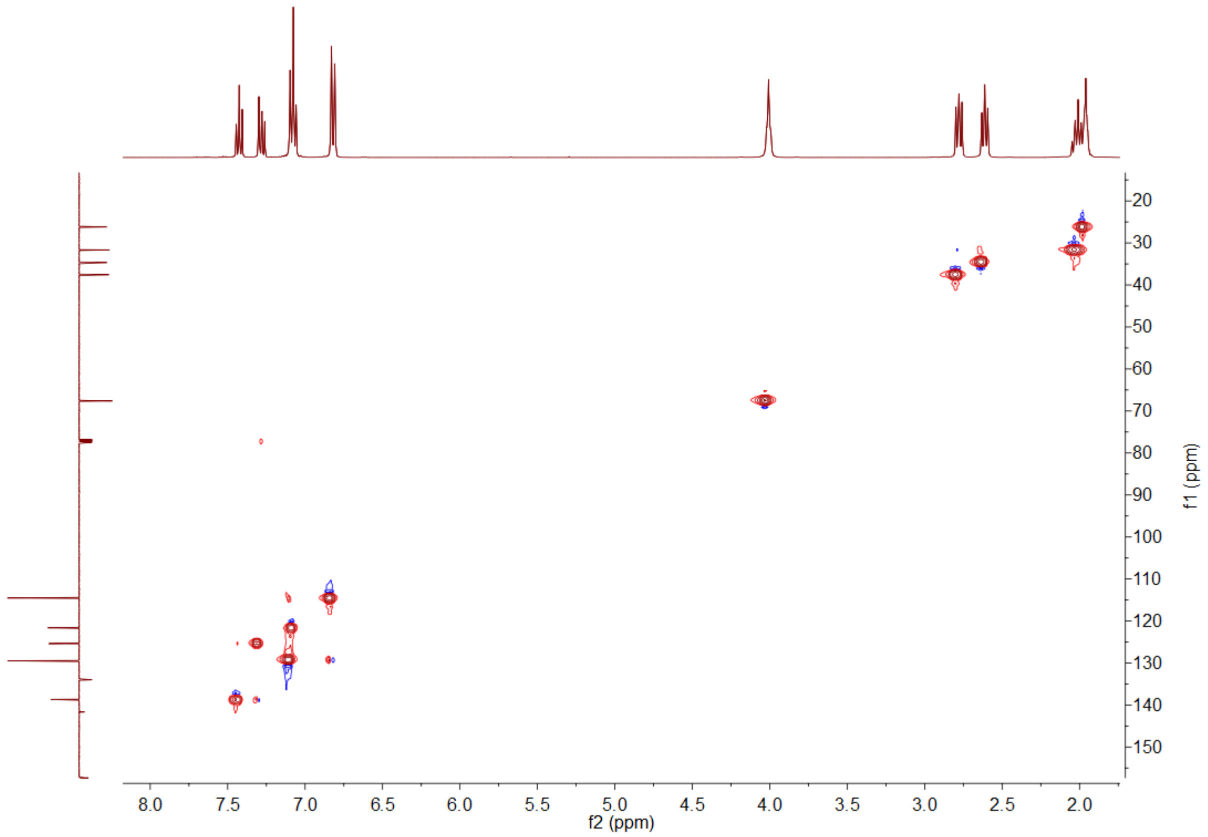

Figure S17 HSQC NMR (CDCl3, 400 MHz) of 1a.

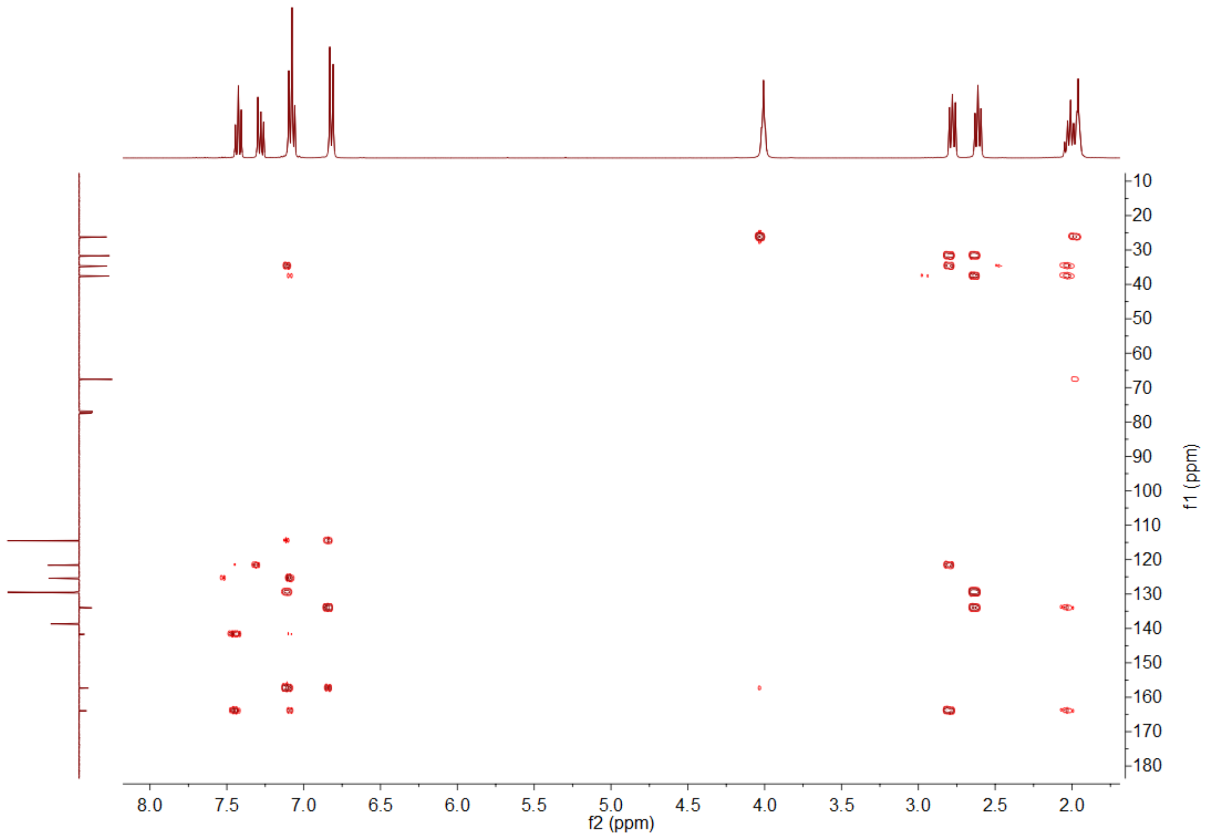

Figure S18 HMBC NMR (CDCl3, 400 MHz) of 1a.

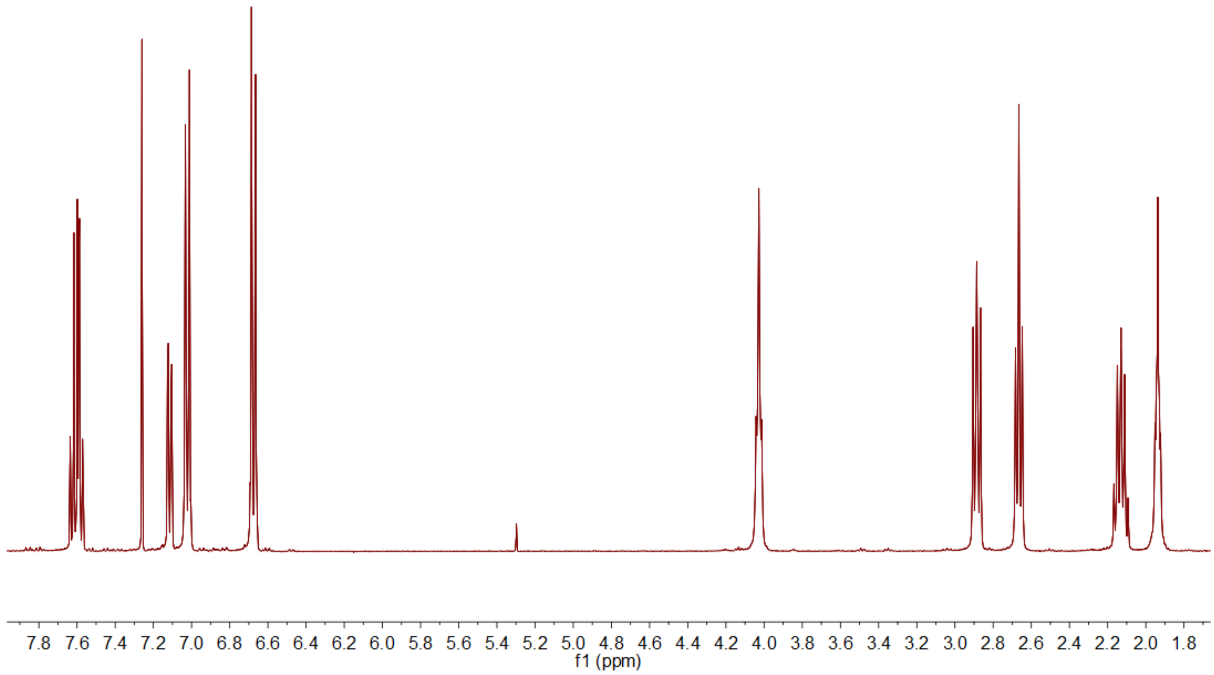

Figure S19 ^{1}H NMR ($CDCl_3$, 400 MHz) of 2a.

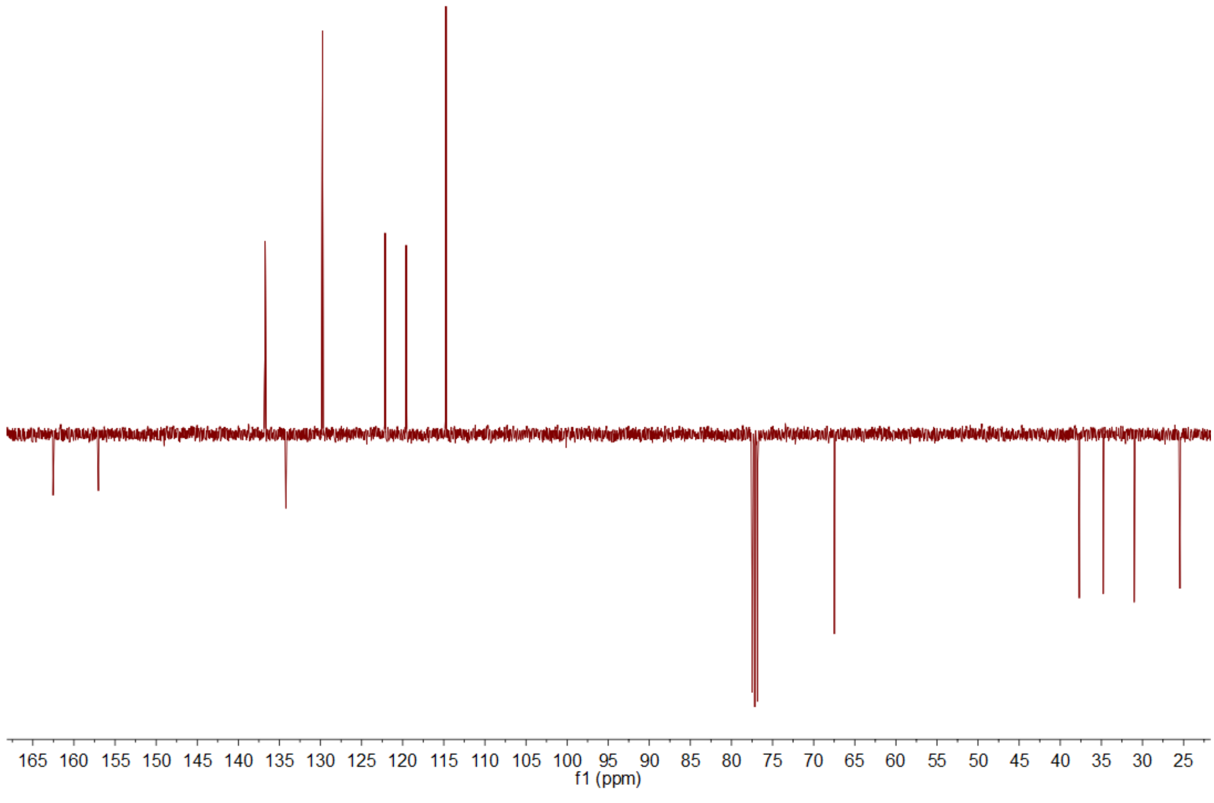

Figure S20 JMOD NMR ($CDCl_3$, 100 MHz) of 2a.

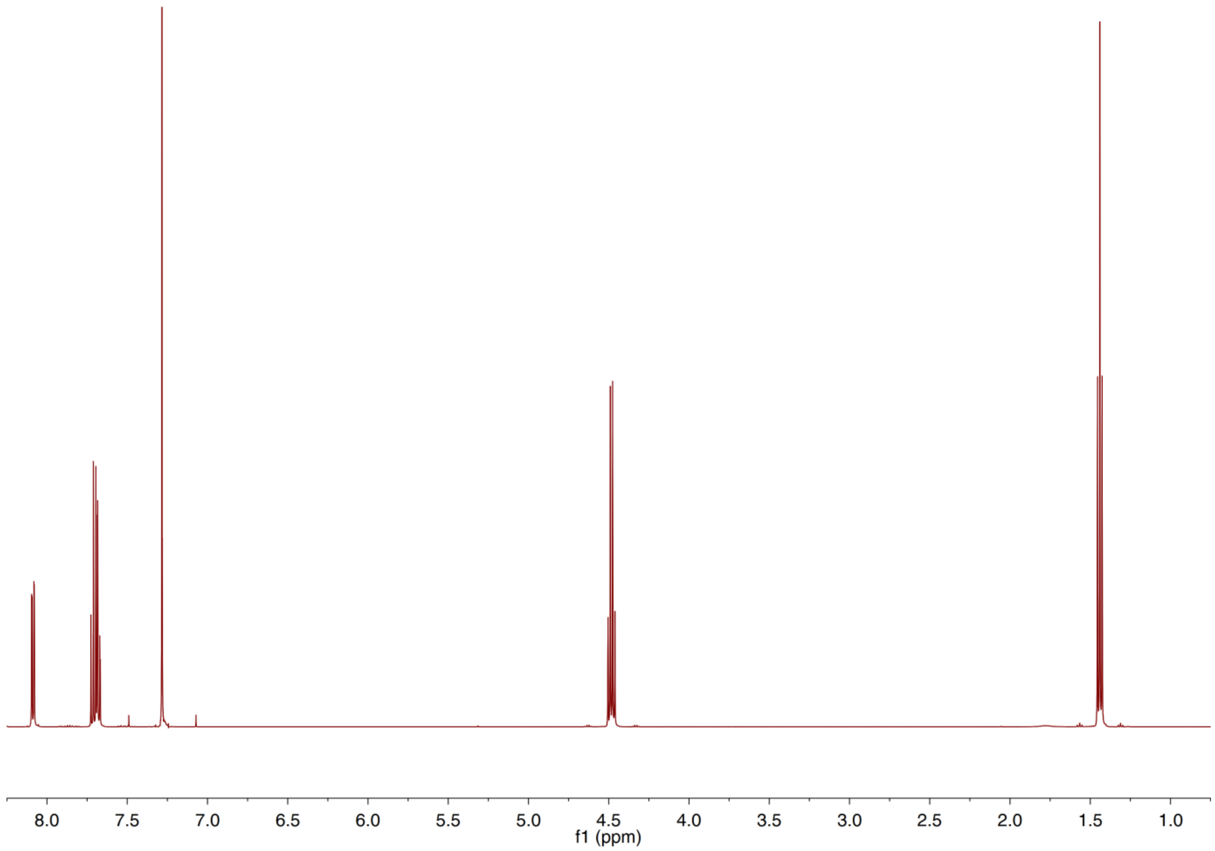

Figure S21 ^{1}H NMR ($CDCl_3$, 400 MHz) of S2.

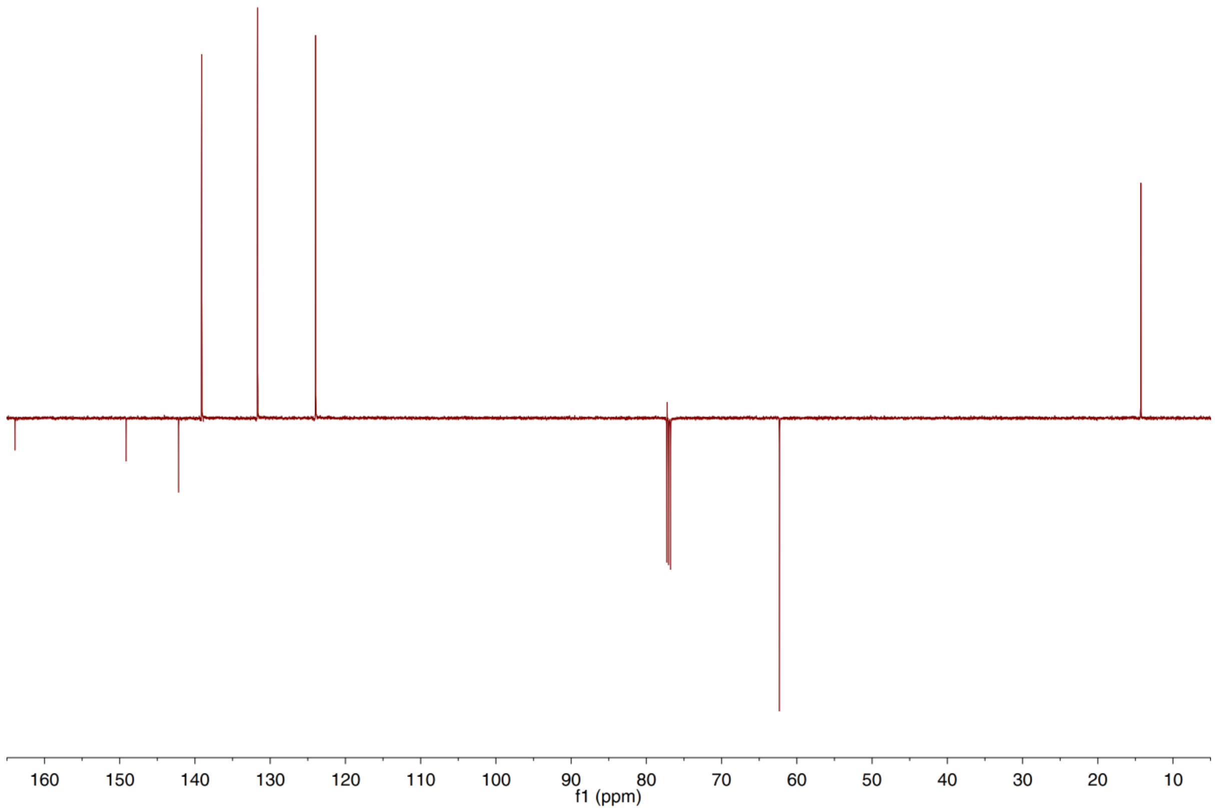

Figure S22 JMOD NMR ($CDCl_3$, 100 MHz) of S2.

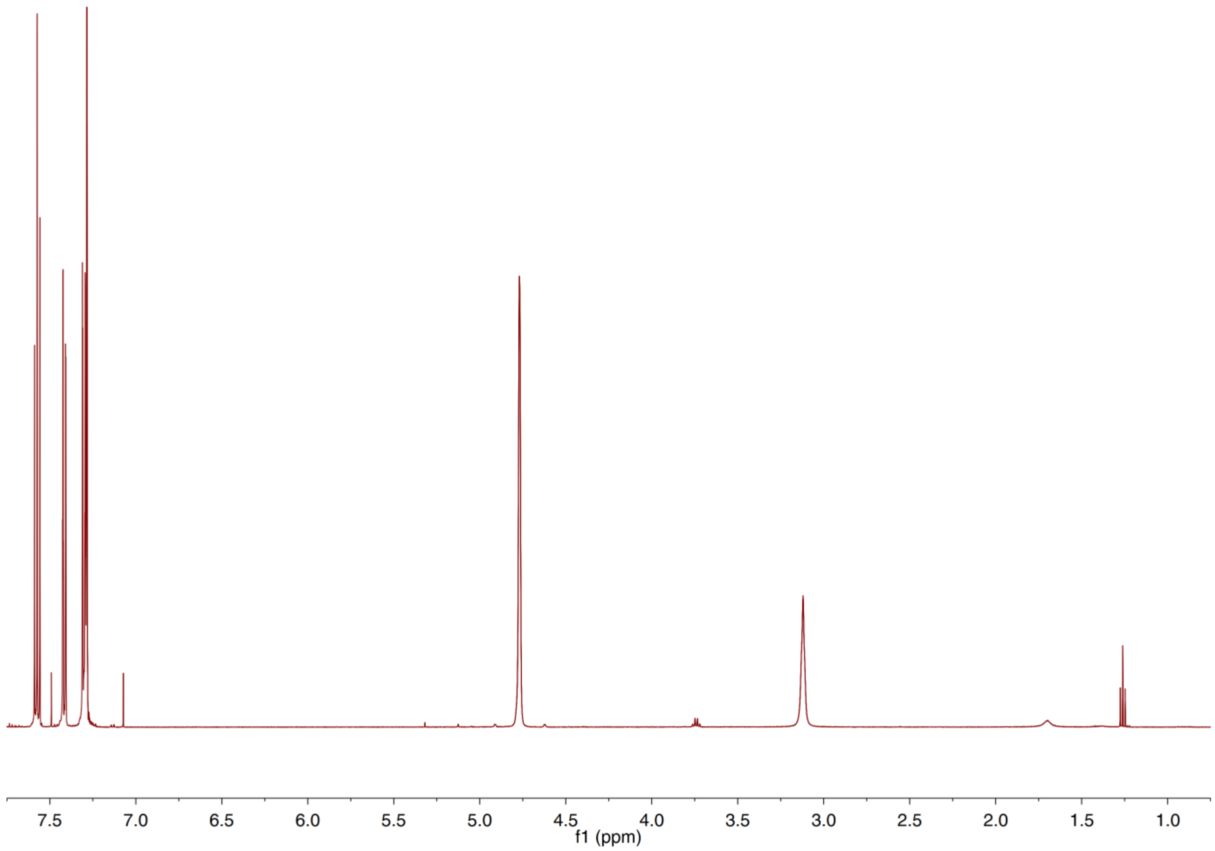

Figure S23 ^{1}H NMR ($CDCl_3$, 400 MHz) of S3.

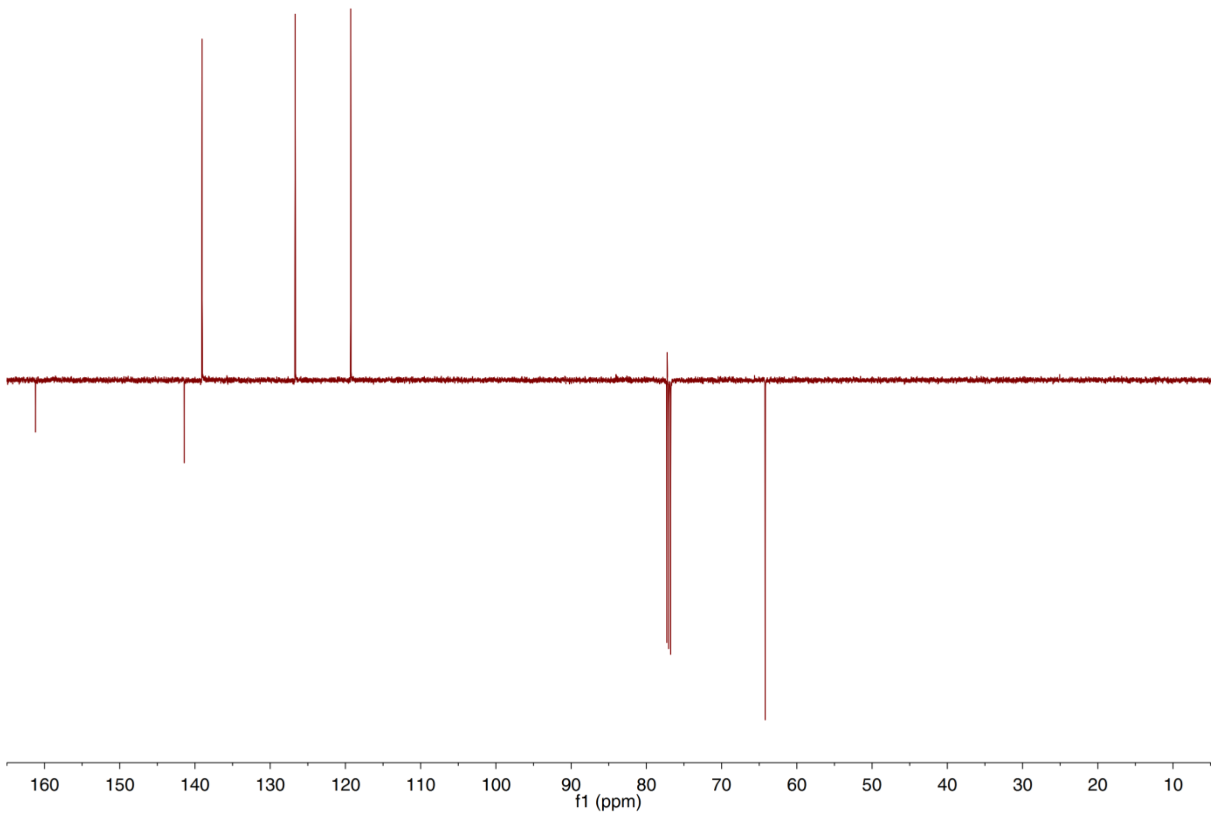

Figure S24 JMOD NMR ($CDCl_3$, 100 MHz) of S3.

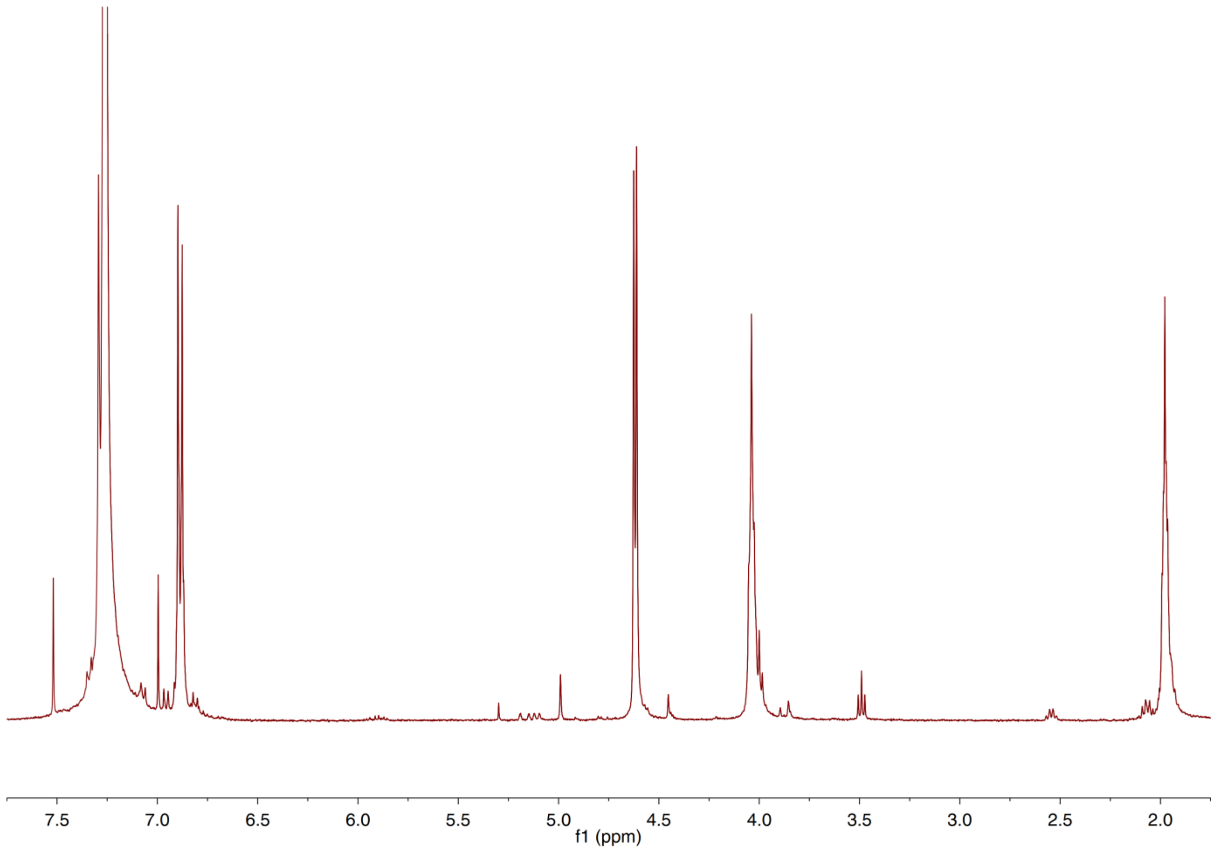

Figure S25 ^{1}H NMR ($CDCl_3$, 400 MHz) of 1,4-di(4-(hydroxymethyl)phenoxy)butane.

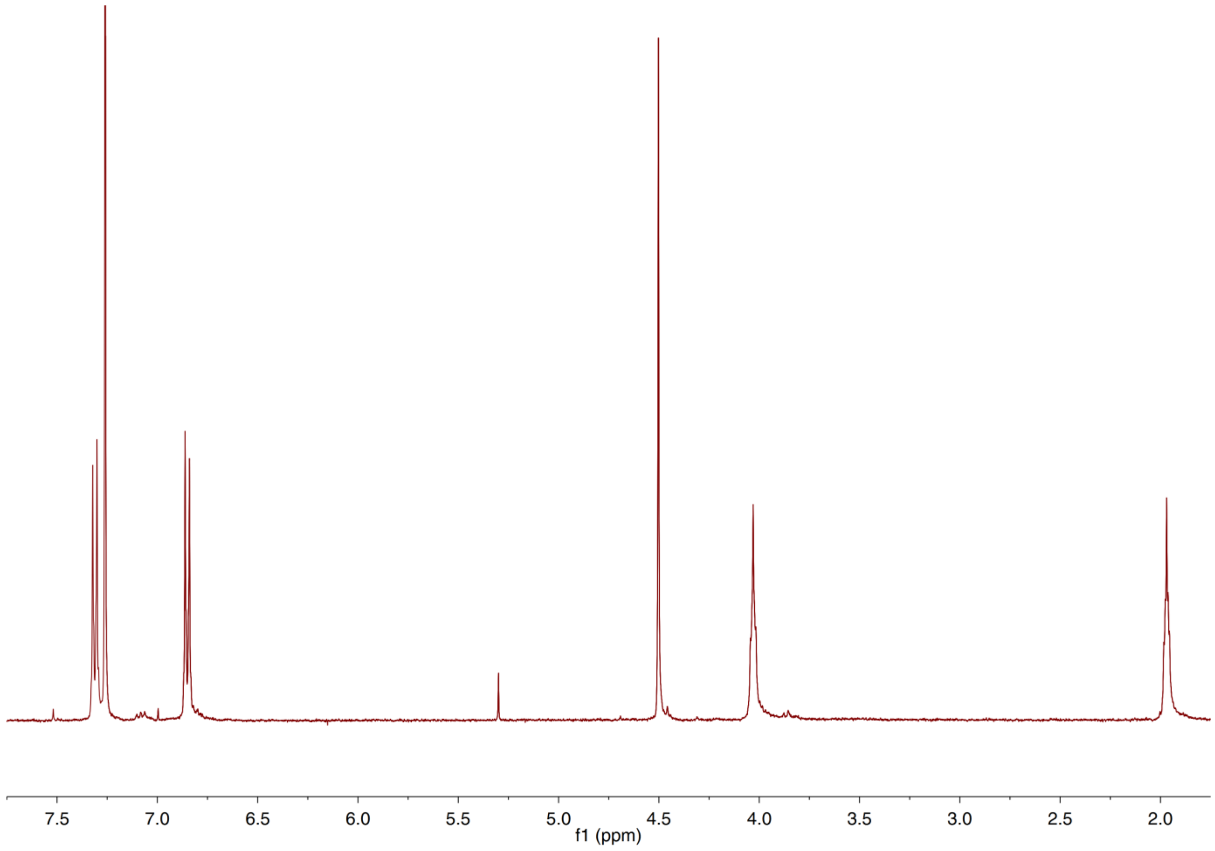

Figure S26 ^{1}H NMR ($CDCl_3$, 400 MHz) of 1,4-di(4-(bromomethyl)phenoxy)butane.

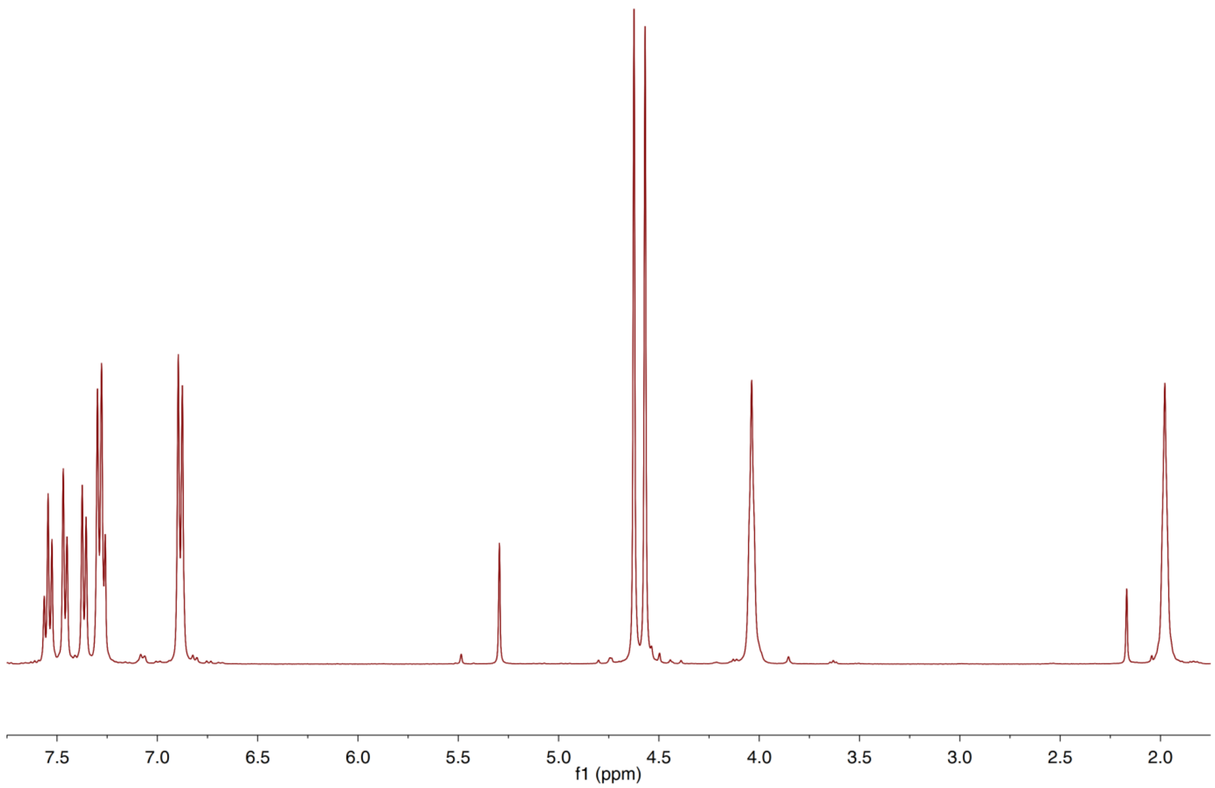

Figure S27 ^{1}H NMR ($CDCl_3$, 400 MHz) of 1b.

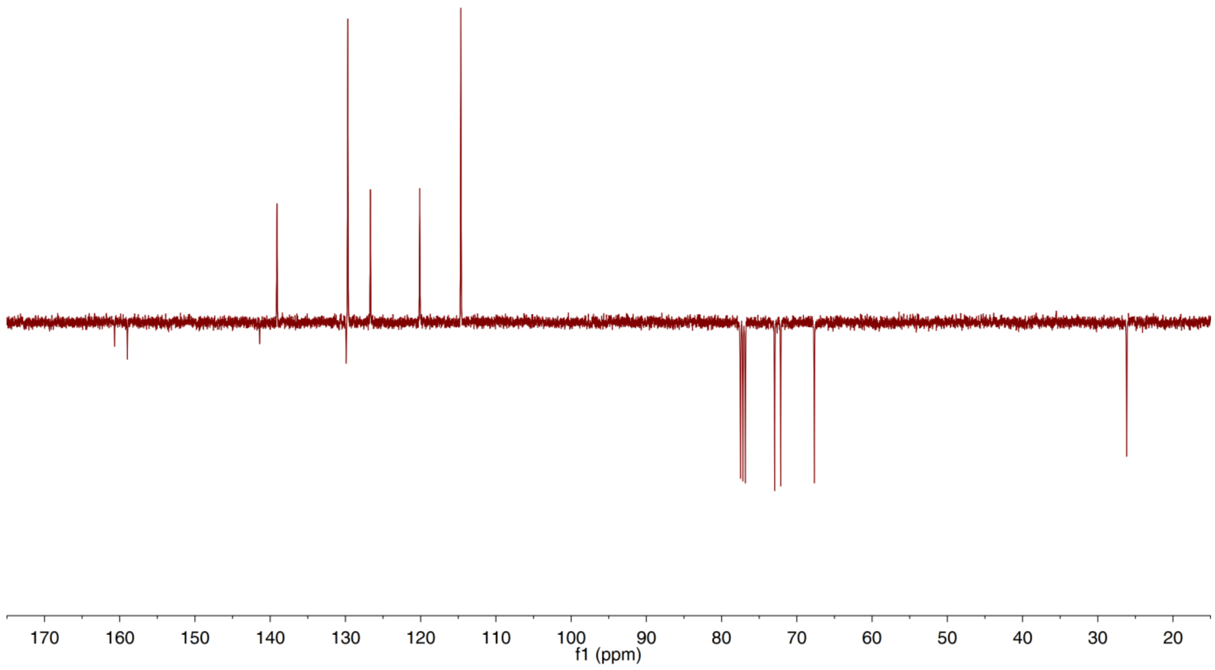

Figure S28 JMOD NMR ($CDCl_3$, 100 MHz) of 1b.

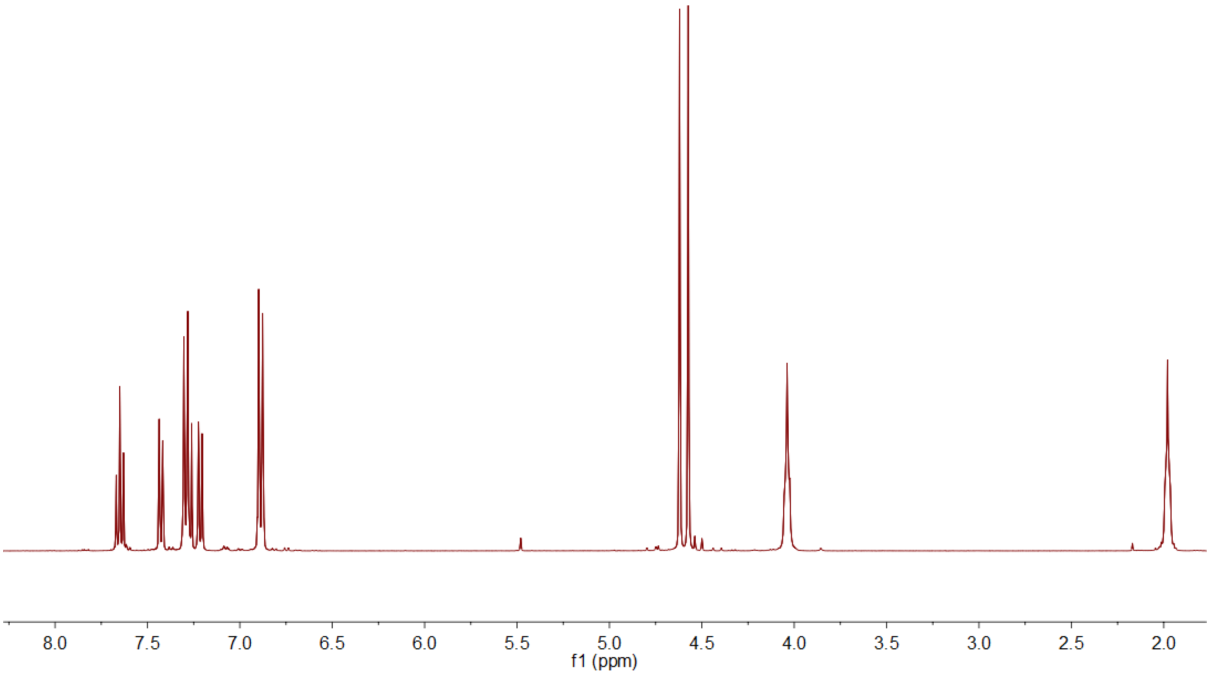

Figure S29 ^{1}H NMR ($CDCl_3$, 400 MHz) of S4.

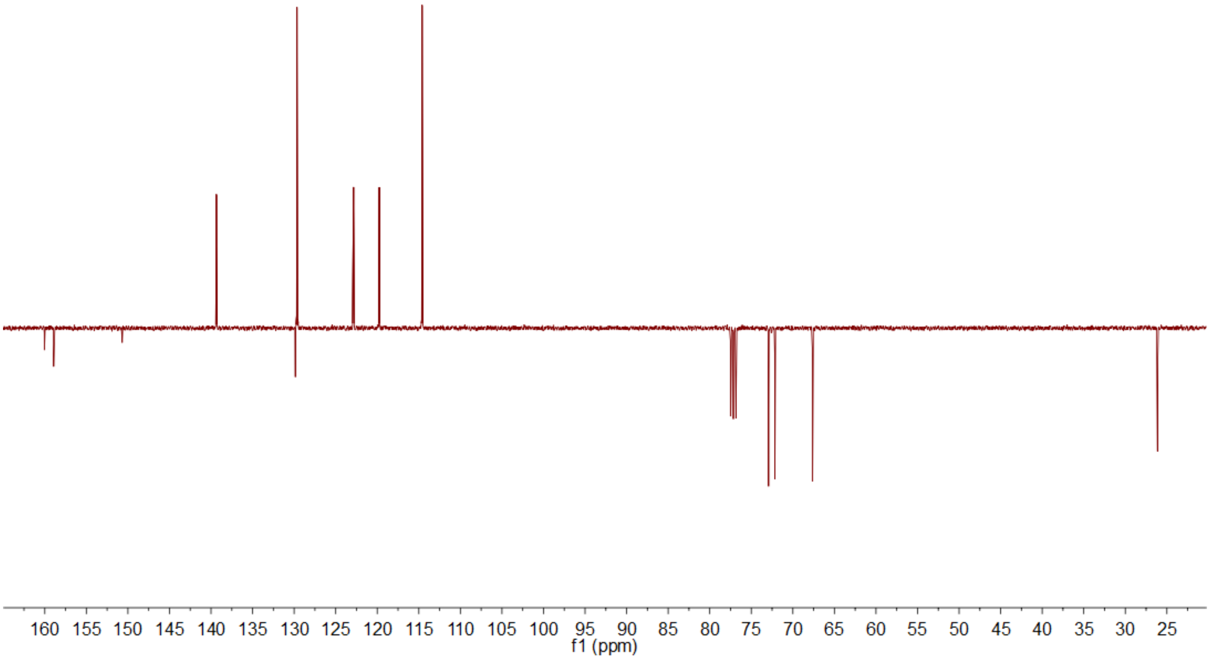

Figure S30 JMOD NMR ($CDCl_3$, 100 MHz) of S4.

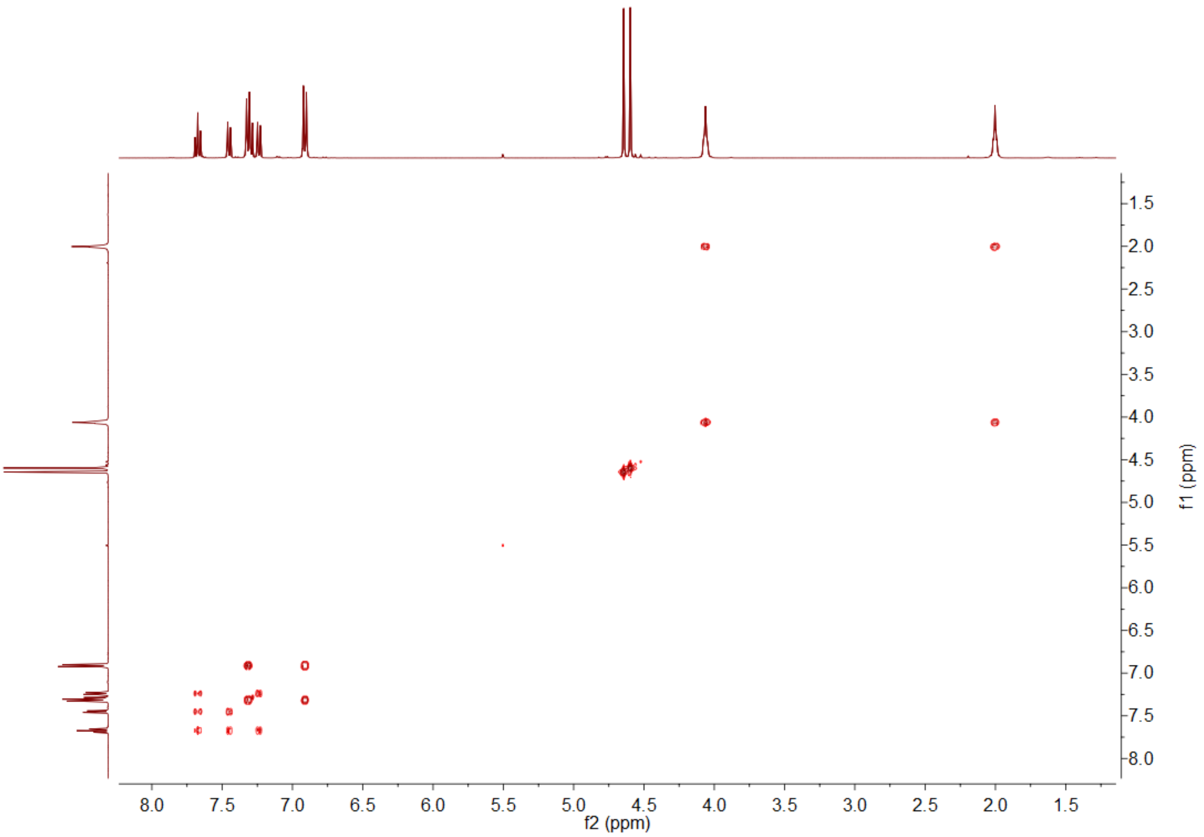

Figure S31 ^{1}H COSY NMR ($CDCl_3$, 400 MHz) of S4.

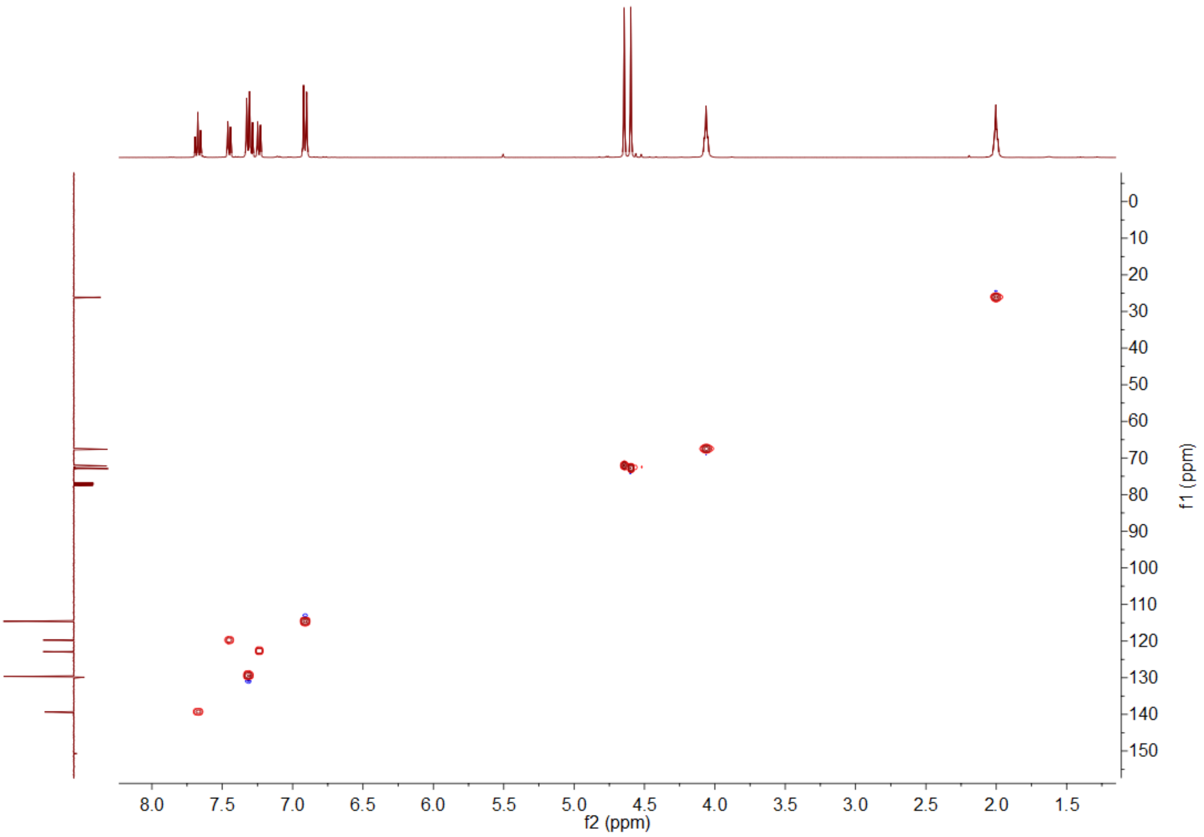

Figure S32 HSQC NMR ($CDCl_3$, 400 MHz) of S4.

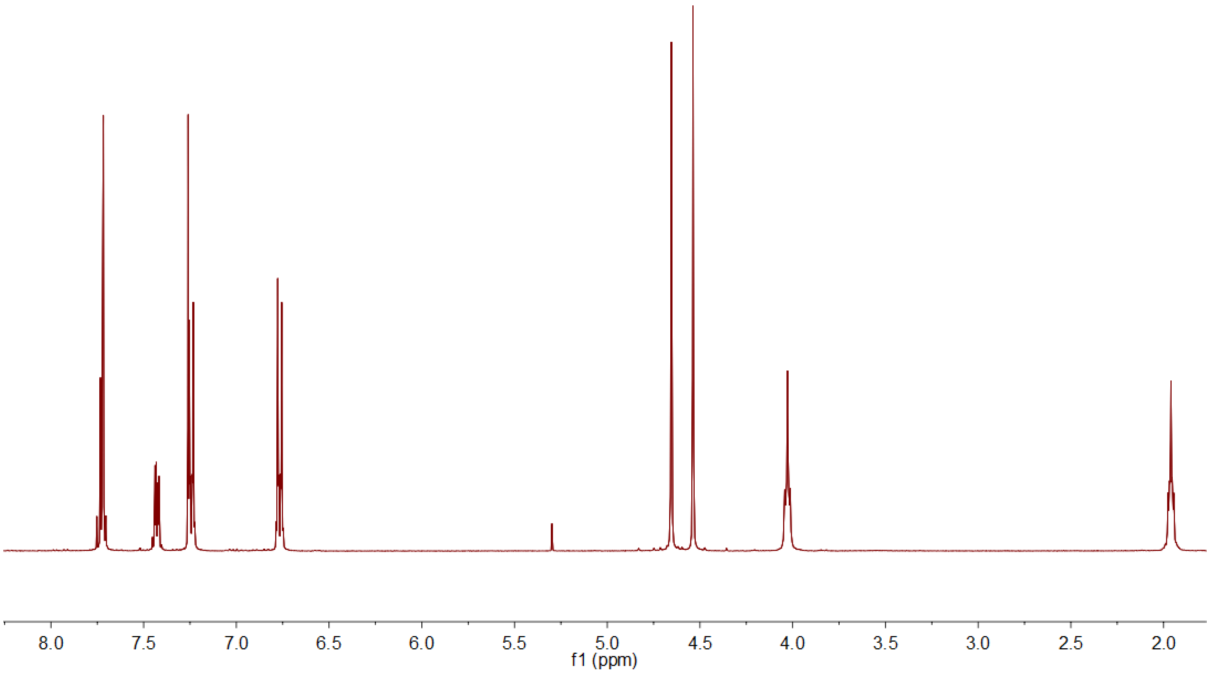

Figure S33 ^{1}H NMR ($CDCl_3$, 400 MHz) of 2b.

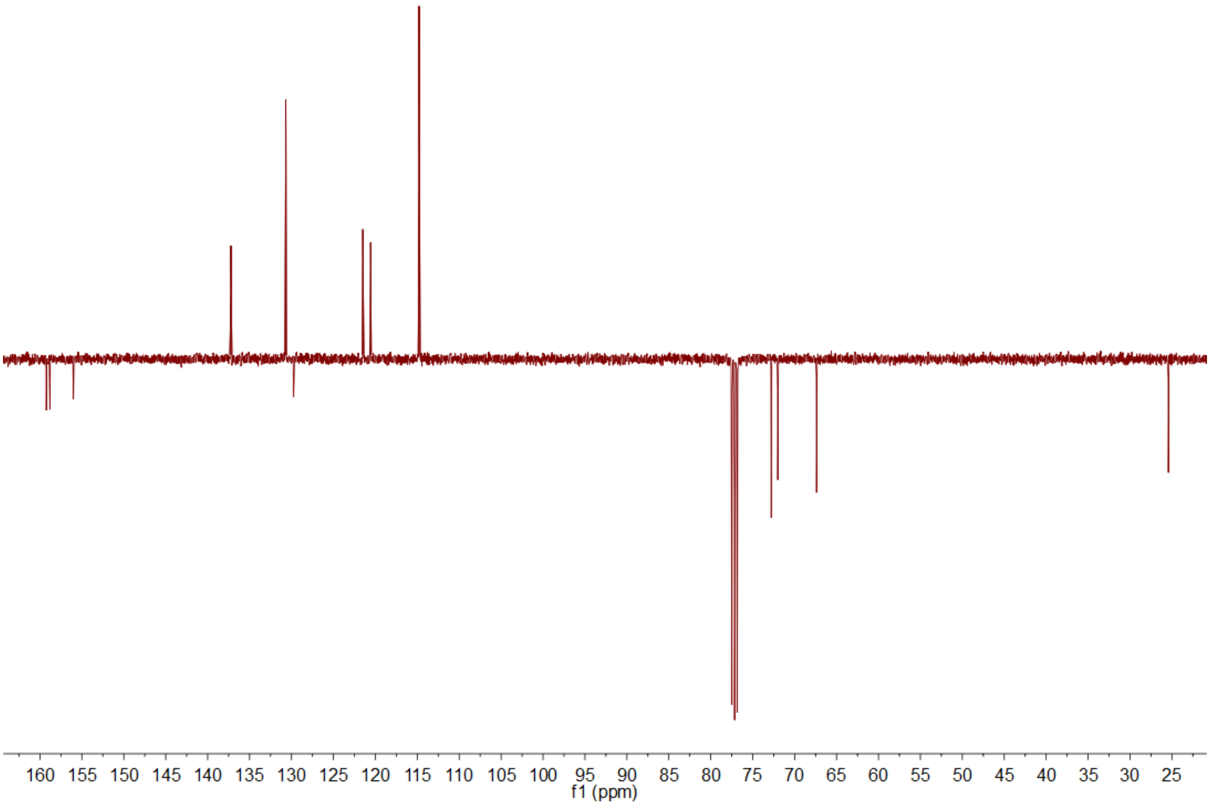

Figure S34 JMOD NMR ($CDCl_3$, 100 MHz) of 2b.

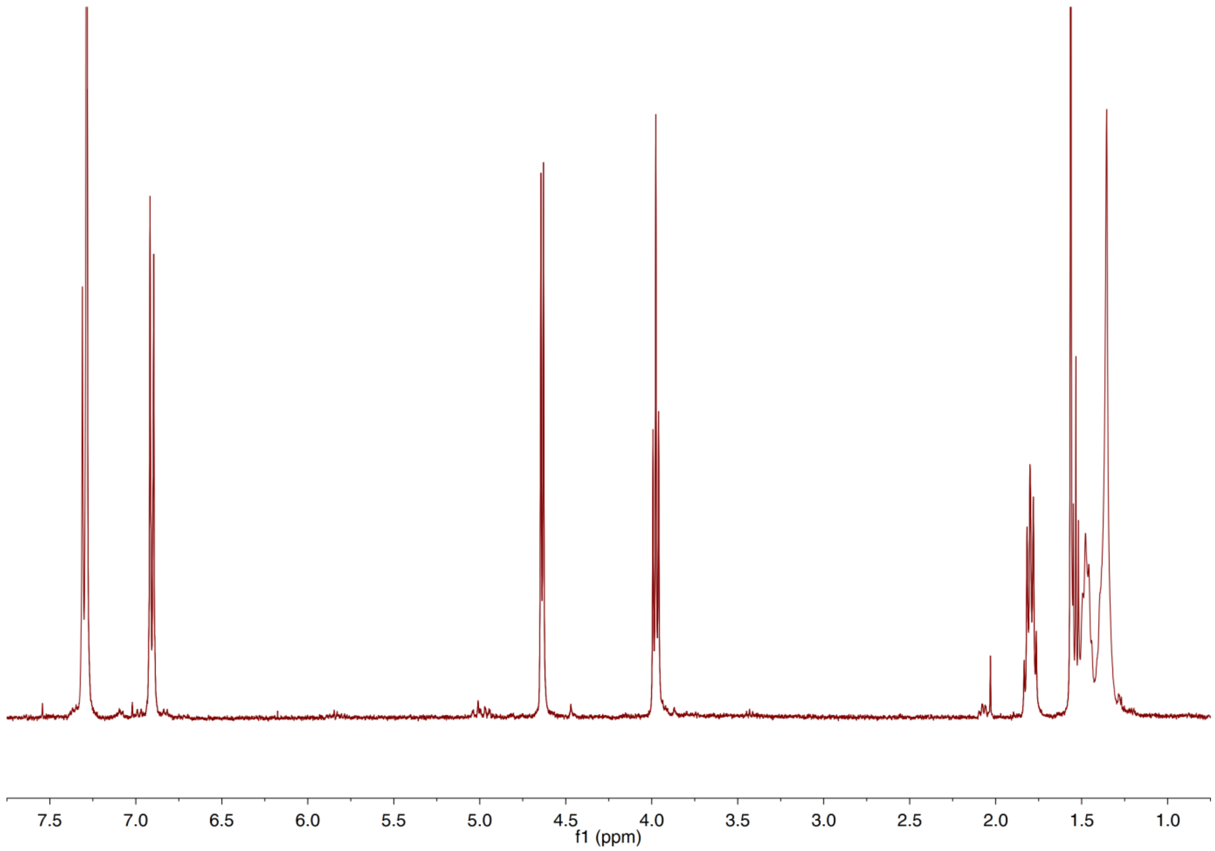

Figure S35 ^{1}H NMR ($CDCl_3$, 400 MHz) of 1,10-di(4-(hydroxymethyl)phenoxy)decane.

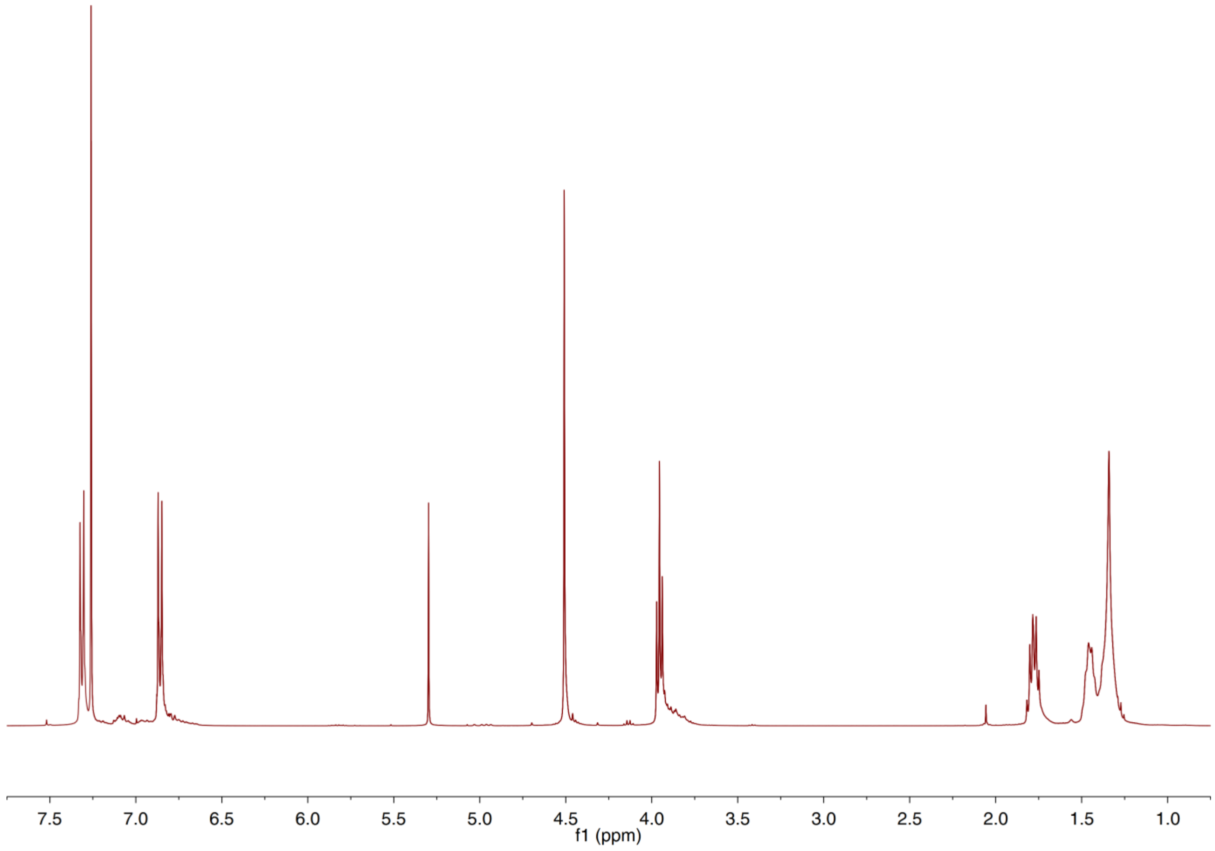

Figure S36 ^{1}H NMR ($CDCl_3$, 400 MHz) of 1,10-di(4-(bromomethyl)phenoxy)decane.

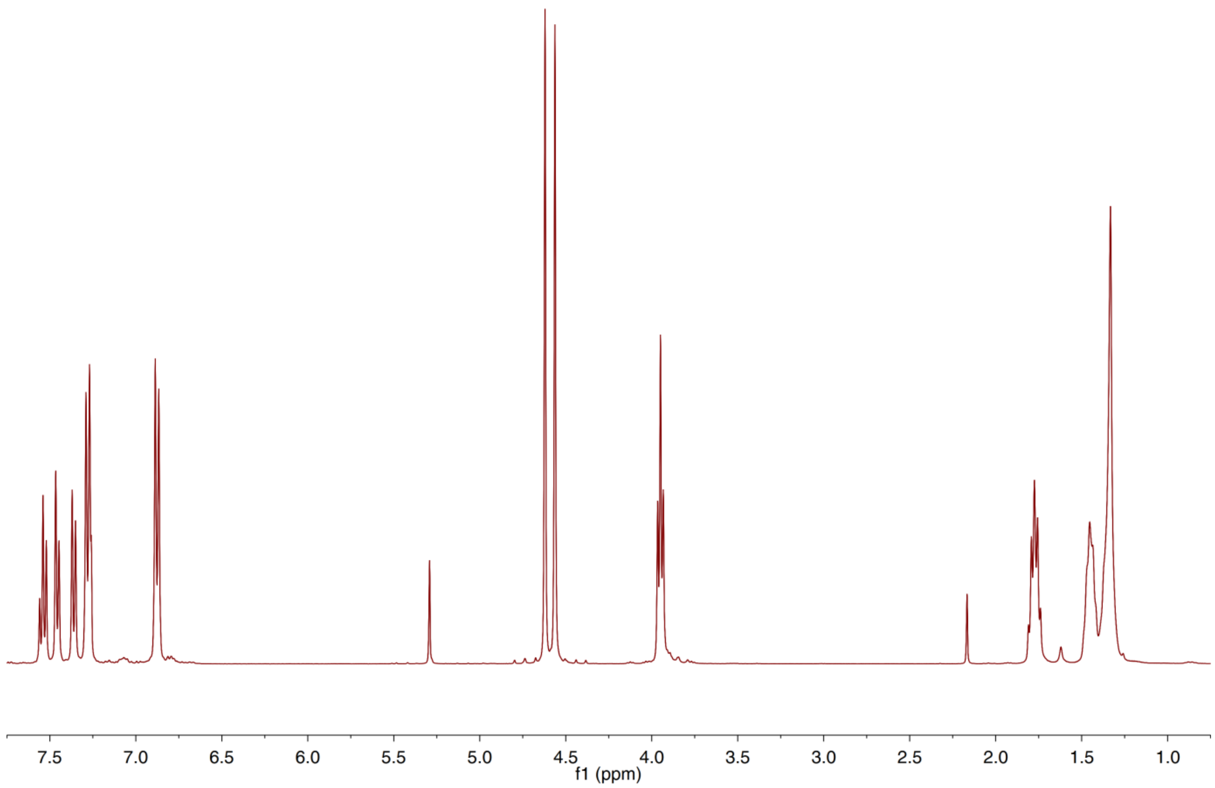

Figure S37 ^{1}H NMR ($CDCl_3$, 400 MHz) of 1c.

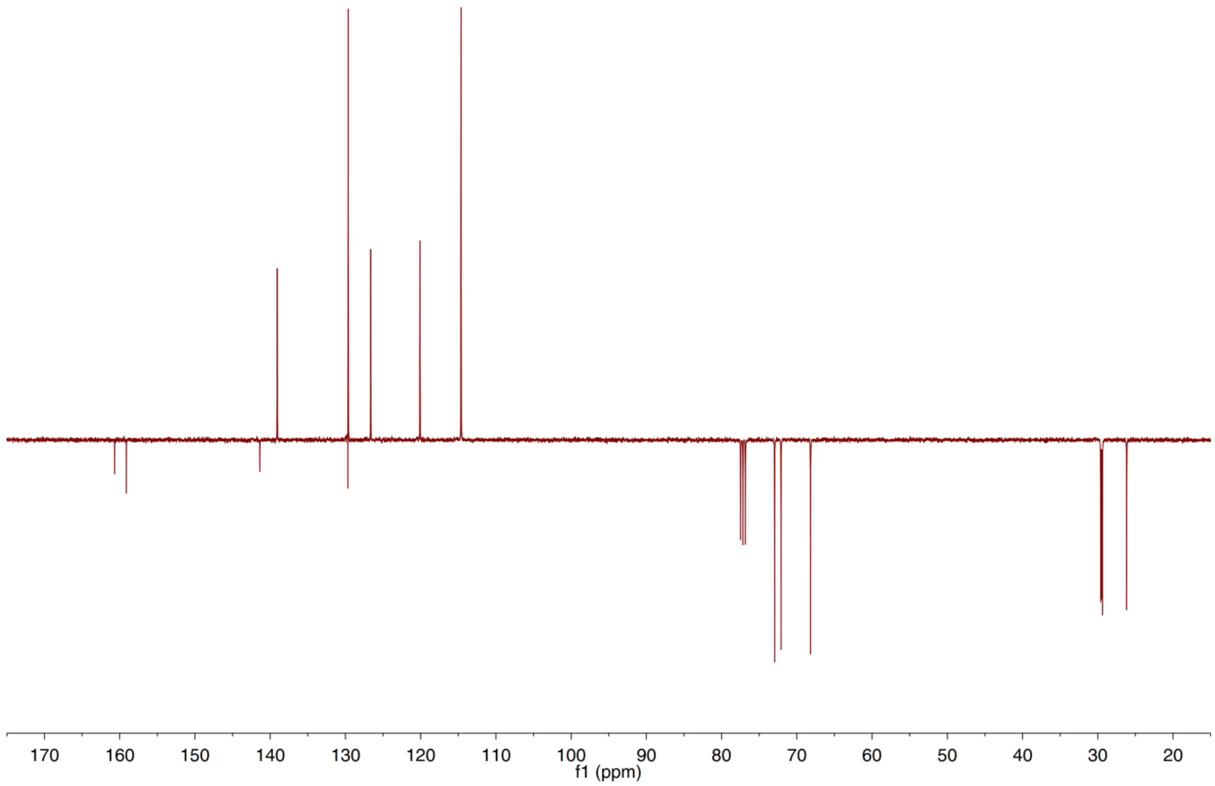

Figure S38 JMOD NMR ($CDCl_3$, 100 MHz) of 1c.

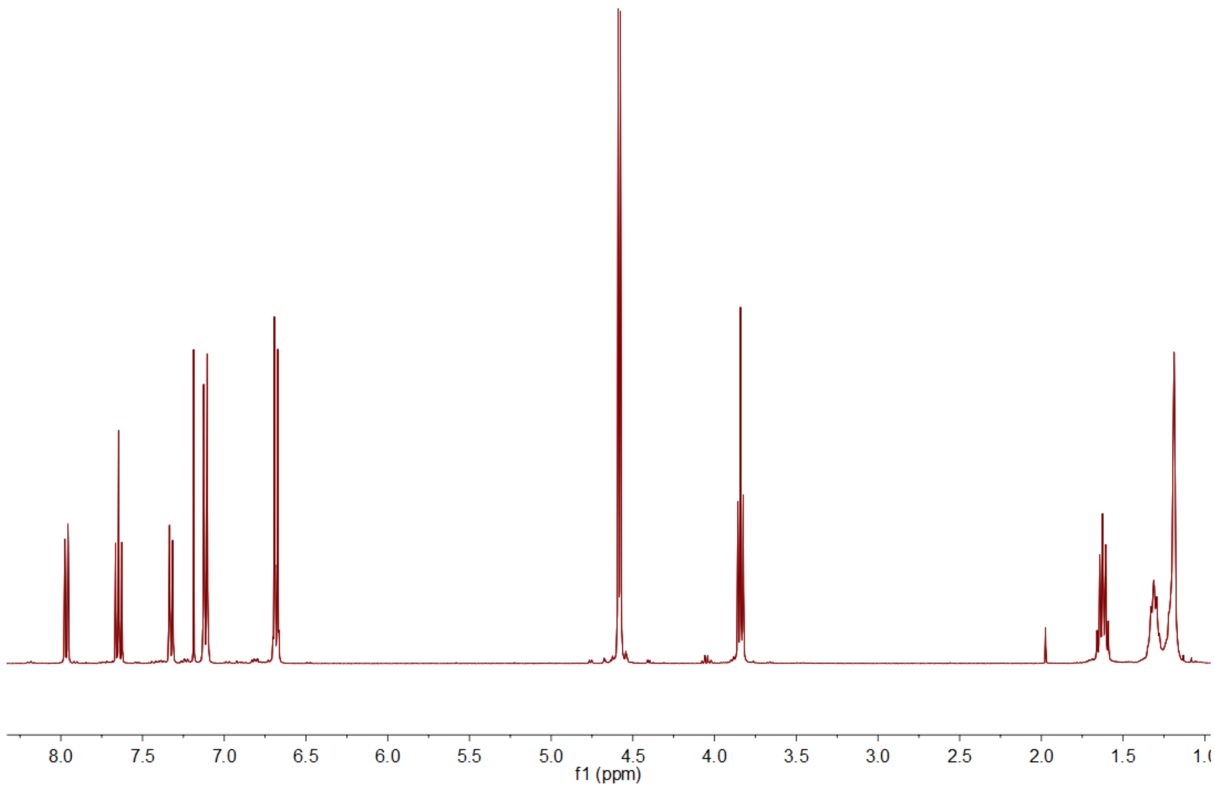

Figure S39 ^{1}H NMR ($CDCl_3$, 400 MHz) of 2c.

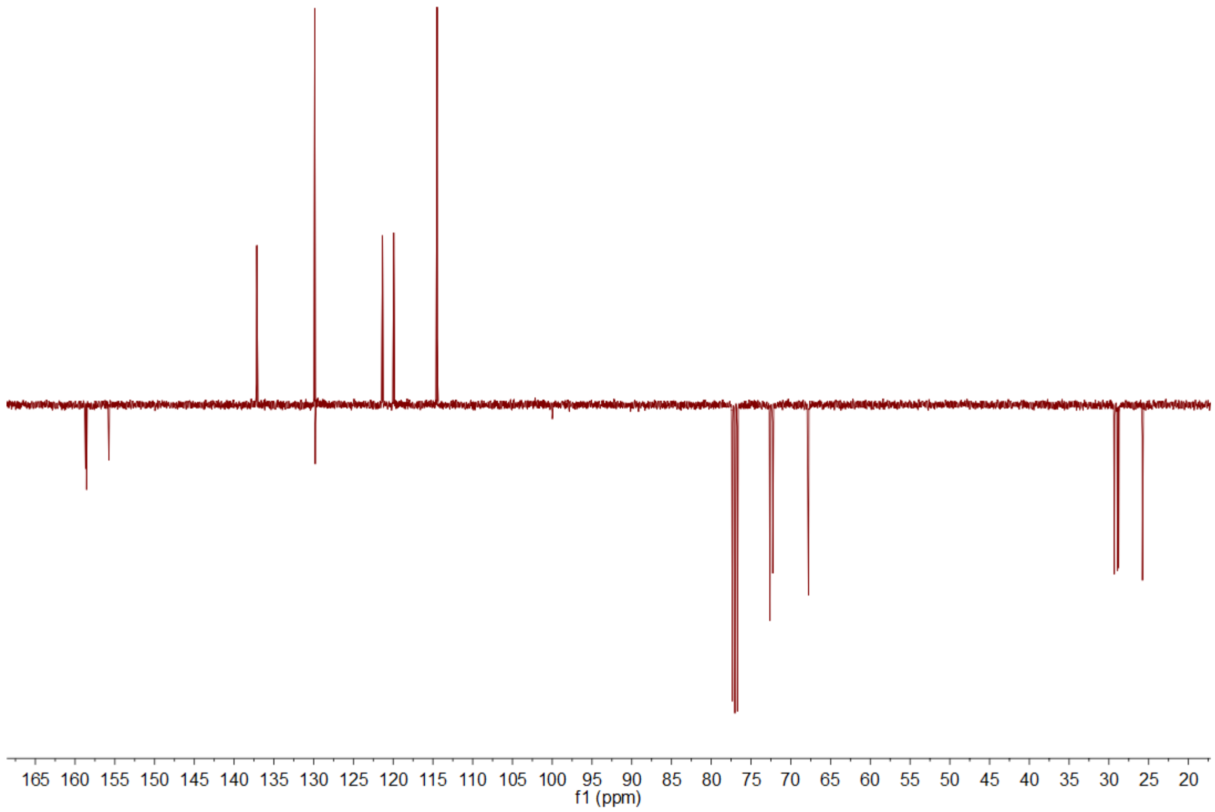

Figure S40 JMOD NMR ($CDCl_3$, 100 MHz) of 2c.

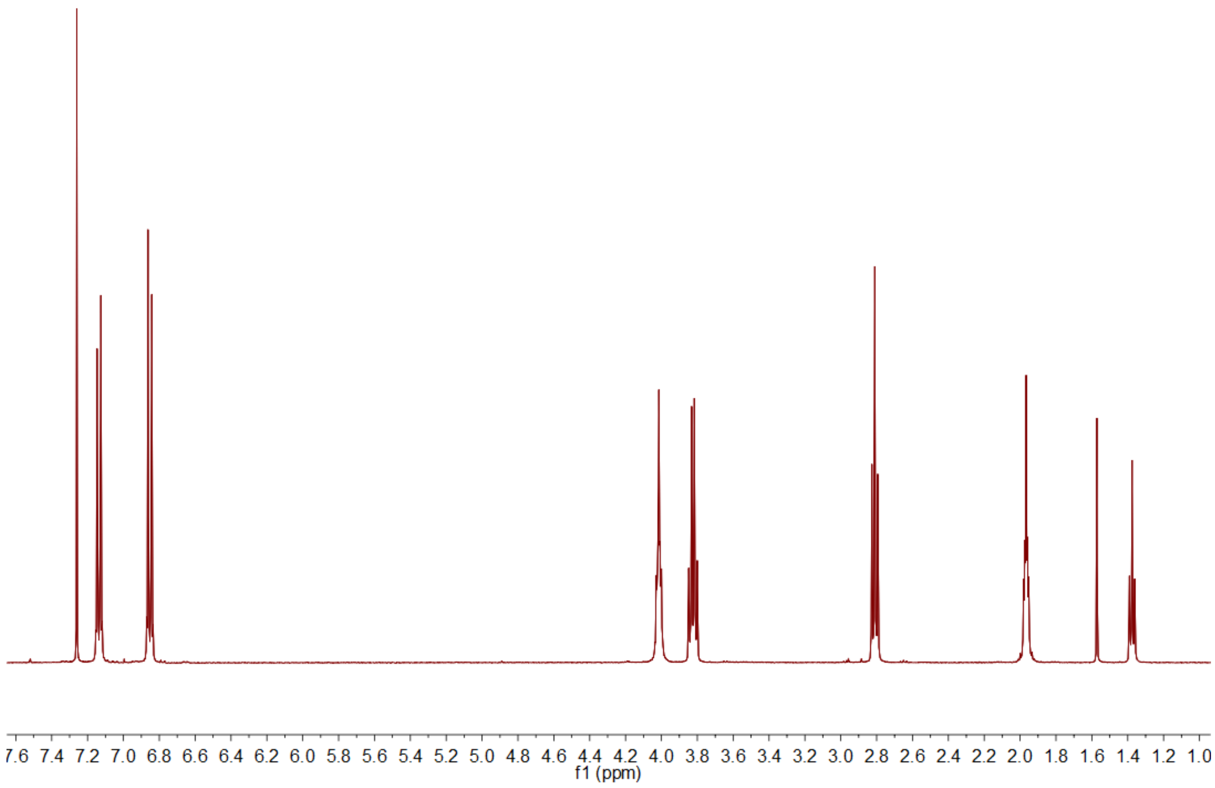

Figure S41 ^{1}H NMR ($CDCl_3$, 400 MHz) of S5.

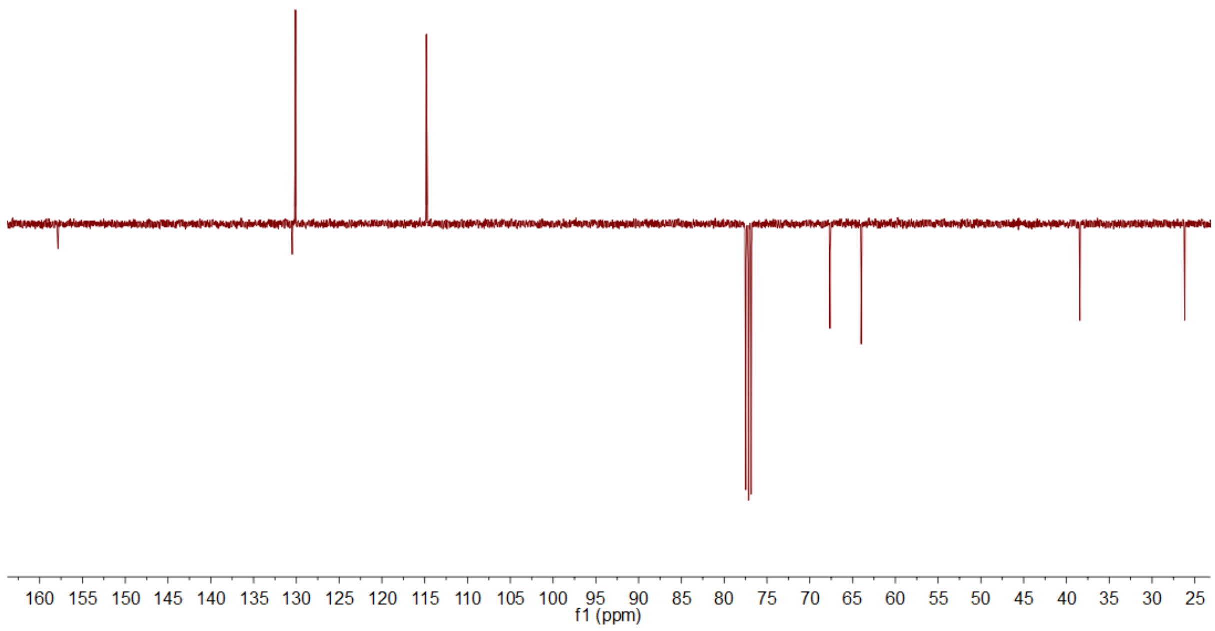

Figure S42 JMOD NMR ($CDCl_3$, 100 MHz) of S5.

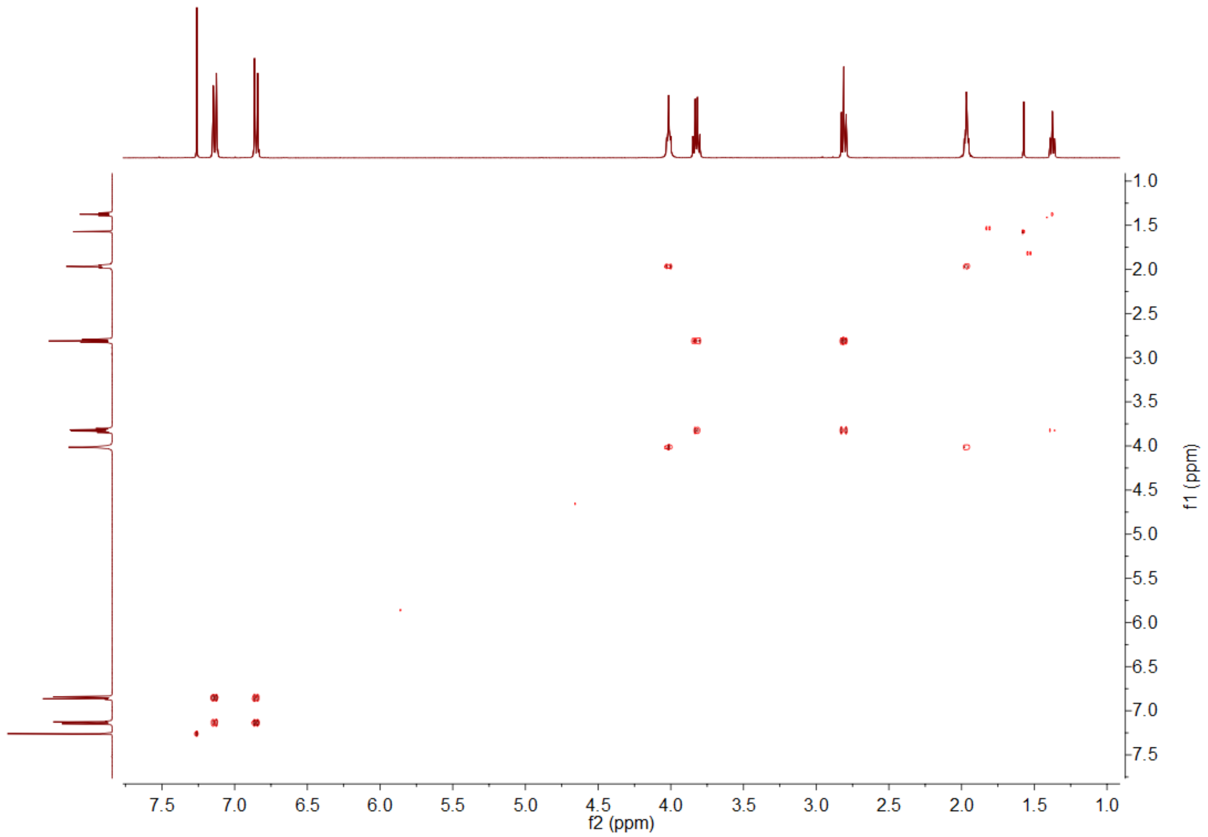

Figure S43 ^{1}H COSY NMR ($CDCl_3$, 400 MHz) of S5.

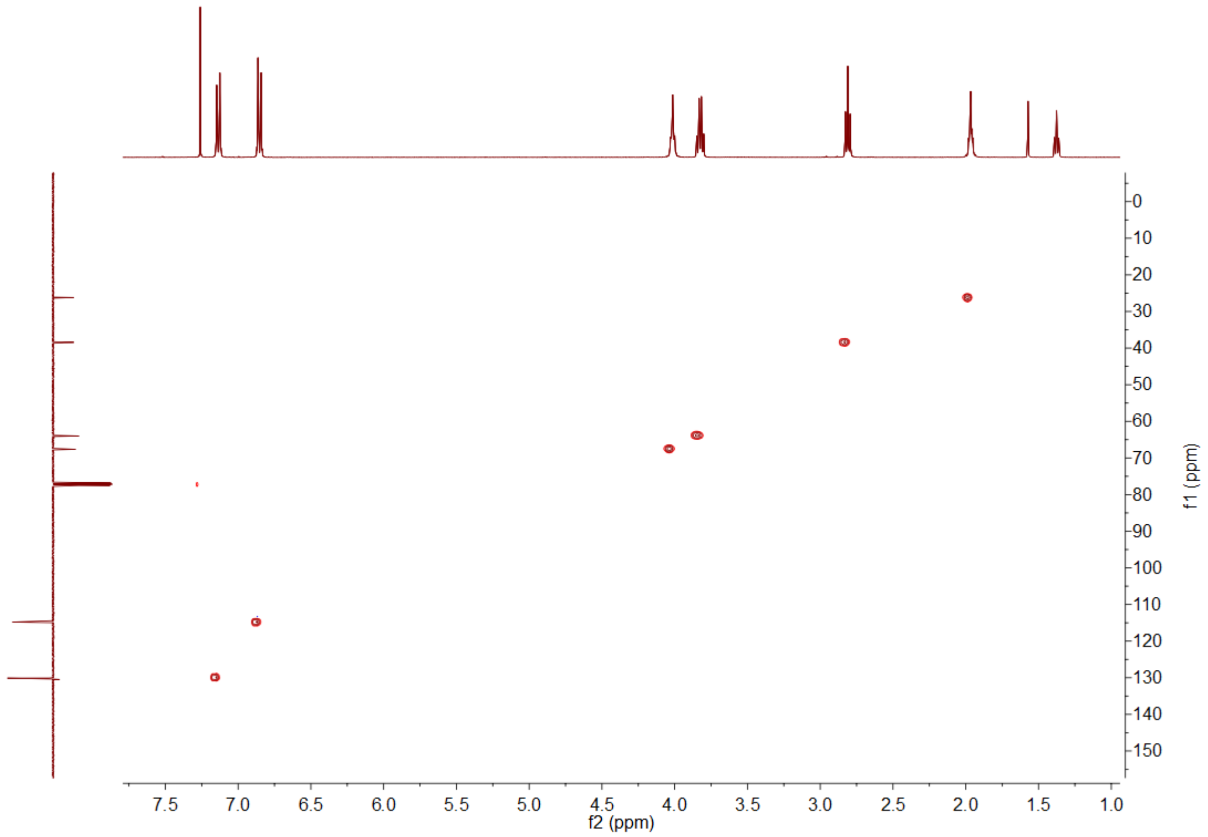

Figure S44 HSQC NMR ($CDCl_3$, 400 MHz) of S5.

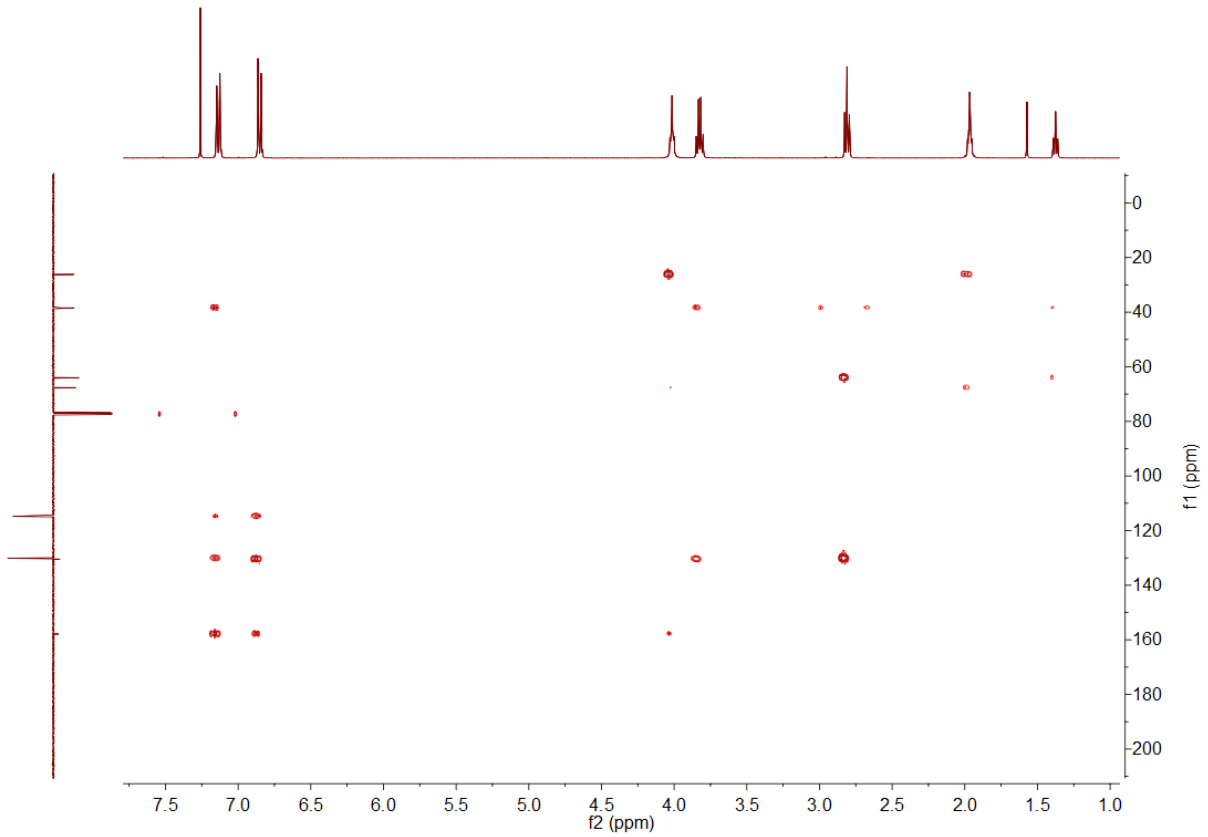

Figure S45 HMBC NMR ($CDCl_3$, 400 MHz) of S5.

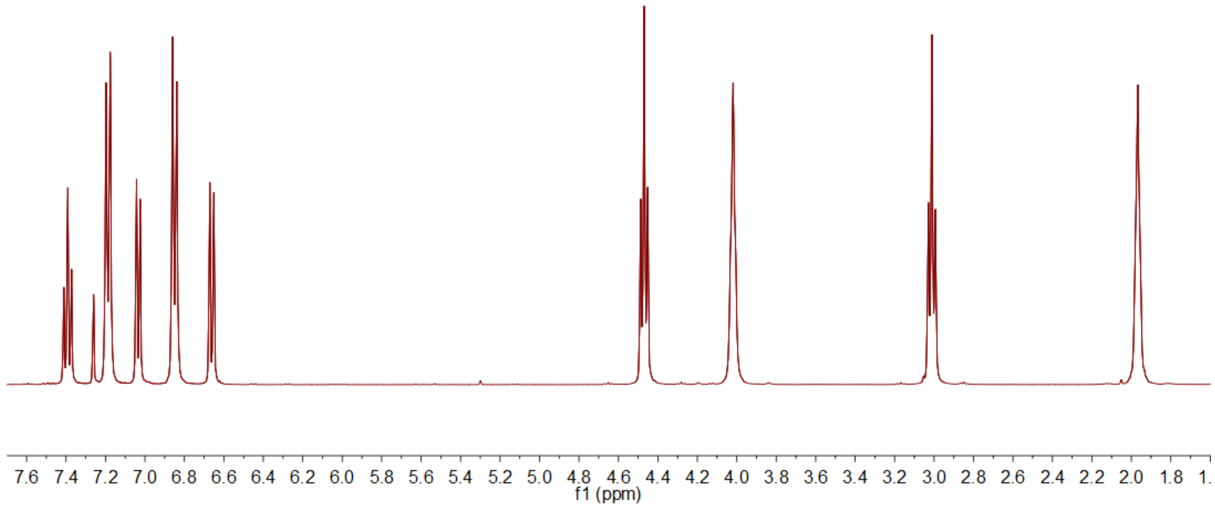

Figure S46 ^{1}H NMR ($CDCl_3$, 400 MHz) of 1d.

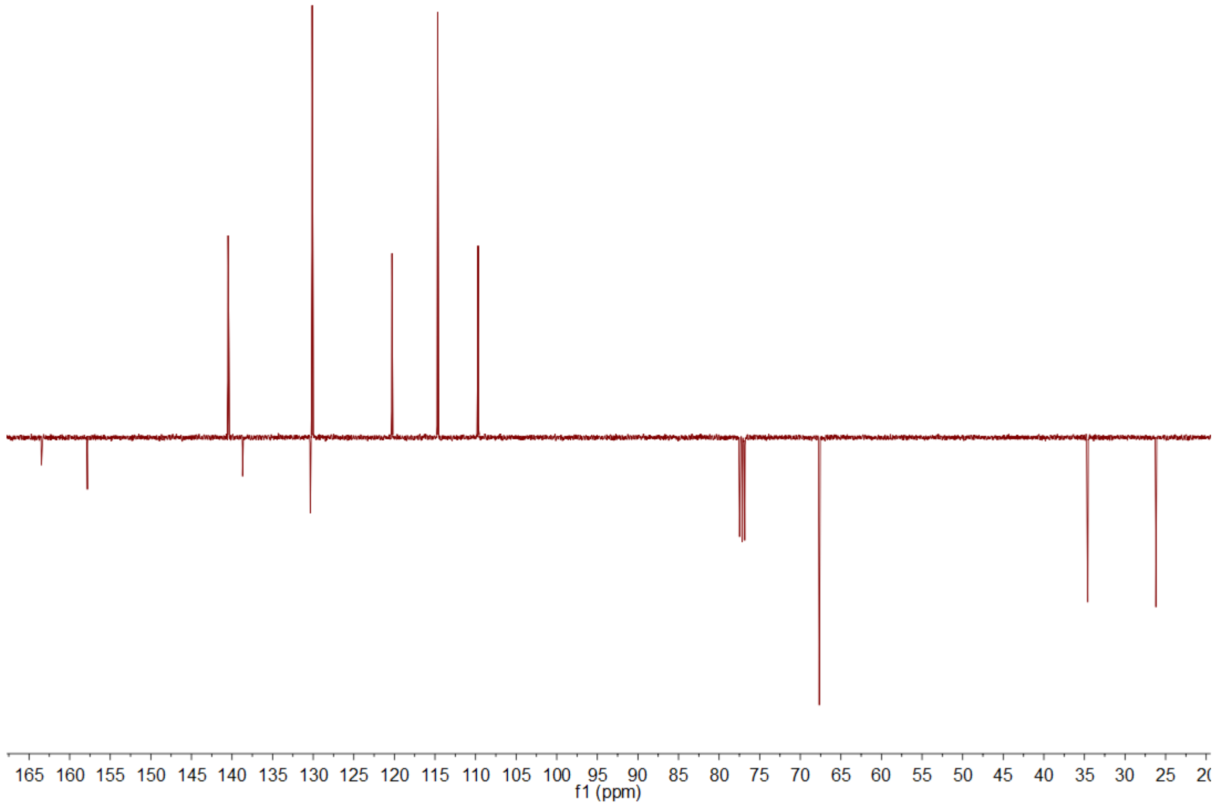

Figure S47 JMOD NMR ($CDCl_3$, 100 MHz) of 1d.

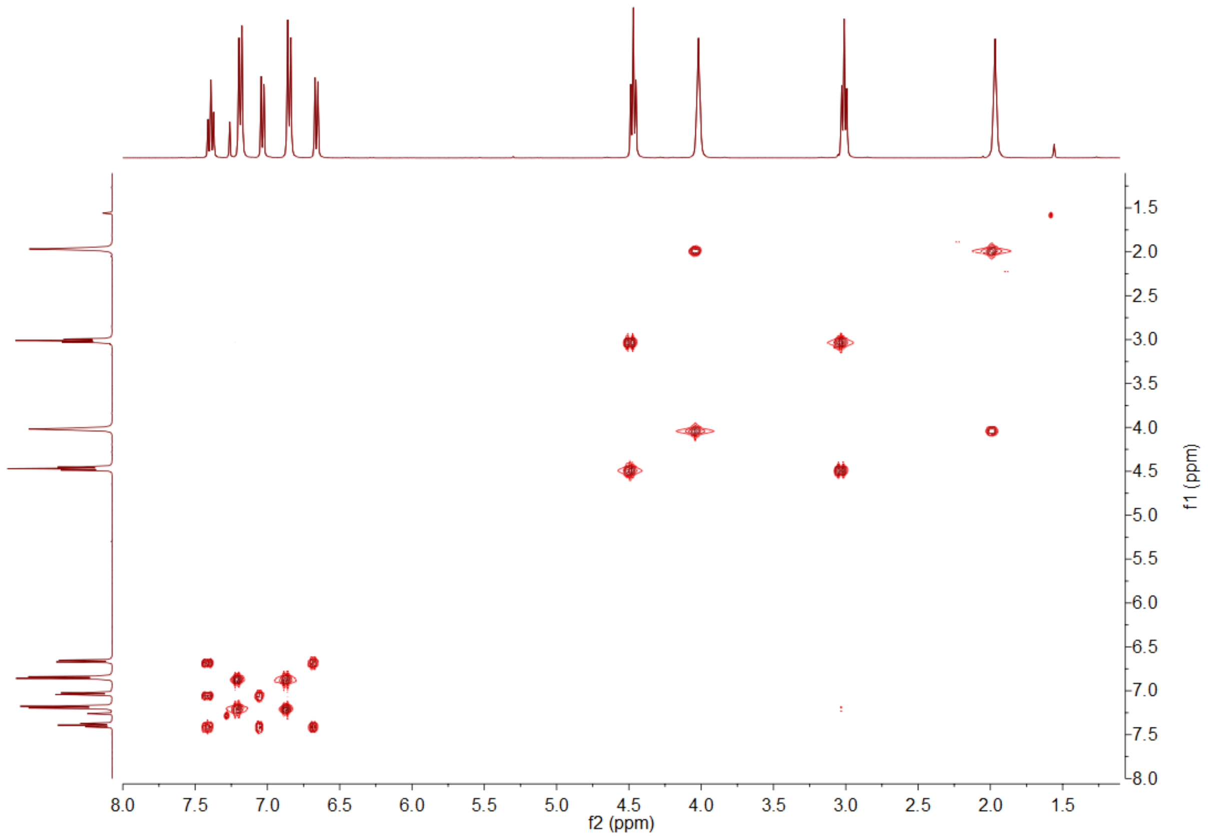

Figure S48 ¹H COSY NMR ($CDCl_3$, 400 MHz) of 1d.

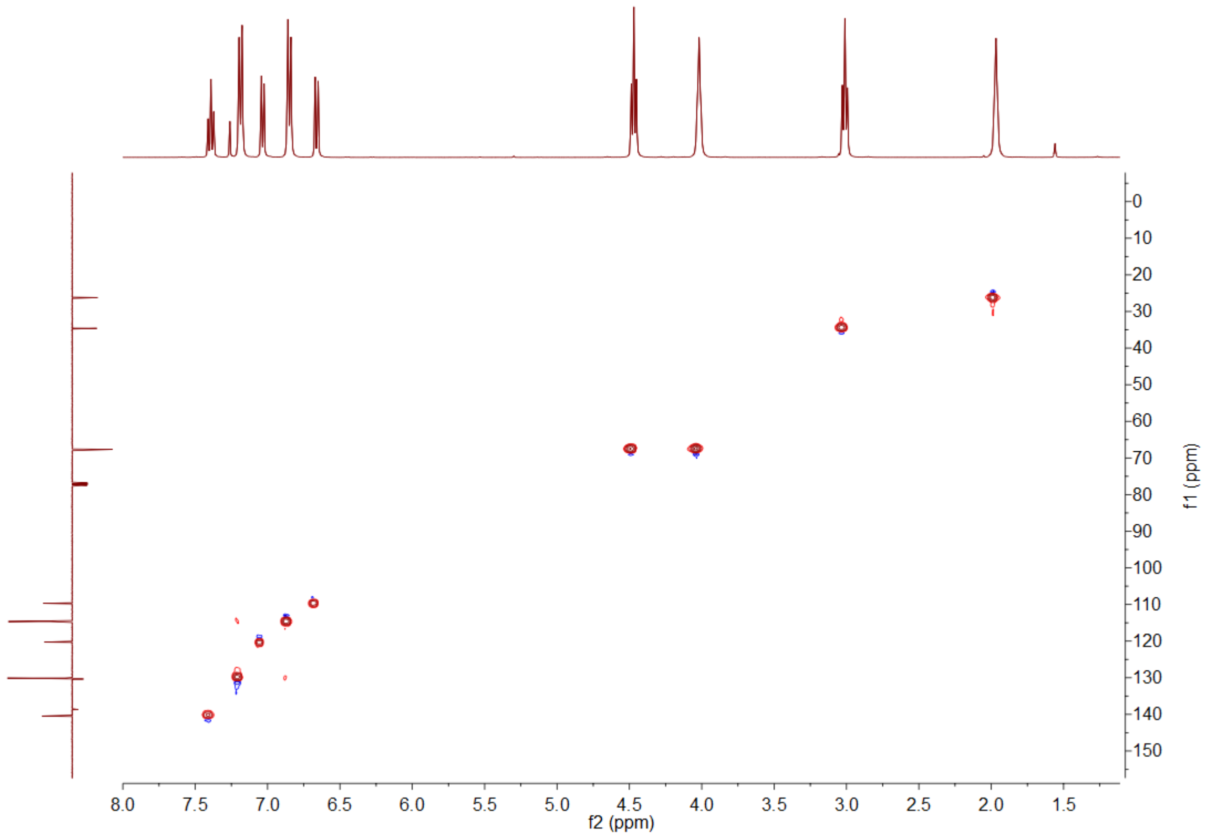

Figure S49 HSQC NMR ($CDCl_3$, 400 MHz) of 1d.

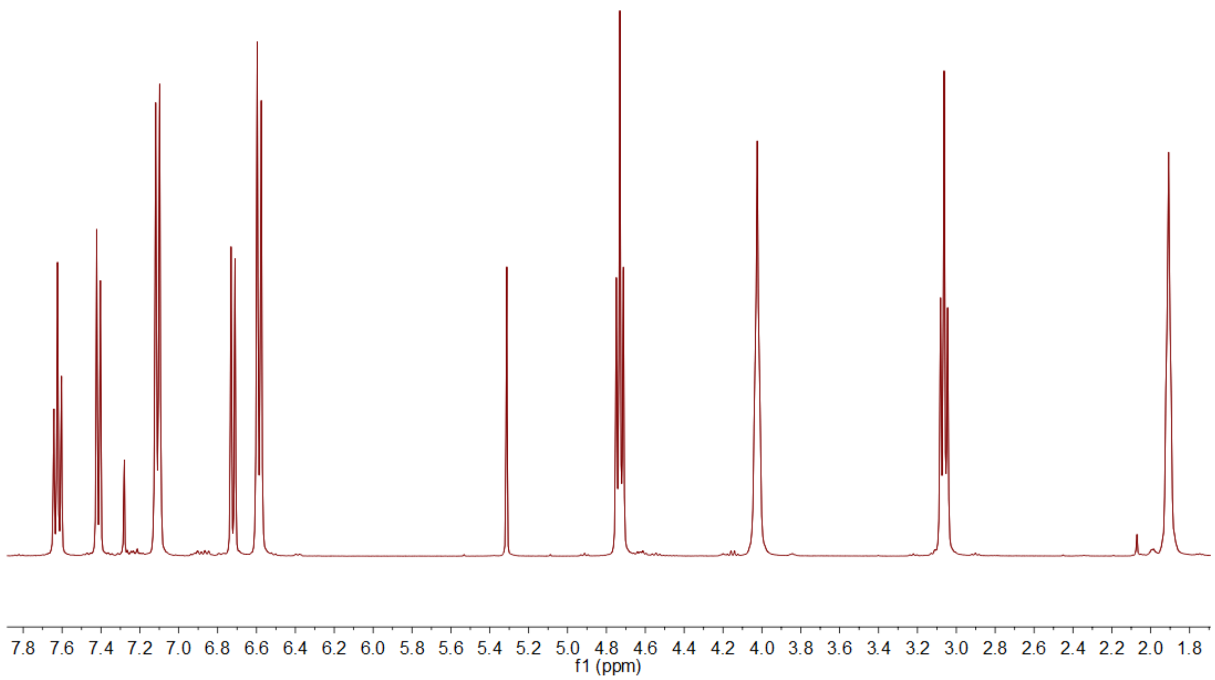

Figure S50 ^{1}H NMR ($CDCl_3$, 400 MHz) of 2d.

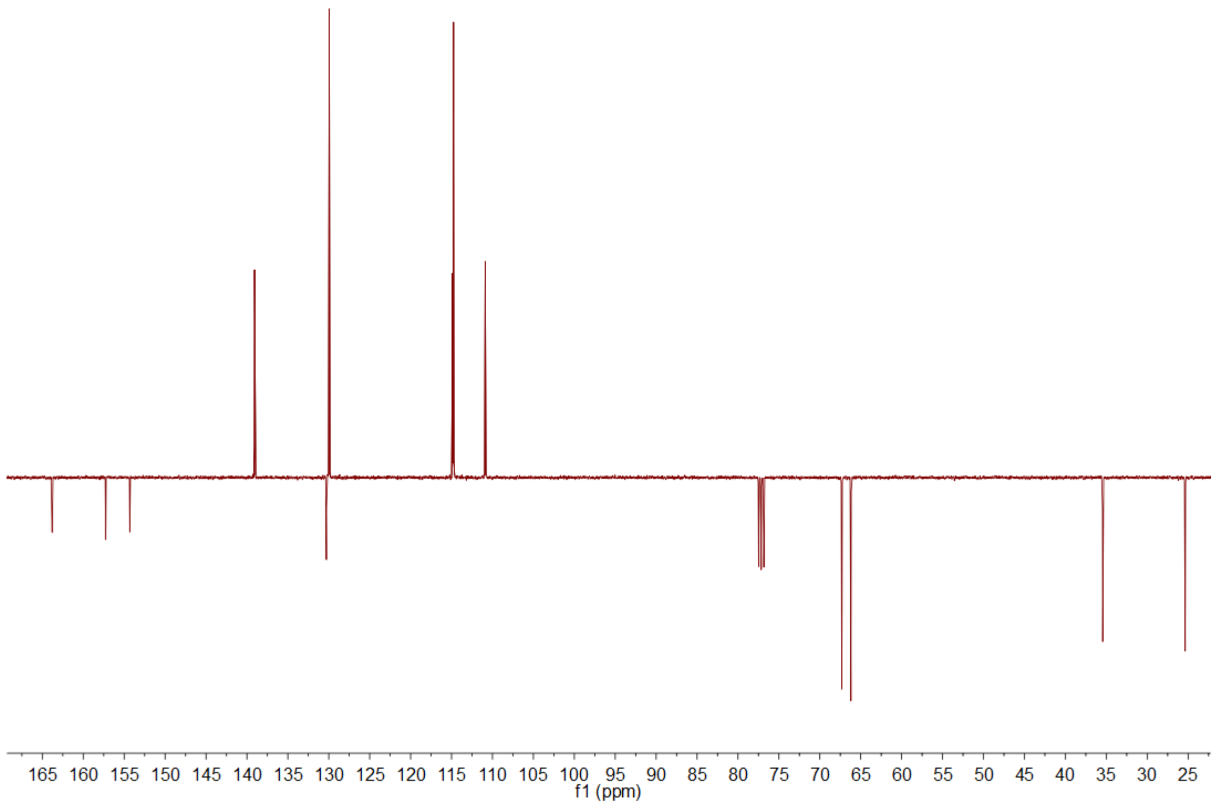

Figure S51 JMOD NMR ($CDCl_3$, 100 MHz) of 2d.

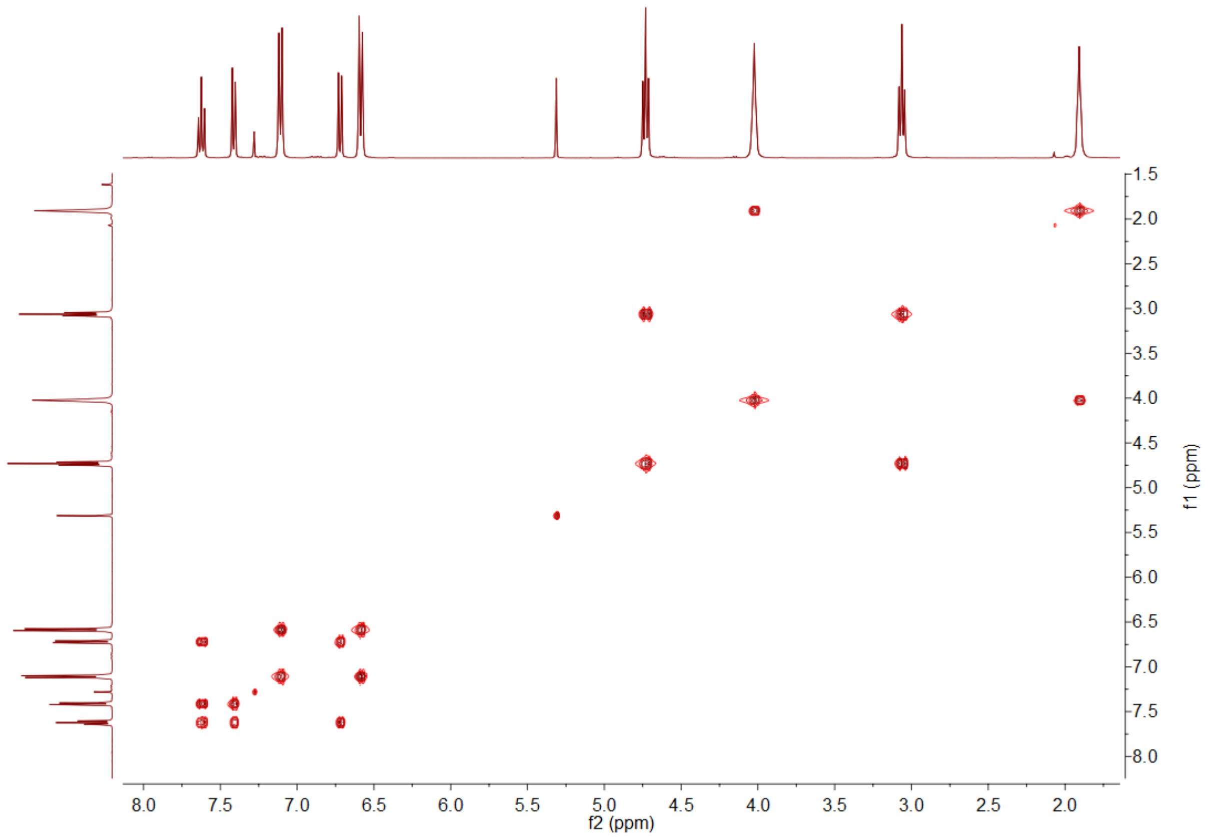

Figure S52 ^{1}H COSY NMR ($CDCl_3$, 400 MHz) of 2d.

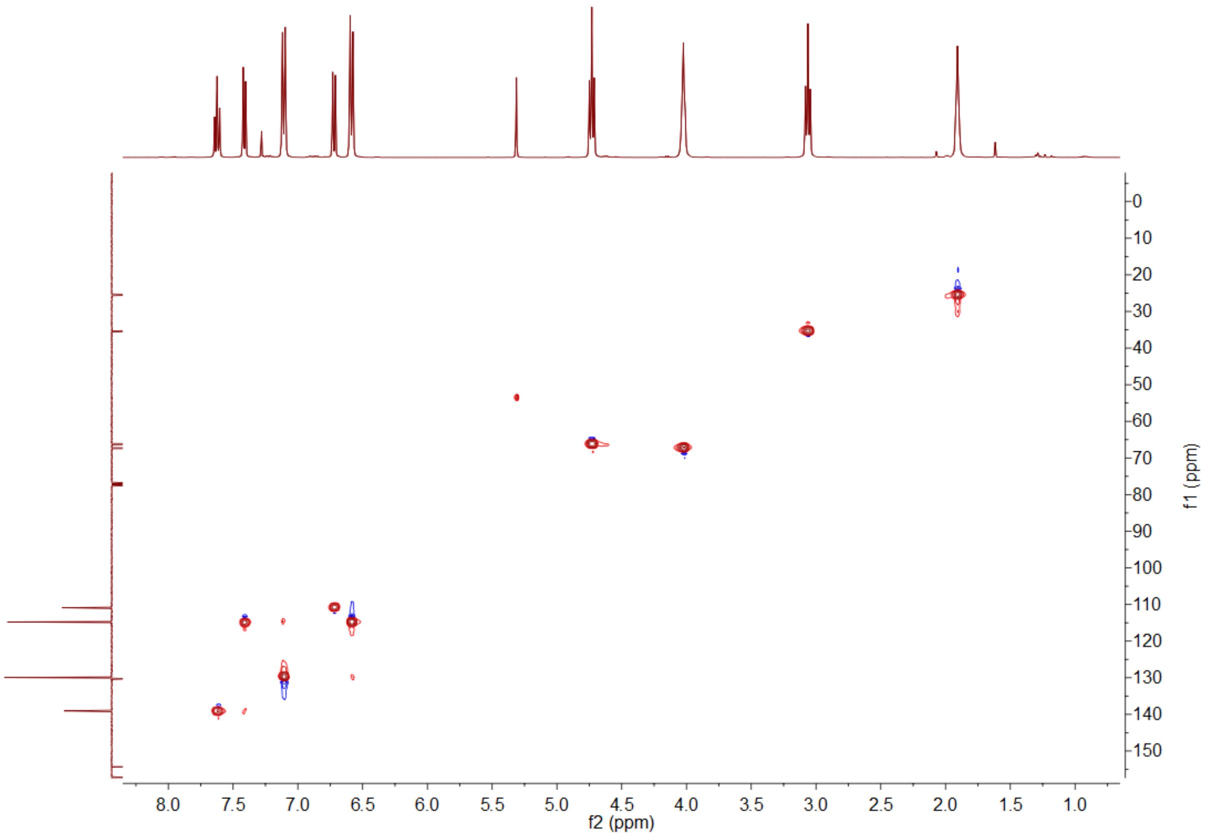

Figure S53 HSQC NMR ($CDCl_3$, 400 MHz) of 2d.

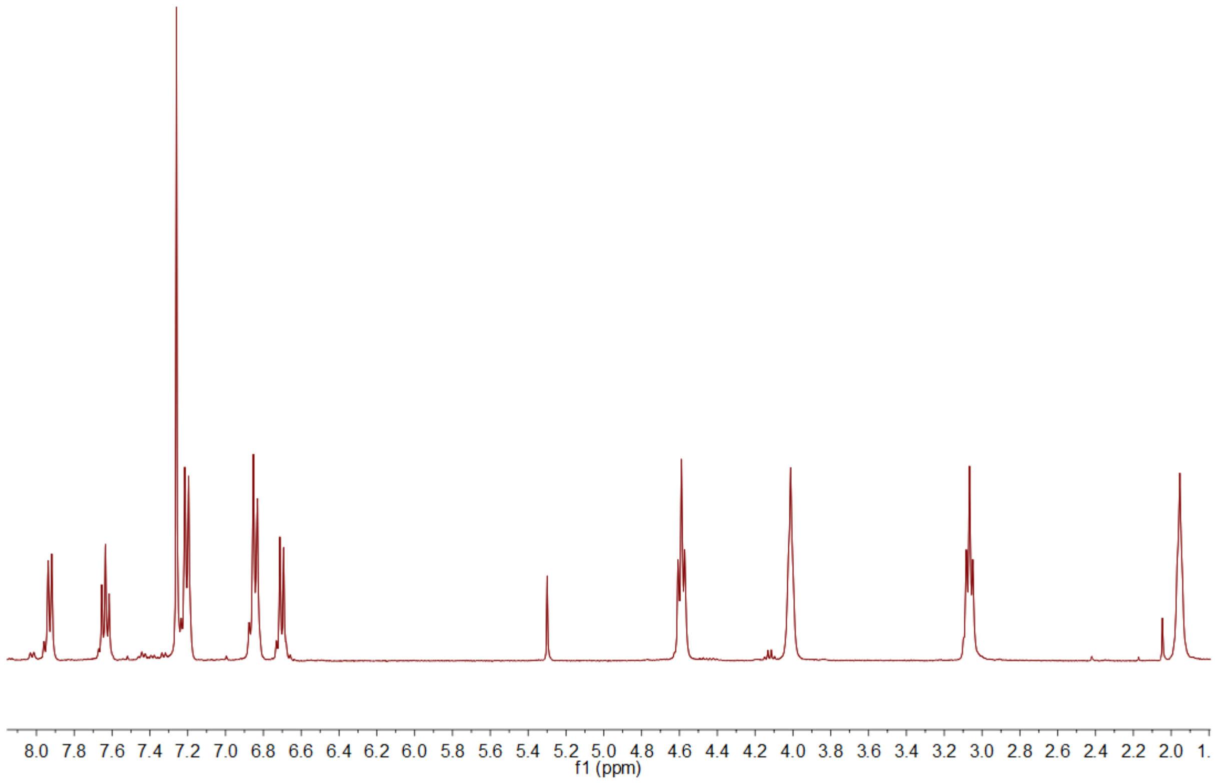

Figure S54 ^{1}H NMR ($CDCl_3$, 400 MHz) of 2d Dimer.

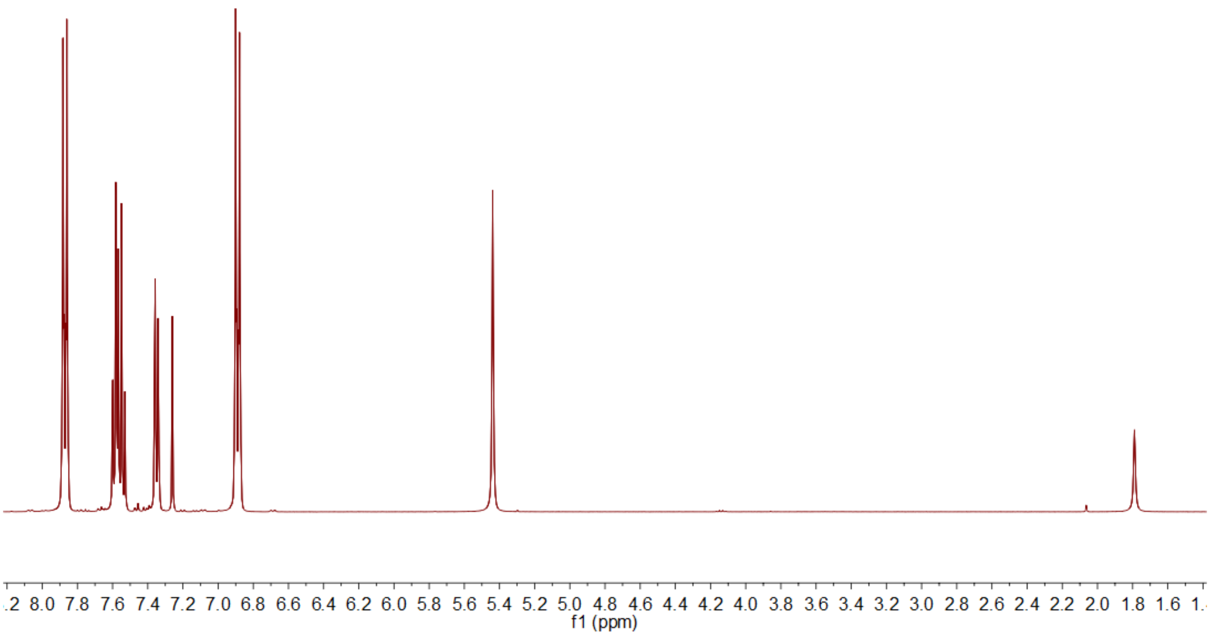

Figure S55 ^{1}H NMR ($CDCl_3$, 400 MHz) of S6.

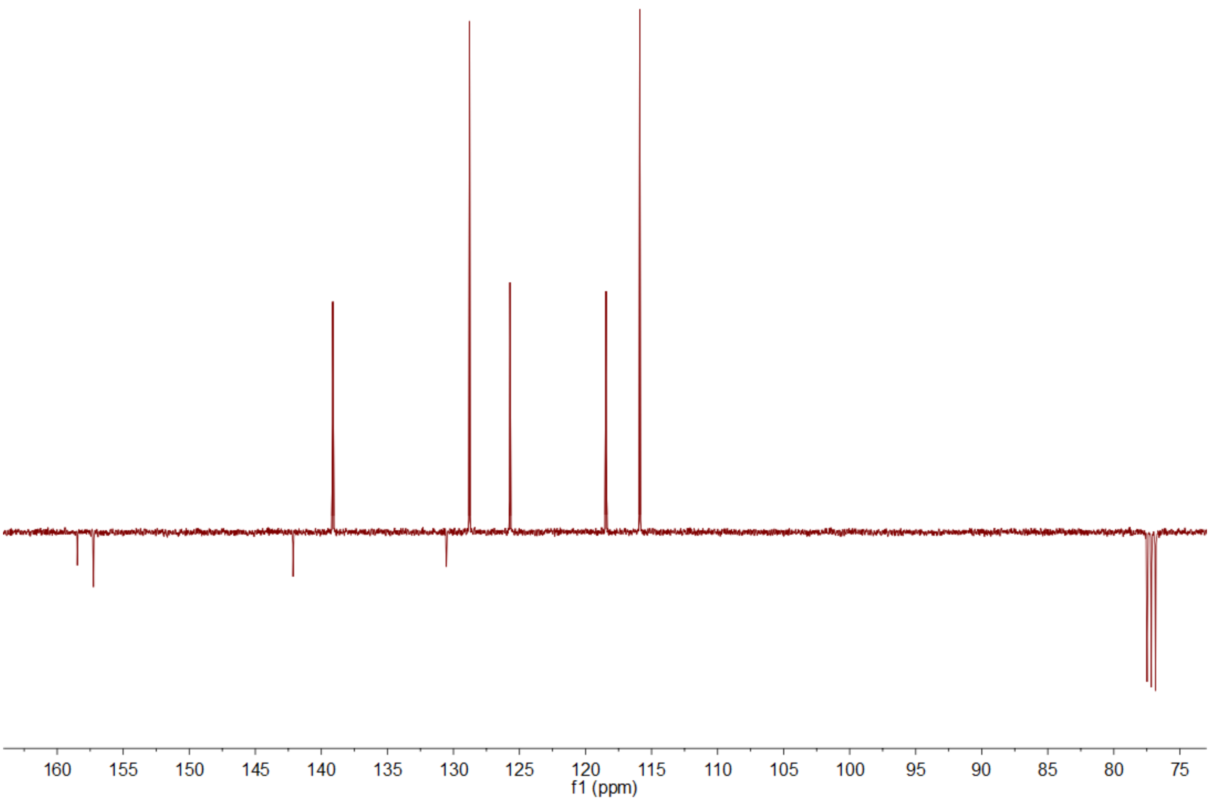

Figure S56 JMOD NMR ($CDCl_3$, 100 MHz) of S6.

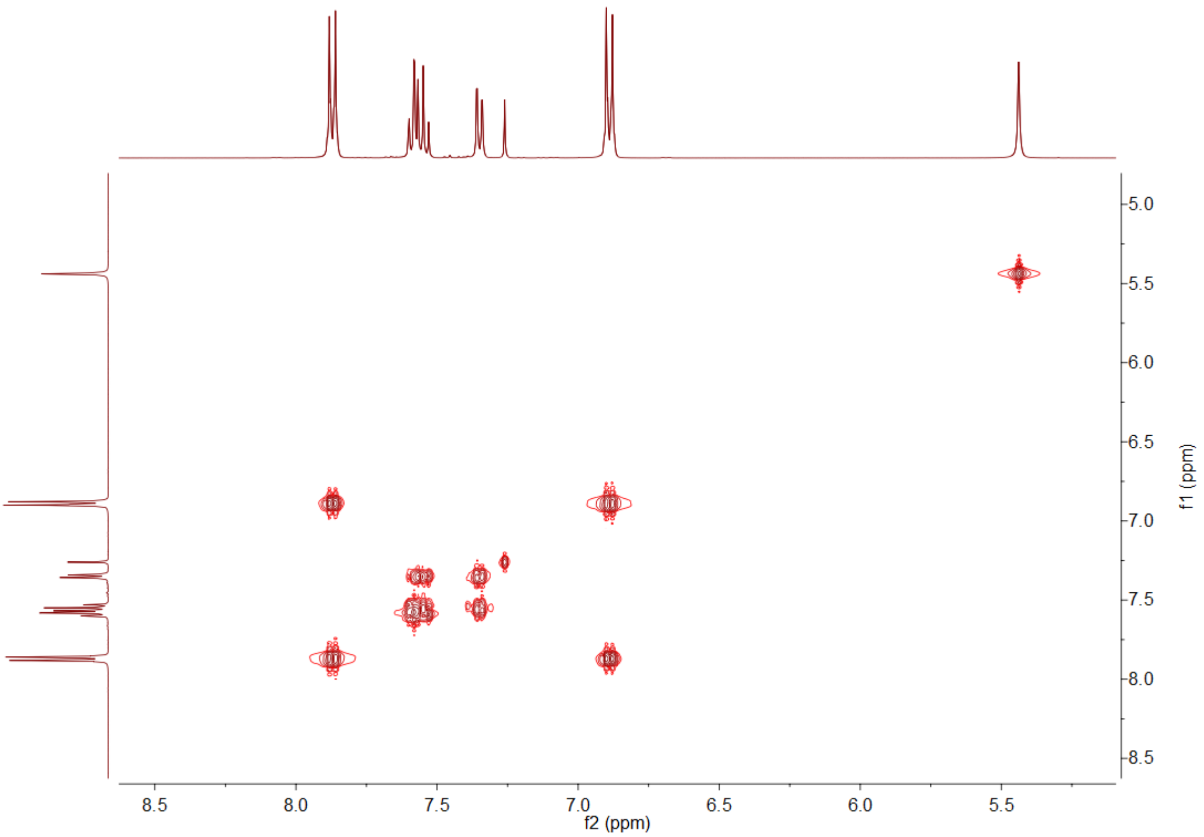

Figure S57 ^{1}H COSY NMR ($CDCl_3$, 400 MHz) of S6.

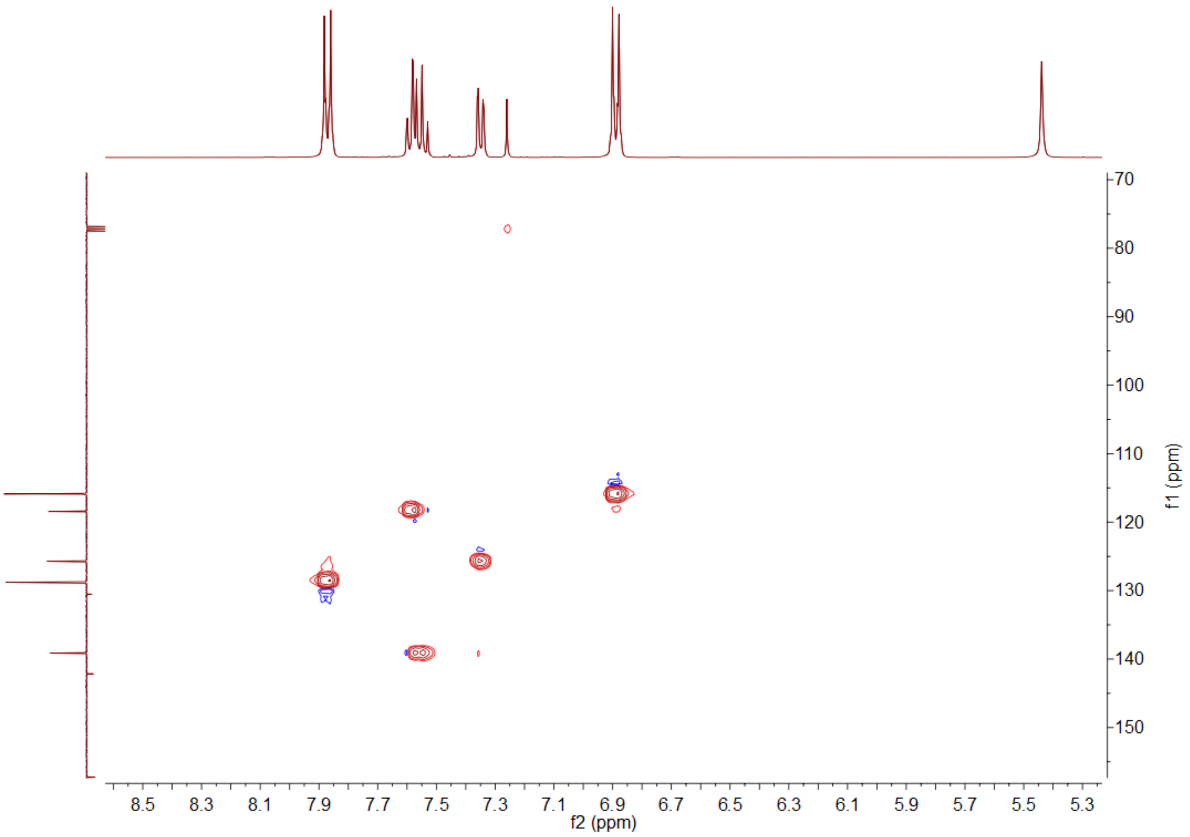

Figure S58 HSQC NMR ($CDCl_3$, 400 MHz) of S6.

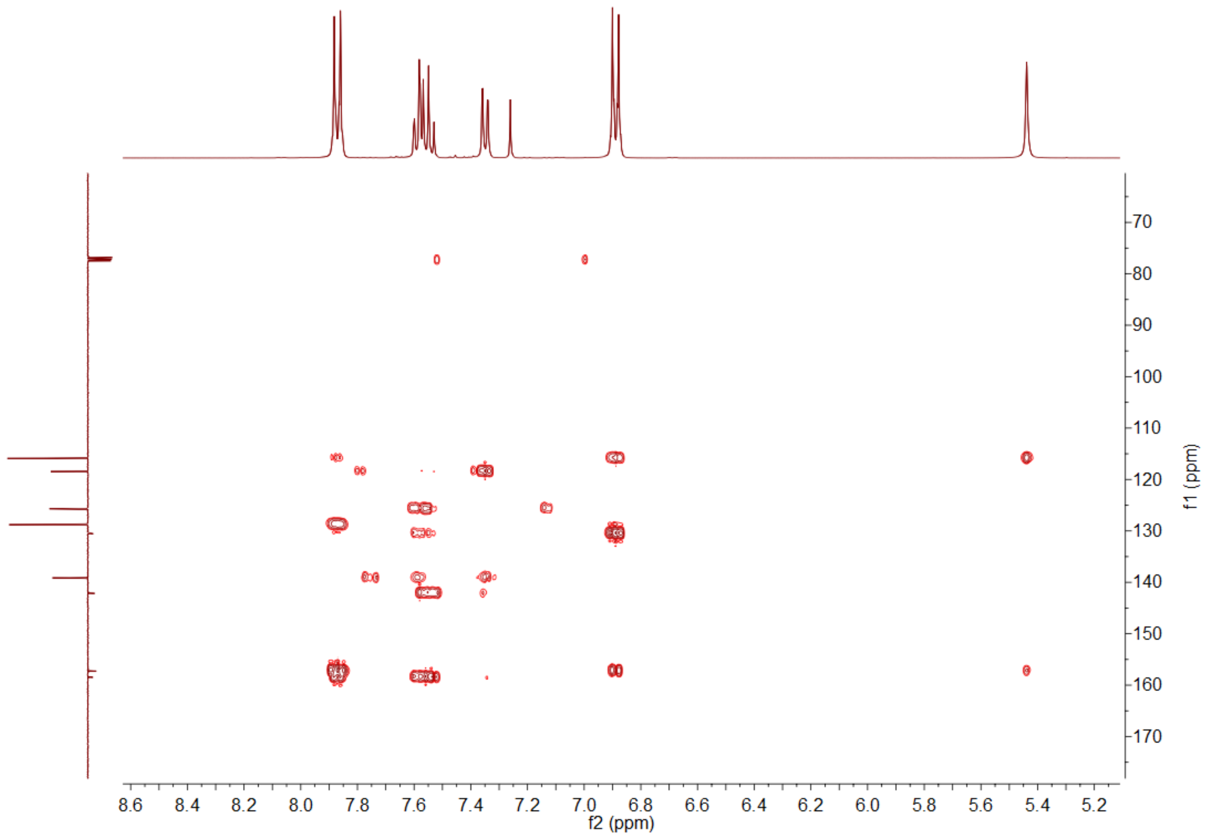

Figure S59 HMBC NMR ($CDCl_3$, 400 MHz) of S6.

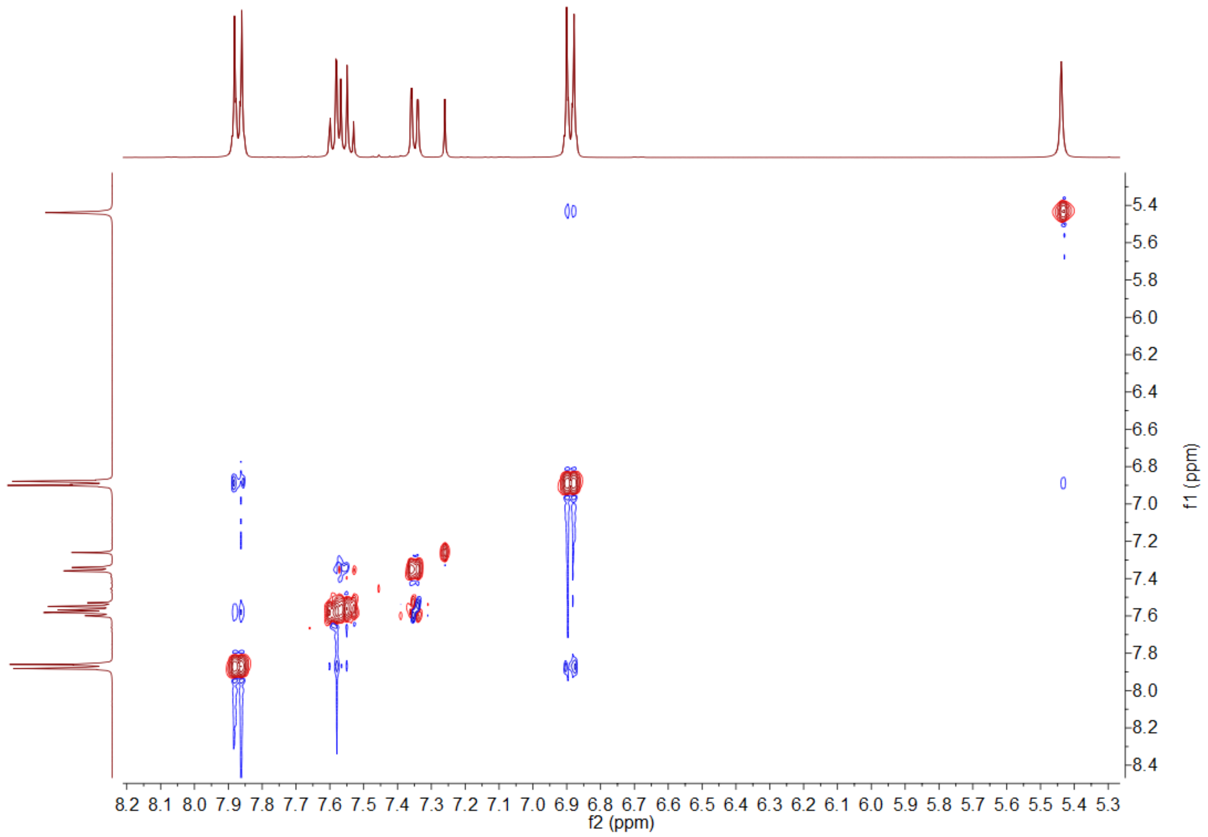

Figure S60 ^{1}H NOESY NMR ($CDCl_3$, 400 MHz) of S6.

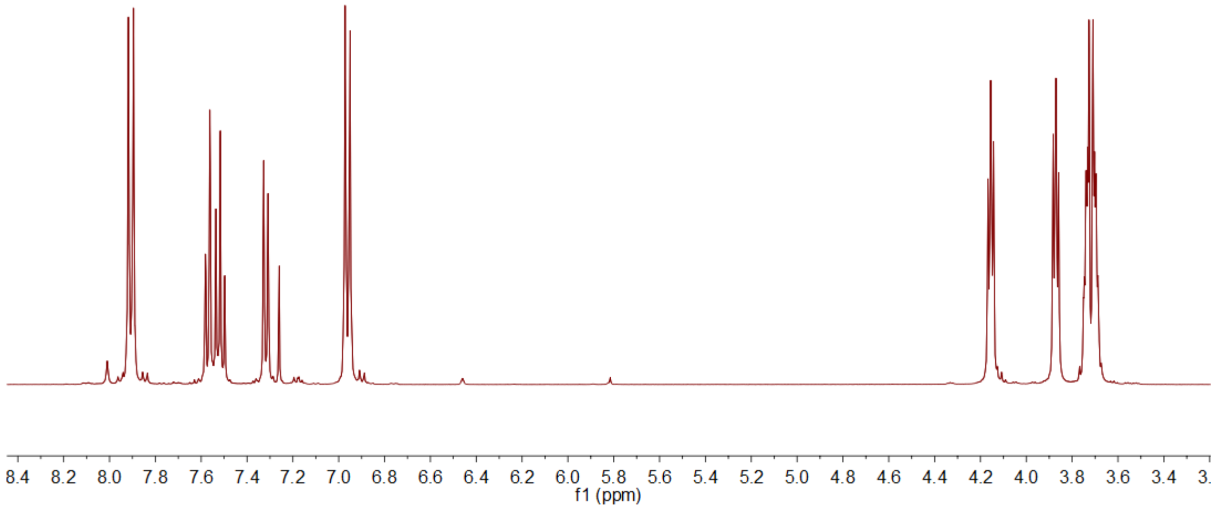

Figure S61 ^{1}H NMR ($CDCl_3$, 400 MHz) of 1e.

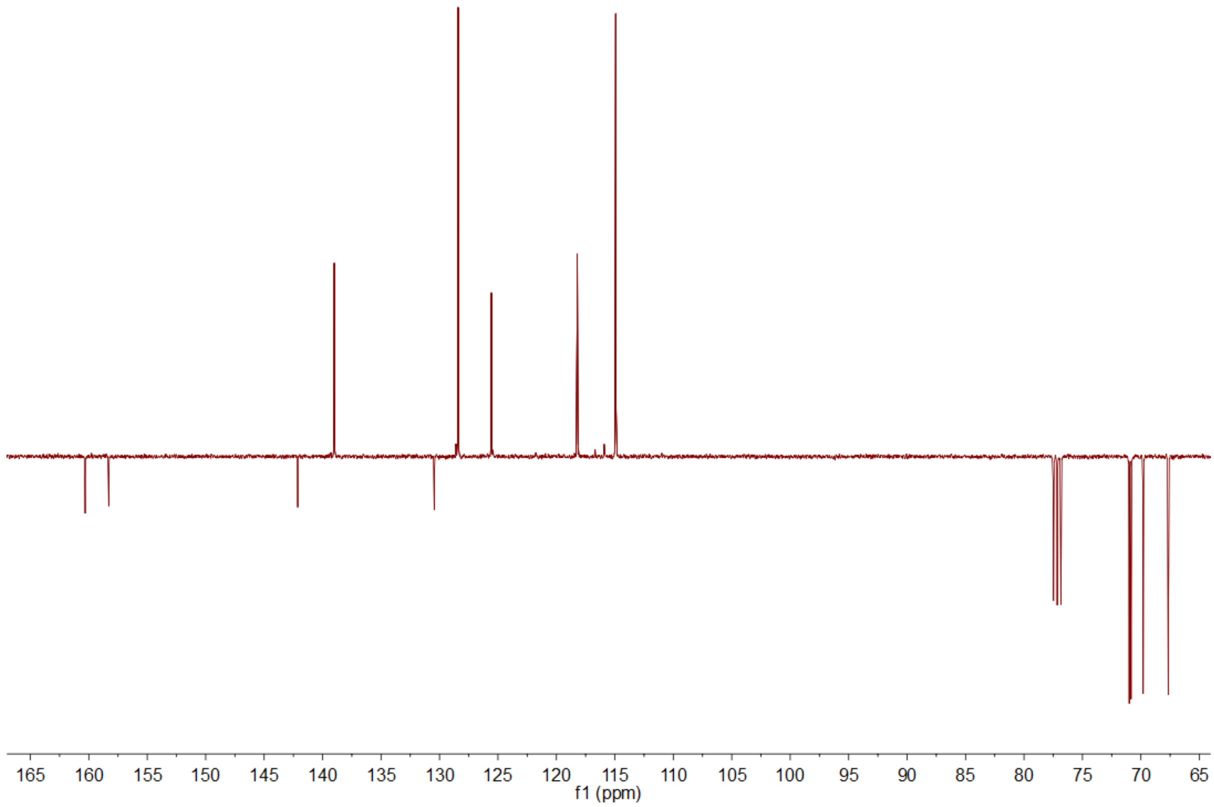

Figure S62 JMOD NMR ($CDCl_3$, 100 MHz) of 1e.

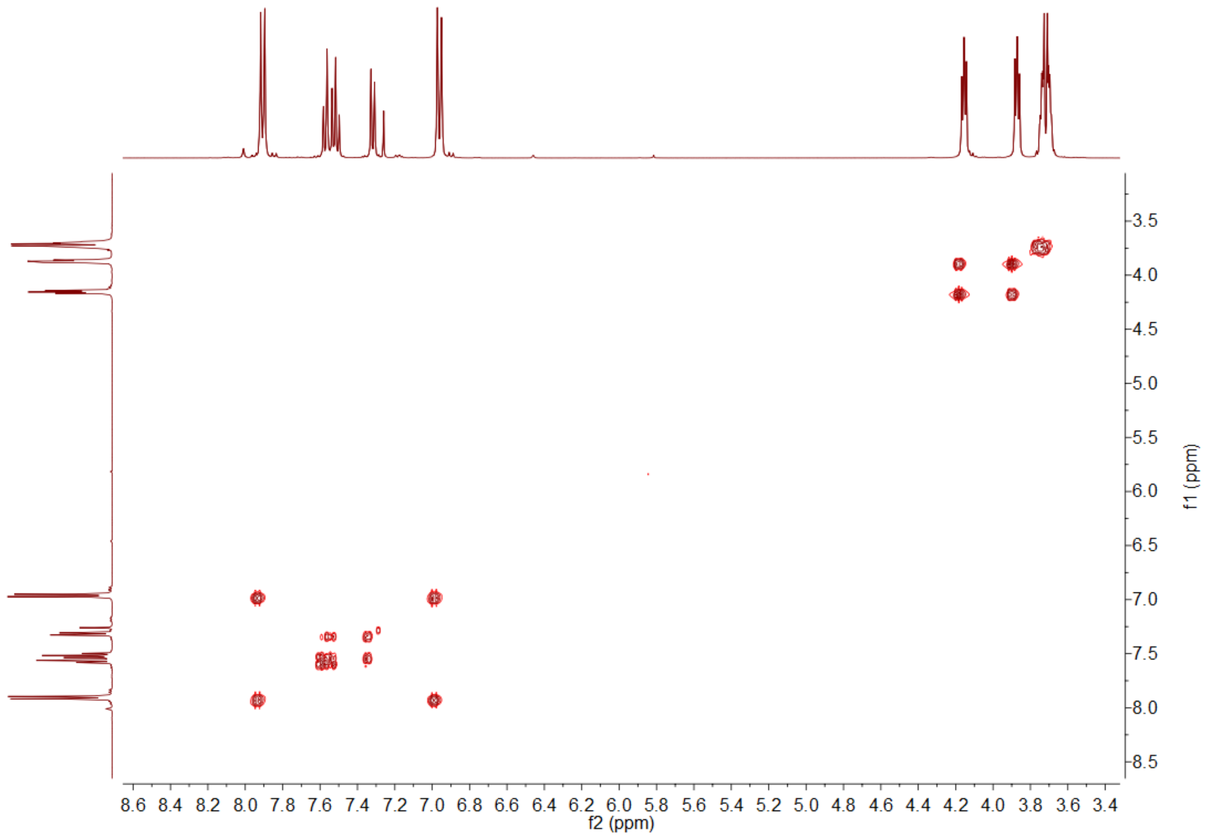

Figure S63 ^{1}H COSY NMR ($CDCl_3$, 400 MHz) of 1e.

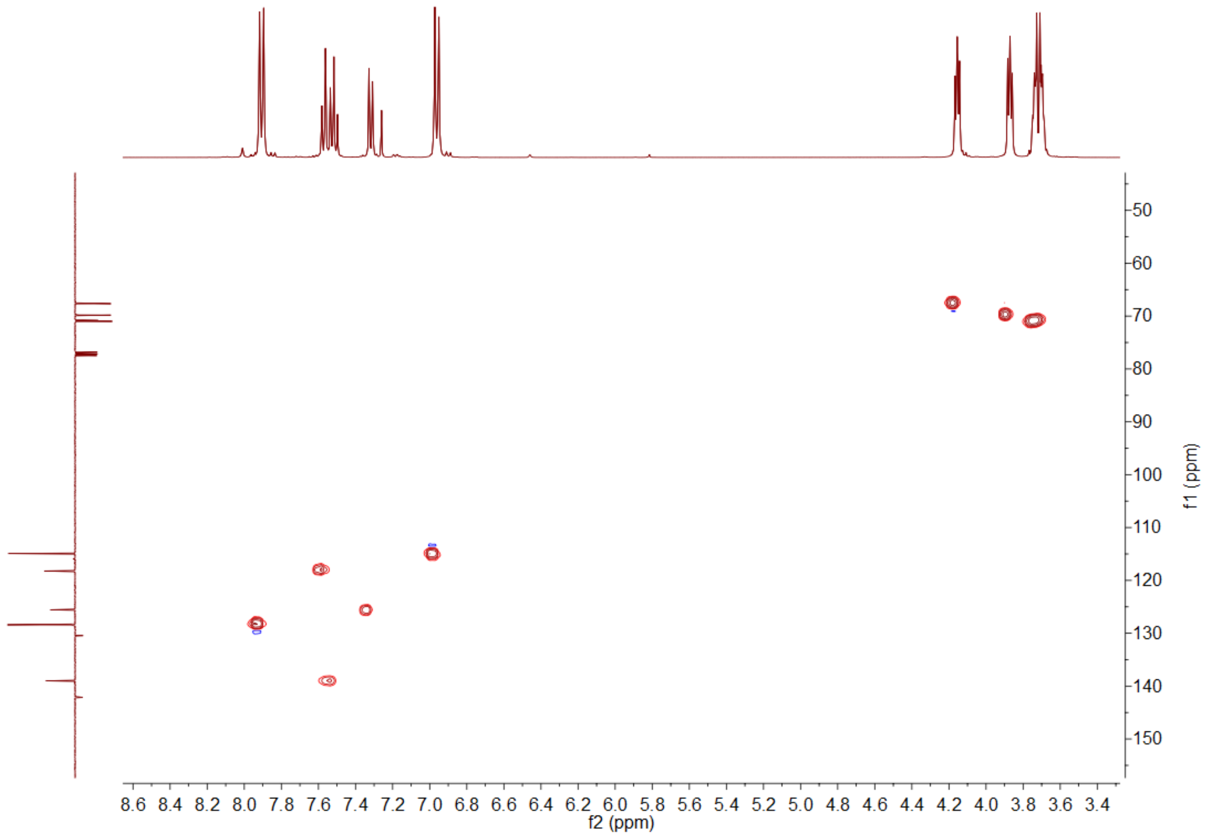

Figure S64 HSQC NMR ($CDCl_3$, 400 MHz) of 1e.

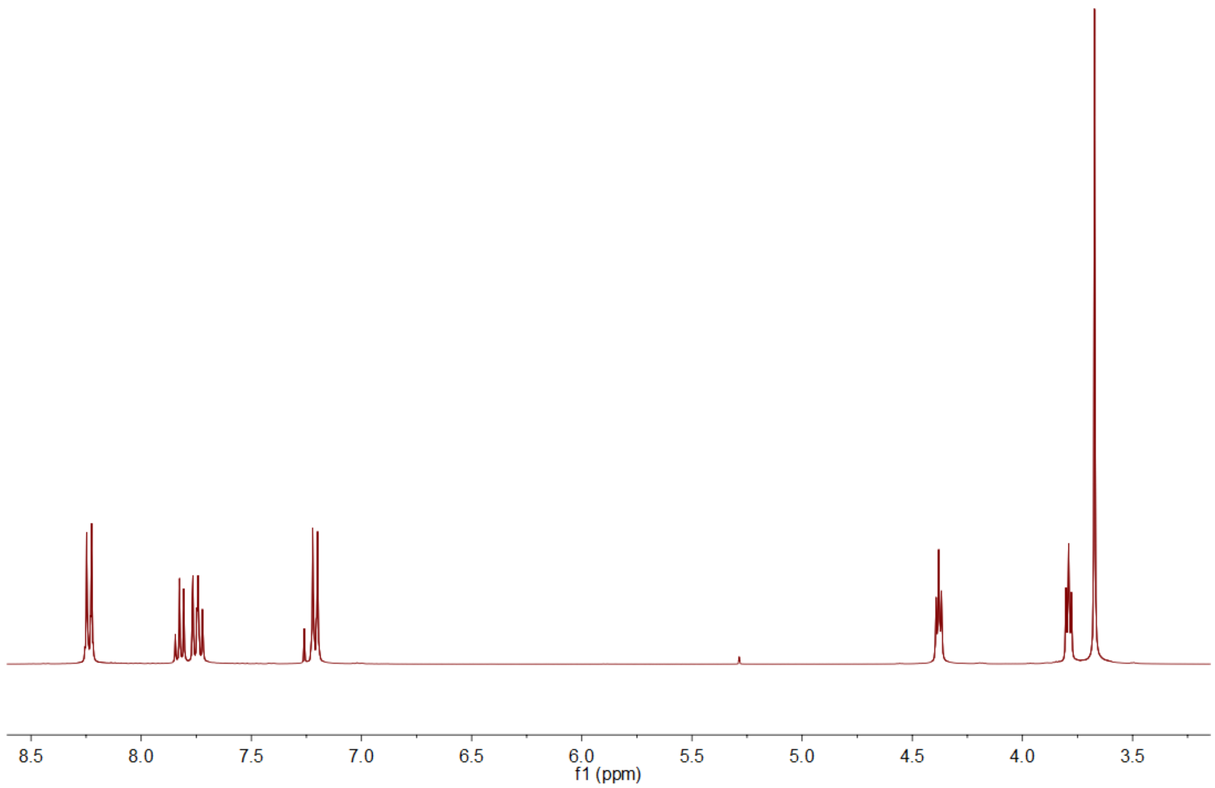

Figure S65 ^{1}H NMR ($CDCl_3$, 400 MHz) of 2e.

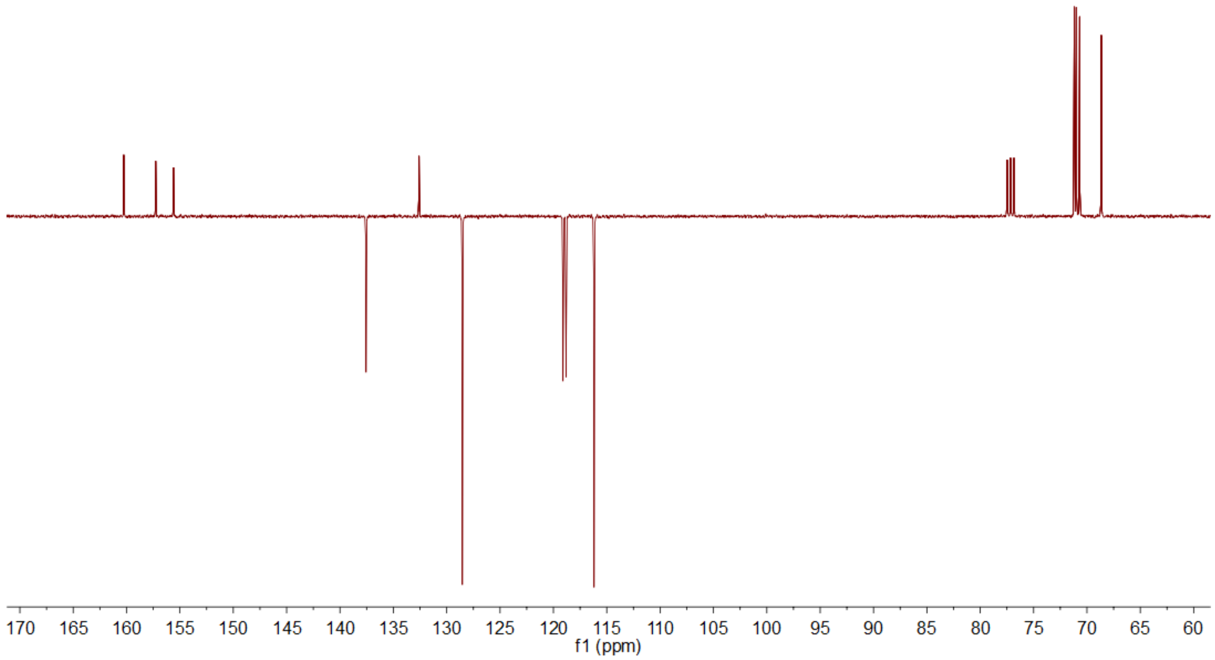

Figure S66 JMOD NMR ($CDCl_3$, 100 MHz) of 2e.

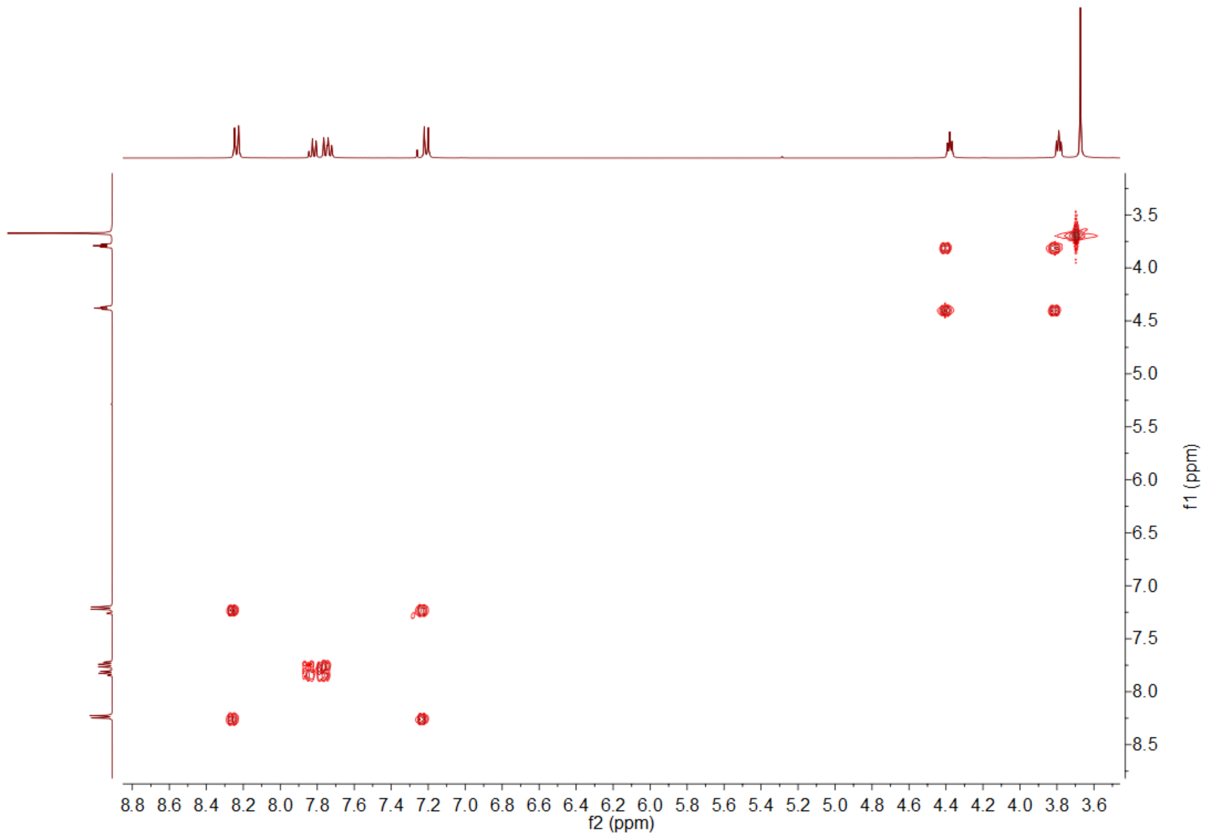

Figure S67 ^{1}H COSY NMR ($CDCl_3$, 400 MHz) of 2e.

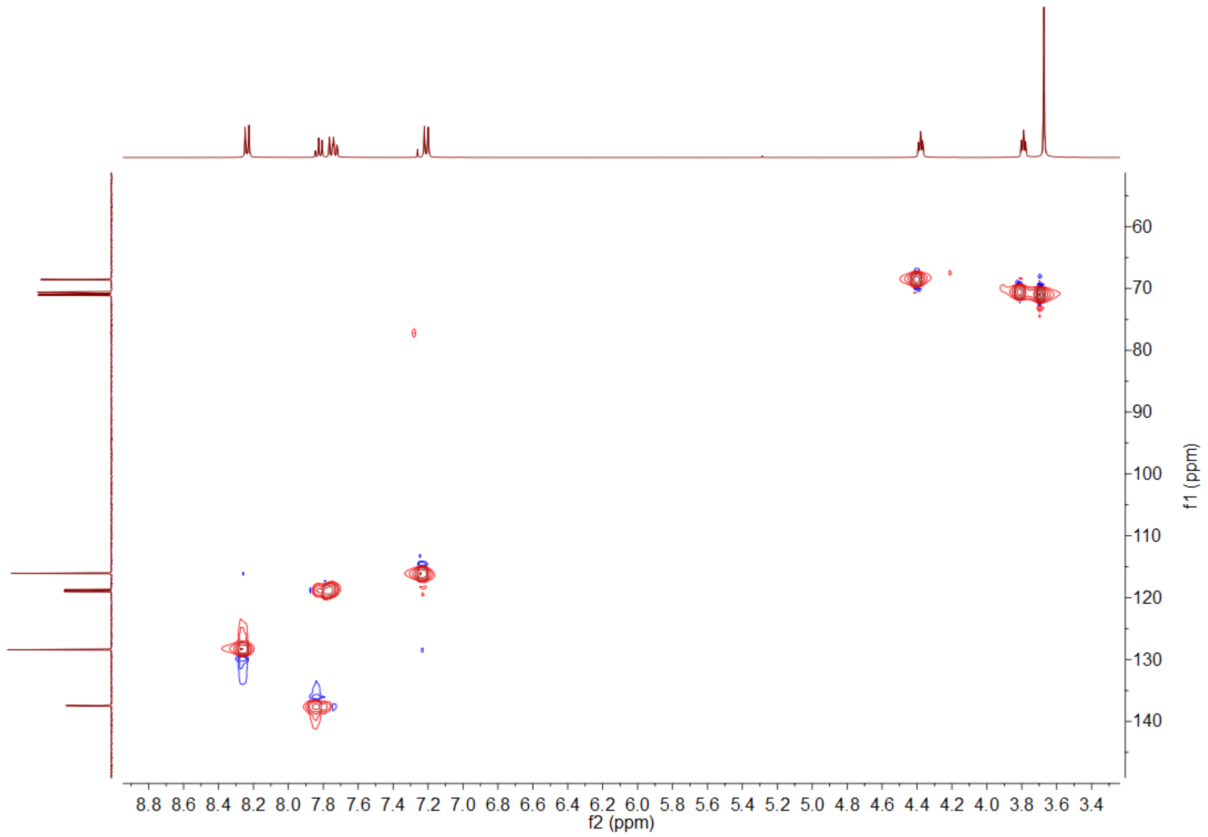

Figure S68 HSQC NMR ($CDCl_3$, 400 MHz) of 2e.

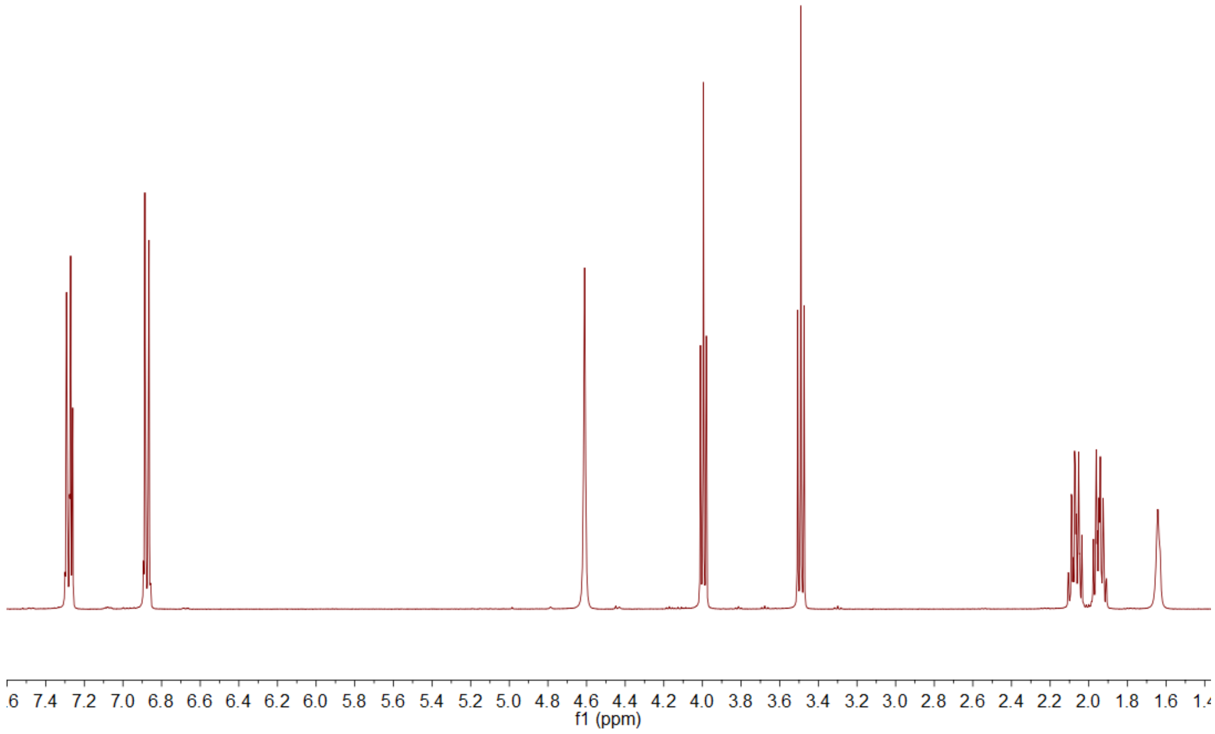

Figure S69 ^{1}H NMR ($CDCl_3$, 400 MHz) of S7.

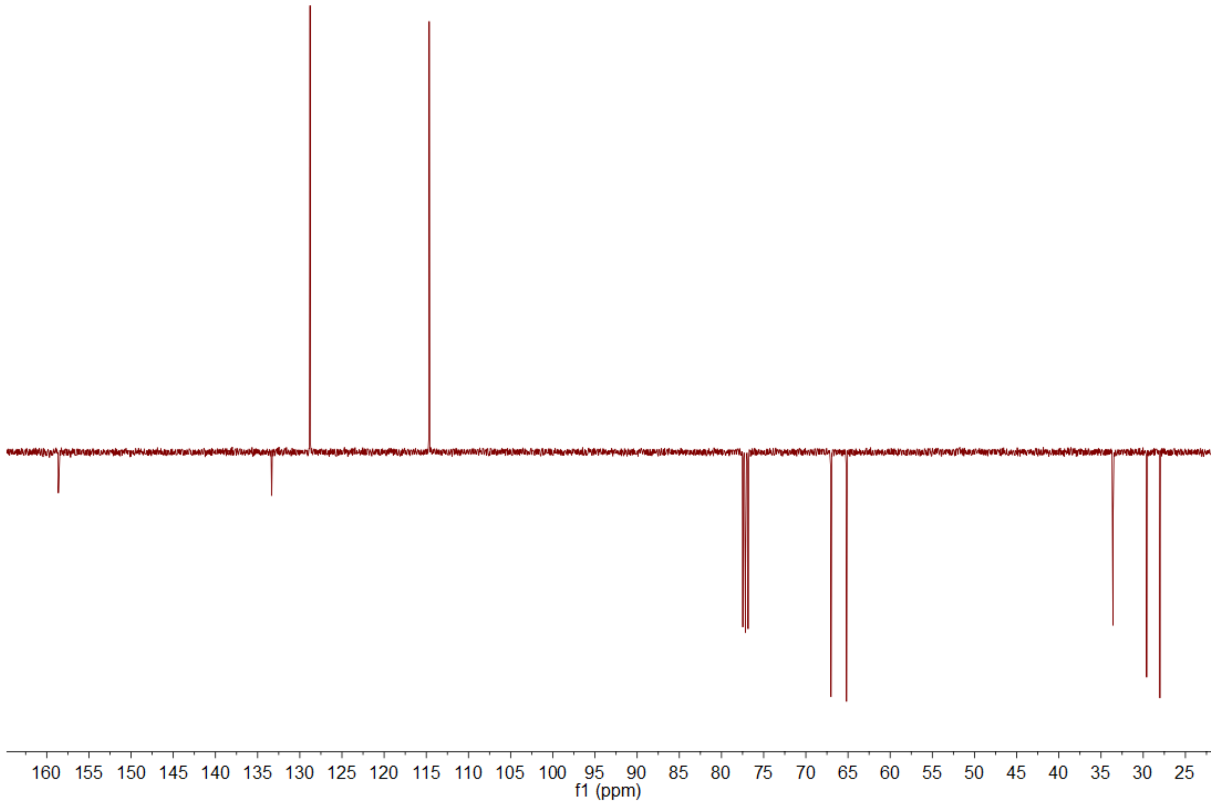

Figure S70 JMOD NMR ($CDCl_3$, 100 MHz) of S7.

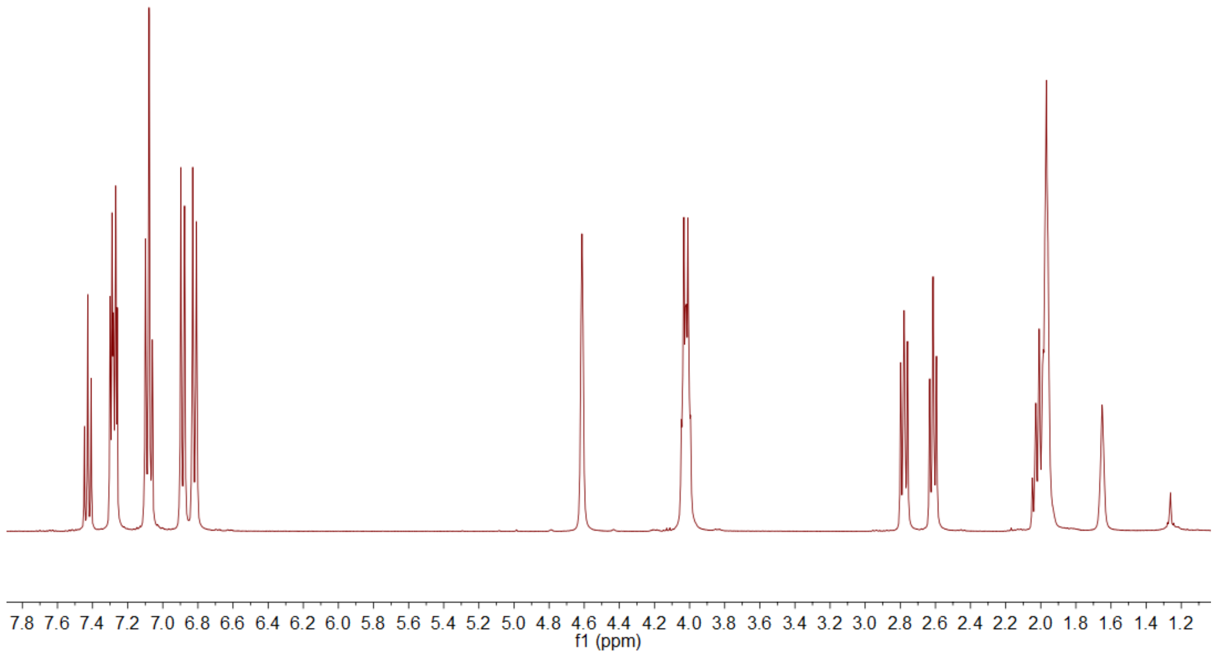

Figure S71 ^{1}H NMR ($CDCl_3$, 400 MHz) of S8.

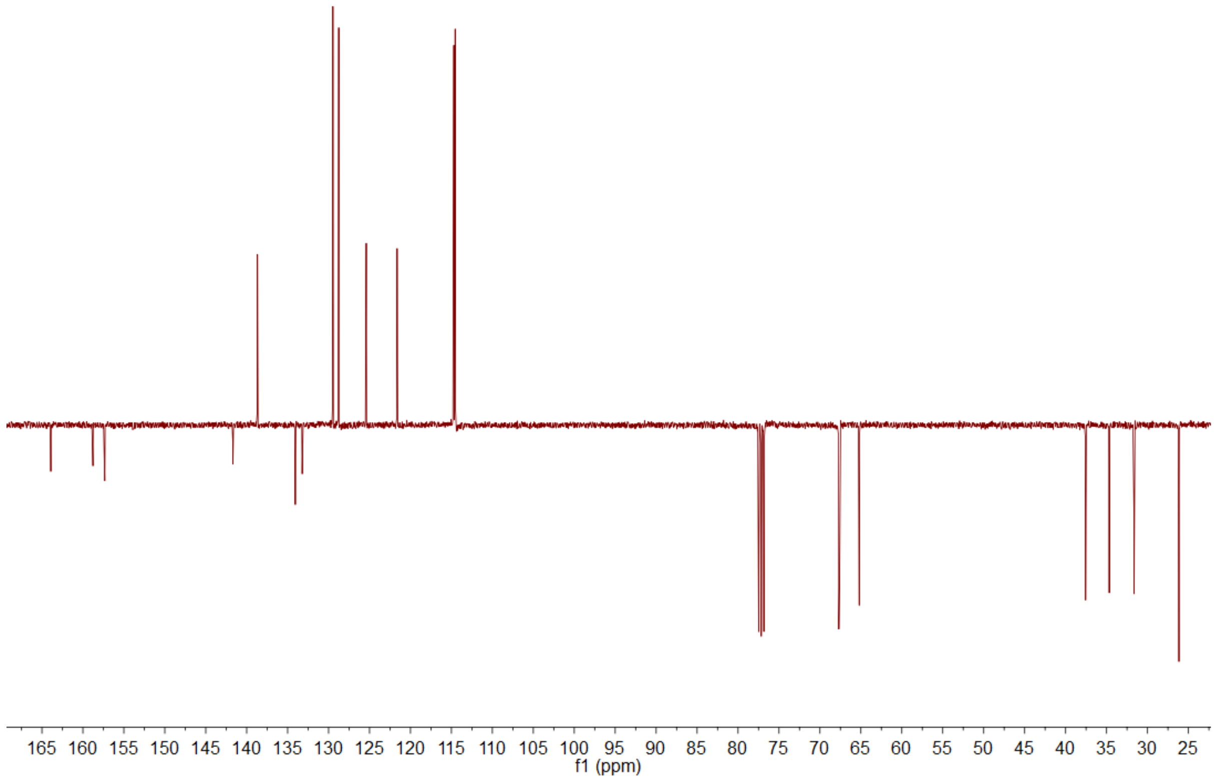

Figure S72 JMOD NMR ($CDCl_3$, 100 MHz) of S8.

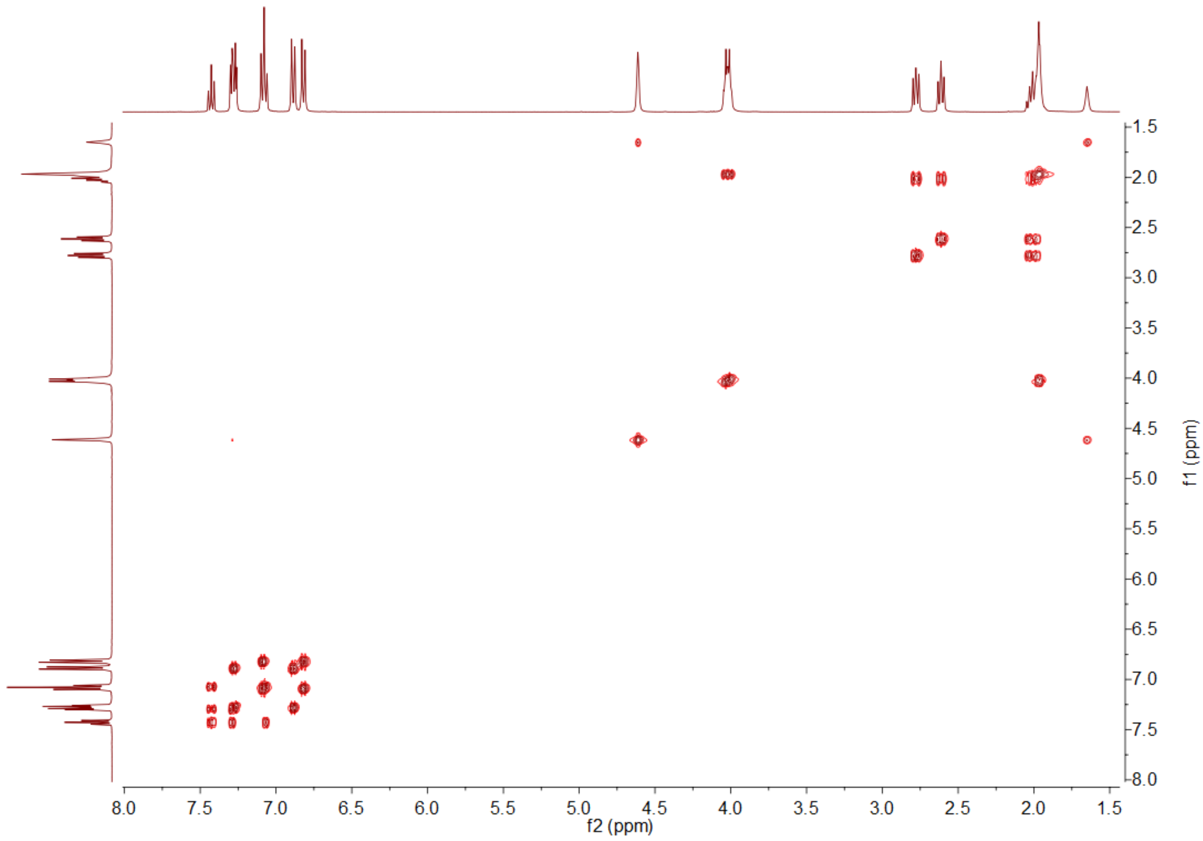

Figure S73 ^{1}H COSY NMR ($CDCl_3$, 400 MHz) of S8.

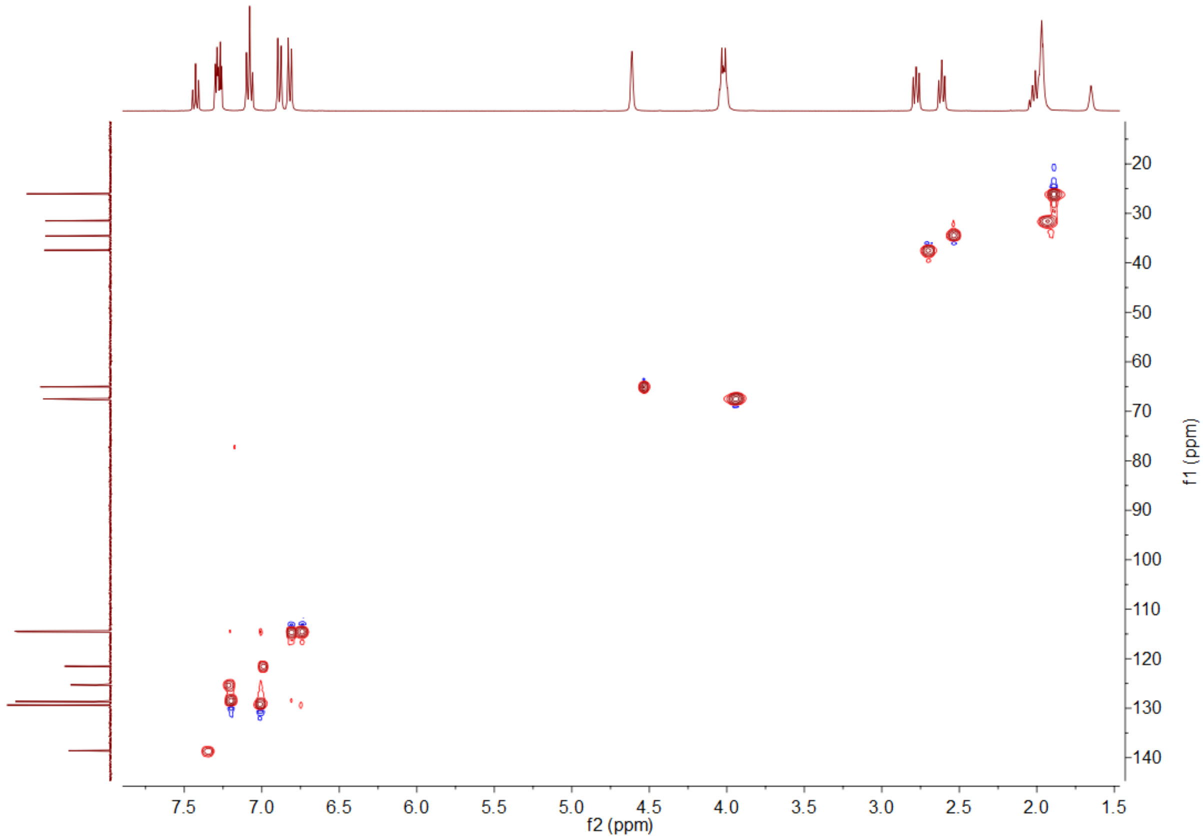

Figure S74 HSQC NMR ($CDCl_3$, 400 MHz) of S8.

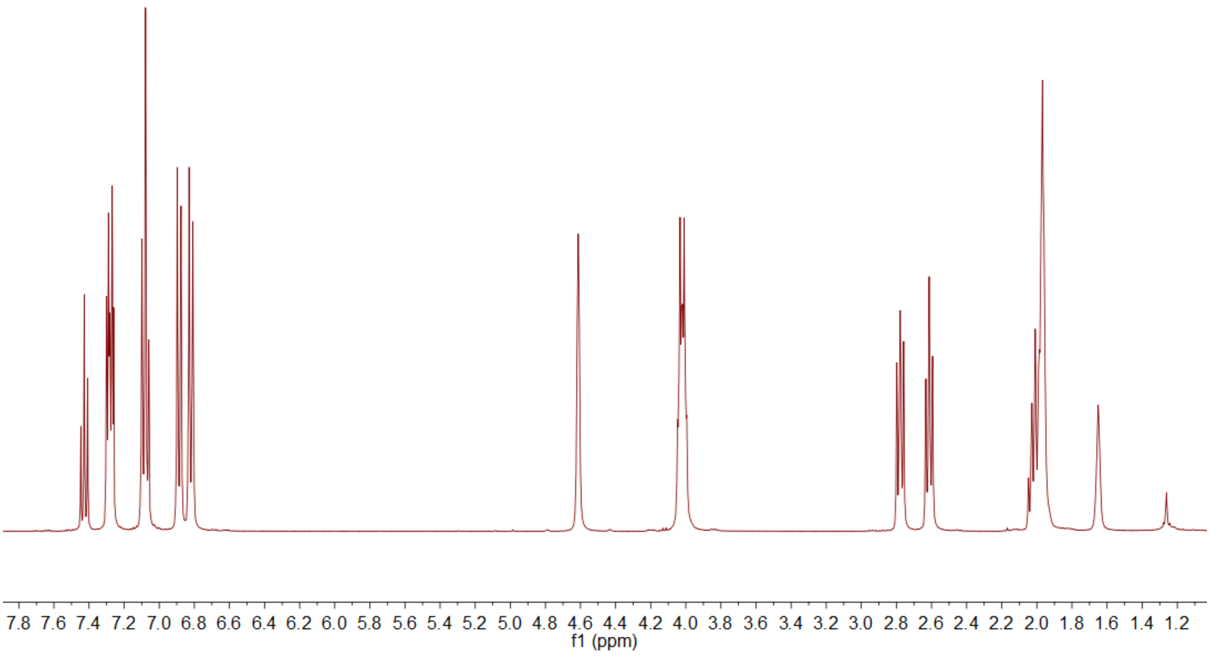

Figure S75 ^{1}H NMR ($CDCl_3$, 400 MHz) of 1f.

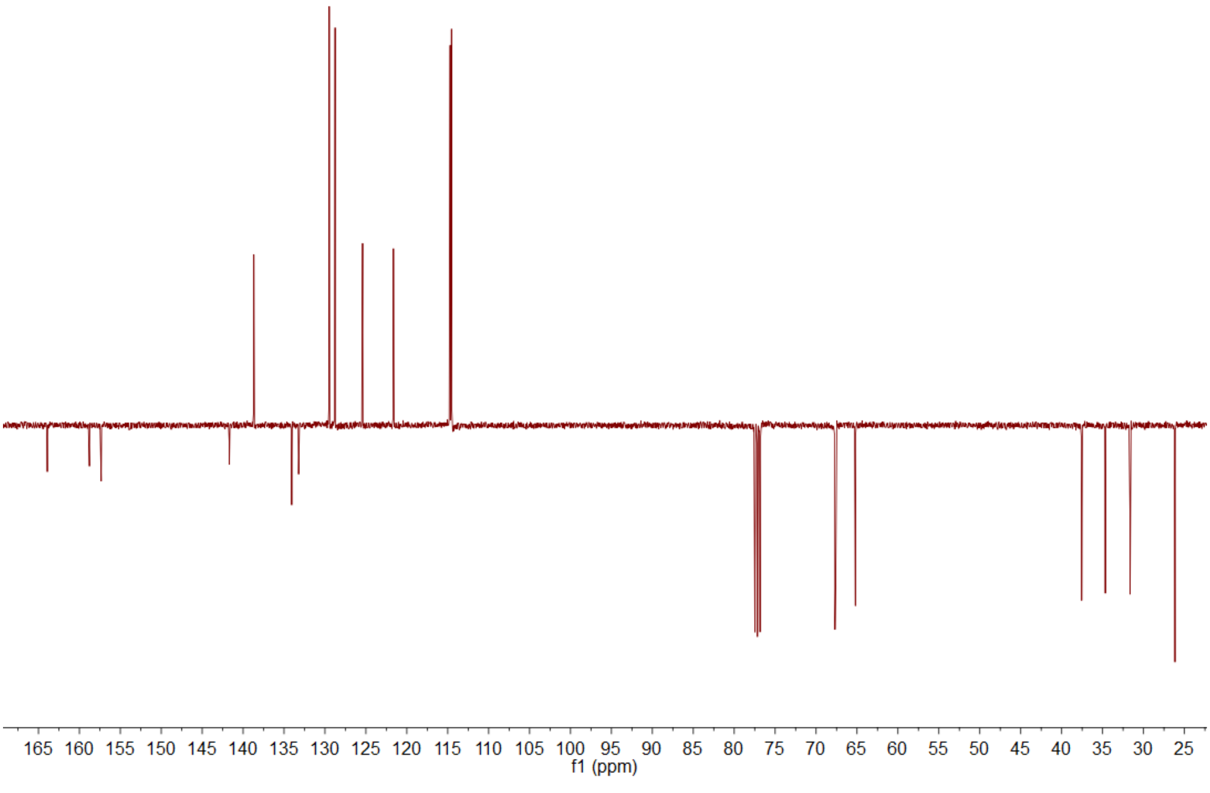

Figure S76 JMOD NMR ($CDCl_3$, 100 MHz) of 1f.

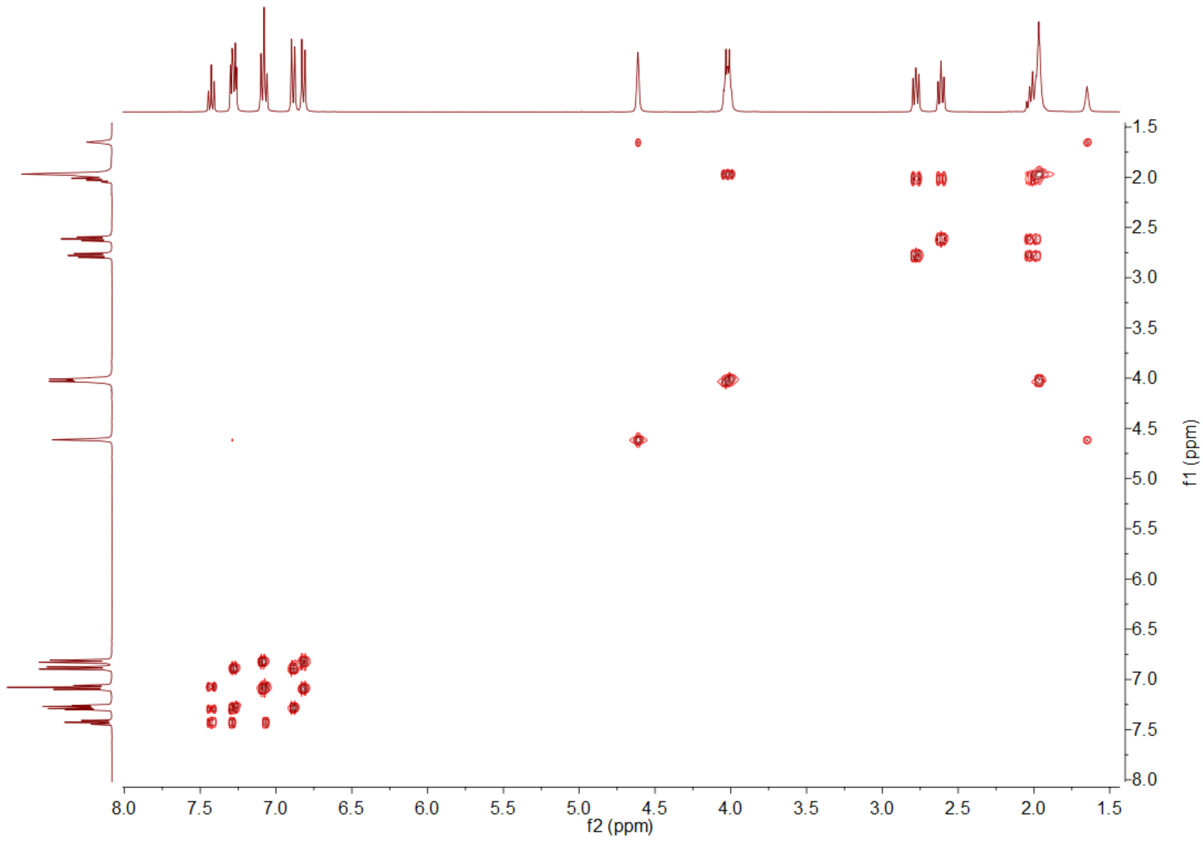

Figure S77 ^{1}H COSY NMR ($CDCl_3$, 400 MHz) of 1f.

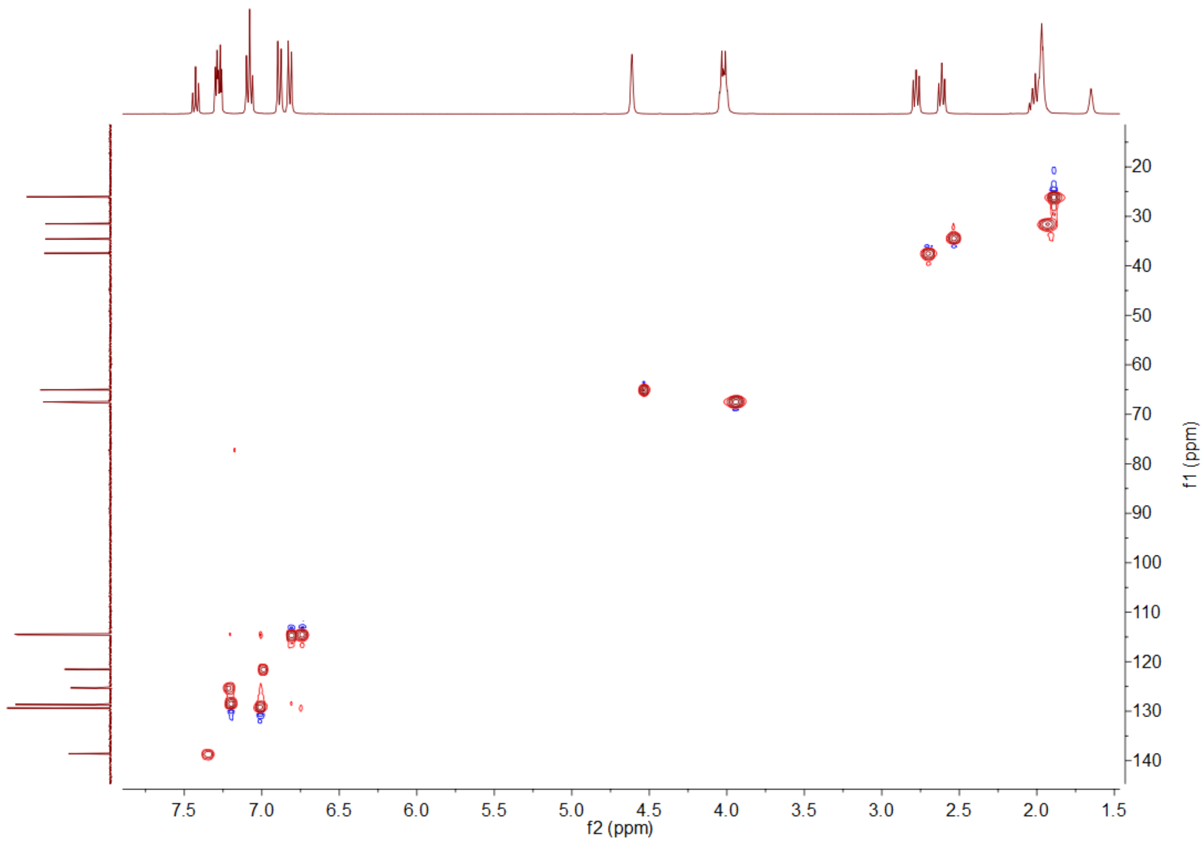

Figure S78 HSQC NMR ($CDCl_3$, 400 MHz) of 1f.

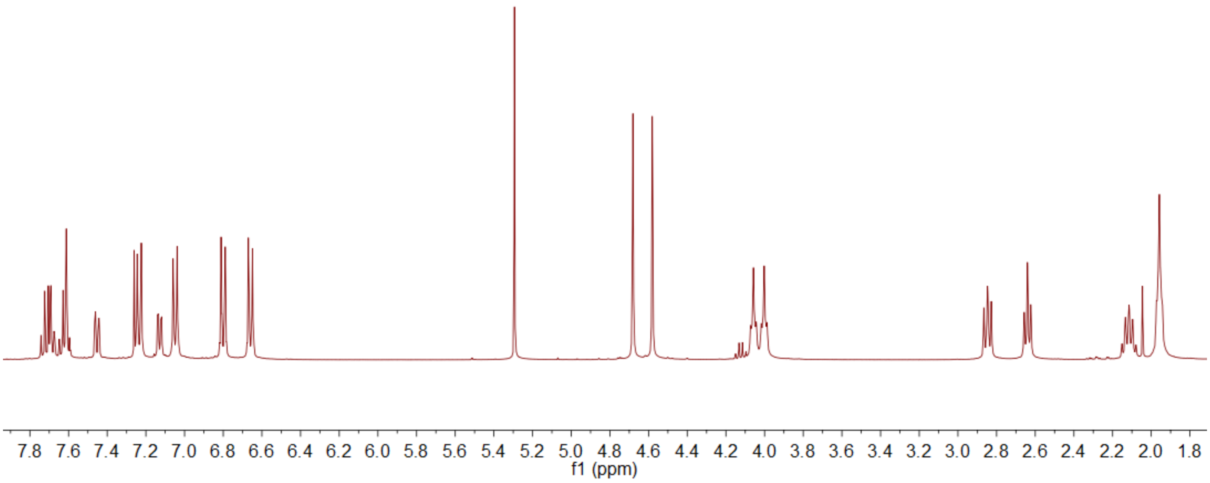

Figure S79 ^{1}H NMR ($CDCl_3$, 400 MHz) of 2f.

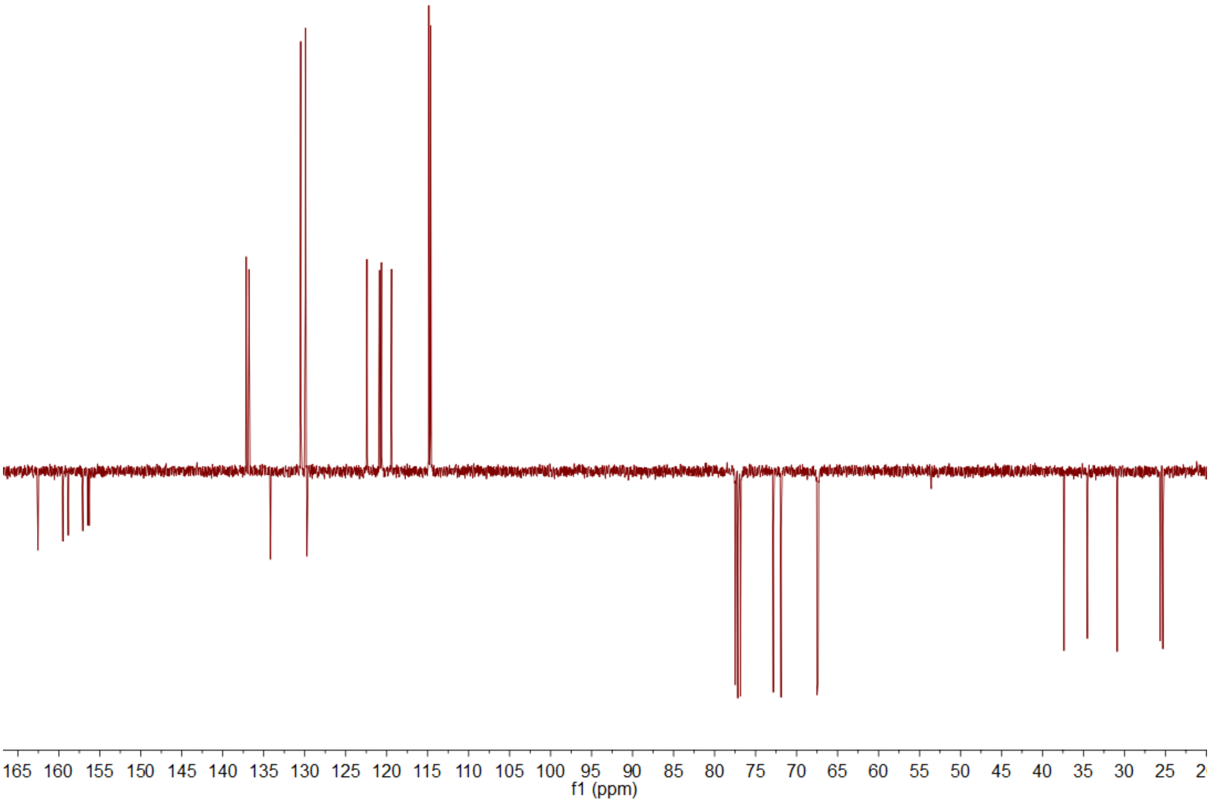

Figure S80 JMOD NMR ($CDCl_3$, 100 MHz) of 2f.

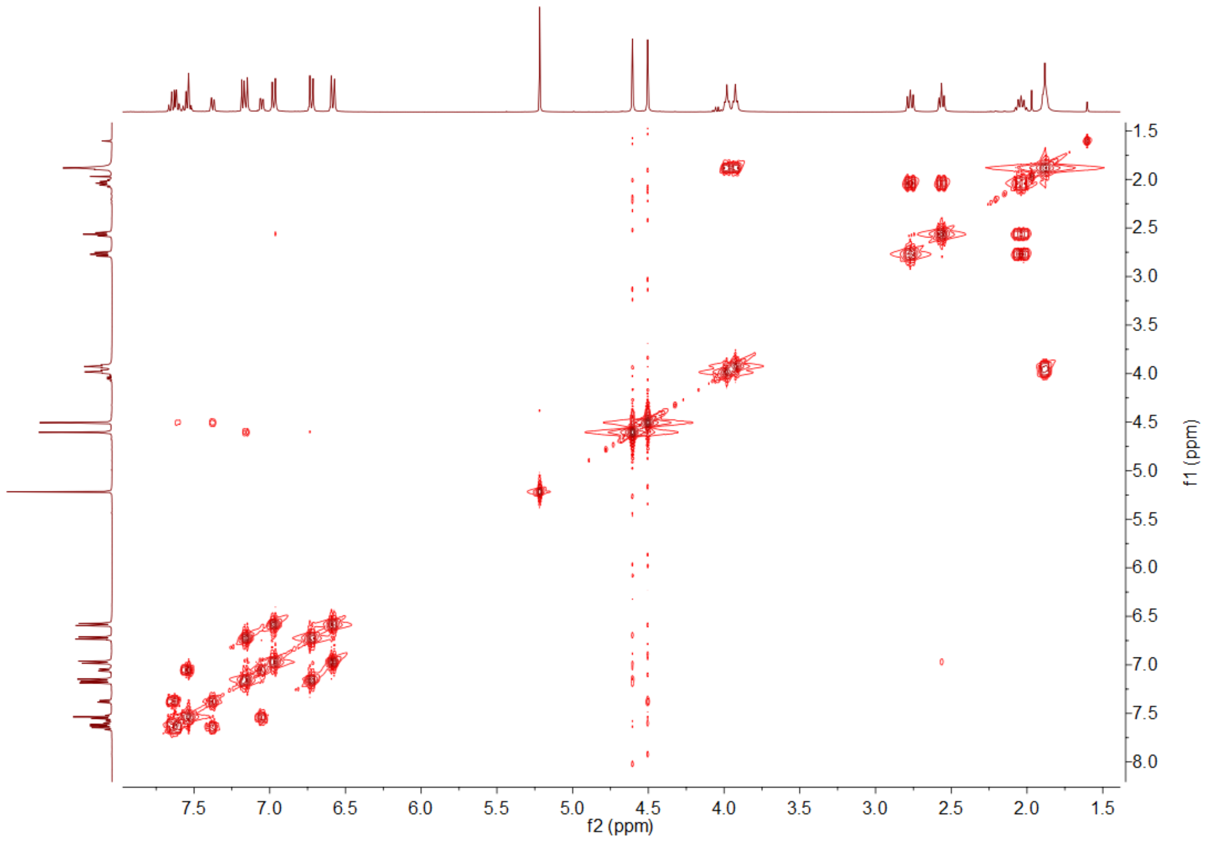

Figure S81 ^{1}H COSY NMR ($CDCl_3$, 400 MHz) of 2f.

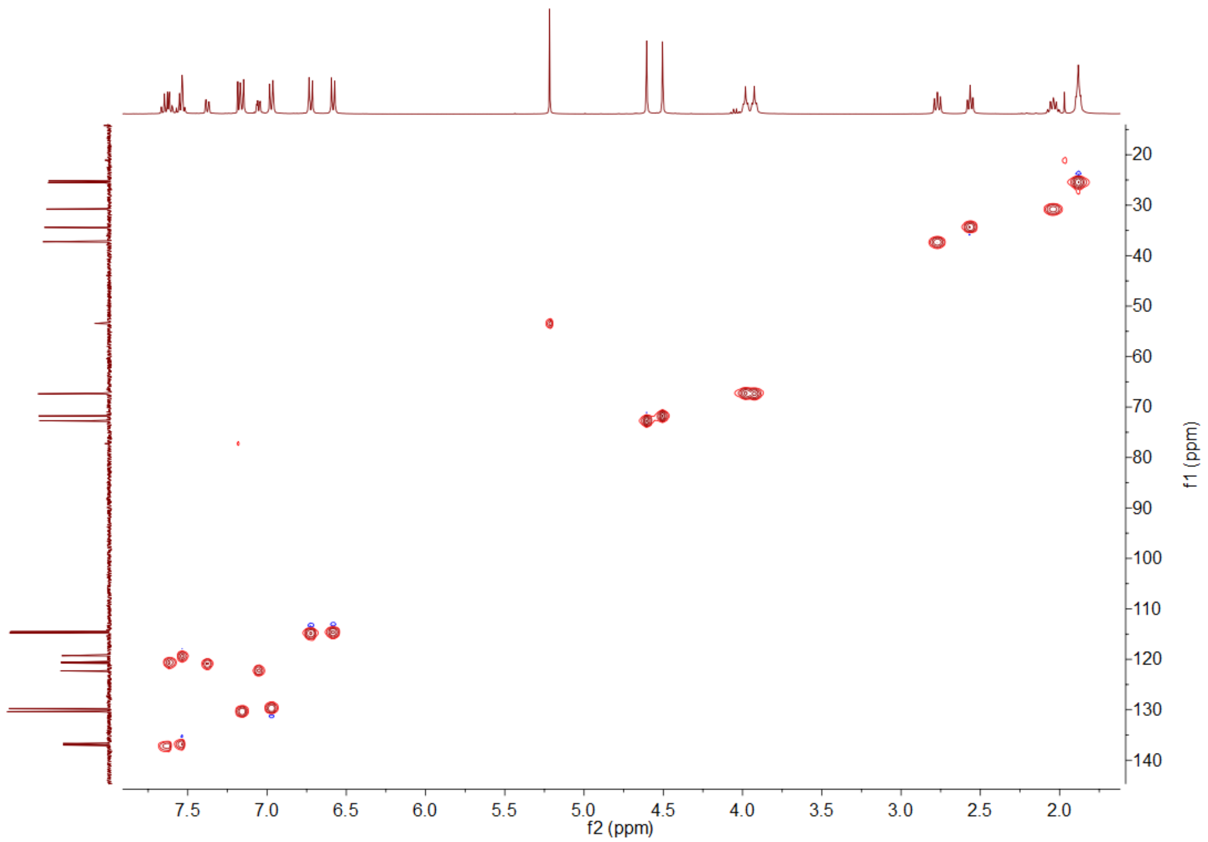

Figure S82 HSQC NMR ($CDCl_3$, 400 MHz) of 2f.

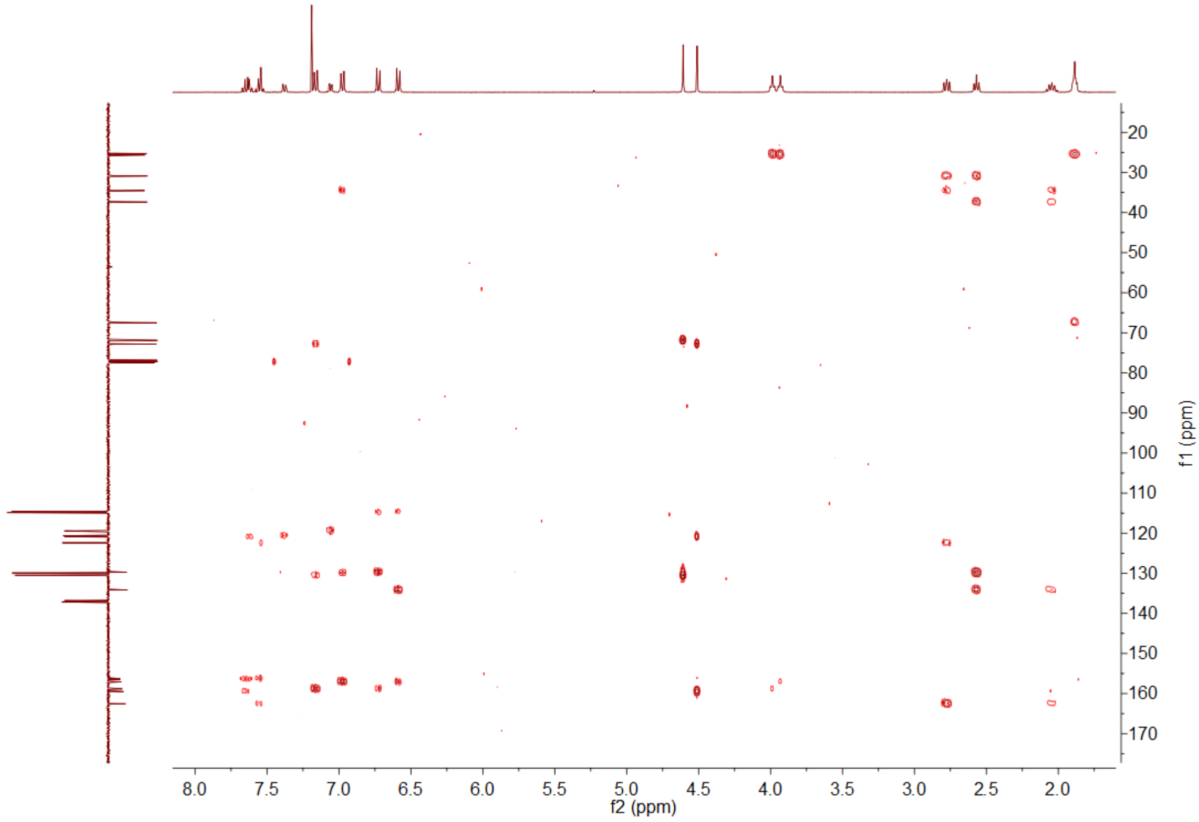

Figure S83 HMBC NMR ($CDCl_3$, 400 MHz) of 2f.

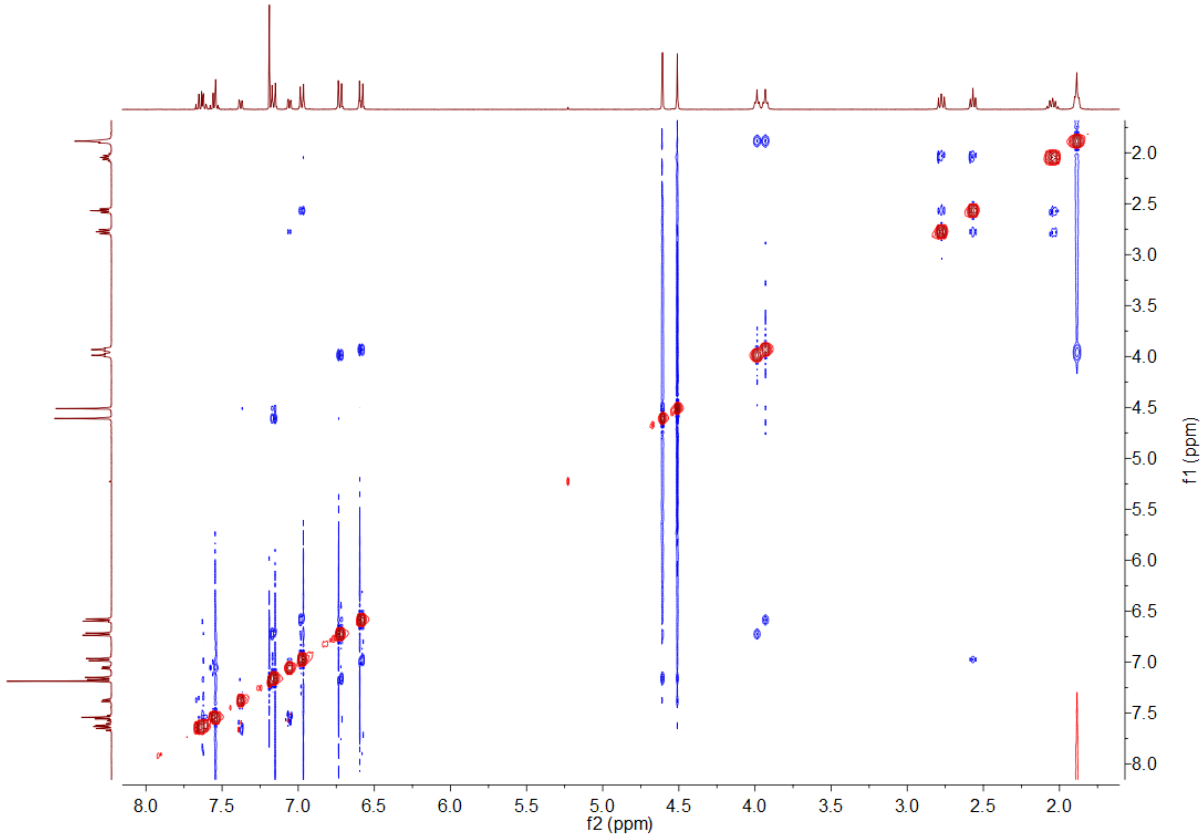

Figure S84 ^{1}H NOESY NMR ($CDCl_3$, 400 MHz) of 2f.

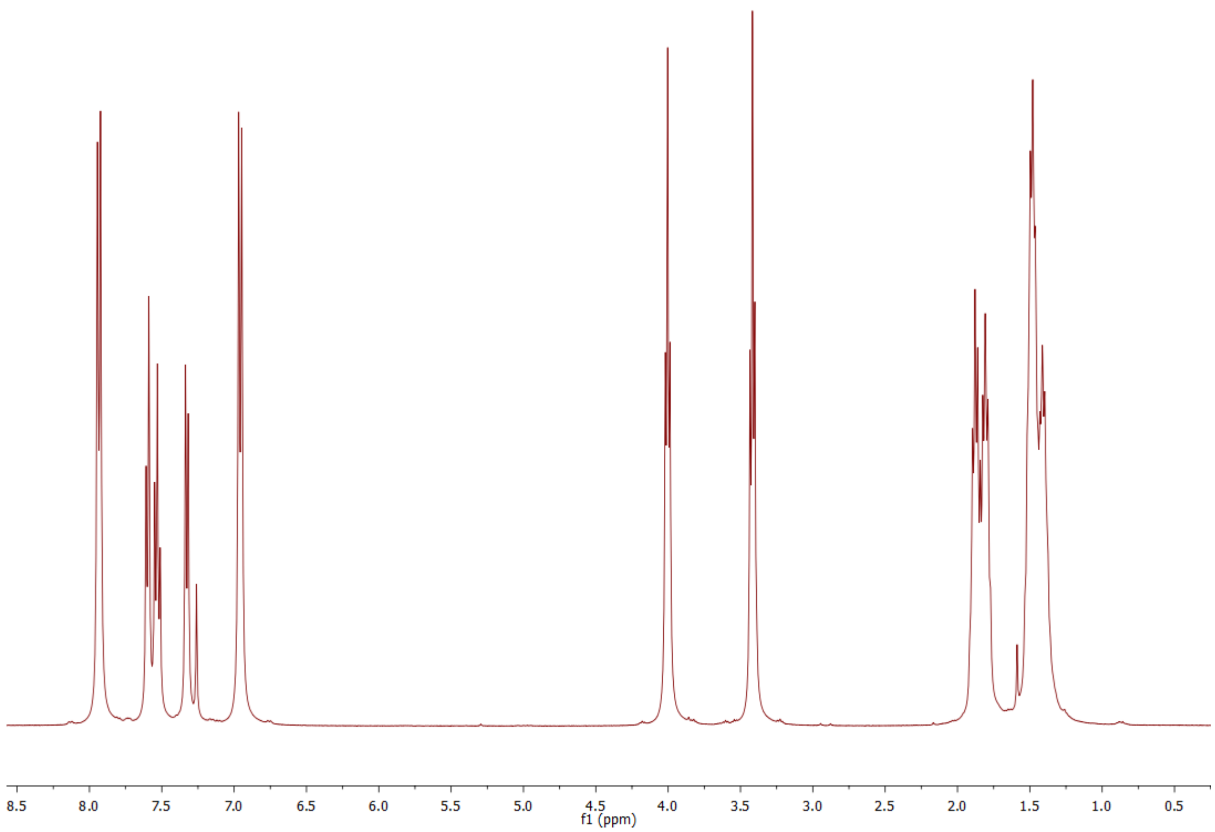

Figure S85 ^{1}H NMR ($CDCl_3$, 400 MHz) of S9.

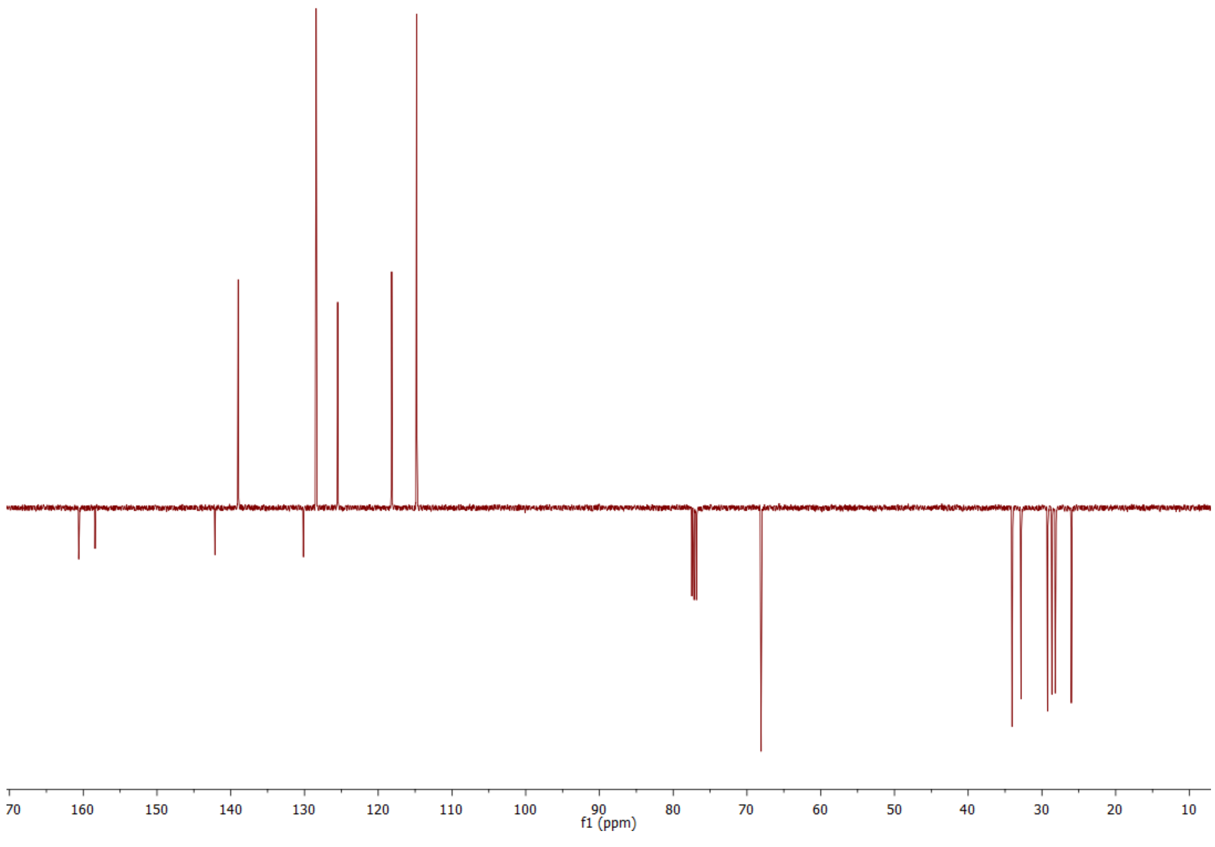

Figure S86 JMOD NMR ($CDCl_3$, 100 MHz) of S9.

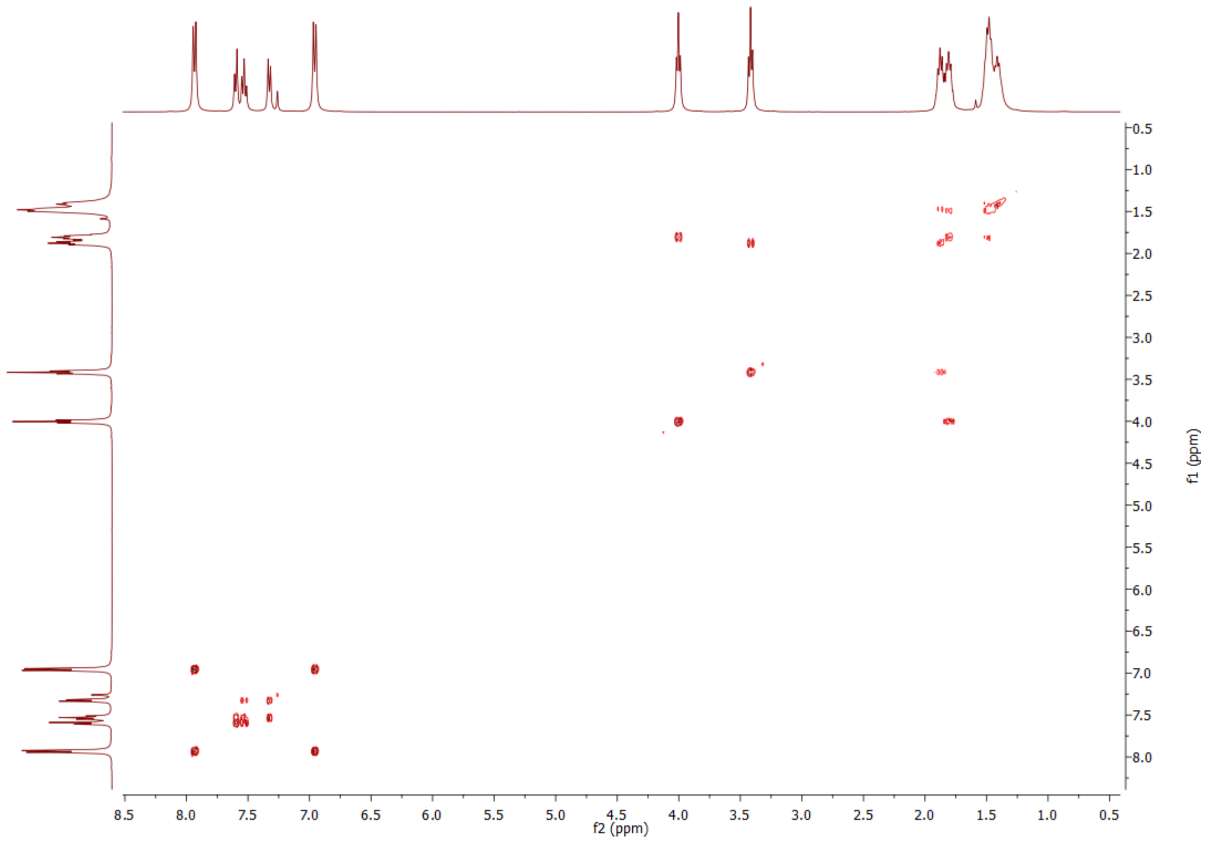

Figure S87 ^{1}H COSY NMR ($CDCl_3$, 400 MHz) of S9.

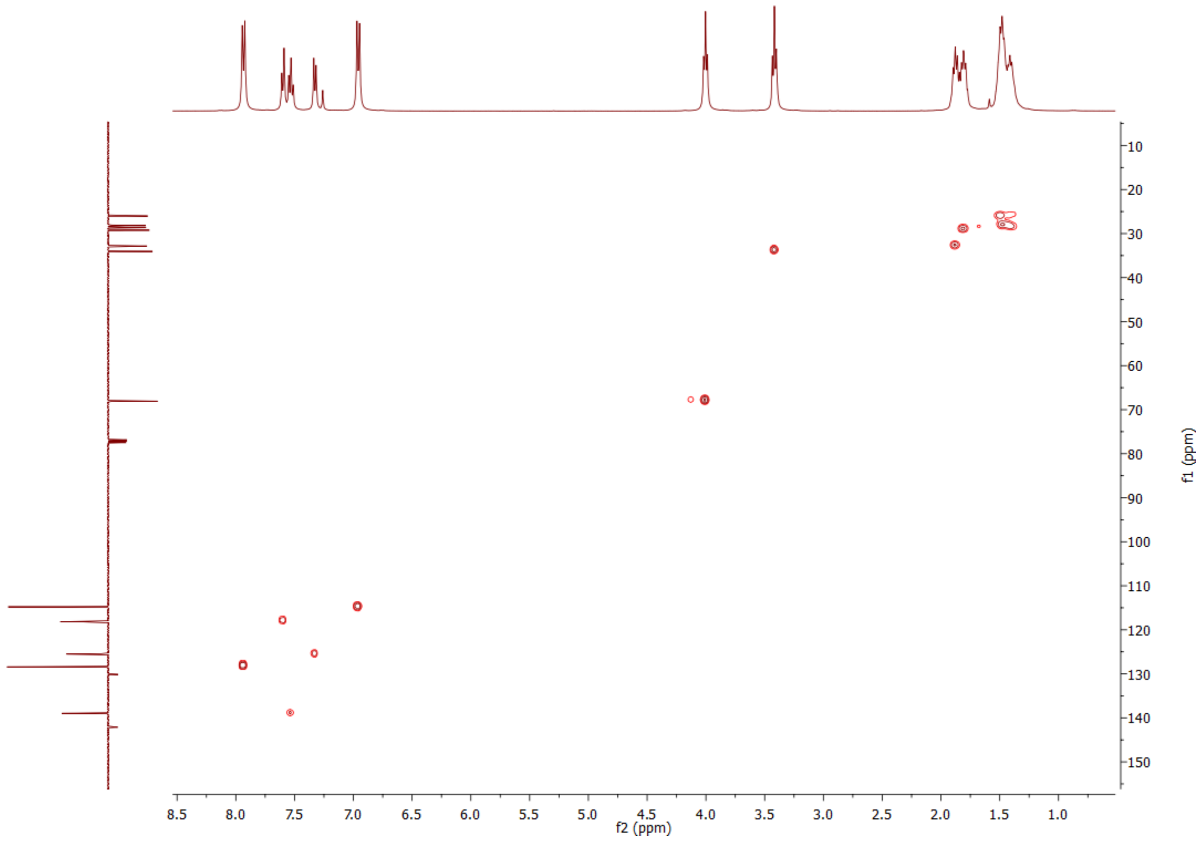

Figure S88 HSQC NMR ($CDCl_3$, 400 MHz) of S9.

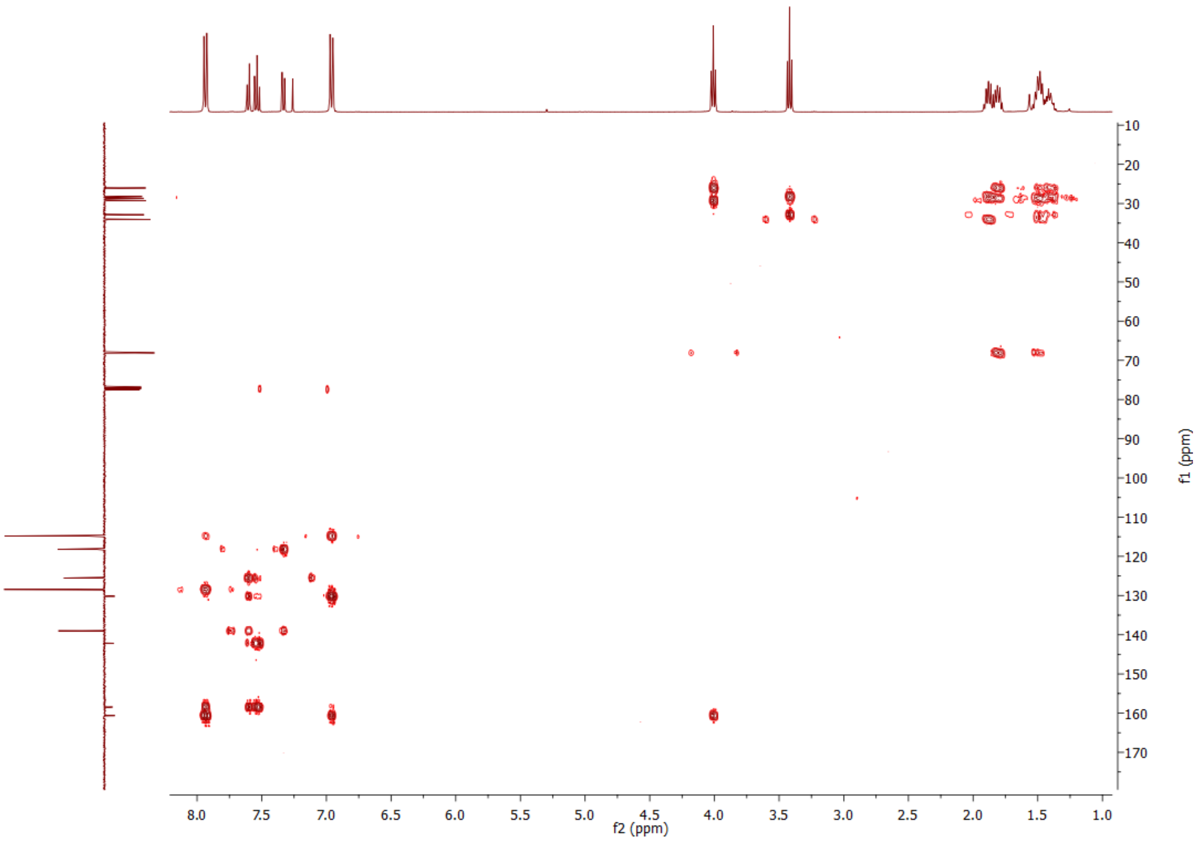

Figure S89 HMBC NMR ($CDCl_3$, 400 MHz) of S9.

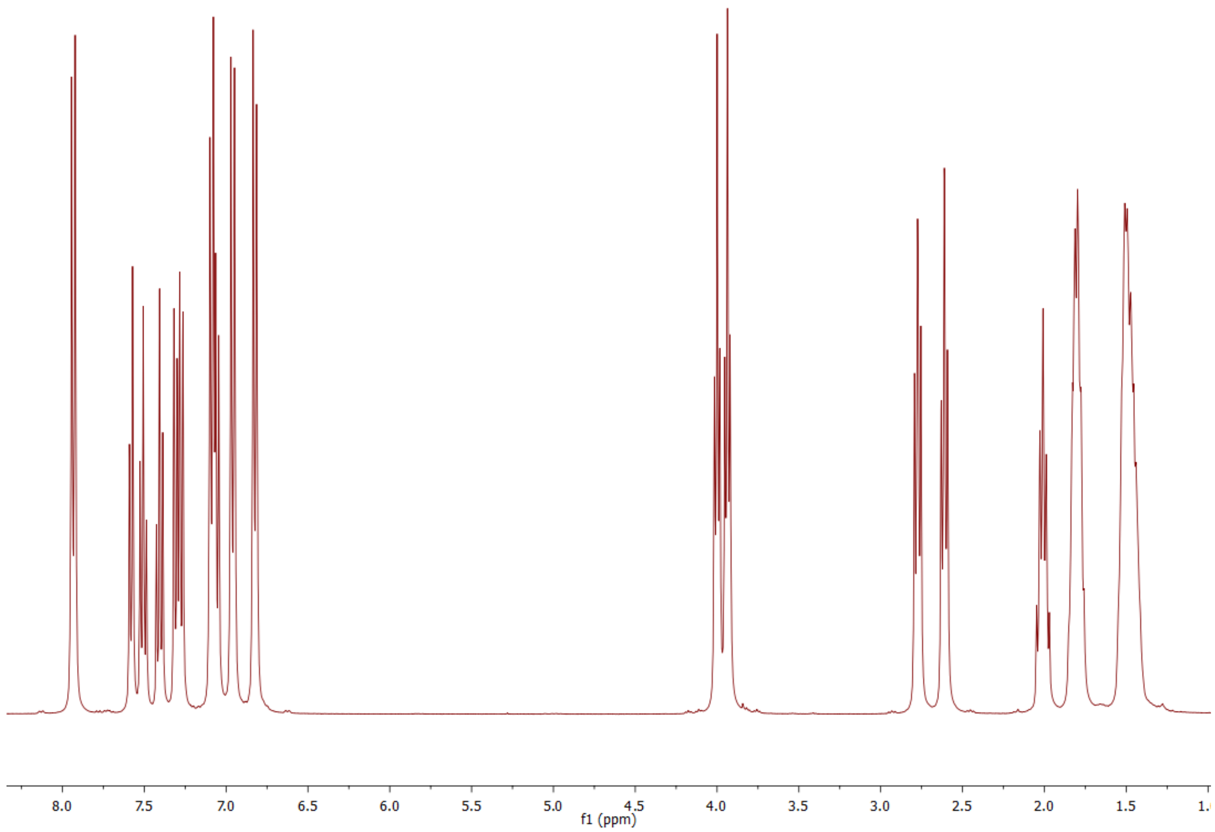

Figure S90 ^{1}H NMR ($CDCl_3$, 400 MHz) of 1g.

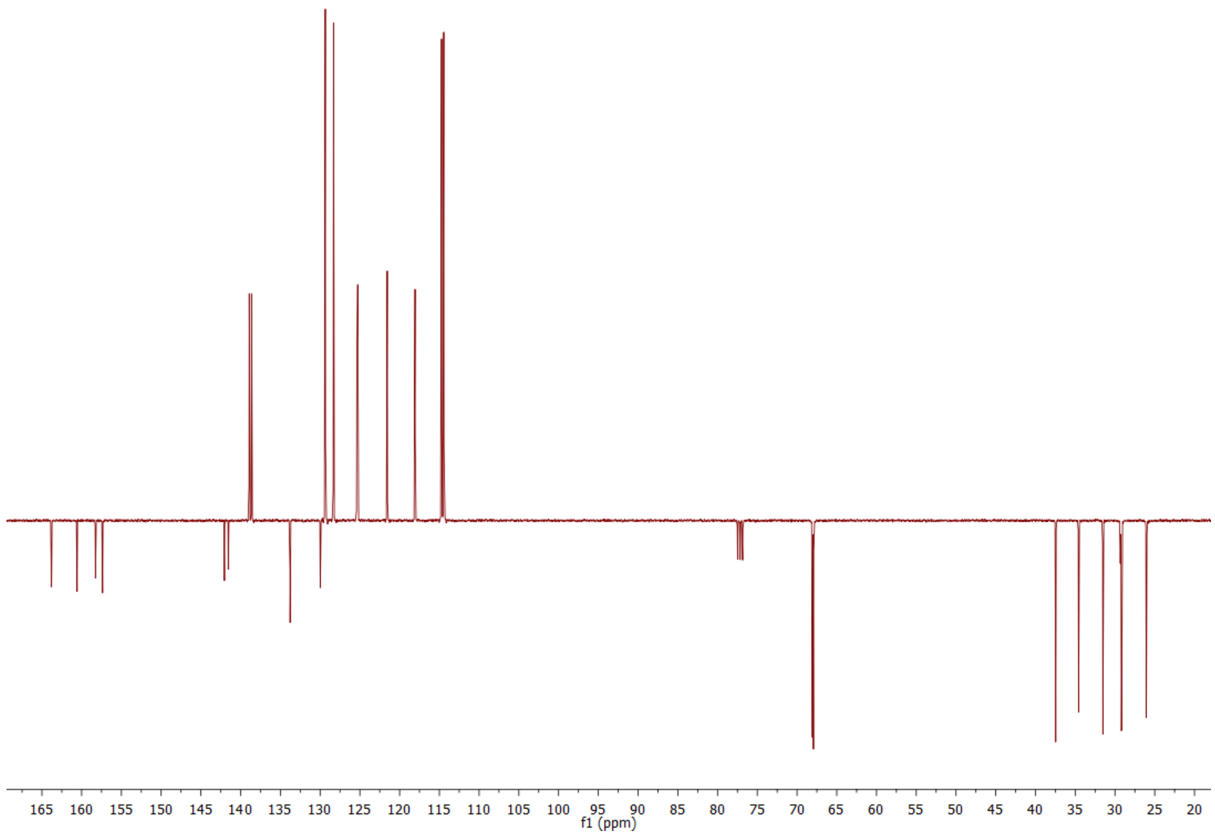

Figure S91 JMOD NMR ($CDCl_3$, 100 MHz) of 1g.

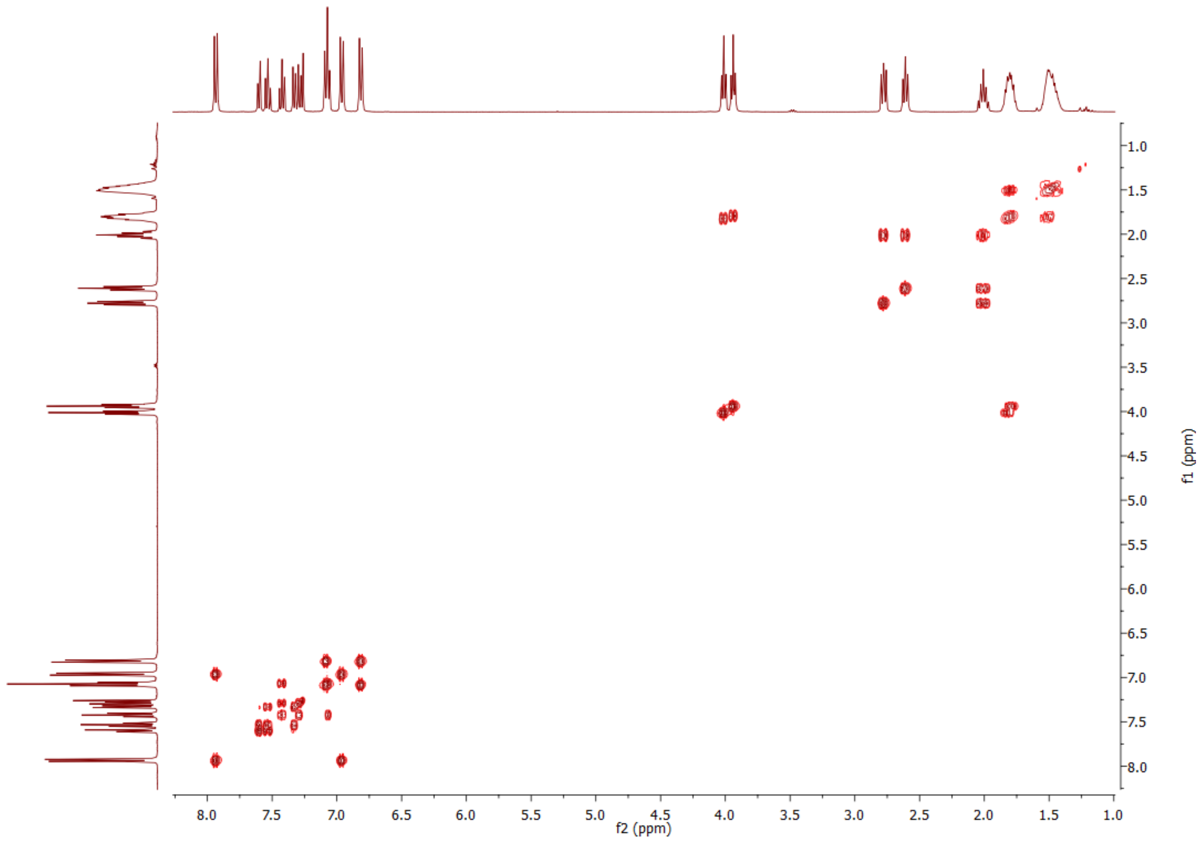

Figure S92 ^{1}H COSY NMR ($CDCl_3$, 400 MHz) of 1g.

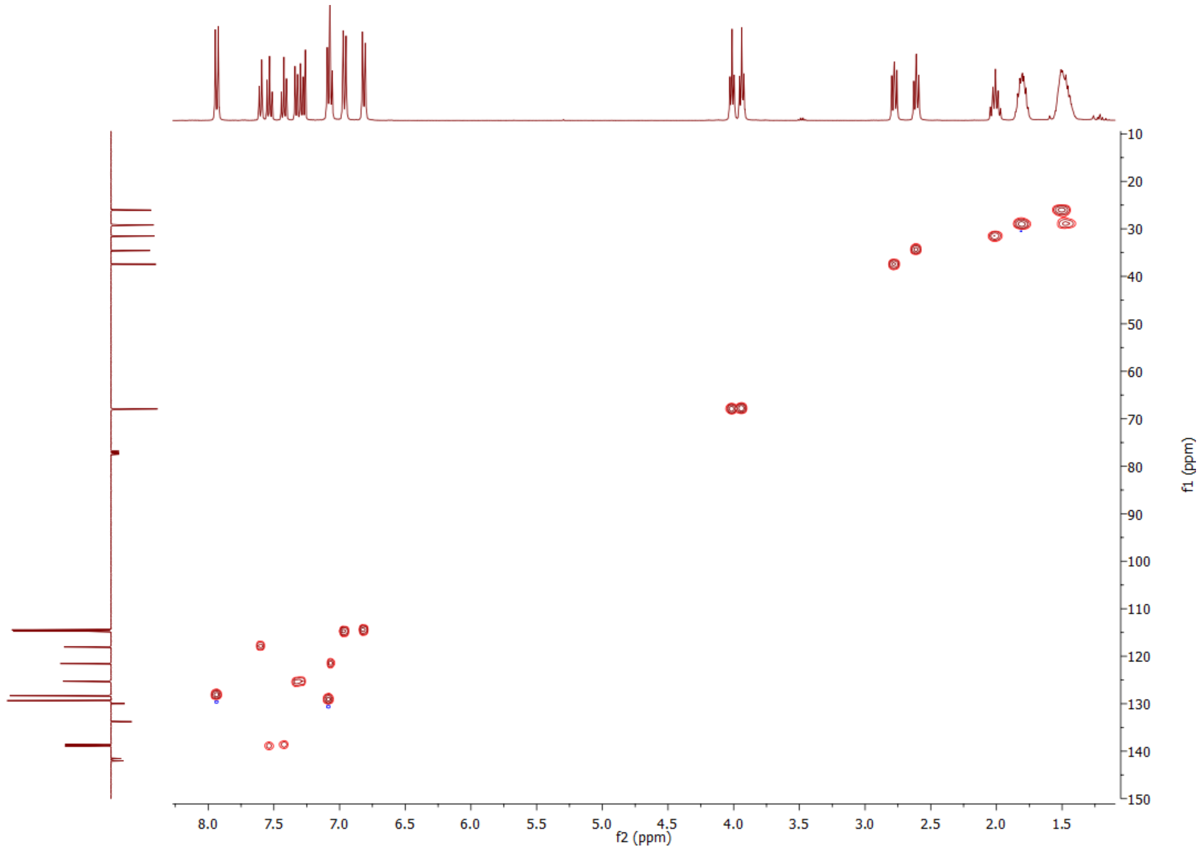

Figure S93 HSQC NMR ($CDCl_3$, 400 MHz) of 1g.

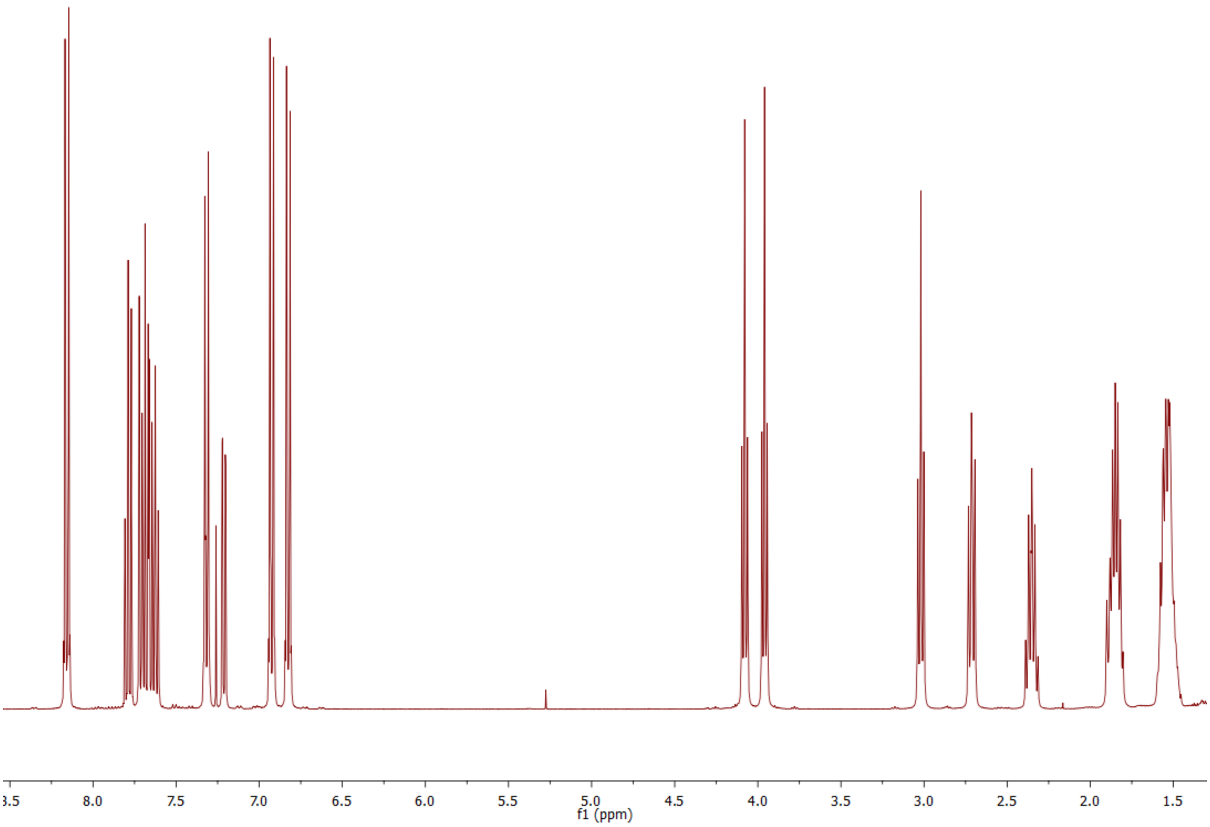

Figure S94 ¹H NMR ($CDCl_3$, 400 MHz) of 2g.

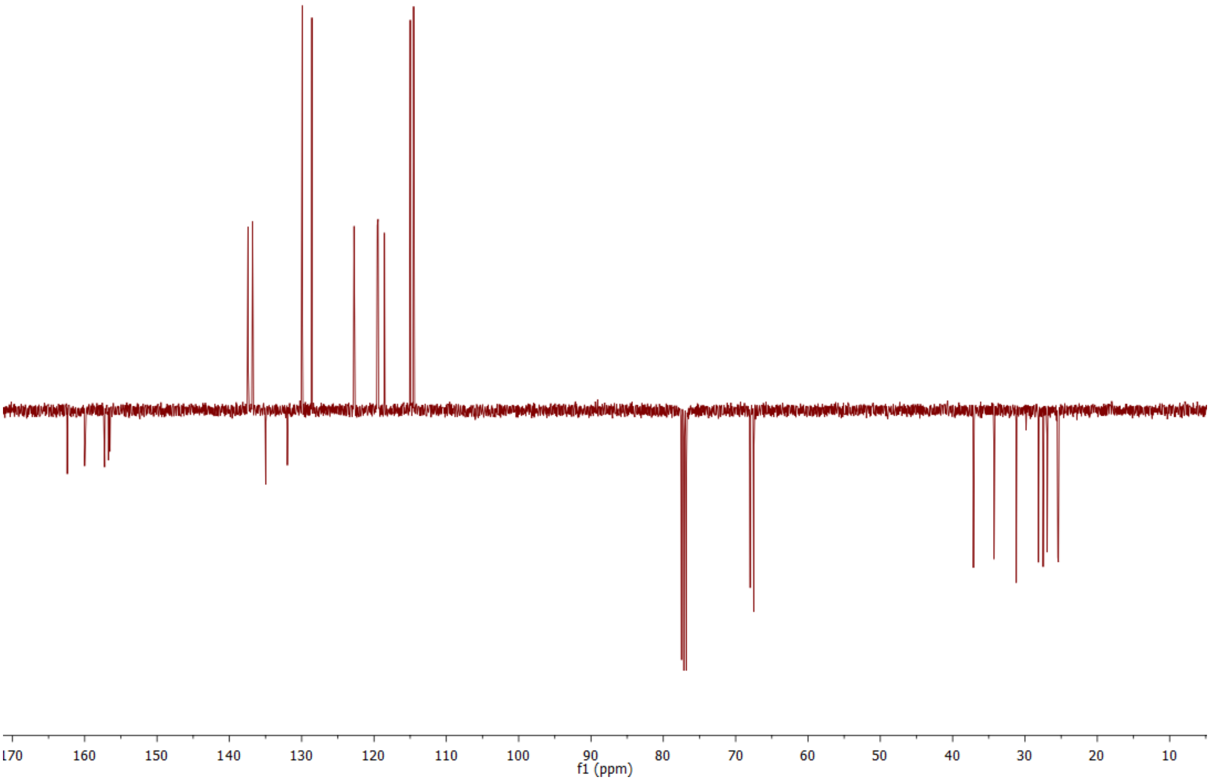

Figure S95 JMOD NMR ($CDCl_3$, 100 MHz) of 2g.

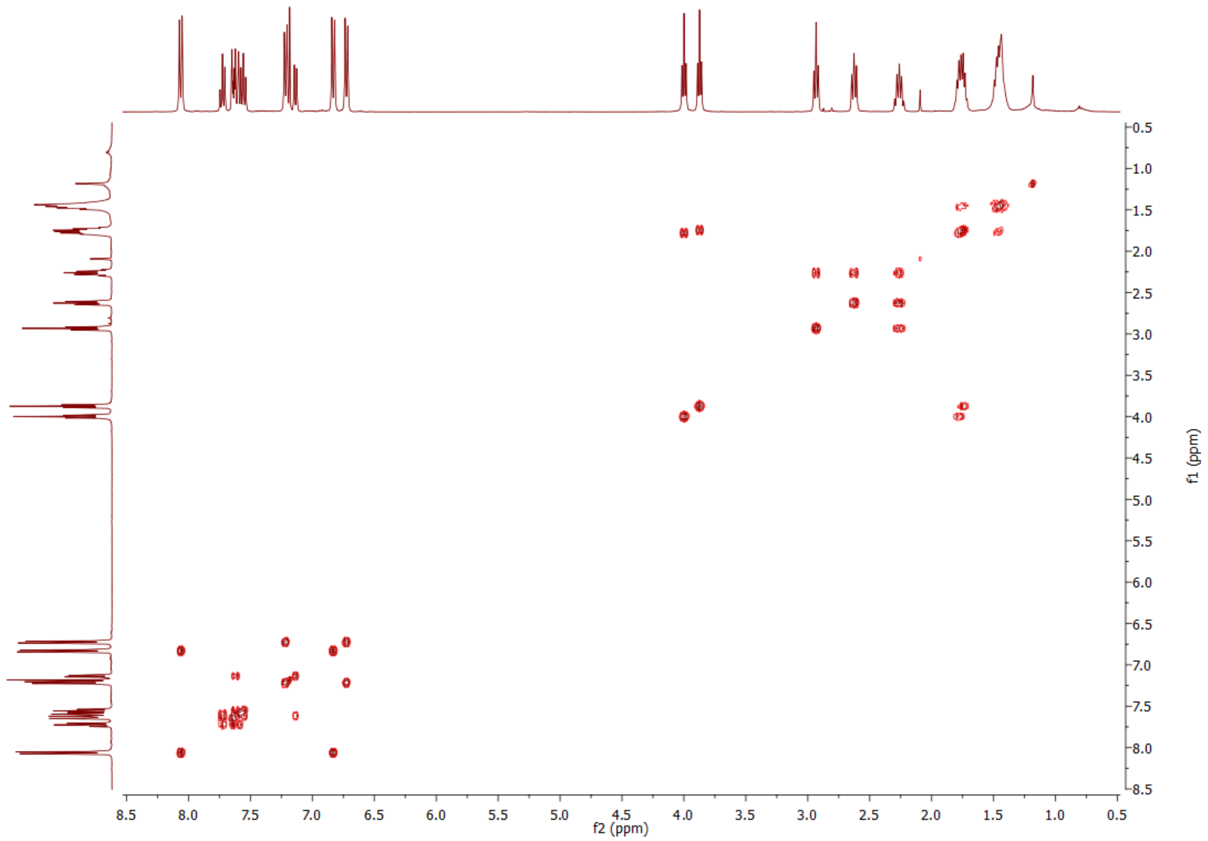

Figure S96 ^{1}H COSY NMR ($CDCl_3$, 400 MHz) of 2g.

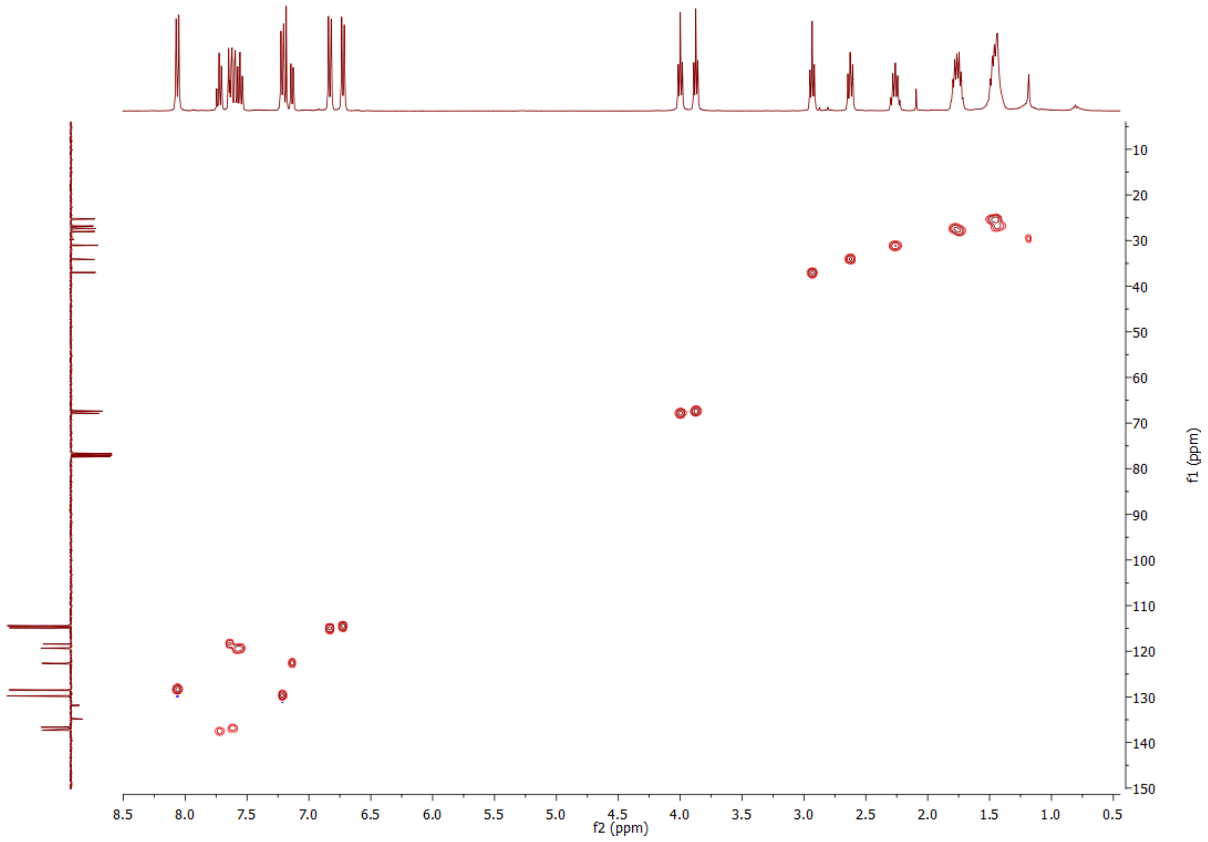

Figure S97 HSQC NMR ($CDCl_3$, 400 MHz) of 2g.

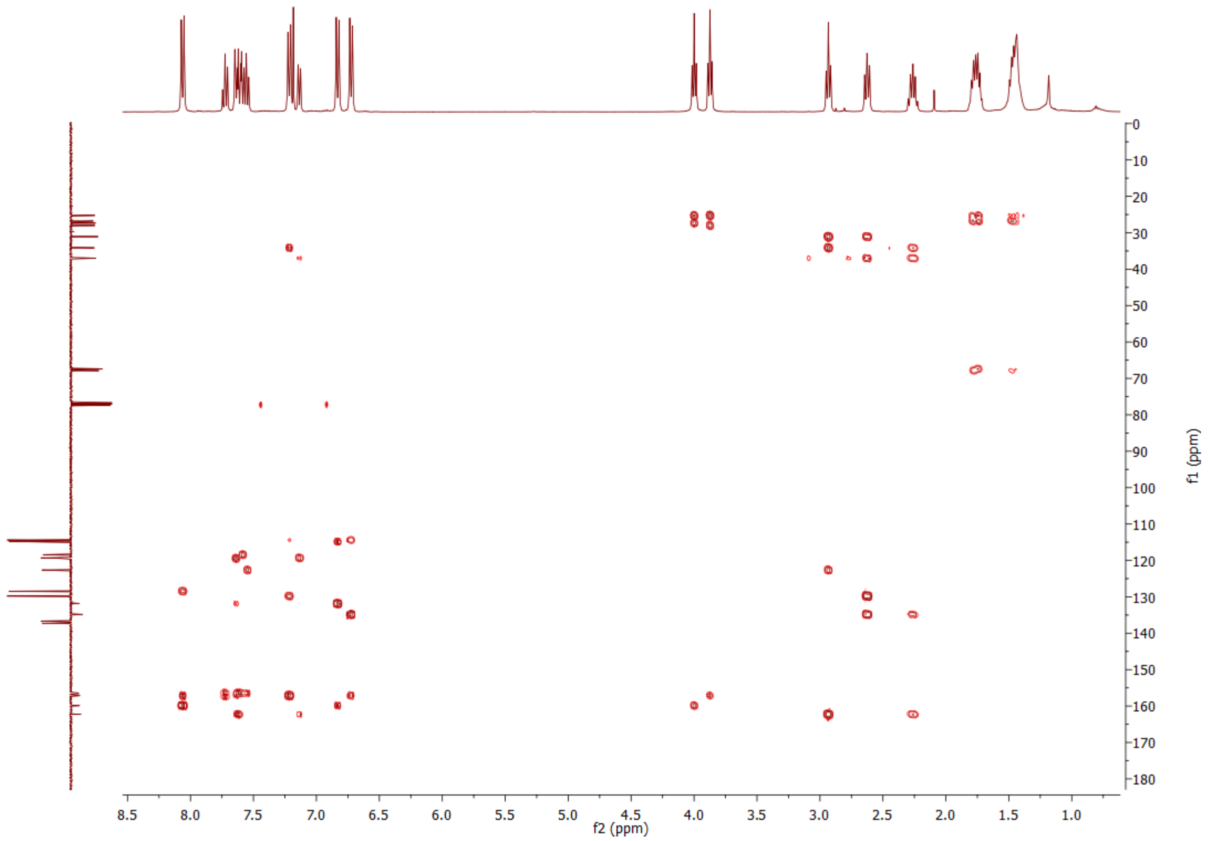

Figure S98 HMBC NMR ($CDCl_3$, 400 MHz) of 2g.

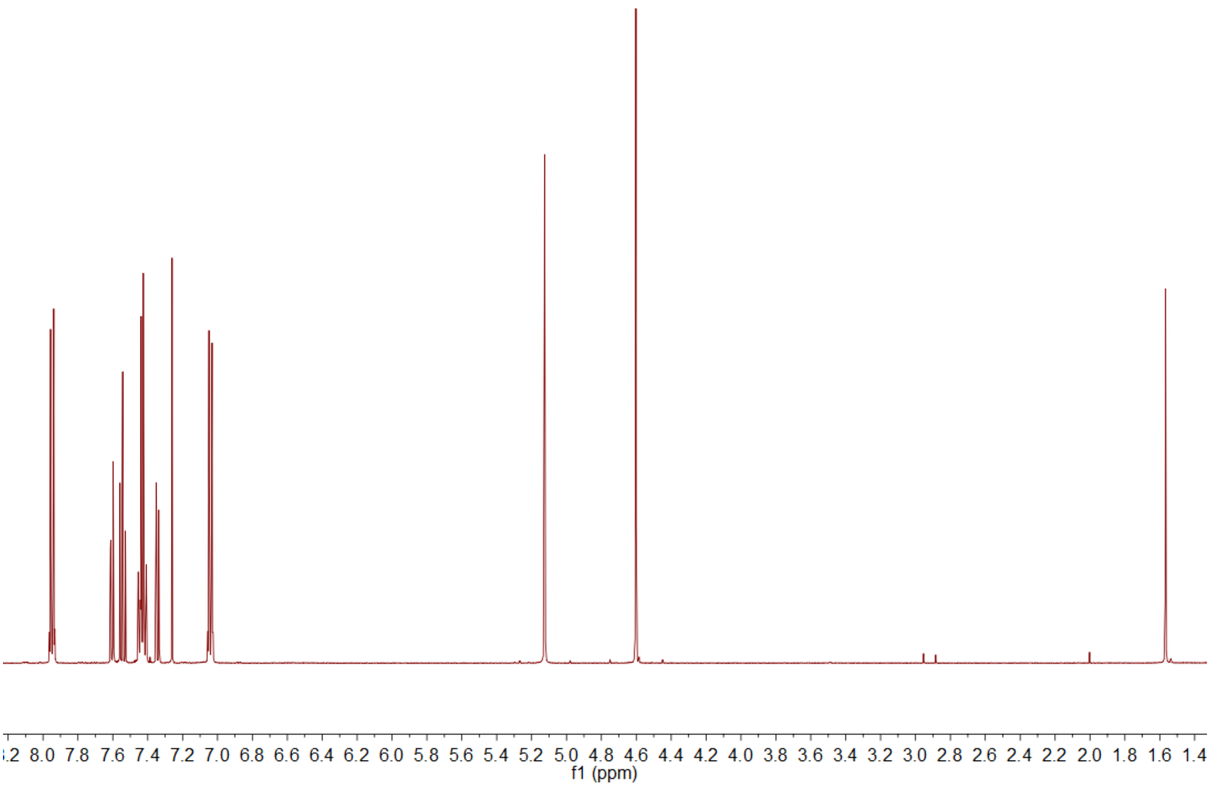

Figure S99 ^{1}H NMR ($CDCl_3$, 500 MHz) of S10.

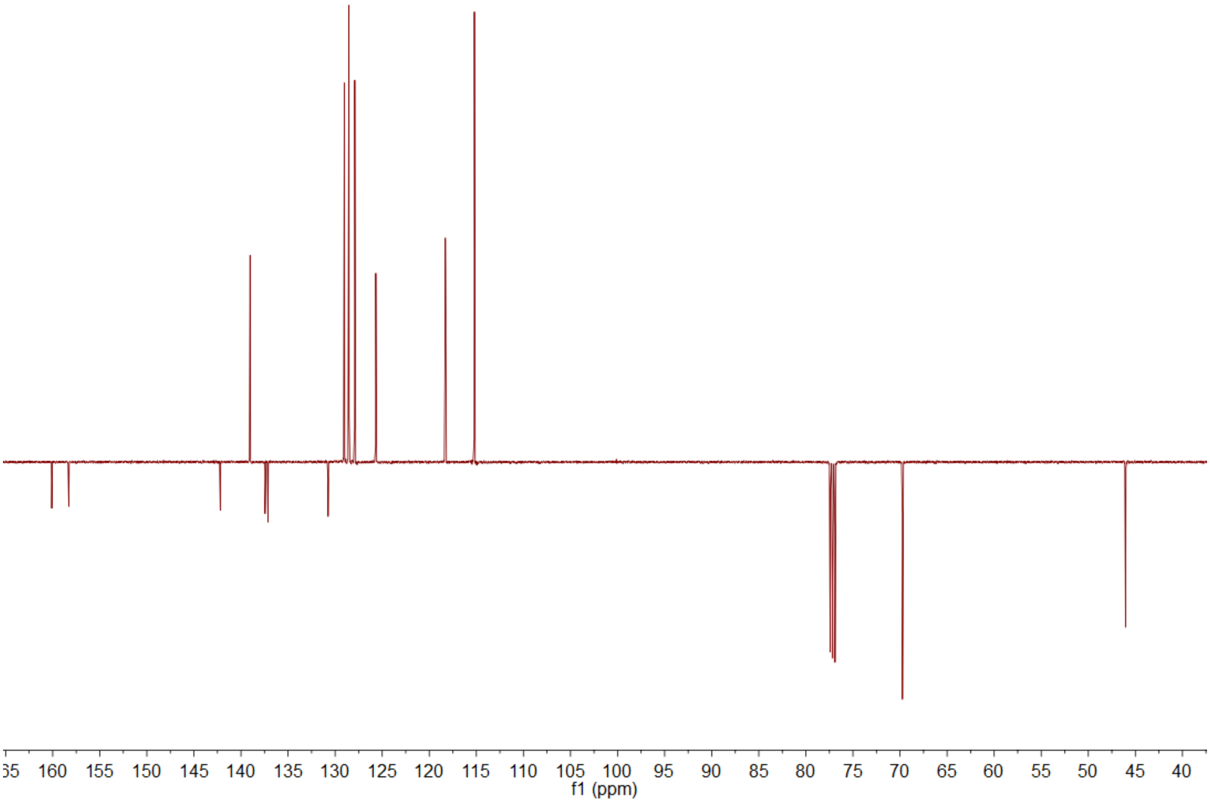

Figure S100 JMOD NMR ($CDCl_3$, 125 MHz) of S10.

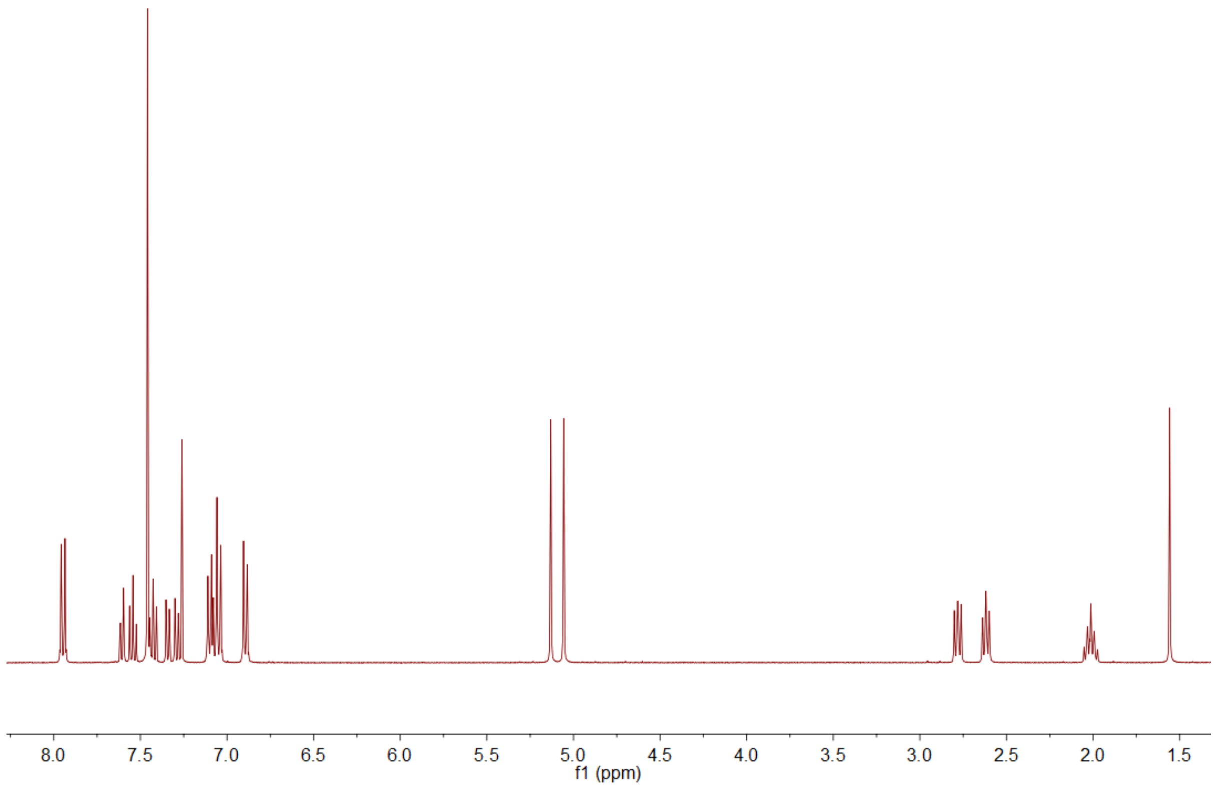

Figure S101 ^{1}H NMR ($CDCl_3$, 400 MHz) of 1h.

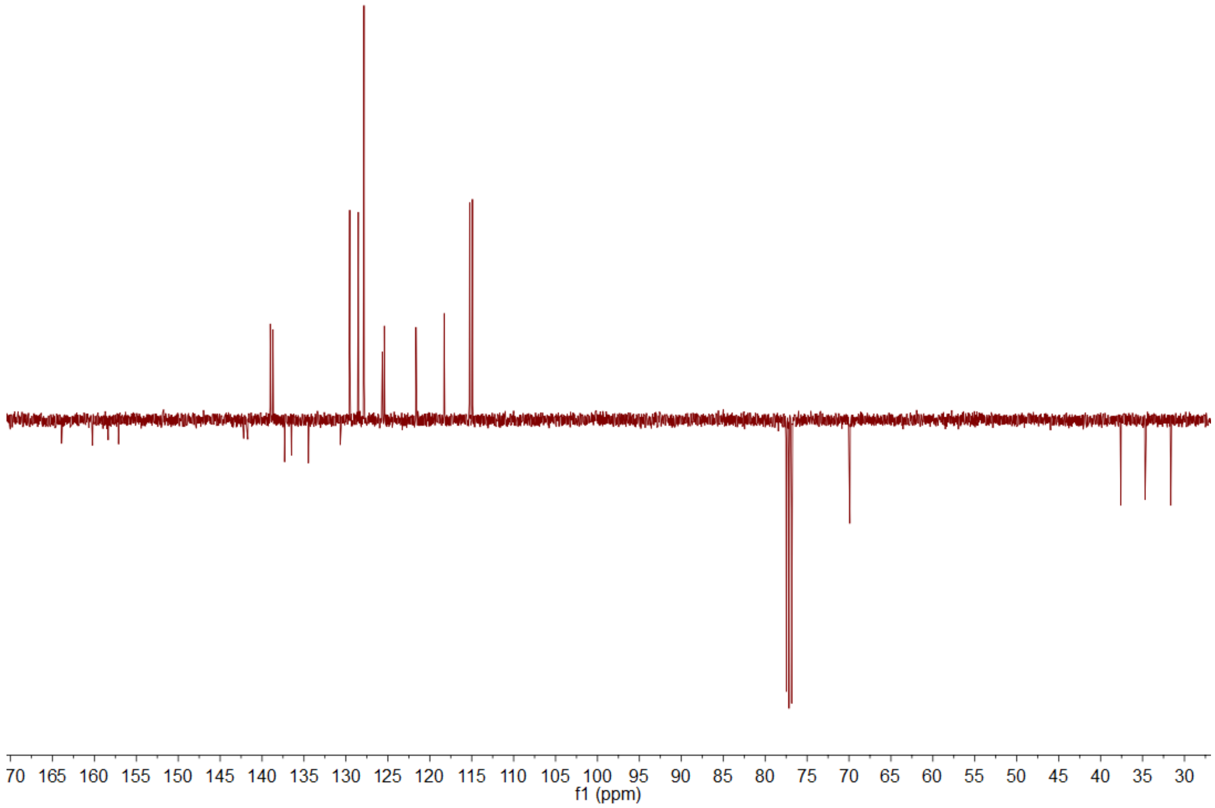

Figure S102 JMOD NMR ($CDCl_3$, 100 MHz) of 1h.

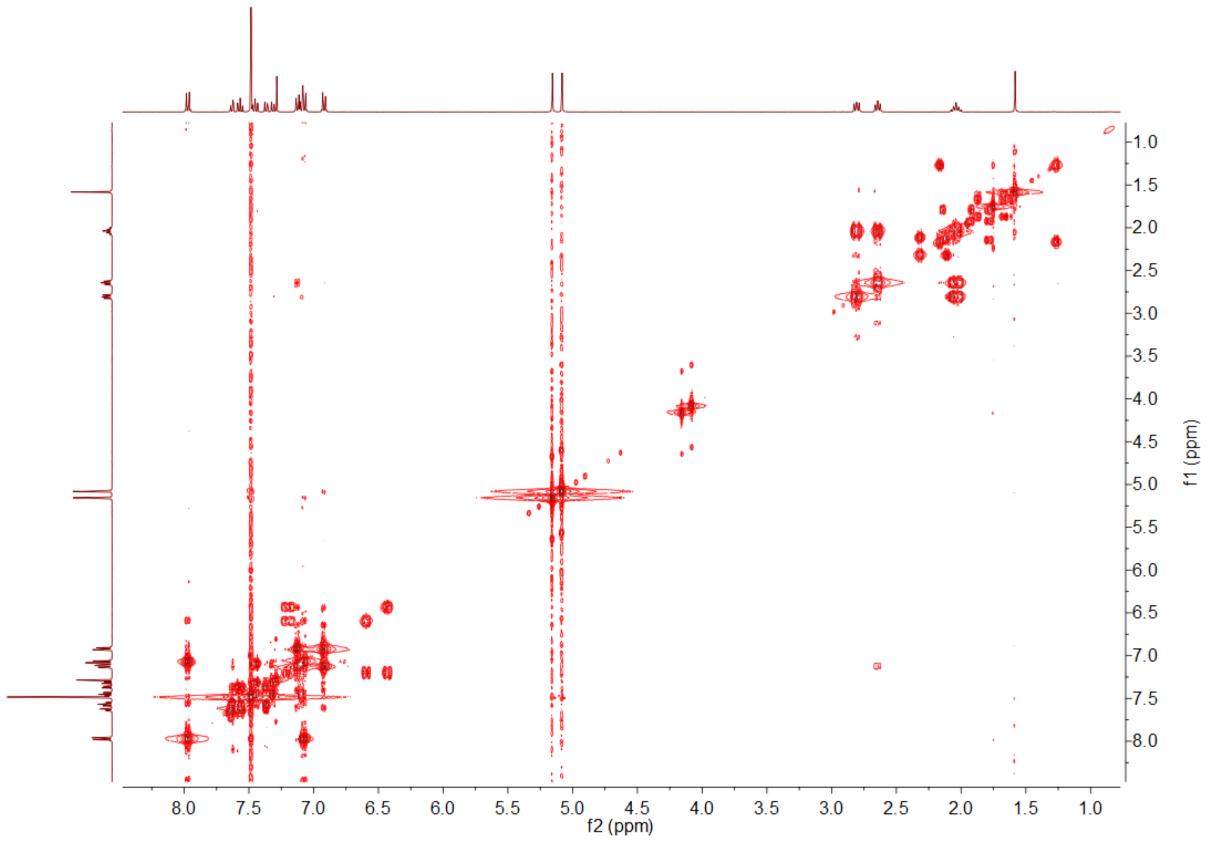

Figure S103 ^{1}H COSY NMR ($CDCl_3$, 400 MHz) of 1h.

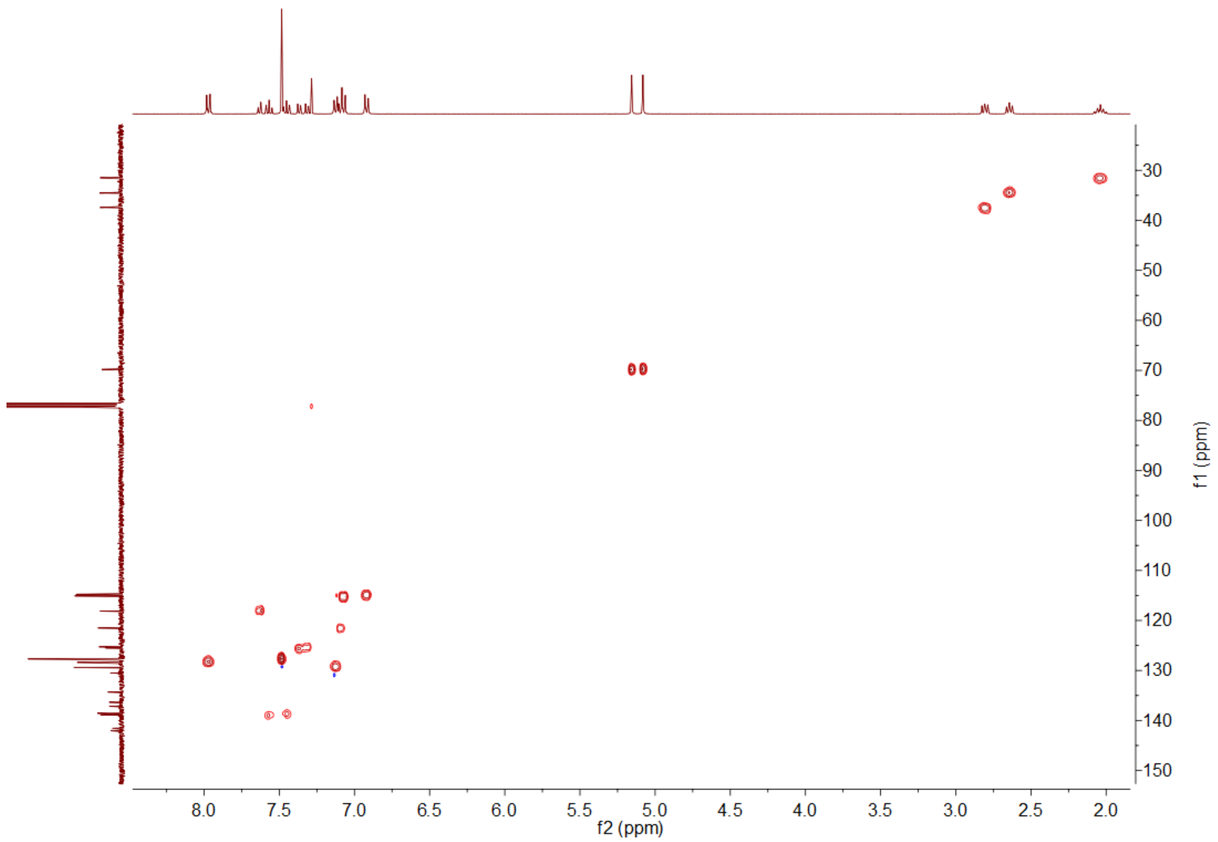

Figure S104 HSQC NMR ($CDCl_3$, 400 MHz) of 1h.

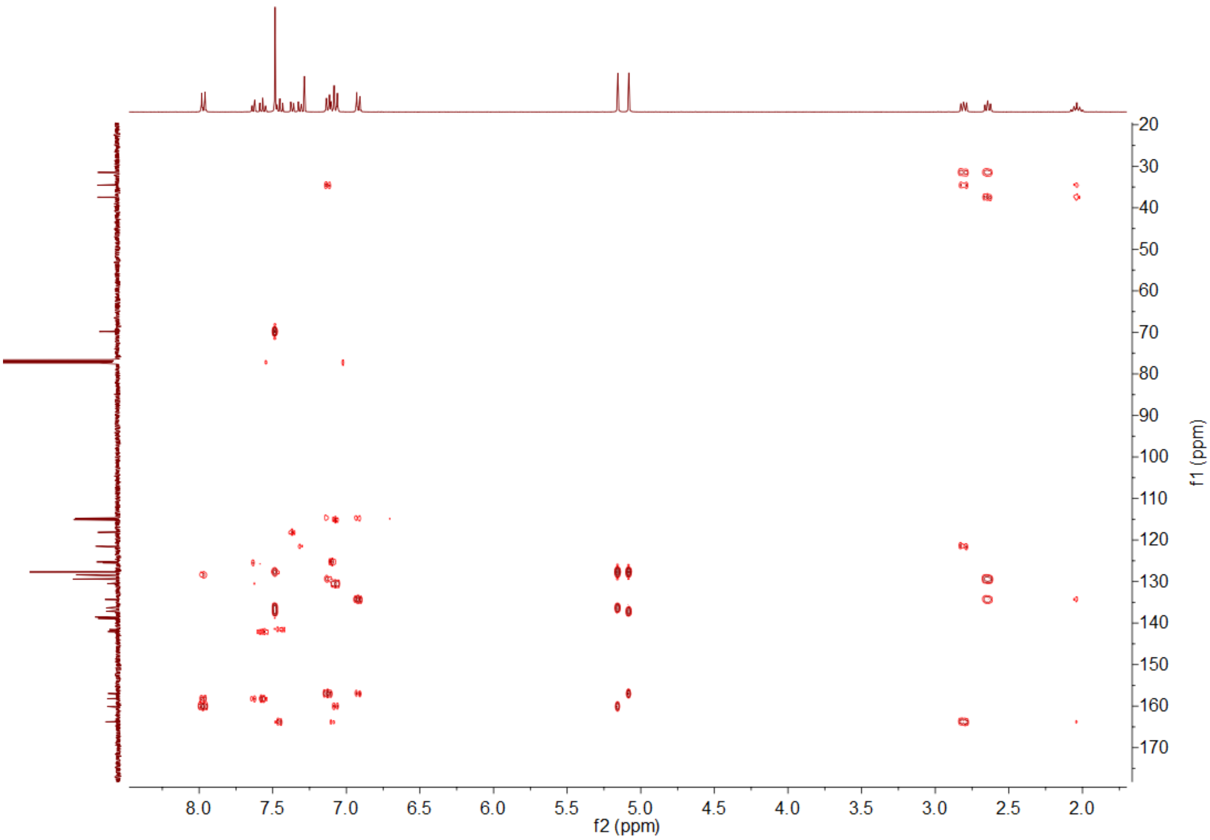

Figure S105 HMBC NMR ($CDCl_3$, 400 MHz) of 1h.

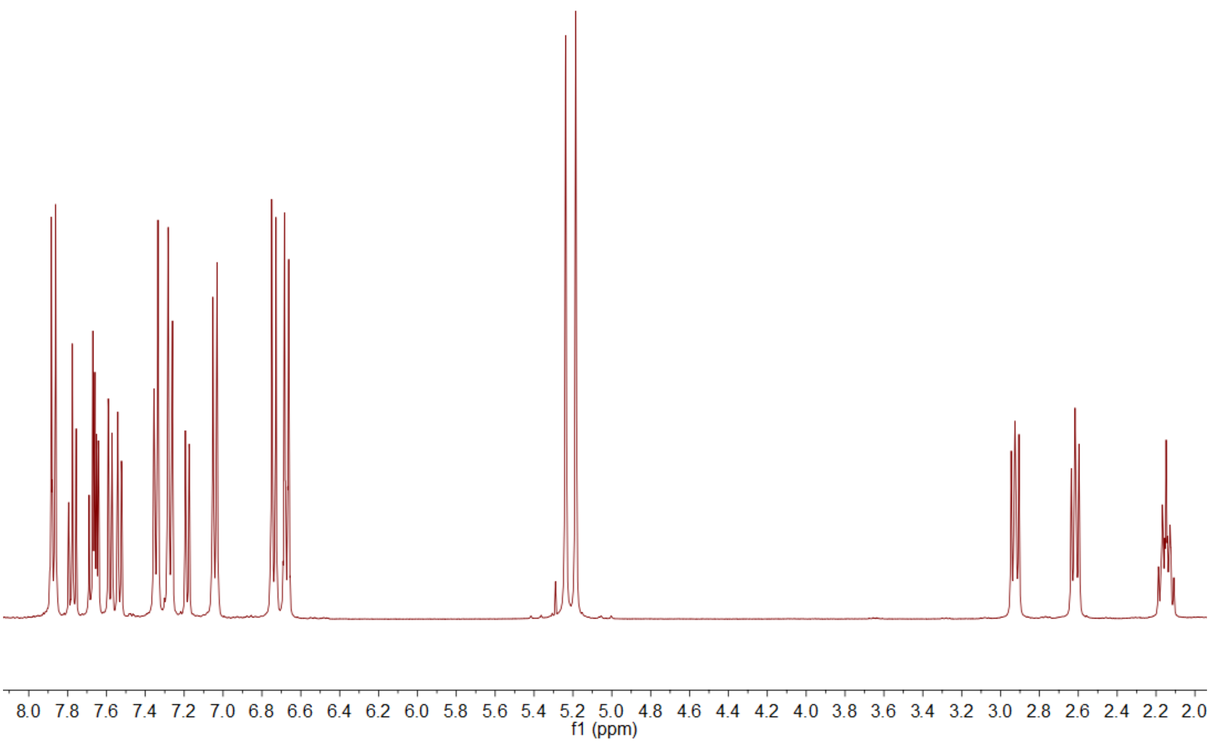

Figure S106 ^{1}H NMR ($CDCl_3$, 400 MHz) of 2h.

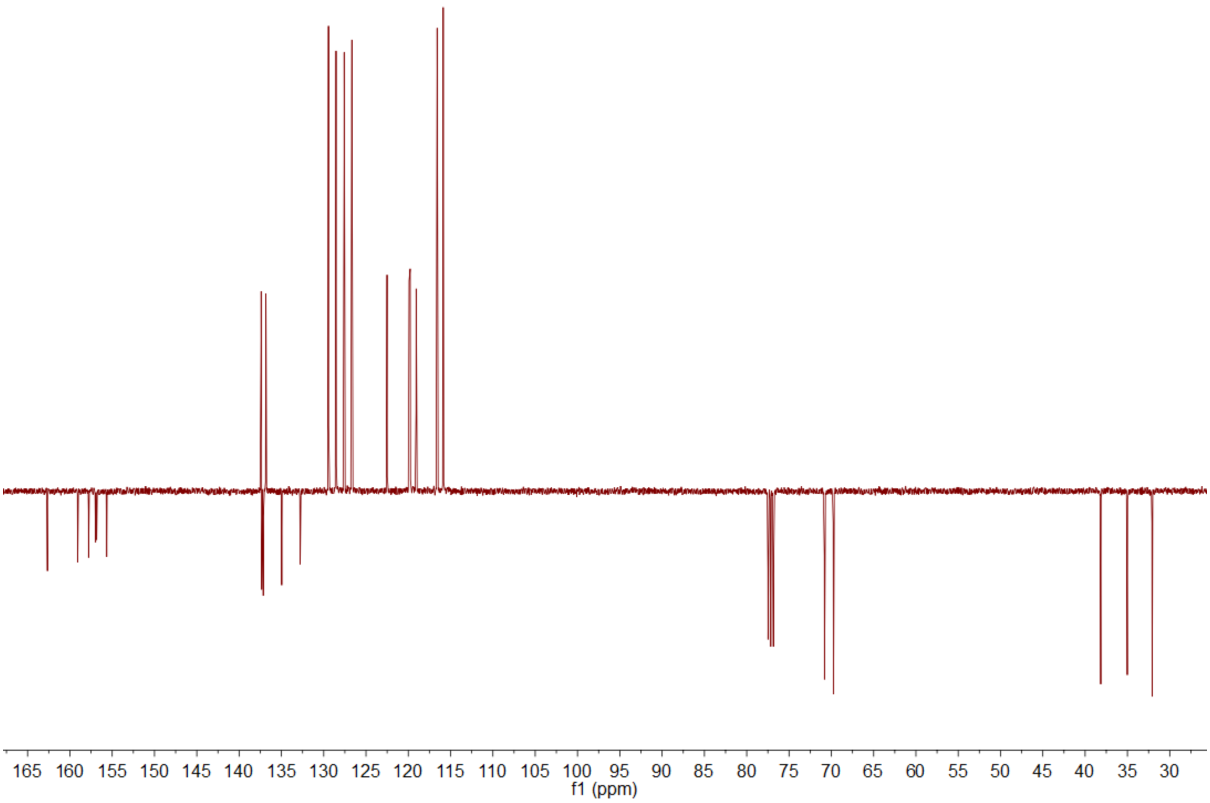

Figure S107 JMOD NMR ($CDCl_3$, 100 MHz) of 2h.

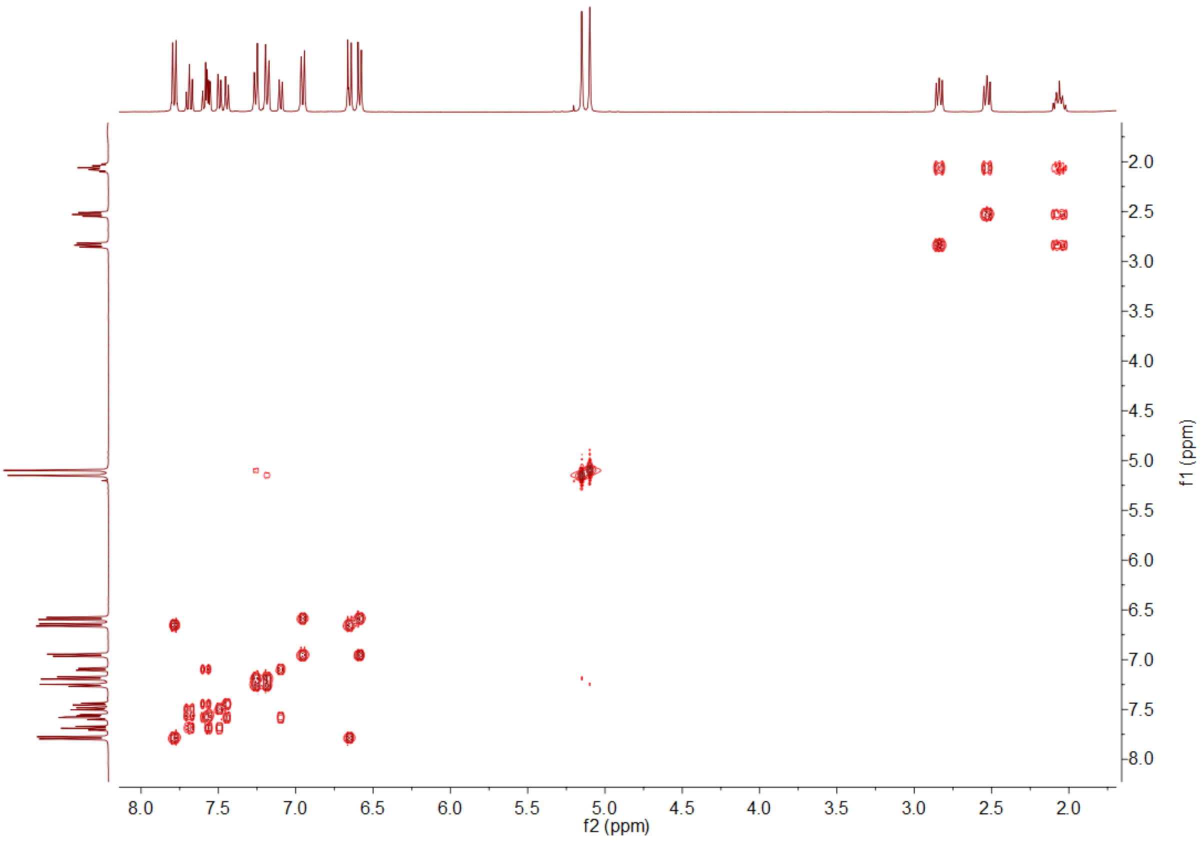

Figure S108 ^{1}H COSY NMR ($CDCl_3$, 400 MHz) of 2h.

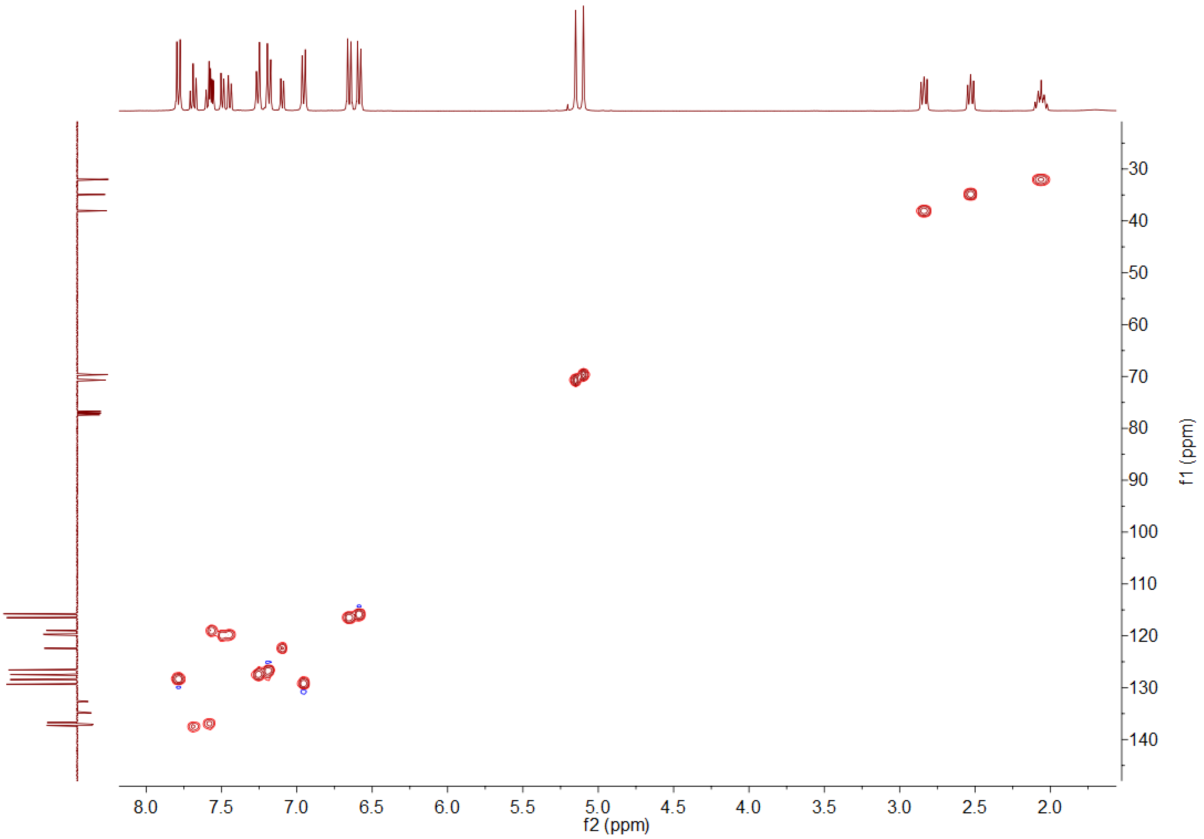

Figure S109 HSQC NMR ($CDCl_3$, 400 MHz) of 2h.

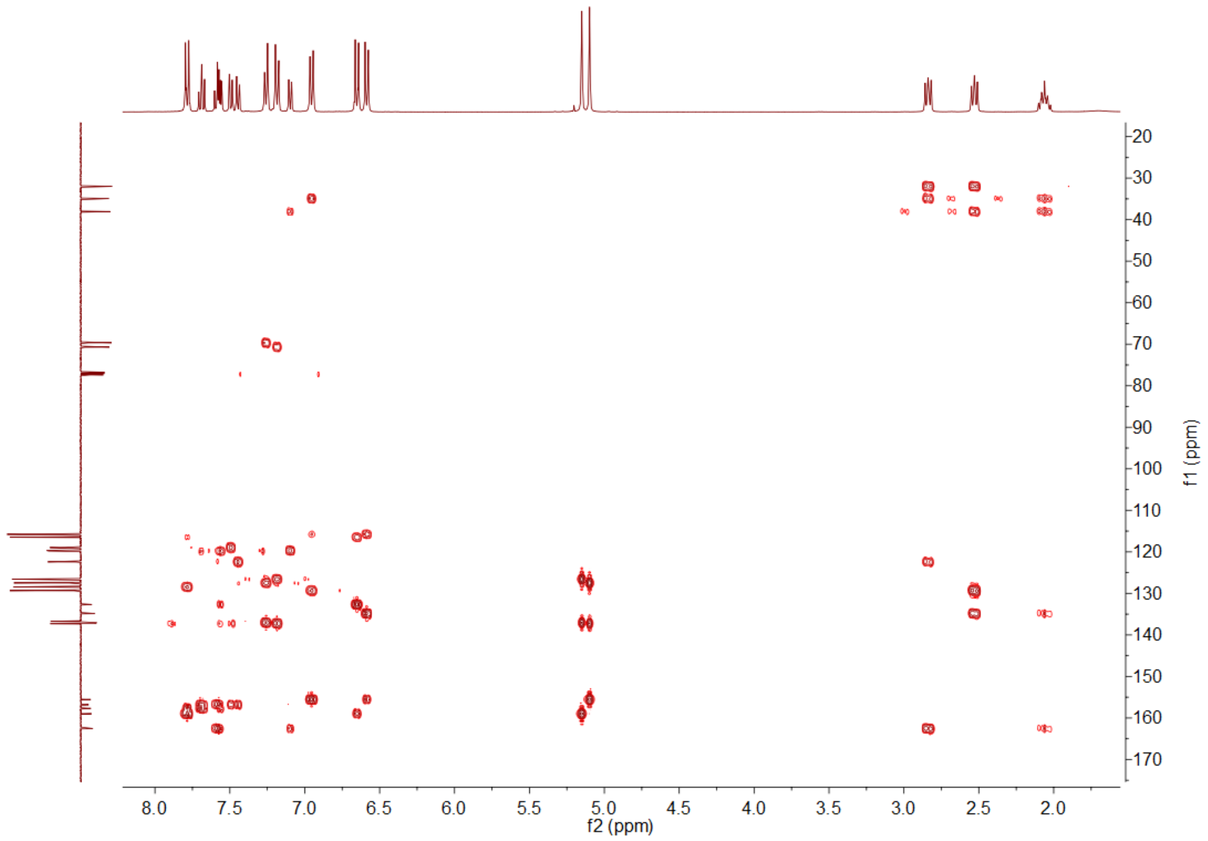

Figure S110 HMBC NMR ($CDCl_3$, 400 MHz) of 2h.

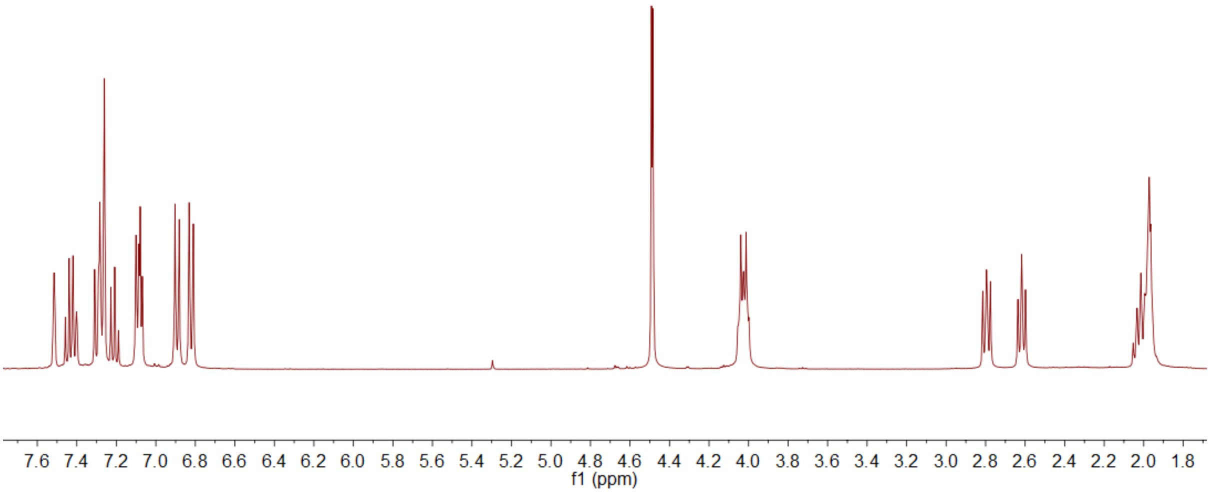

Figure S111 ^{1}H NMR ($CDCl_3$, 400 MHz) of $1i^{Br}$.

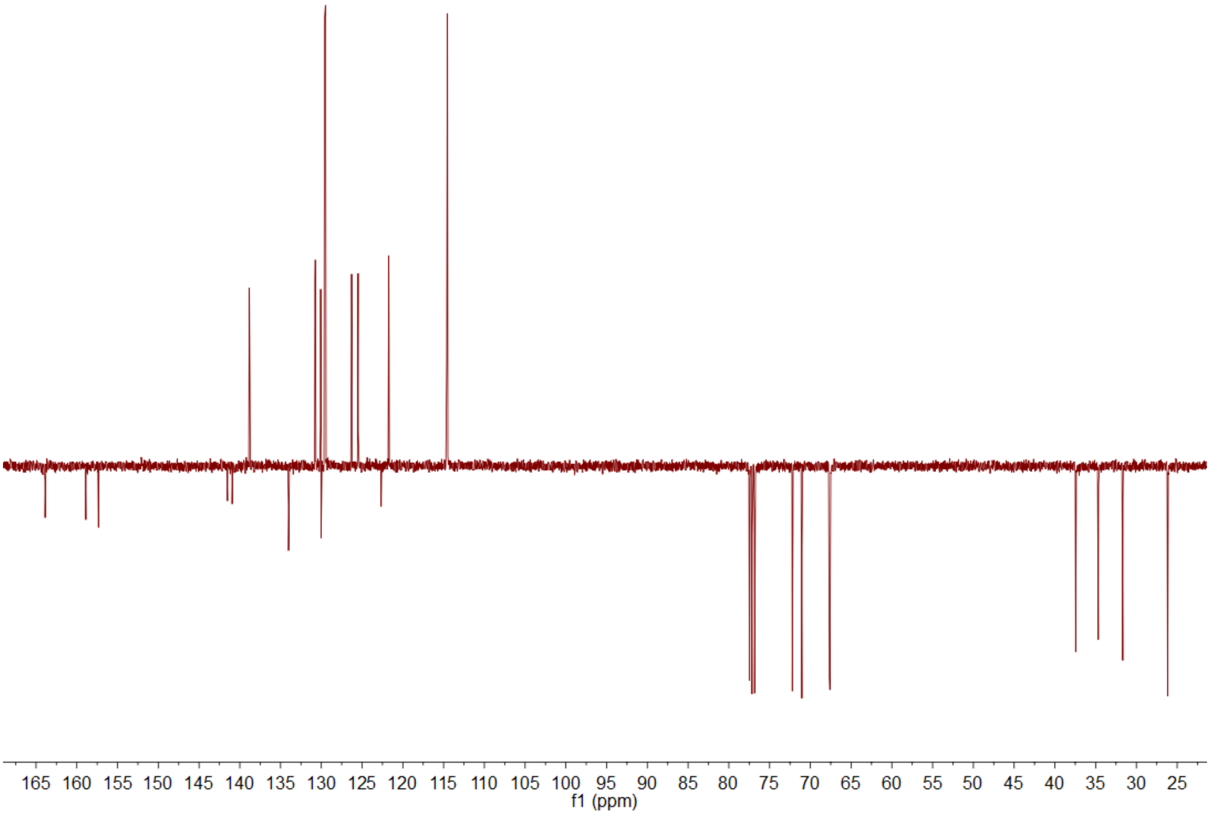

Figure S112 JMOD NMR ($CDCl_3$, 100 MHz) of $1i^{Br}$.

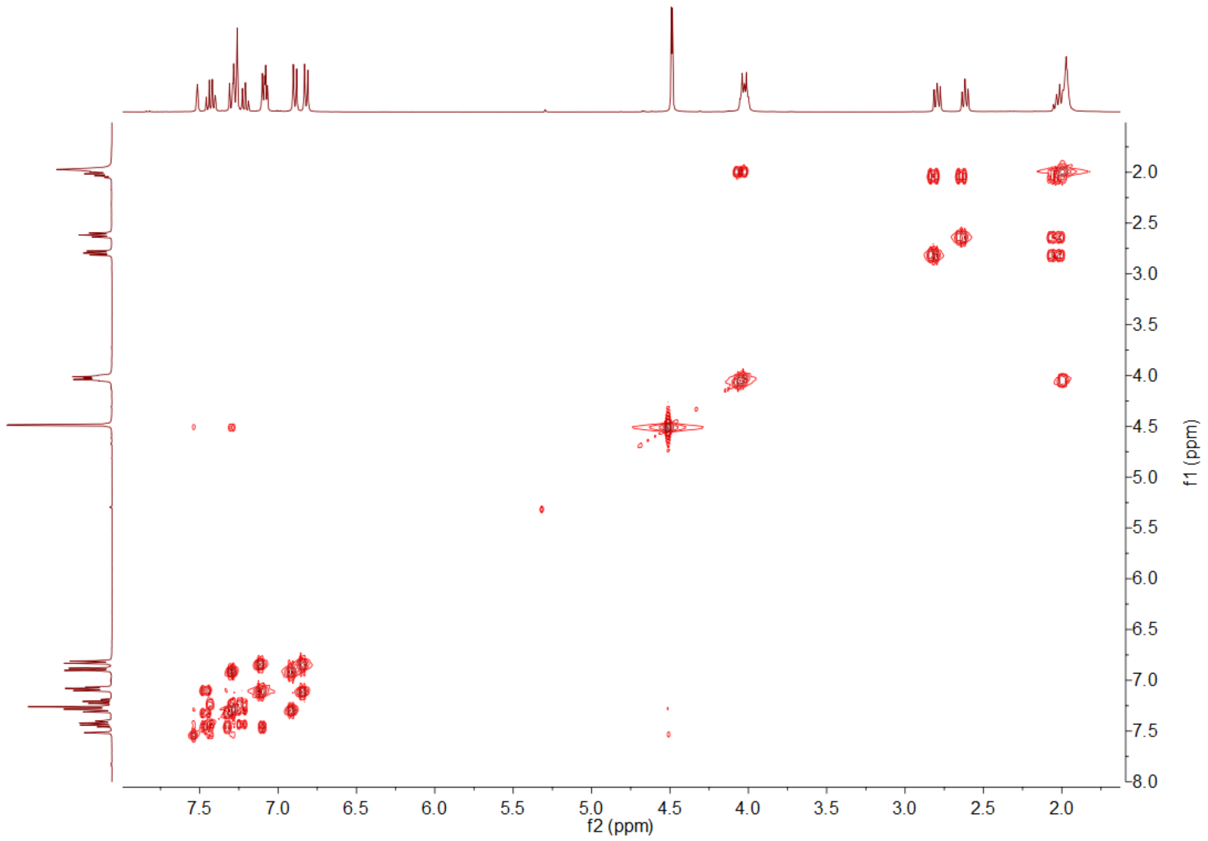

Figure S113 ^{1}H COSY NMR ($CDCl_3$, 400 MHz) of $1i^{Br}$.

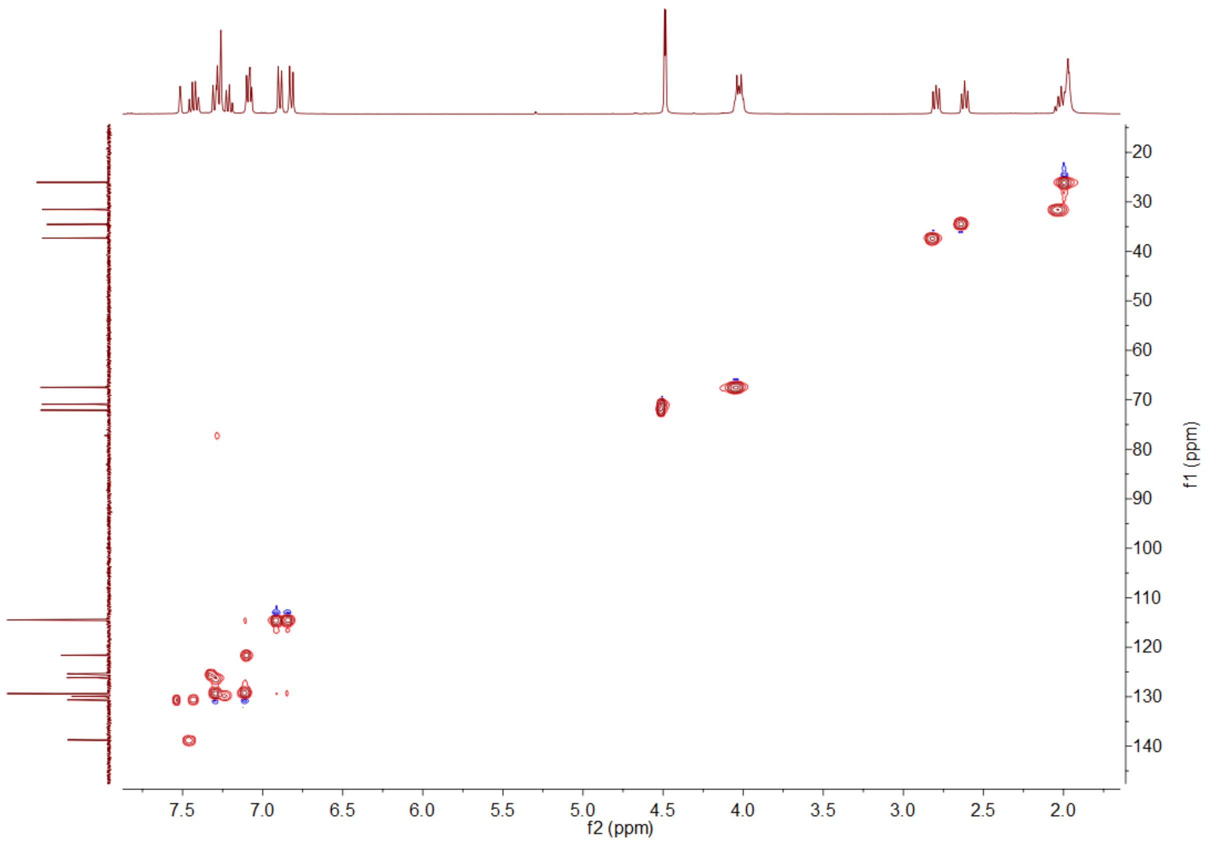

Figure S114 HSQC NMR ($CDCl_3$, 400 MHz) of $1i^{Br}$.

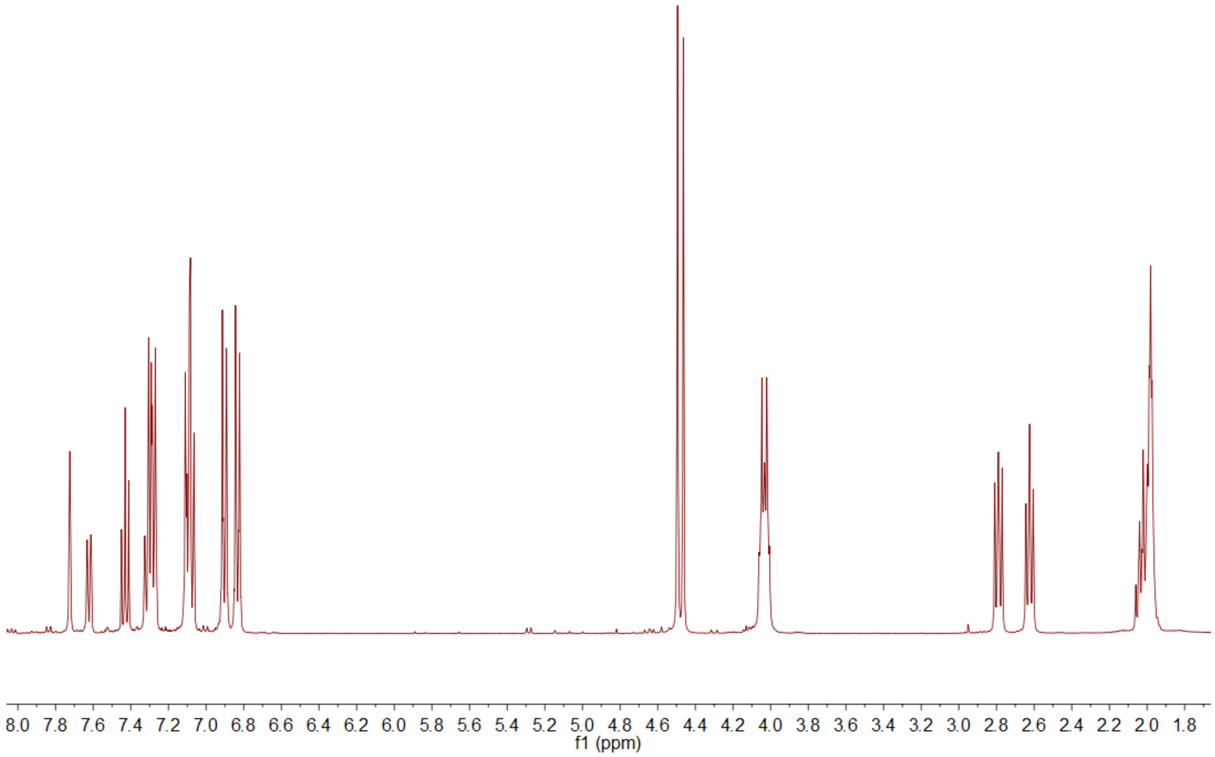

Figure S115 ^{1}H NMR ($CDCl_3$, 400 MHz) of 1i^{I}.

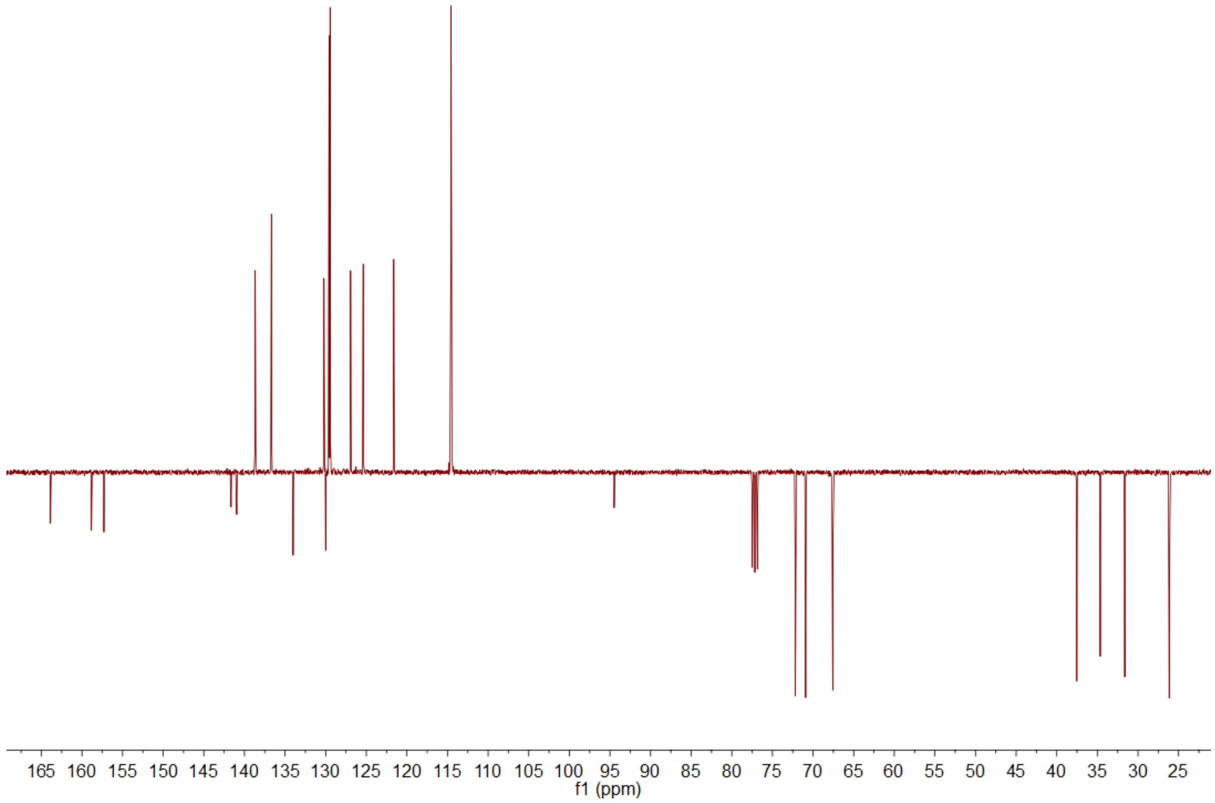

Figure S116 JMOD NMR ($CDCl_3$, 100 MHz) of 1i^{I}.

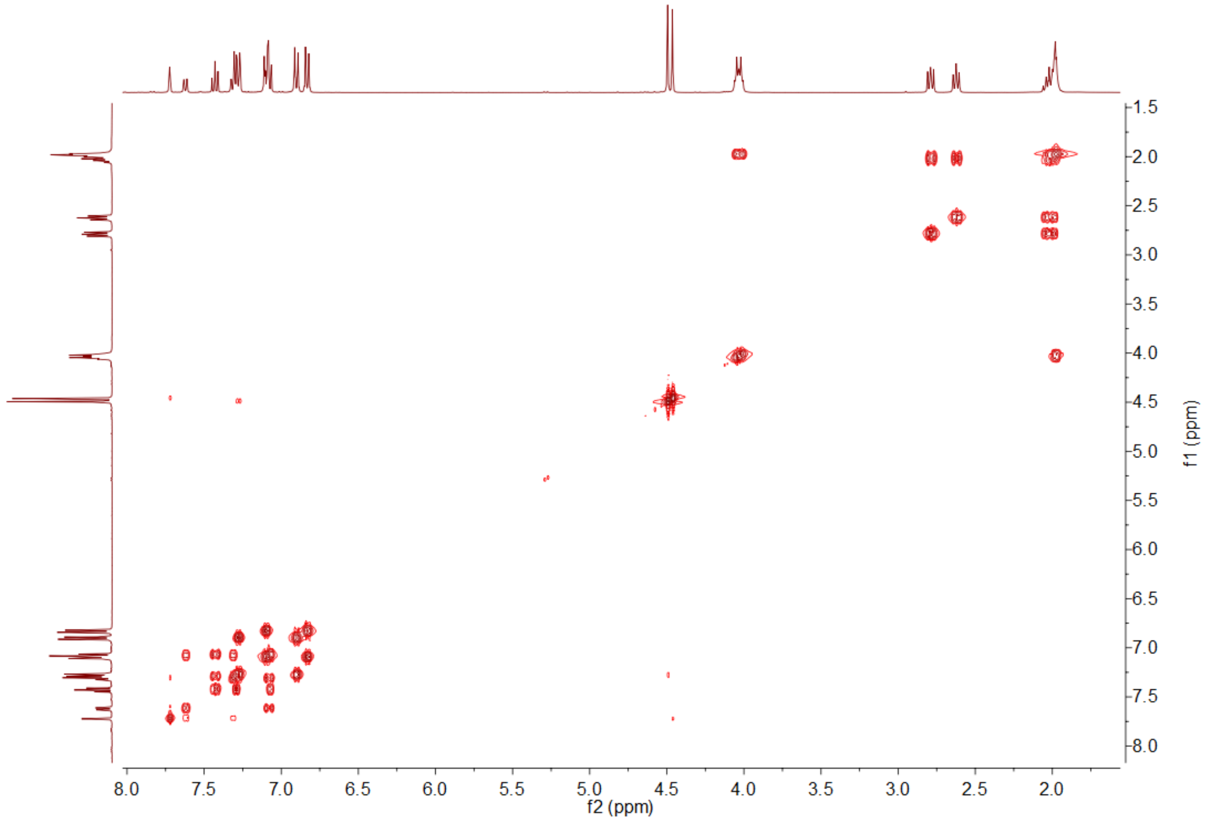

Figure S117 ^{1}H COSY NMR ($CDCl_3$, 400 MHz) of 1i^{I}.

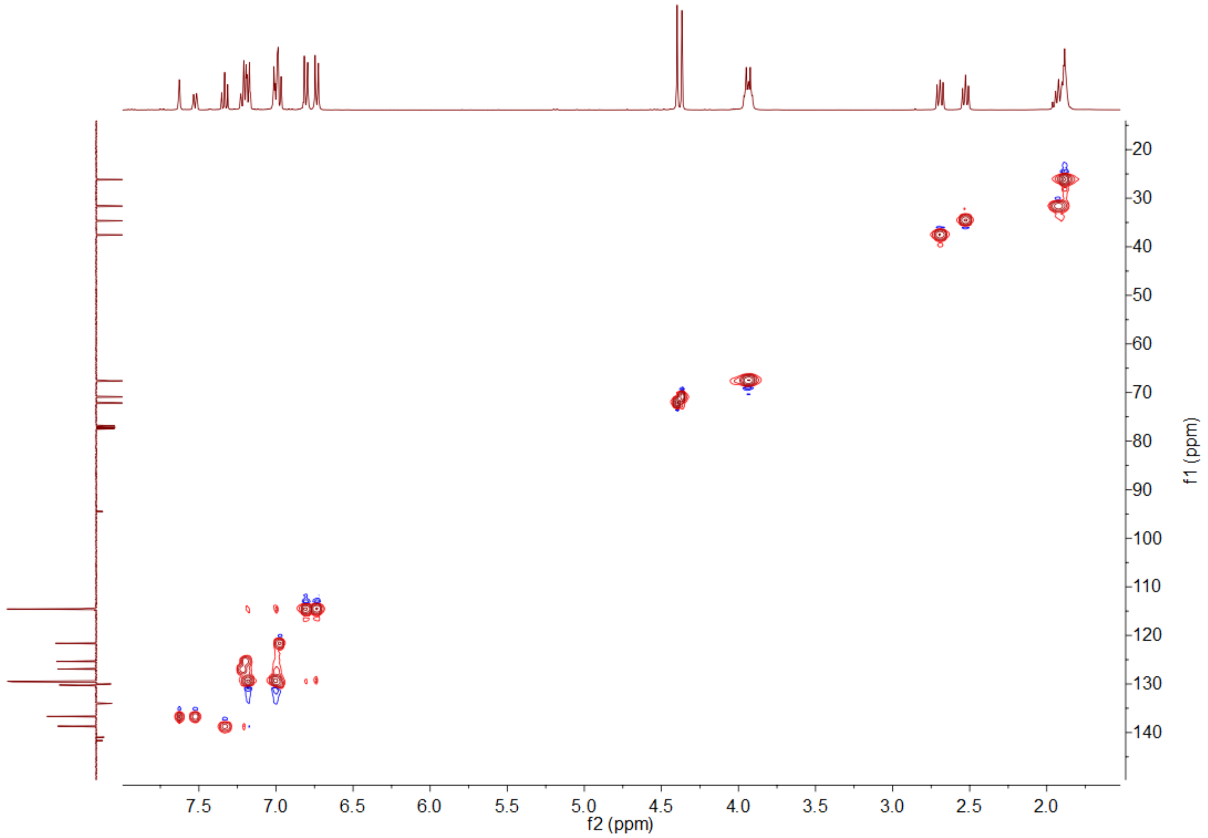

Figure S118 HSQC NMR ($CDCl_3$, 400 MHz) of 1i^{I}.

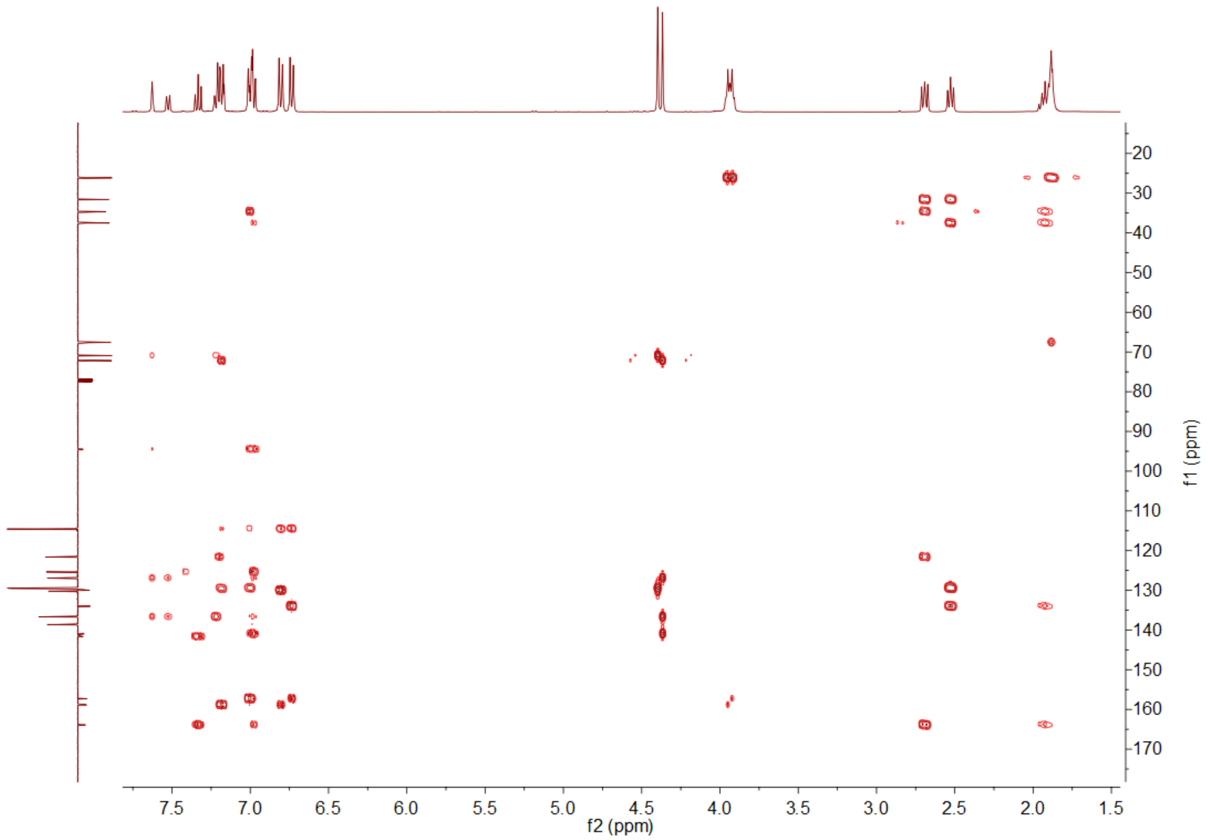

Figure S119 HMBC NMR ($CDCl_3$, 400 MHz) of 1i[I].

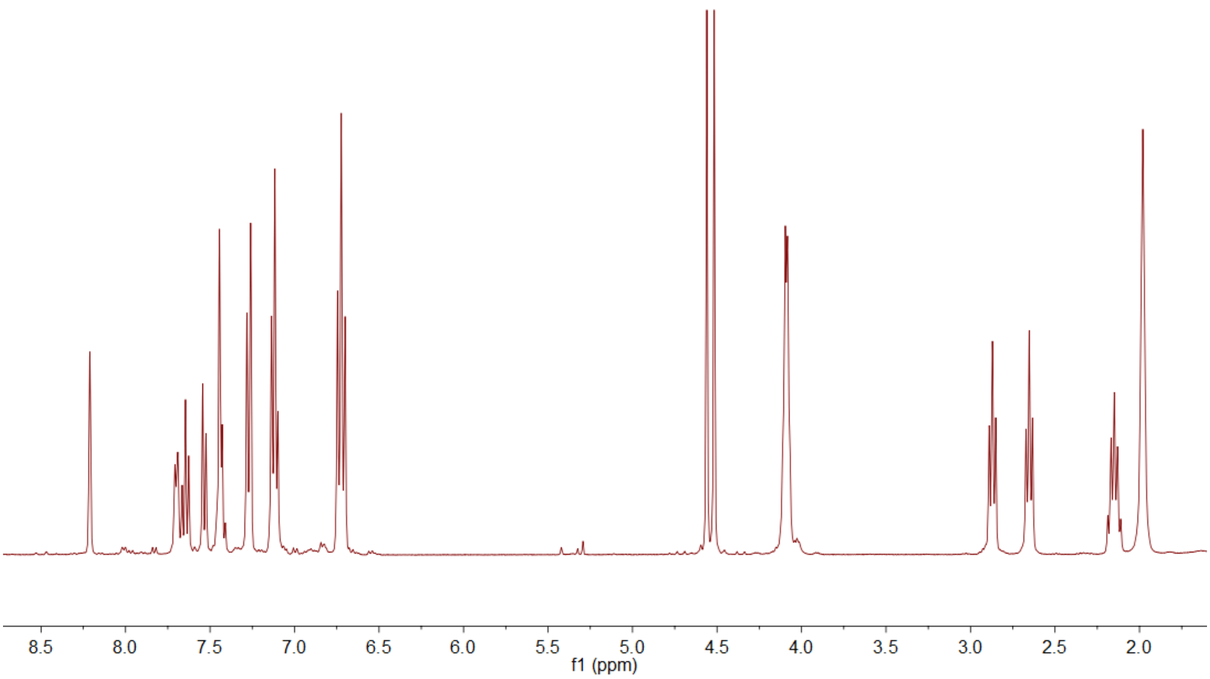

Figure S120 ^{1}H NMR ($CDCl_3$, 400 MHz) of 2i.

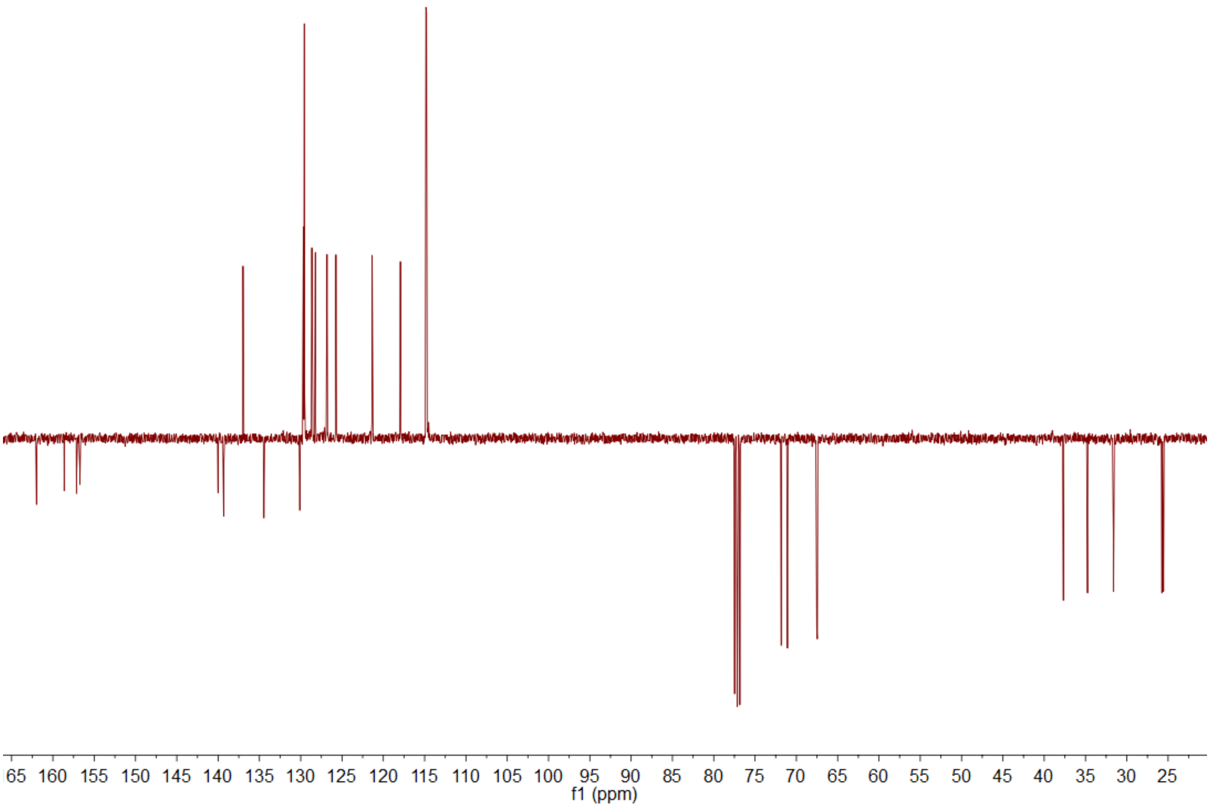

Figure S121 JMOD NMR ($CDCl_3$, 100 MHz) of 2i.

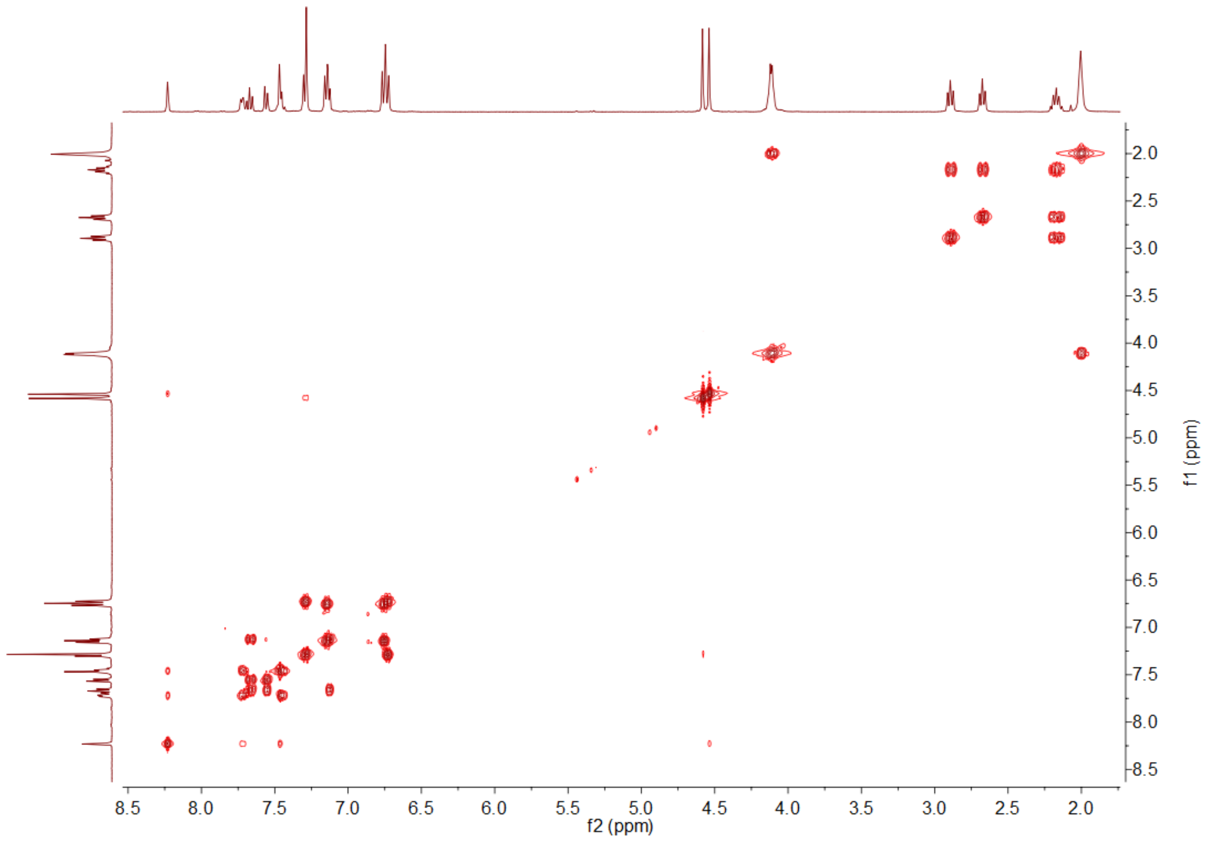

Figure S122 ^{1}H COSY NMR ($CDCl_3$, 400 MHz) of 2i.

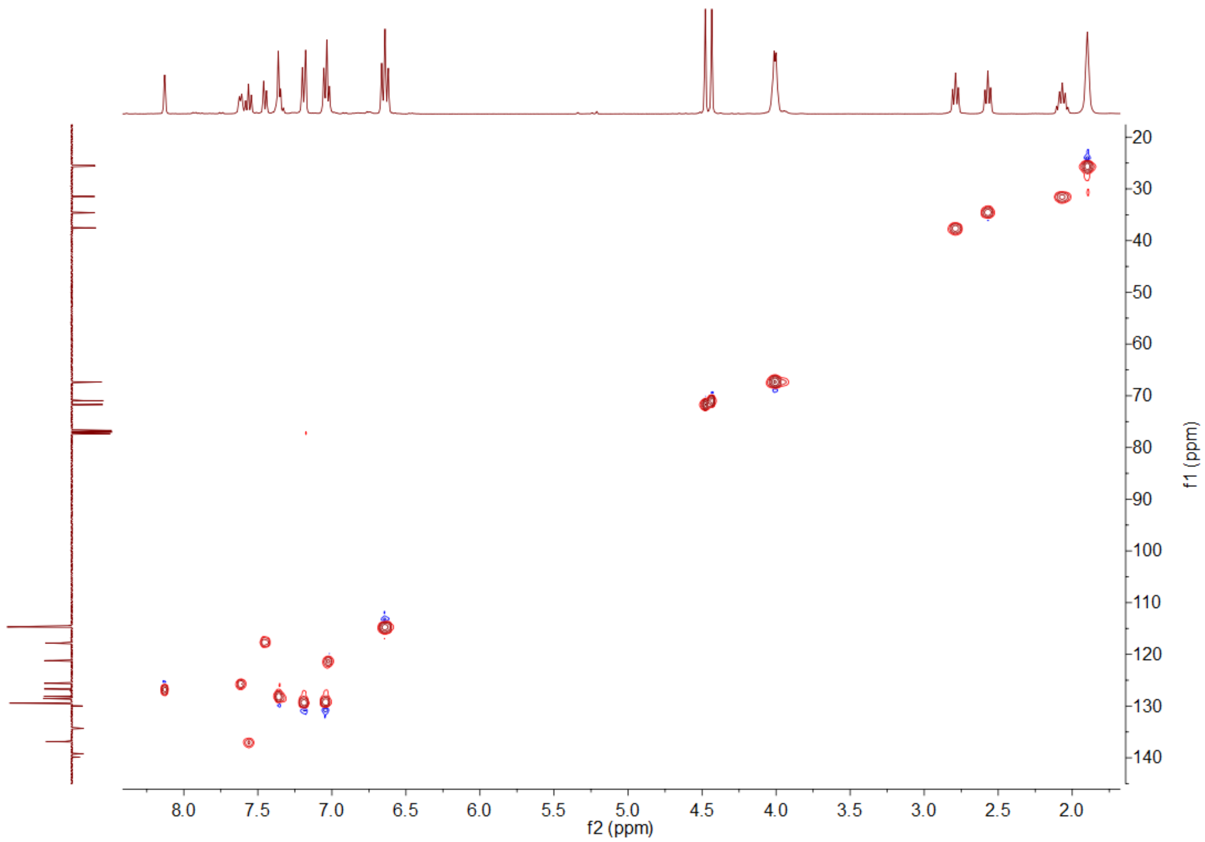

Figure S123 HSQC NMR ($CDCl_3$, 400 MHz) of 2i.

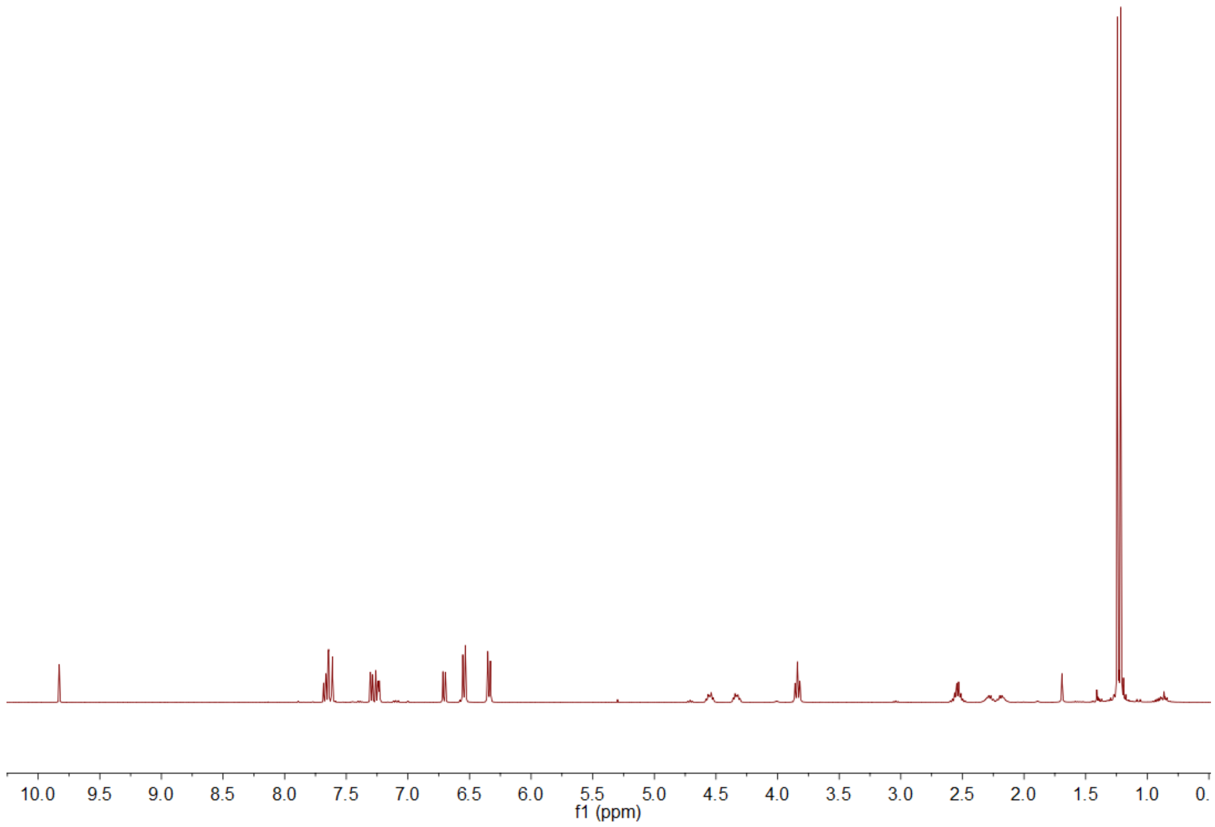

Figure S124 ^{1}H NMR ($CDCl_3$, 400 MHz) of S11.

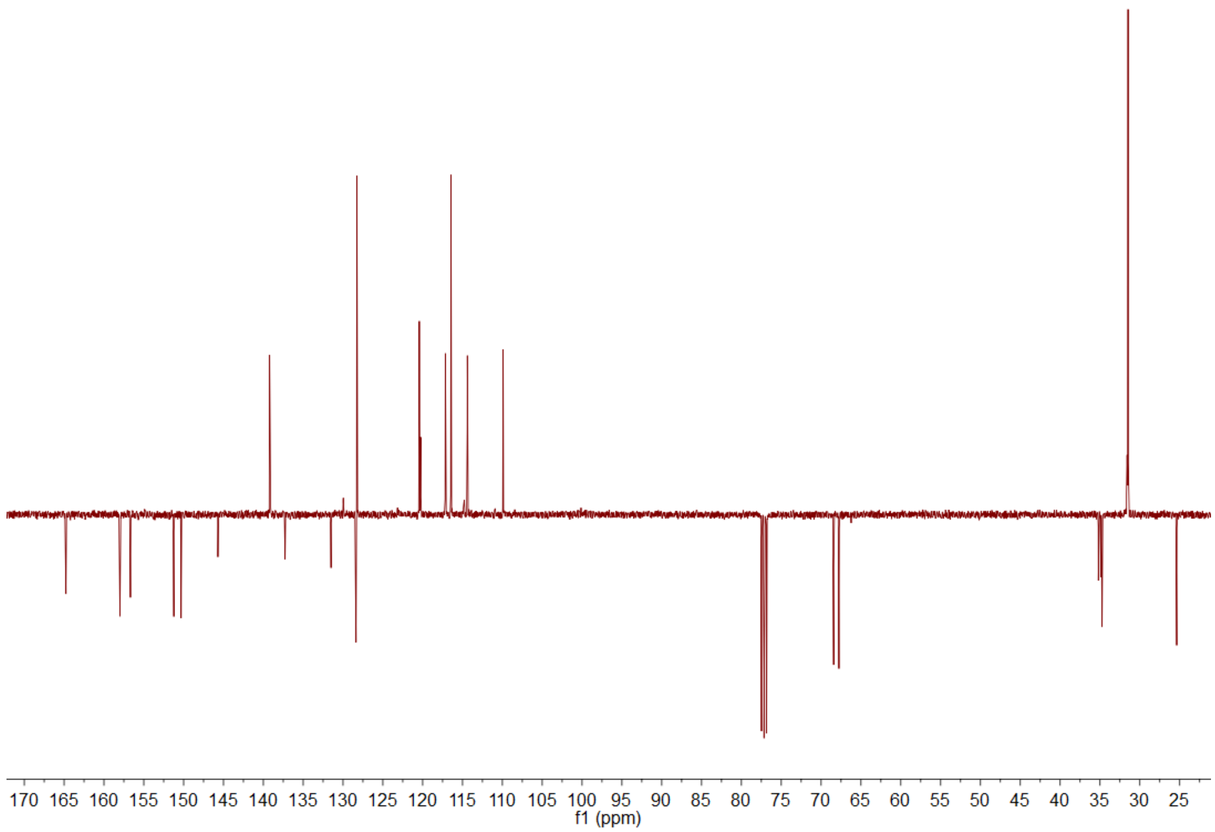

Figure S125 JMOD NMR ($CDCl_3$, 100 MHz) of S11.

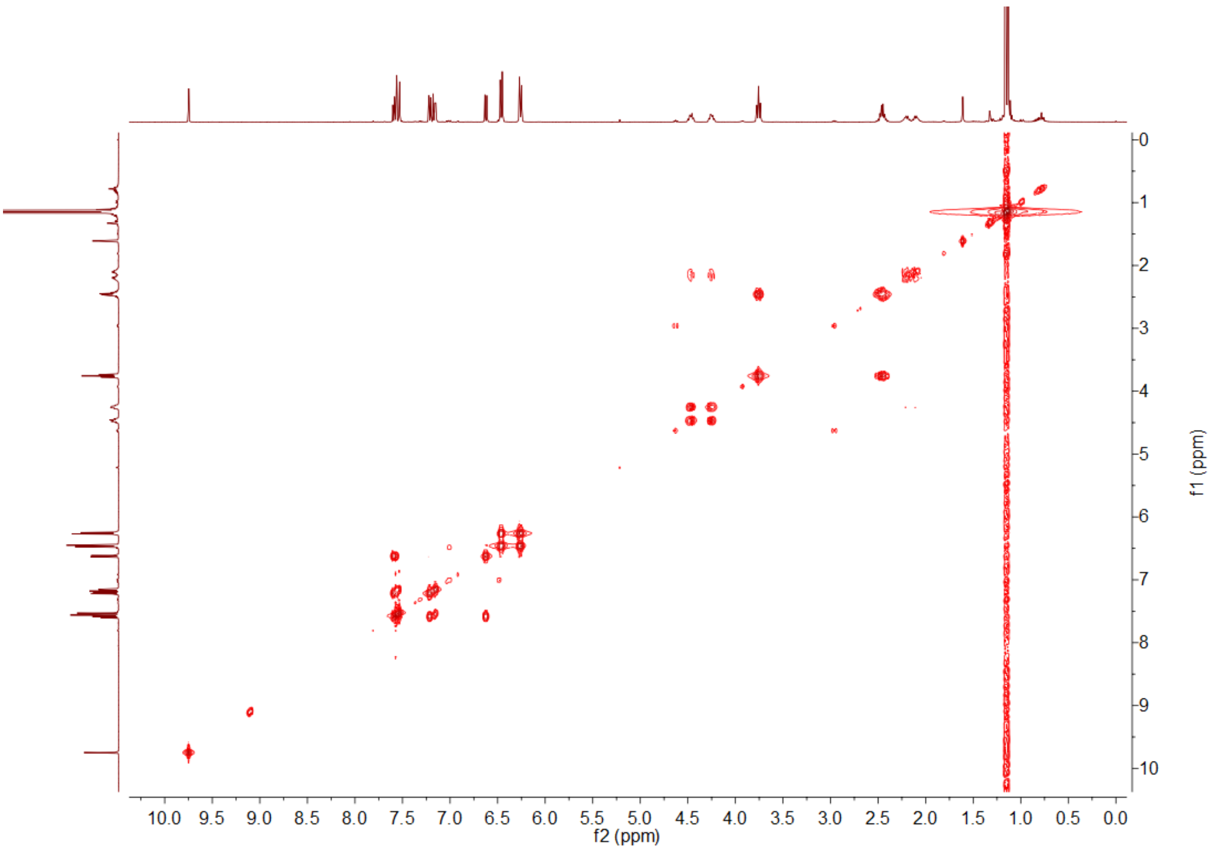

Figure S126 ^{1}H COSY NMR ($CDCl_3$, 400 MHz) of S11.

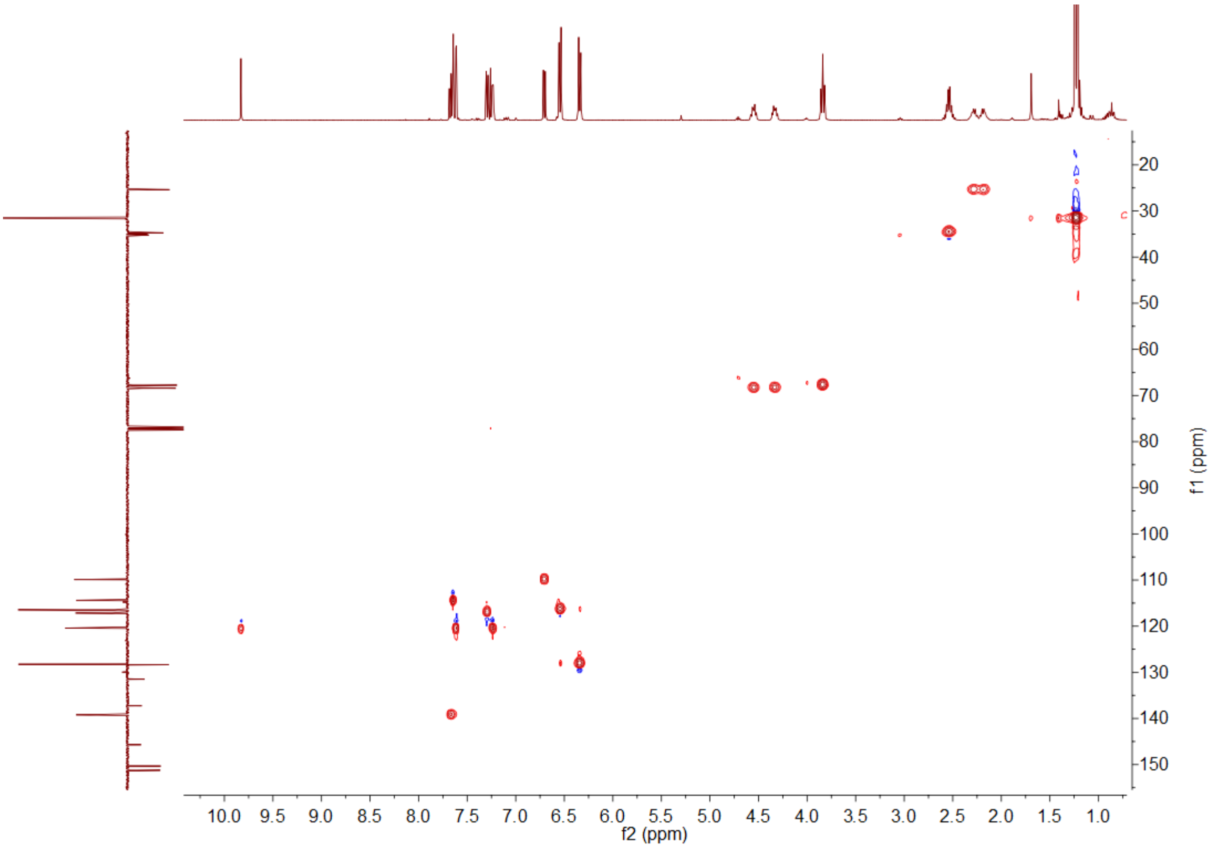

Figure S127 HSQC NMR ($CDCl_3$, 400 MHz) of S11.

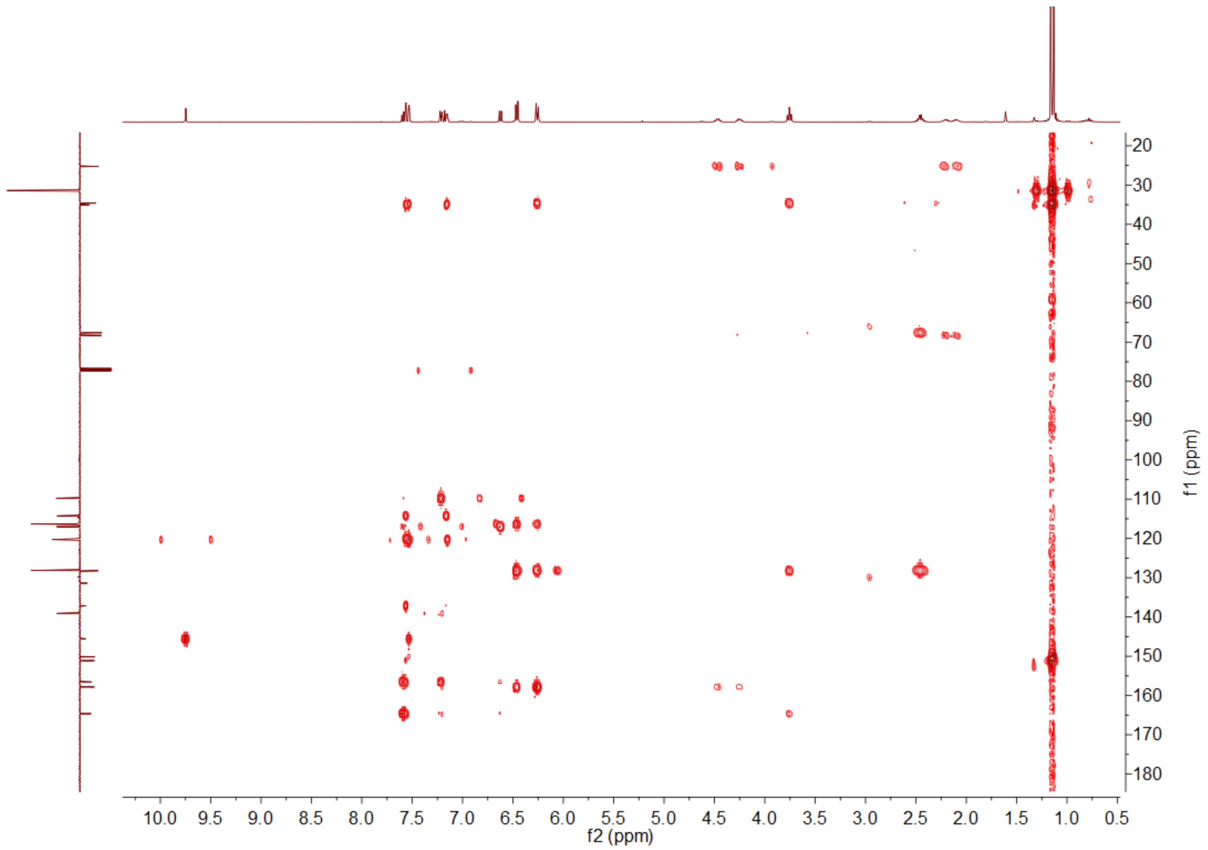

Figure S128 HMBC NMR ($CDCl_3$, 400 MHz) of S11.

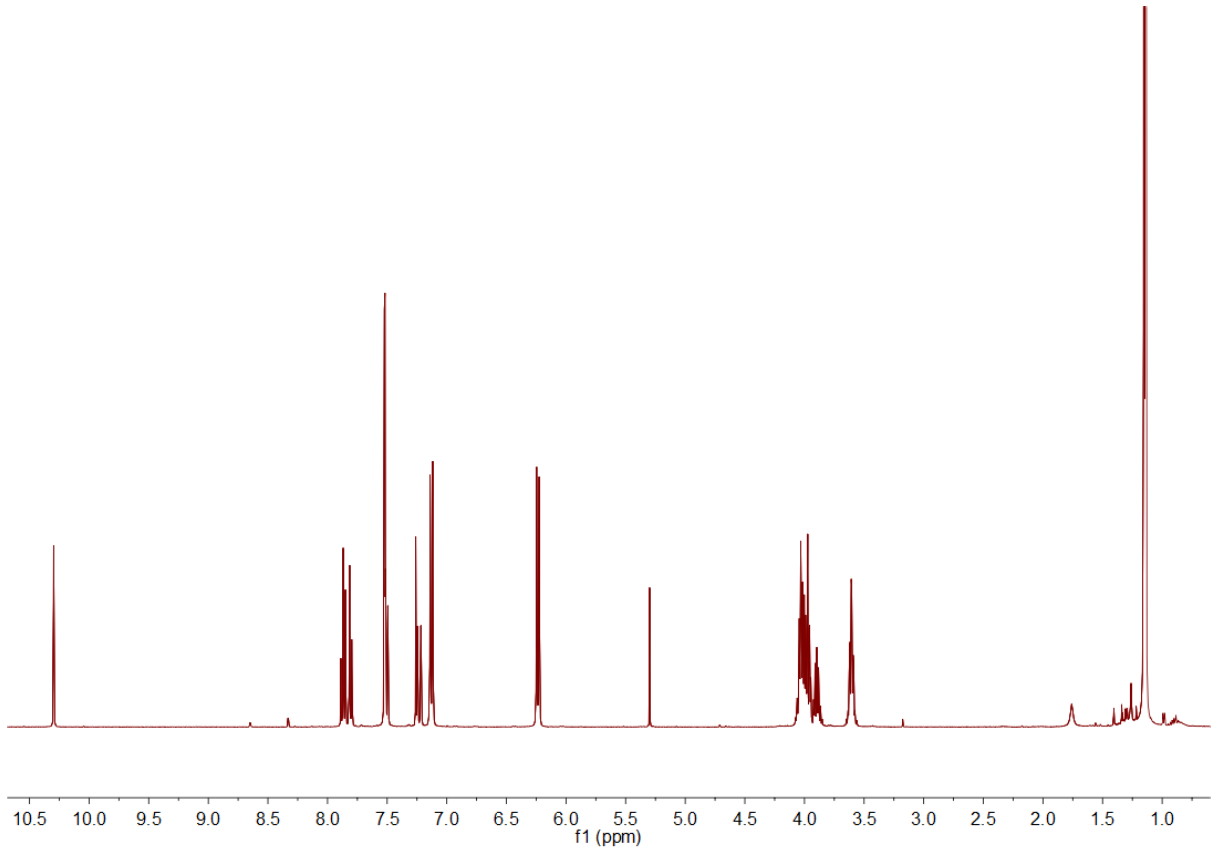

Figure S129 ^{1}H NMR ($CDCl_3$, 400 MHz) of S12.

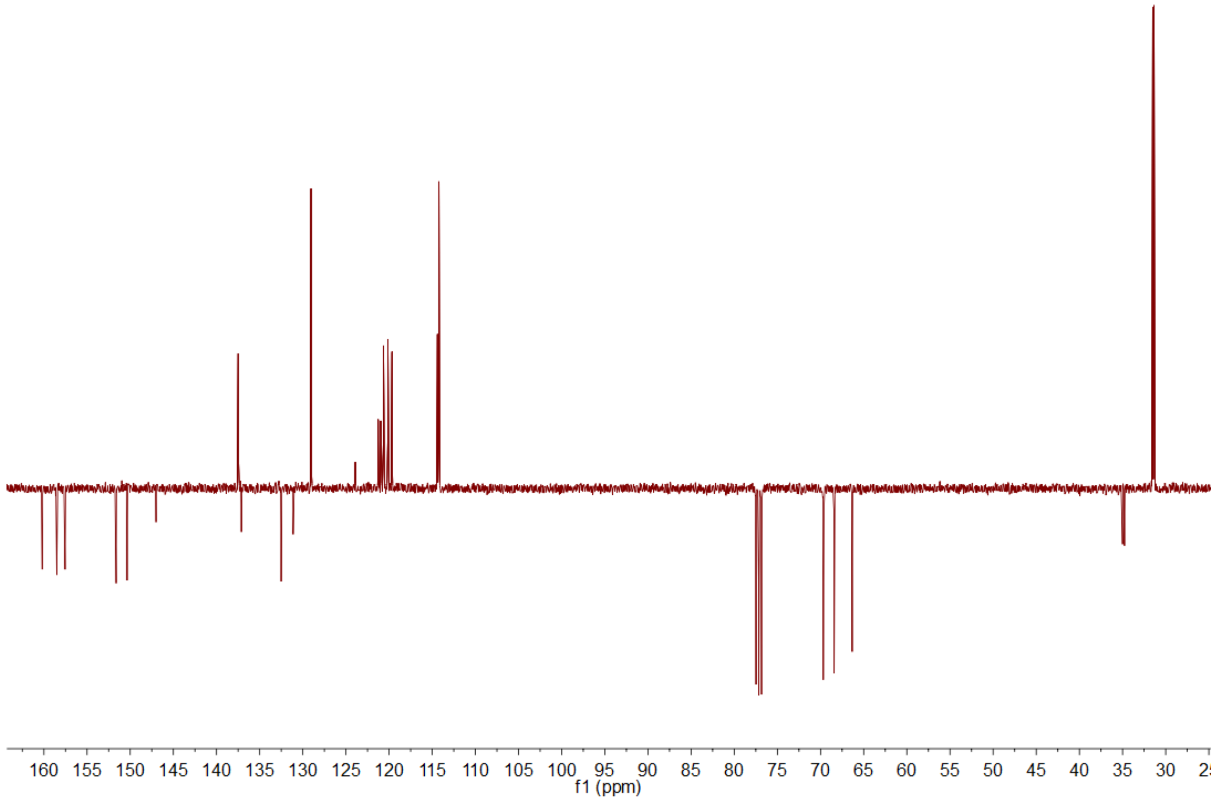

Figure S130 JMOD NMR ($CDCl_3$, 100 MHz) of S12.

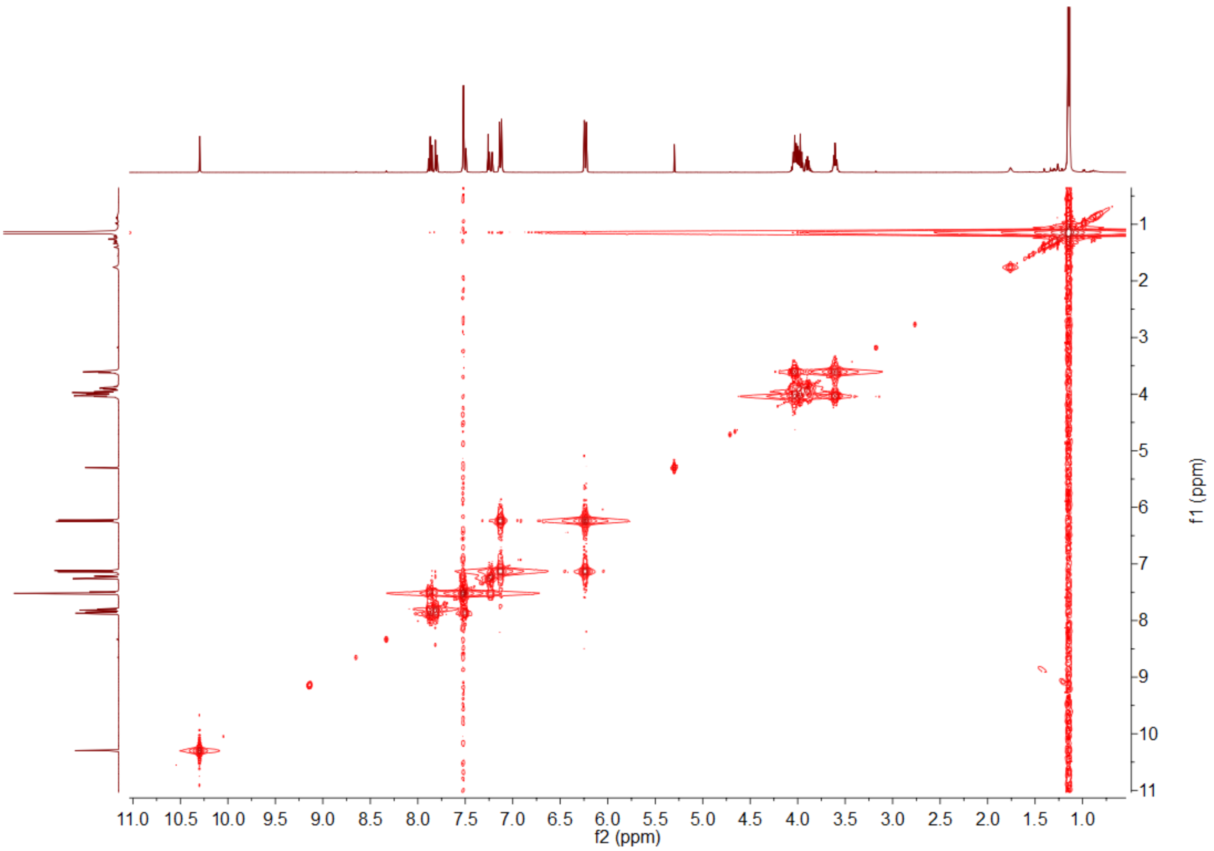

Figure S131 ^{1}H COSY NMR ($CDCl_3$, 400 MHz) of S12.

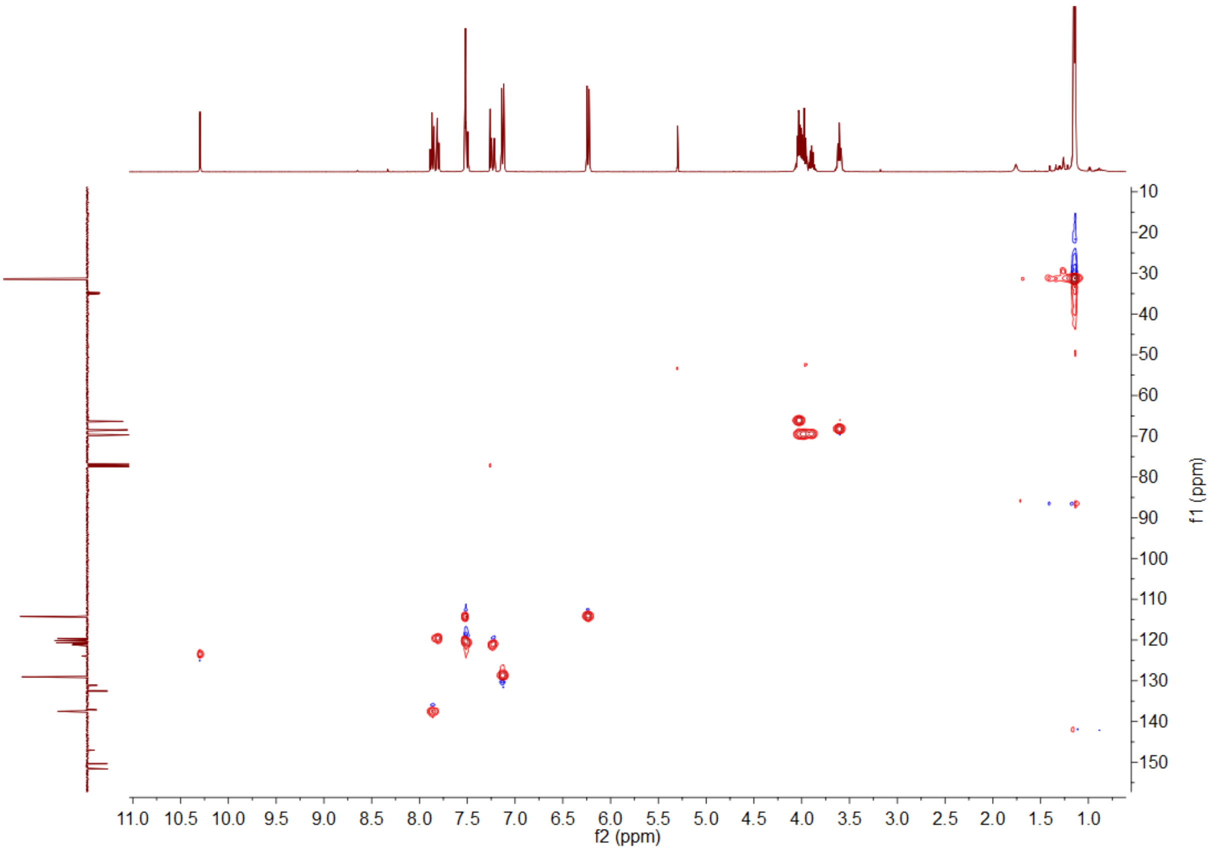

Figure S132 HSQC NMR ($CDCl_3$, 400 MHz) of S12.

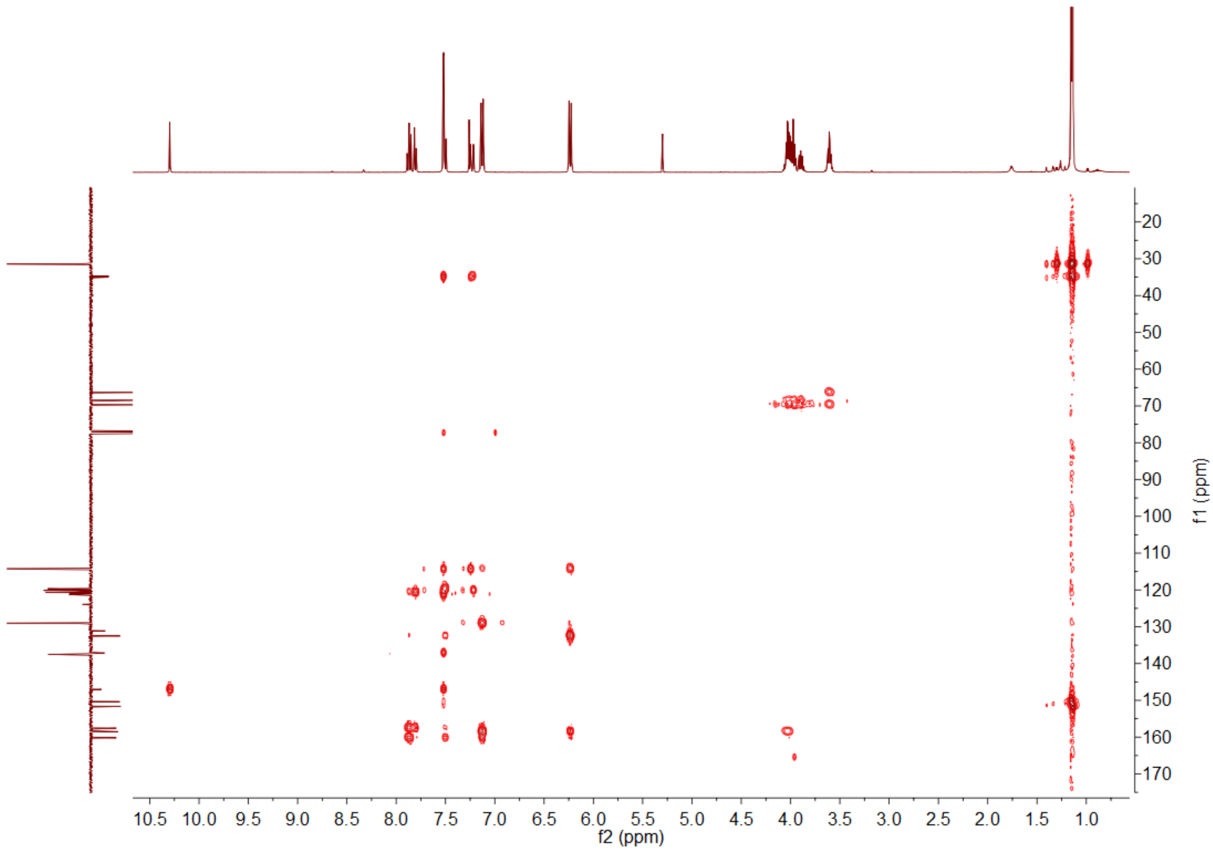

Figure S133 HMBC NMR ($CDCl_3$, 400 MHz) of S12.

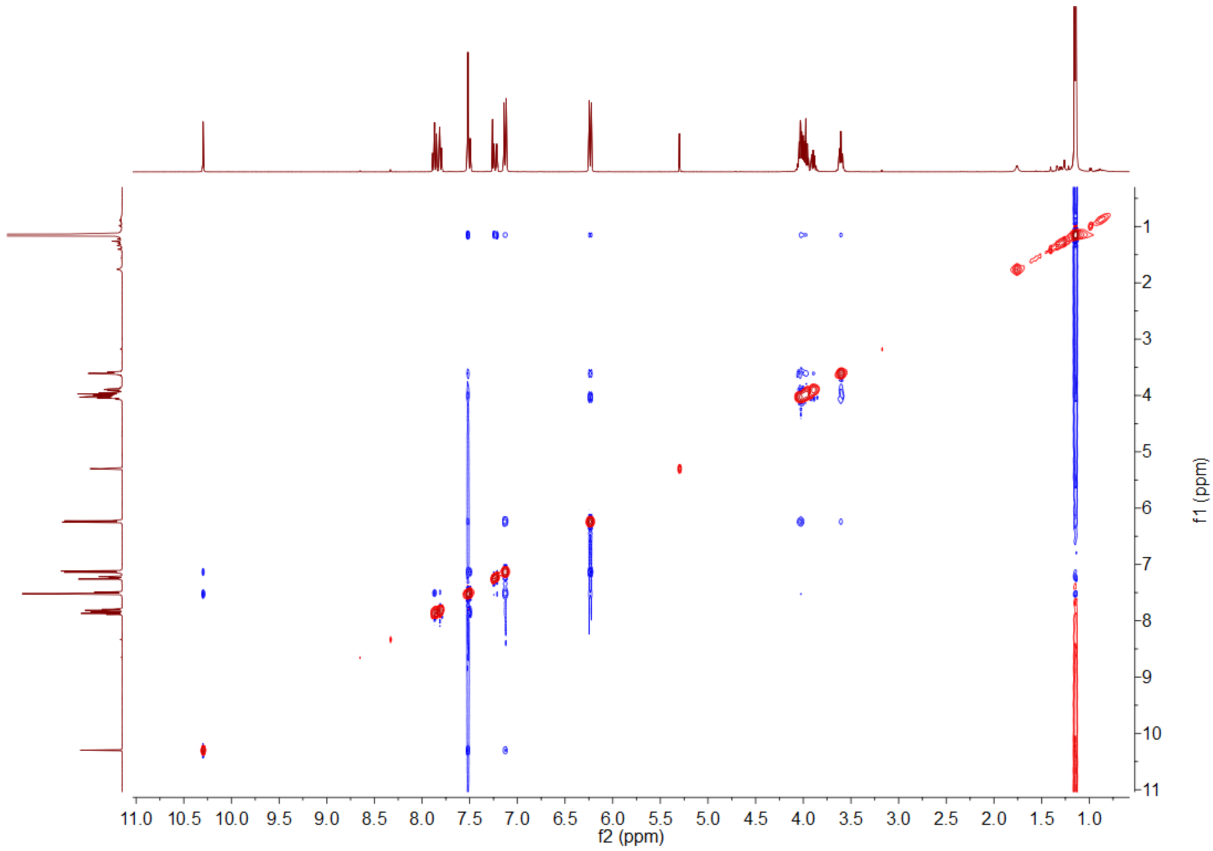

Figure S134 ^{1}H ROESY NMR ($CDCl_3$, 400 MHz) of S12.

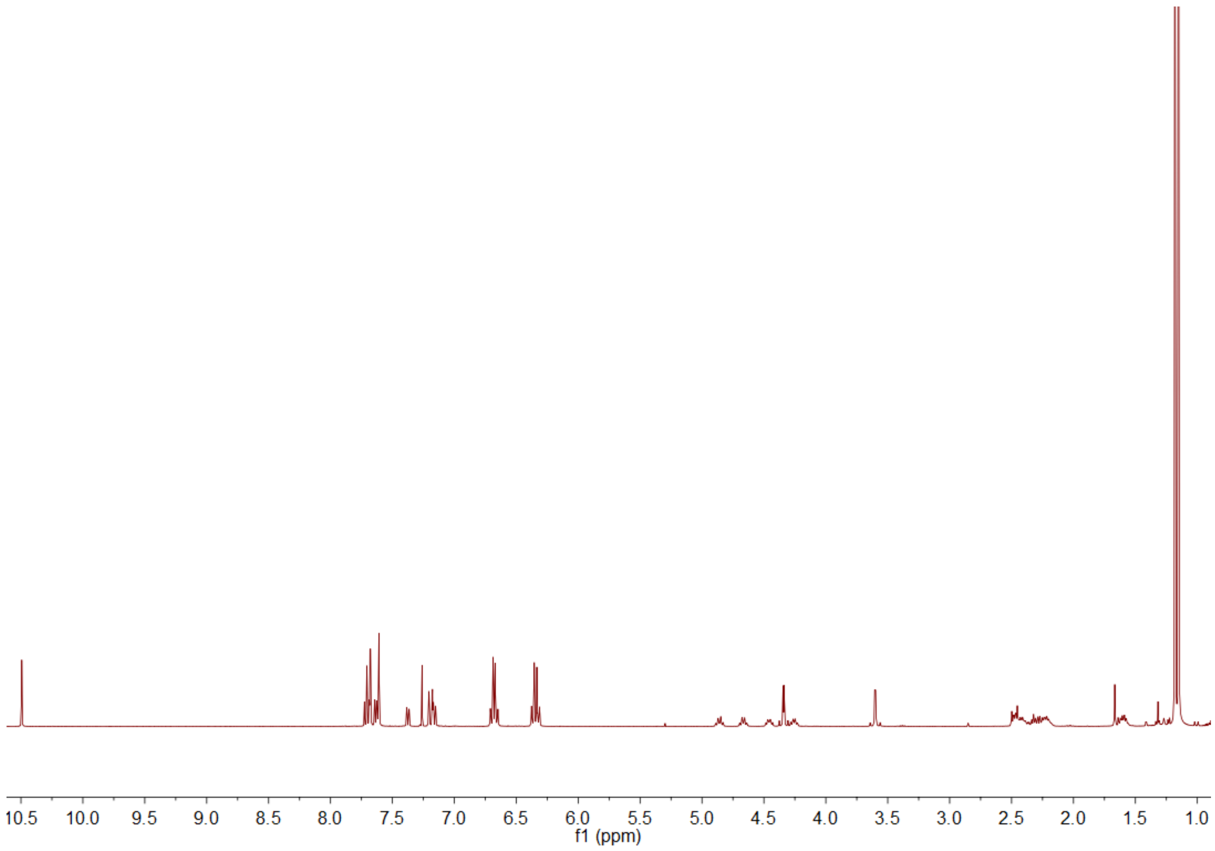

Figure S135 ^{1}H NMR ($CDCl_3$, 400 MHz) of S13.

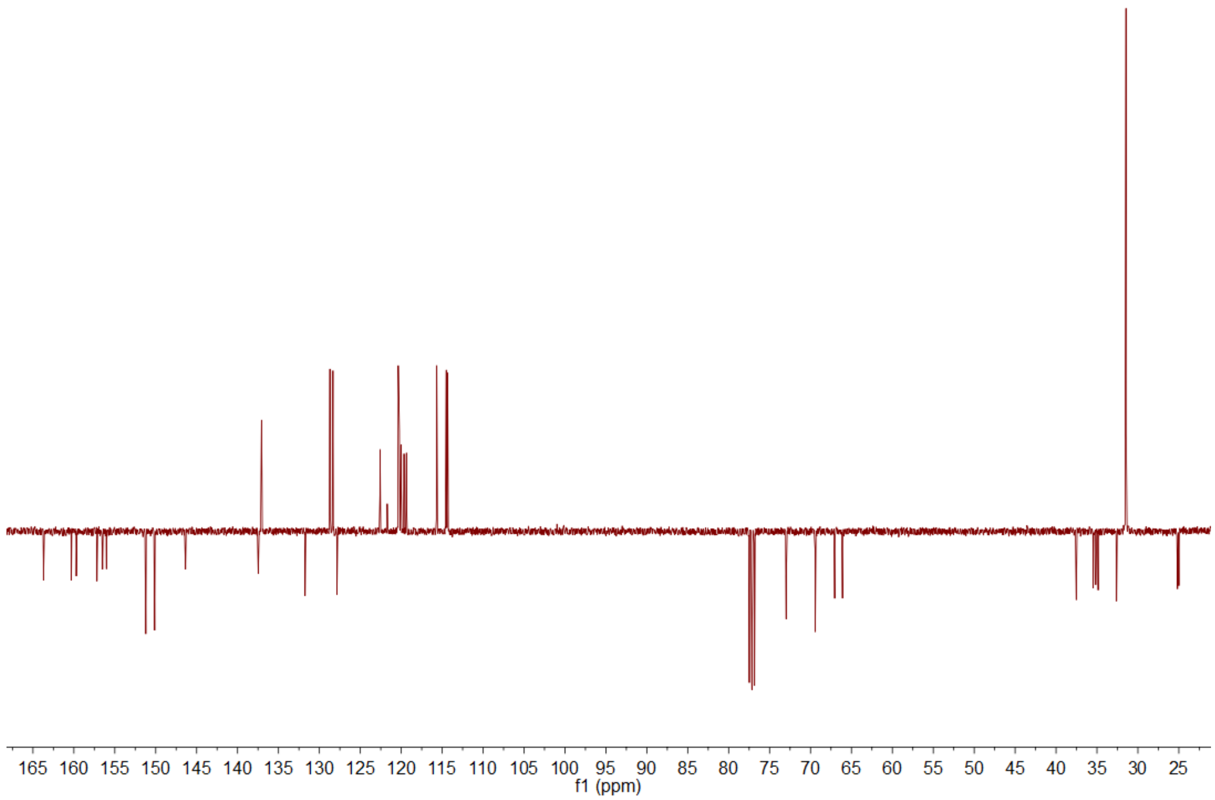

Figure S136 JMOD NMR ($CDCl_3$, 100 MHz) of S13.

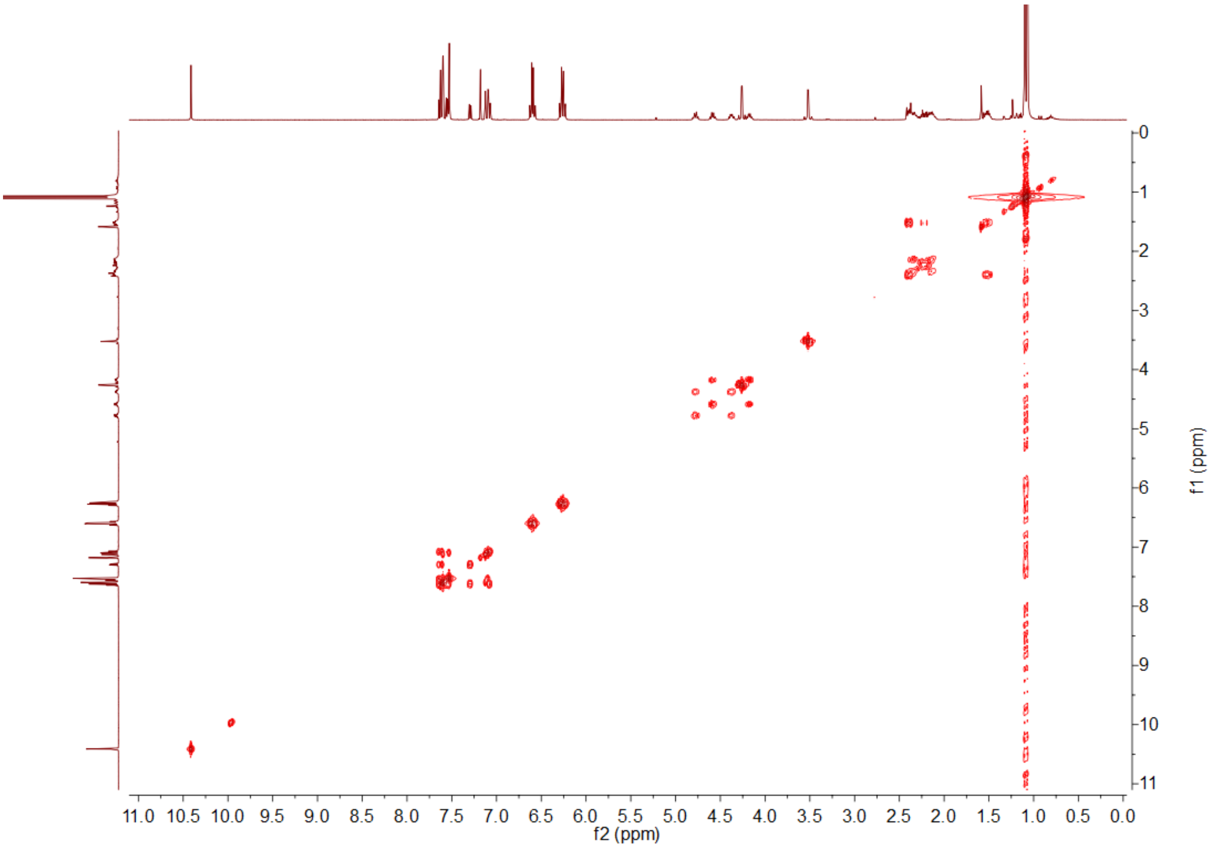

Figure S137 ^{1}H COSY NMR ($CDCl_3$, 400 MHz) of S13.

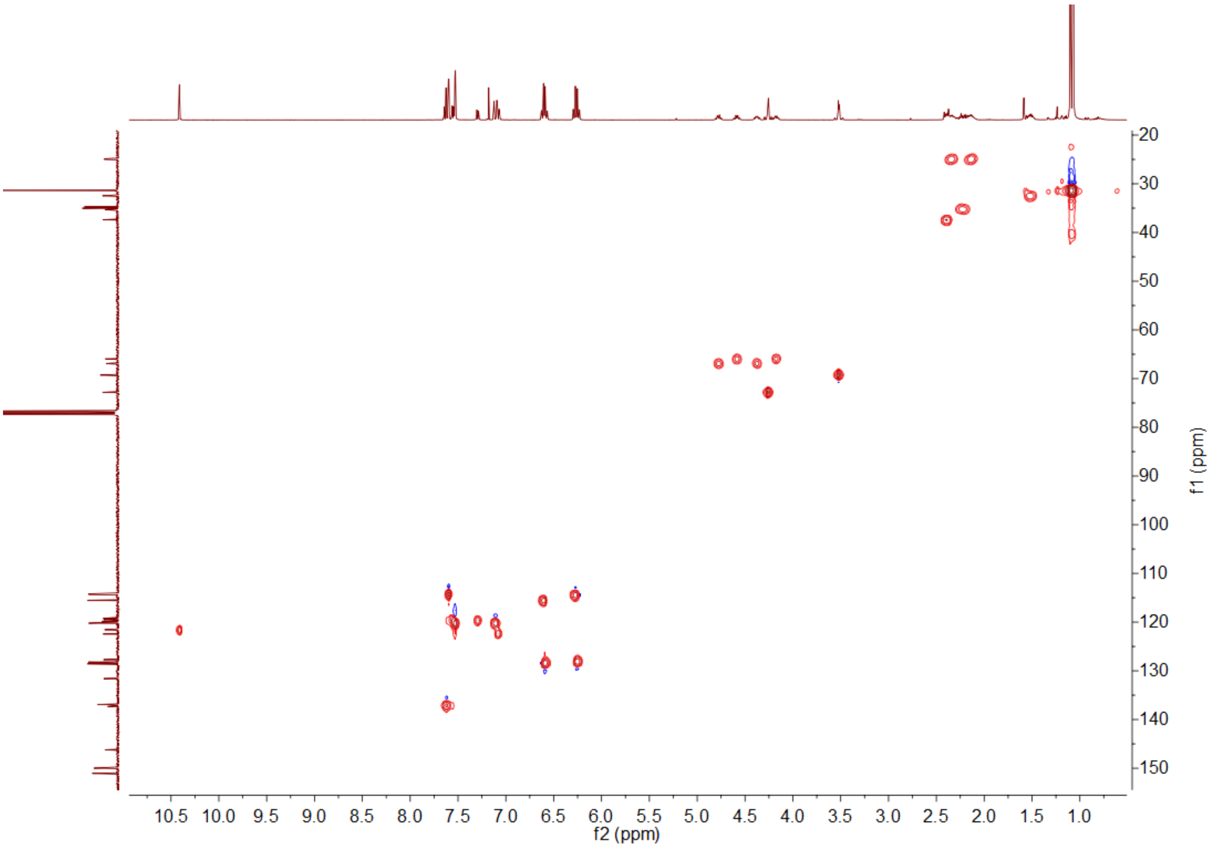

Figure S138 HSQC NMR ($CDCl_3$, 400 MHz) of S13.

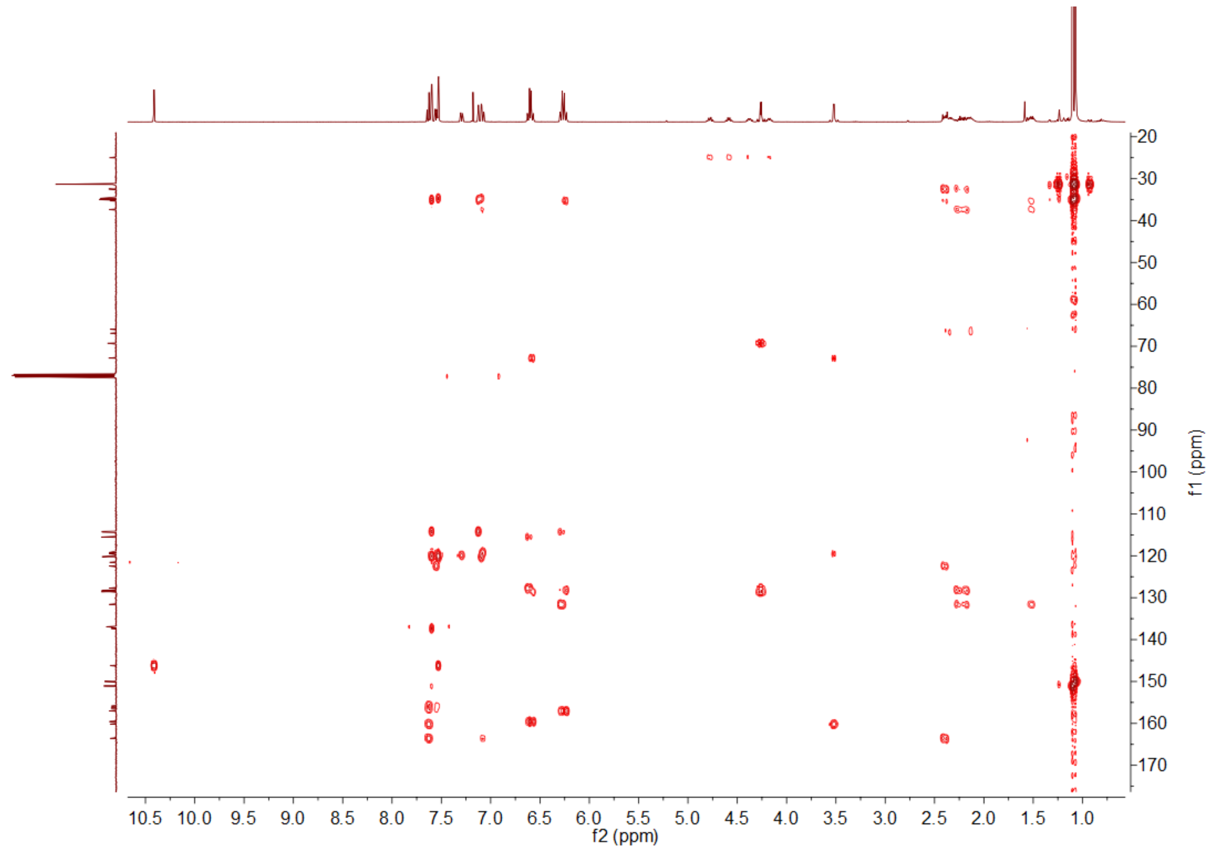

Figure S139 HMBC NMR ($CDCl_3$, 400 MHz) of S13.

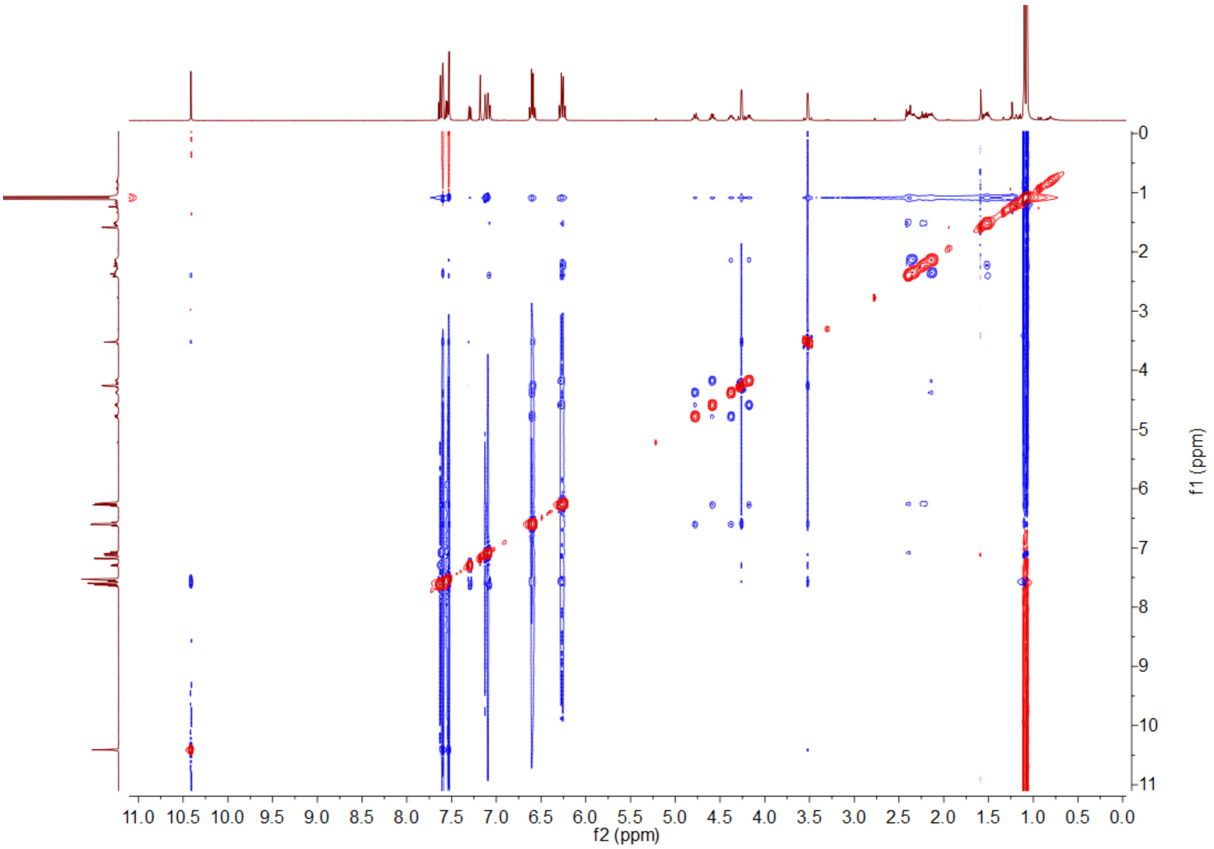

Figure S140 ^{1}H ROESY NMR ($CDCl_3$, 400 MHz) of S13.

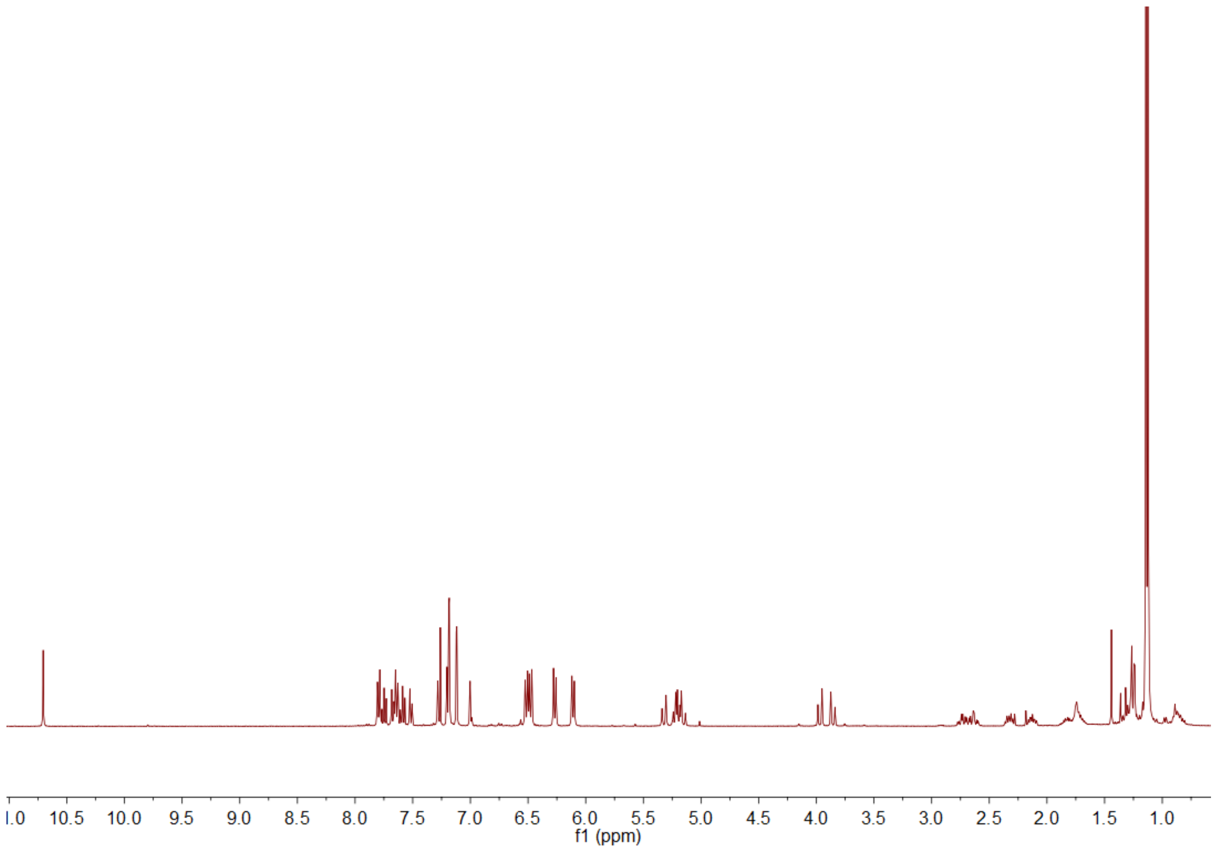

Figure S141 ^{1}H NMR ($CDCl_3$, 400 MHz) of S14.

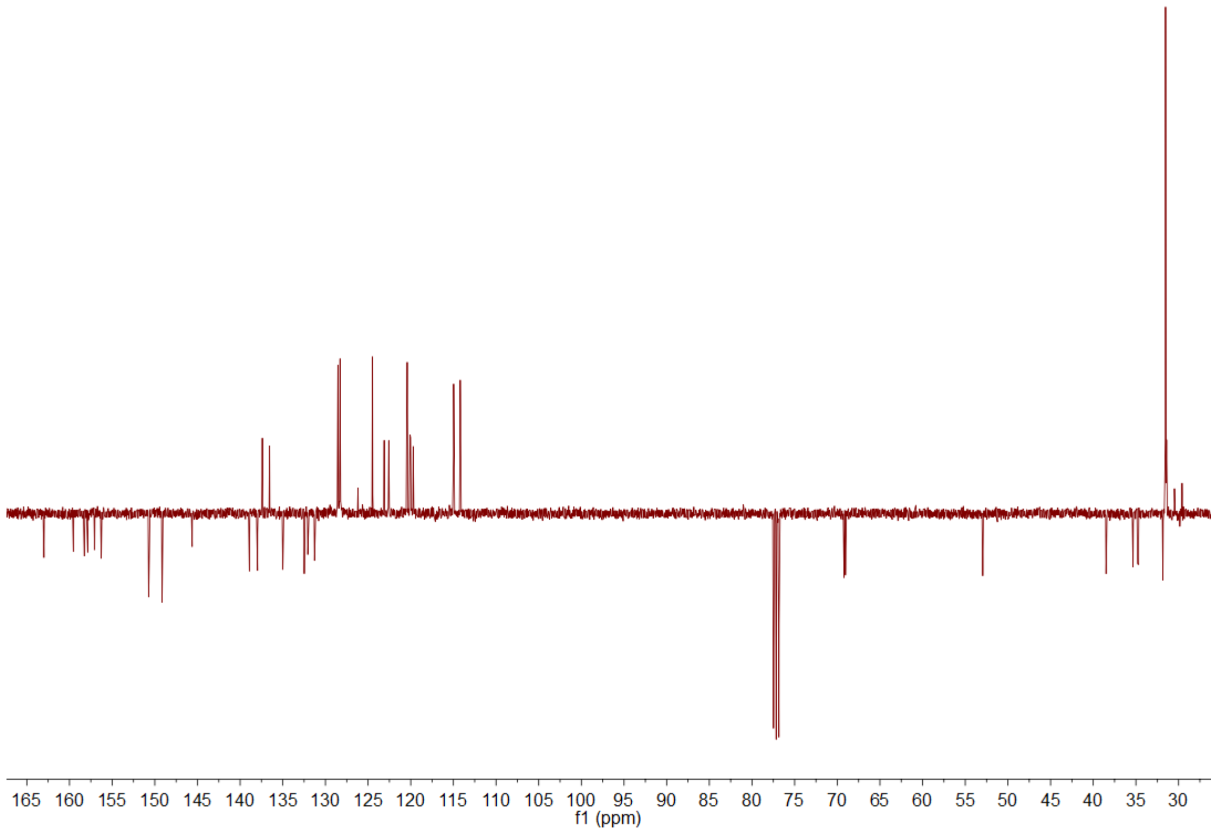

Figure S142 JMOD NMR ($CDCl_3$, 100 MHz) of S14.

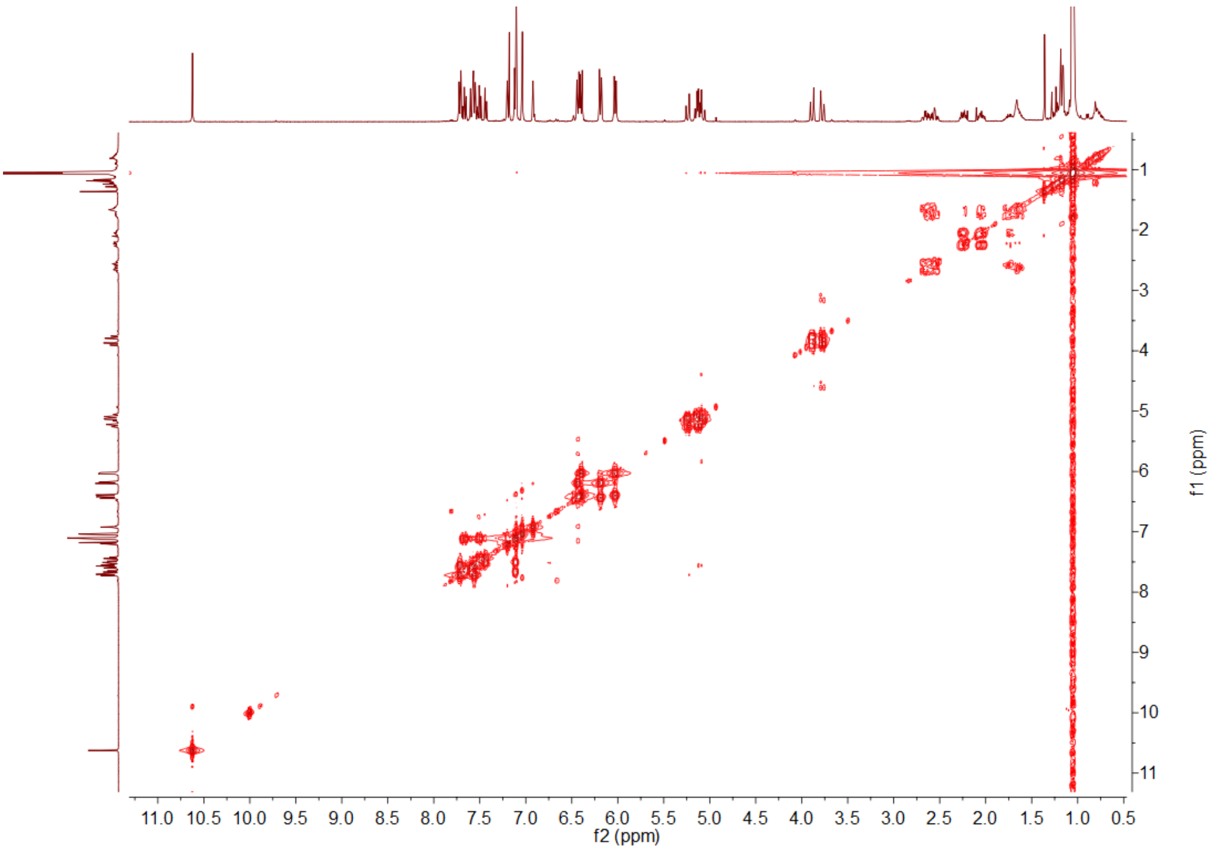

Figure S143 ^{1}H COSY NMR ($CDCl_3$, 400 MHz) of S14.

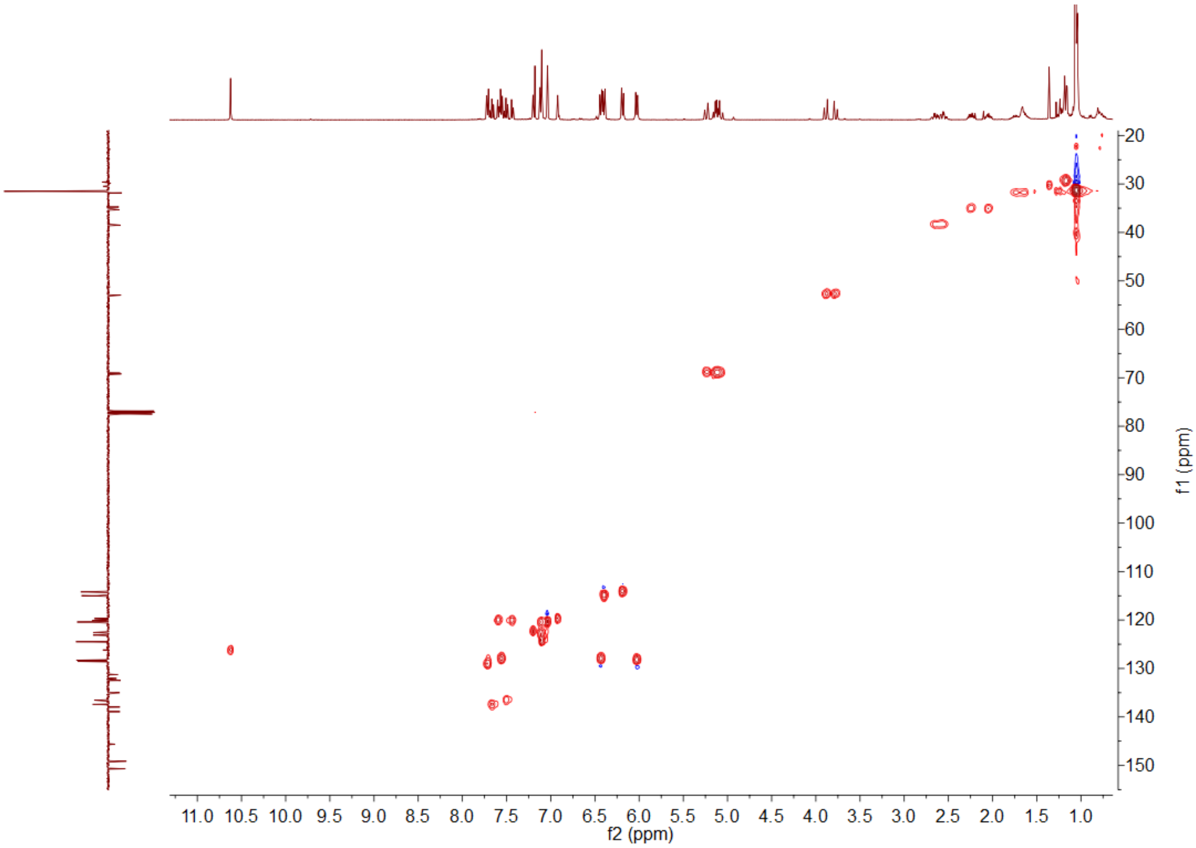

Figure S144 HSQC NMR ($CDCl_3$, 400 MHz) of S14.

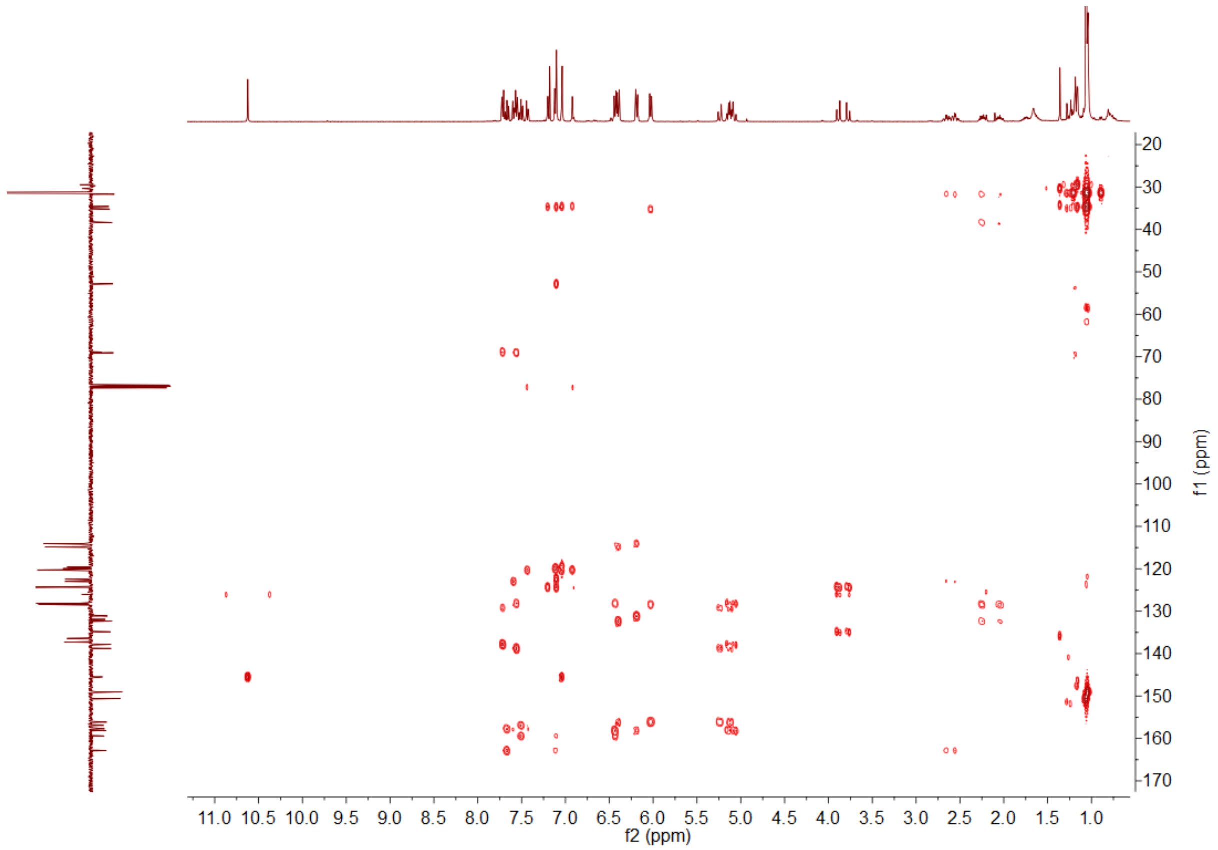

Figure S145 HMBC NMR ($CDCl_3$, 400 MHz) of S14.

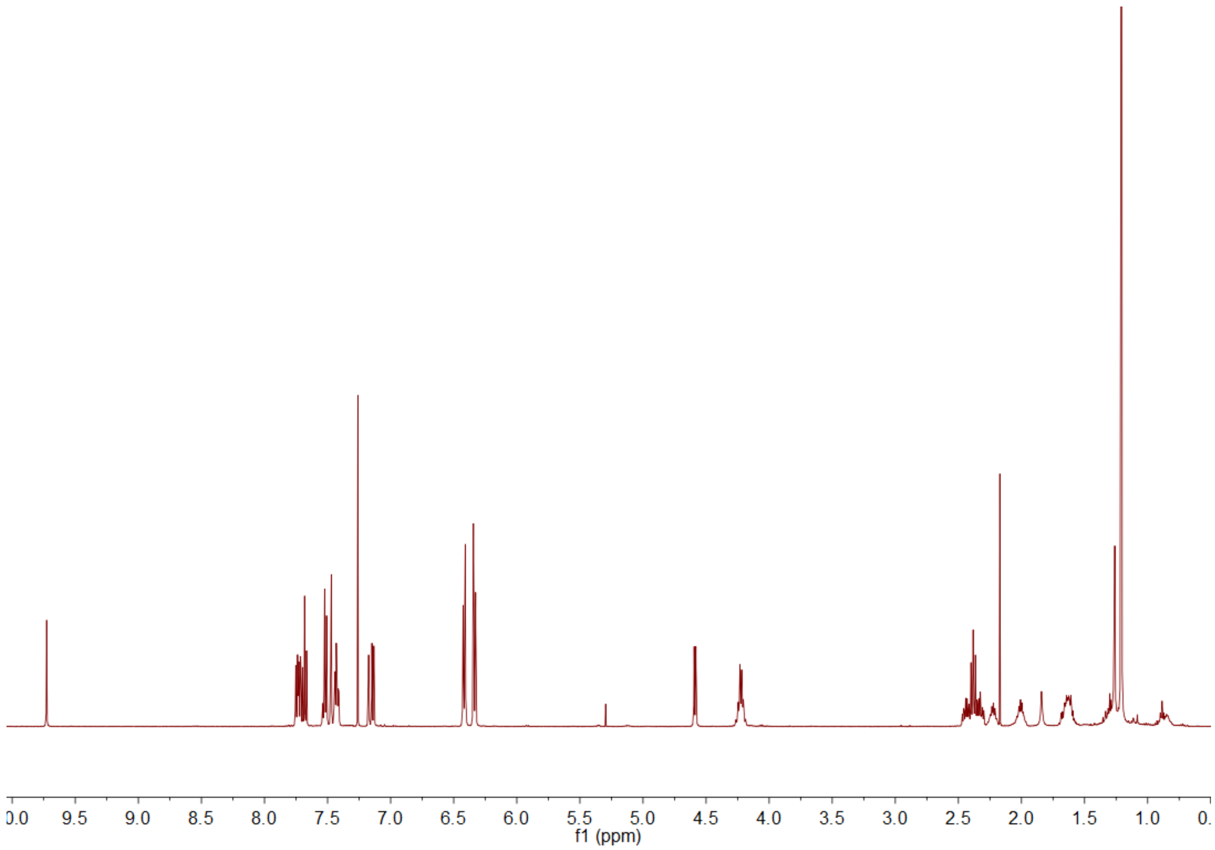

Figure S146 ^{1}H NMR ($CDCl_3$, 500 MHz) of 5.

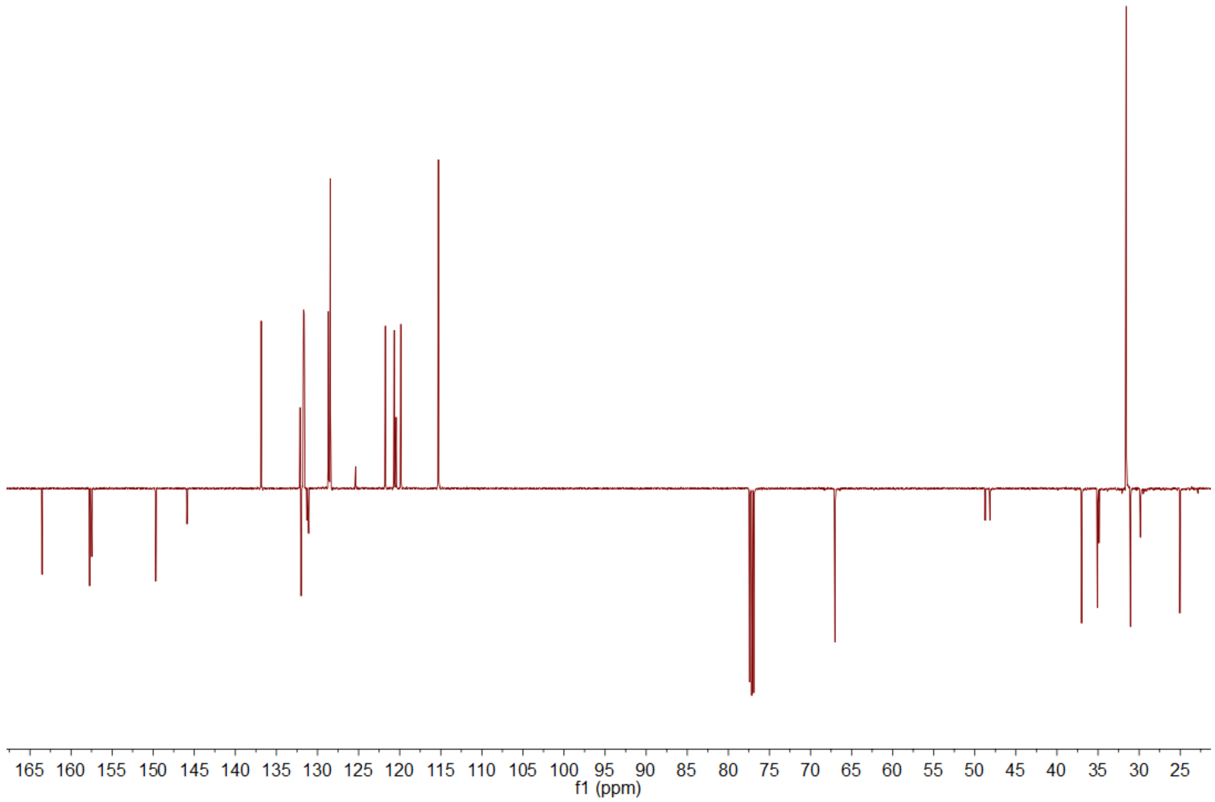

Figure S147 JMOD NMR ($CDCl_3$, 125 MHz) of 5.

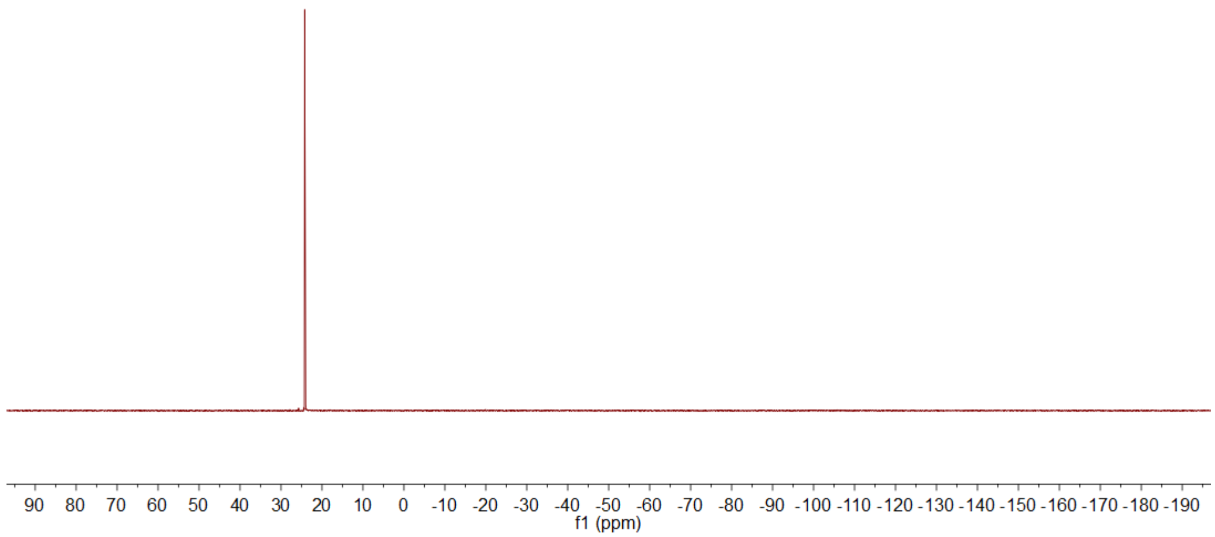

Figure S148 ^{31}P NMR ($CDCl_3$, 202 MHz) of 5.

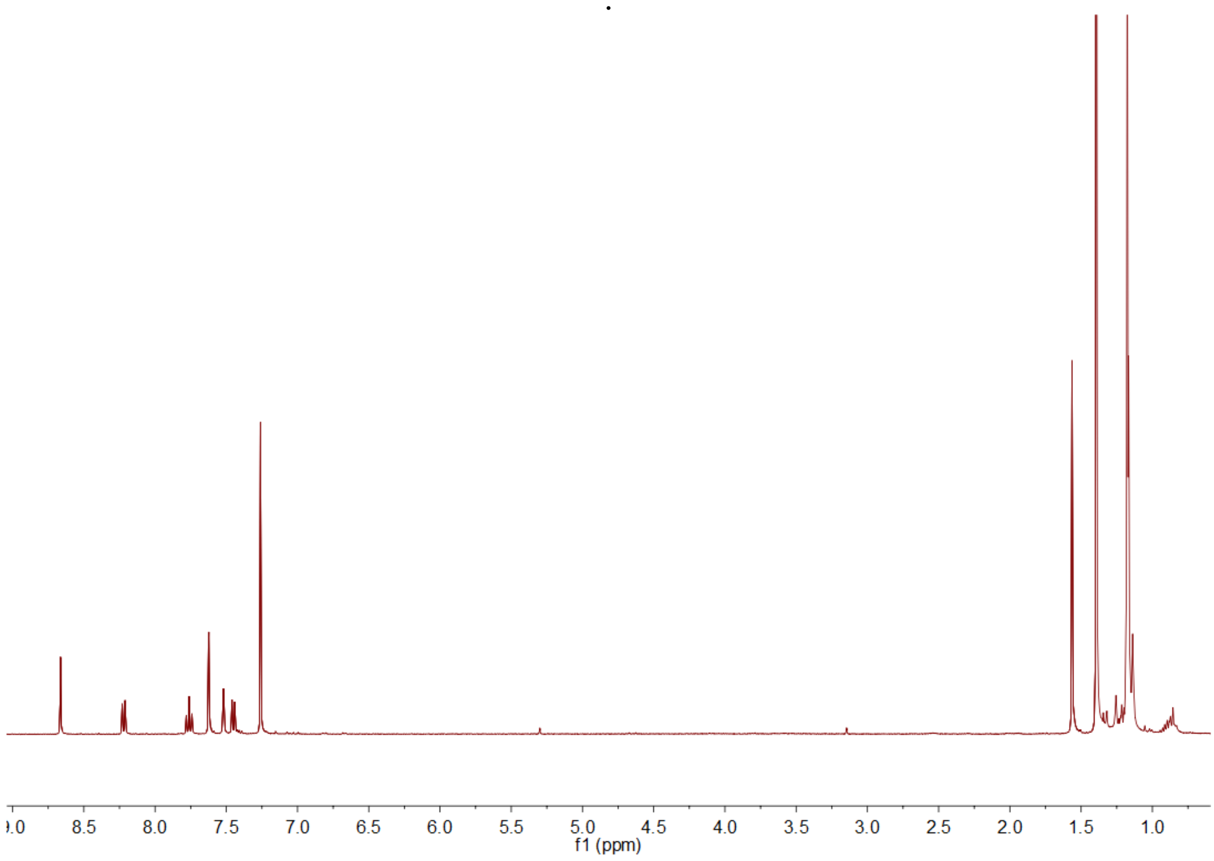

Figure S149 ^{1}H NMR ($CDCl_3$, 400 MHz) of S17.

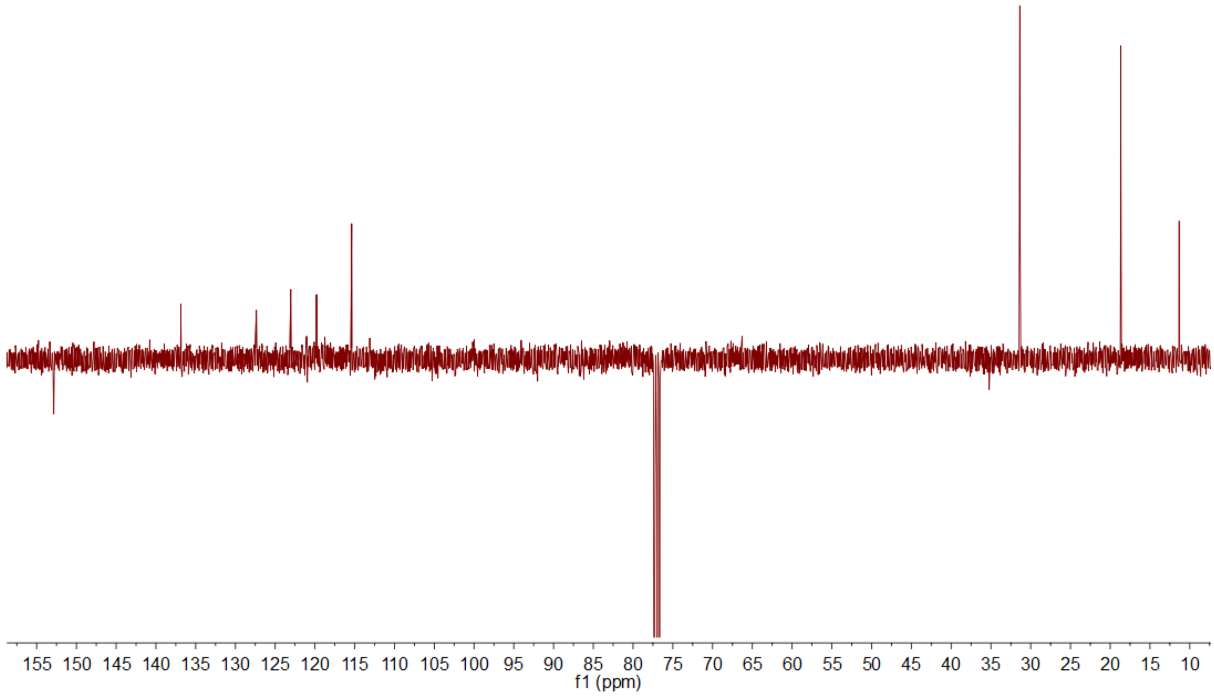

Figure S150 JMOD NMR ($CDCl_3$, 100 MHz) of S17.

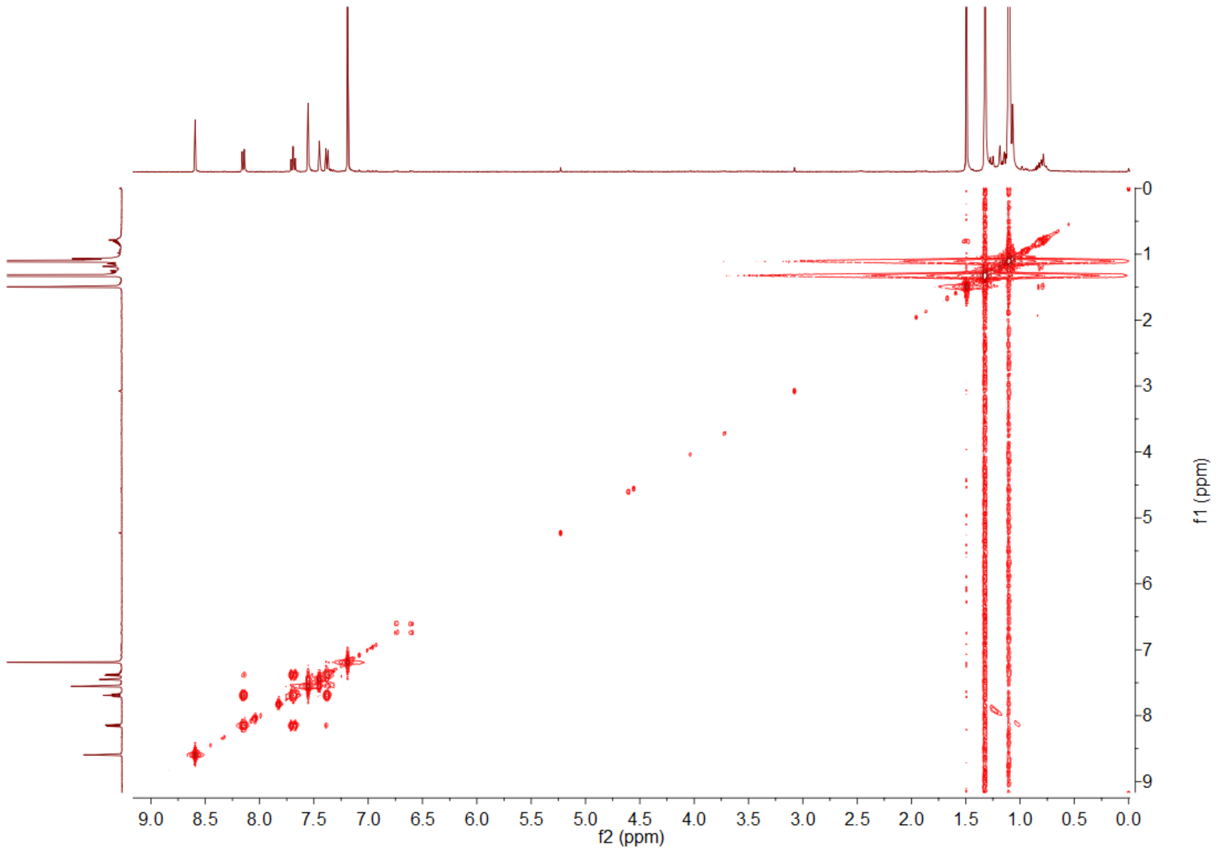

Figure S151 ¹H COSY NMR ($CDCl_3$, 400 MHz) of S17.

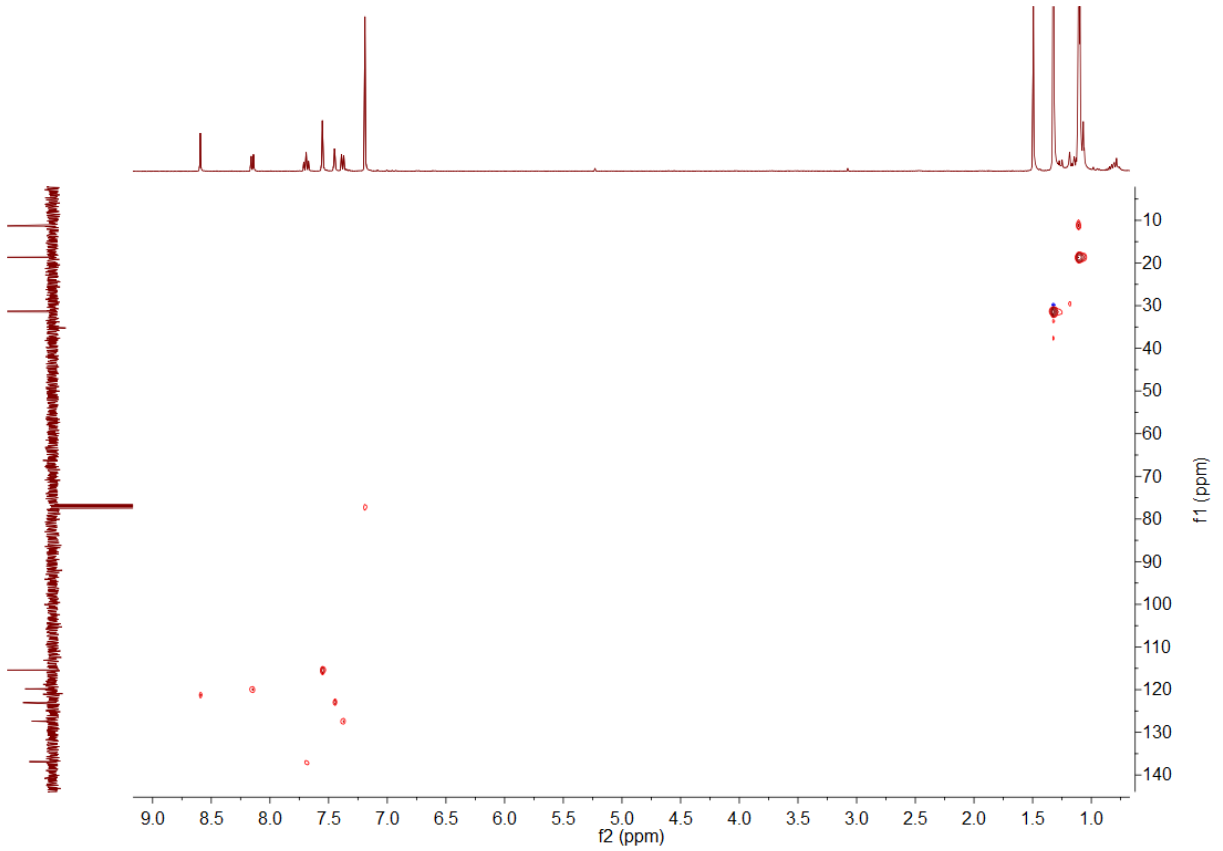

Figure S152 HSQC NMR ($CDCl_3$, 400 MHz) of S17.

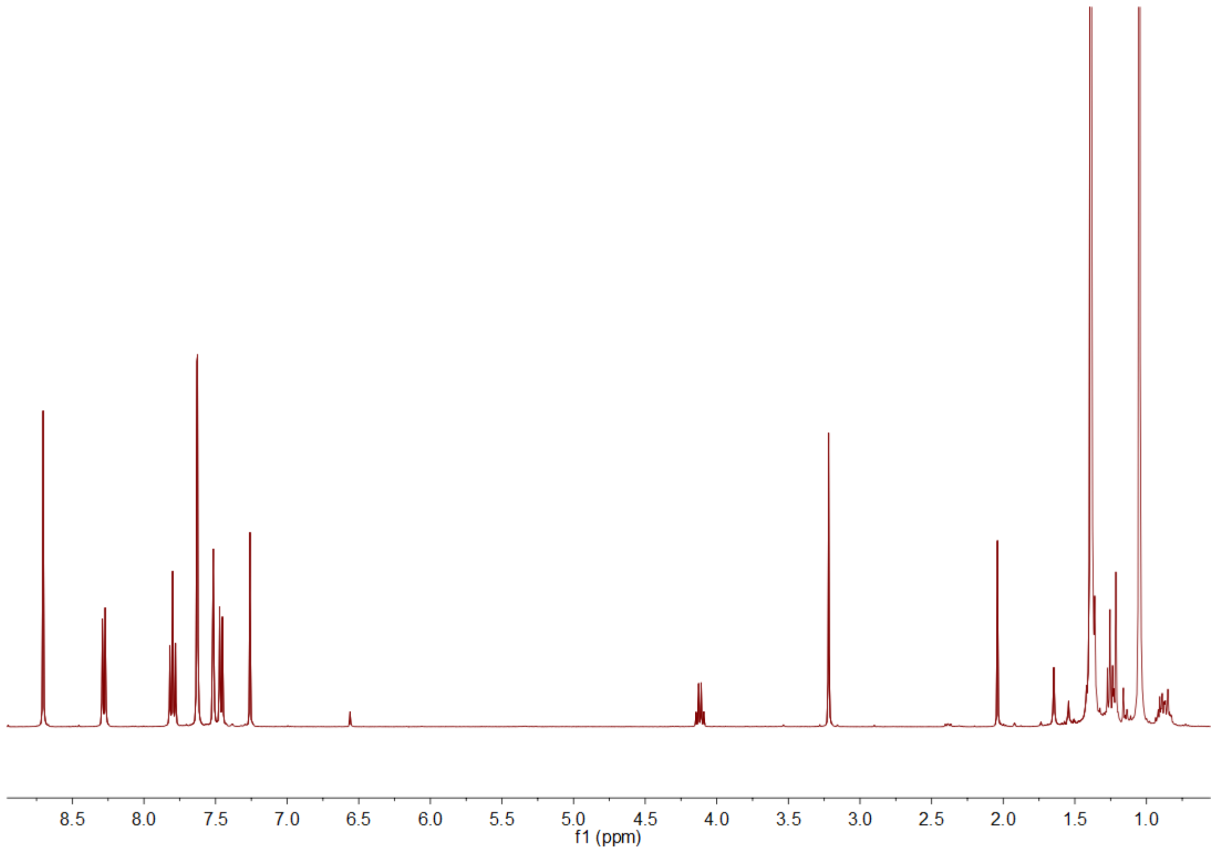

Figure S153 ^{1}H NMR ($CDCl_3$, 400 MHz) of 6.

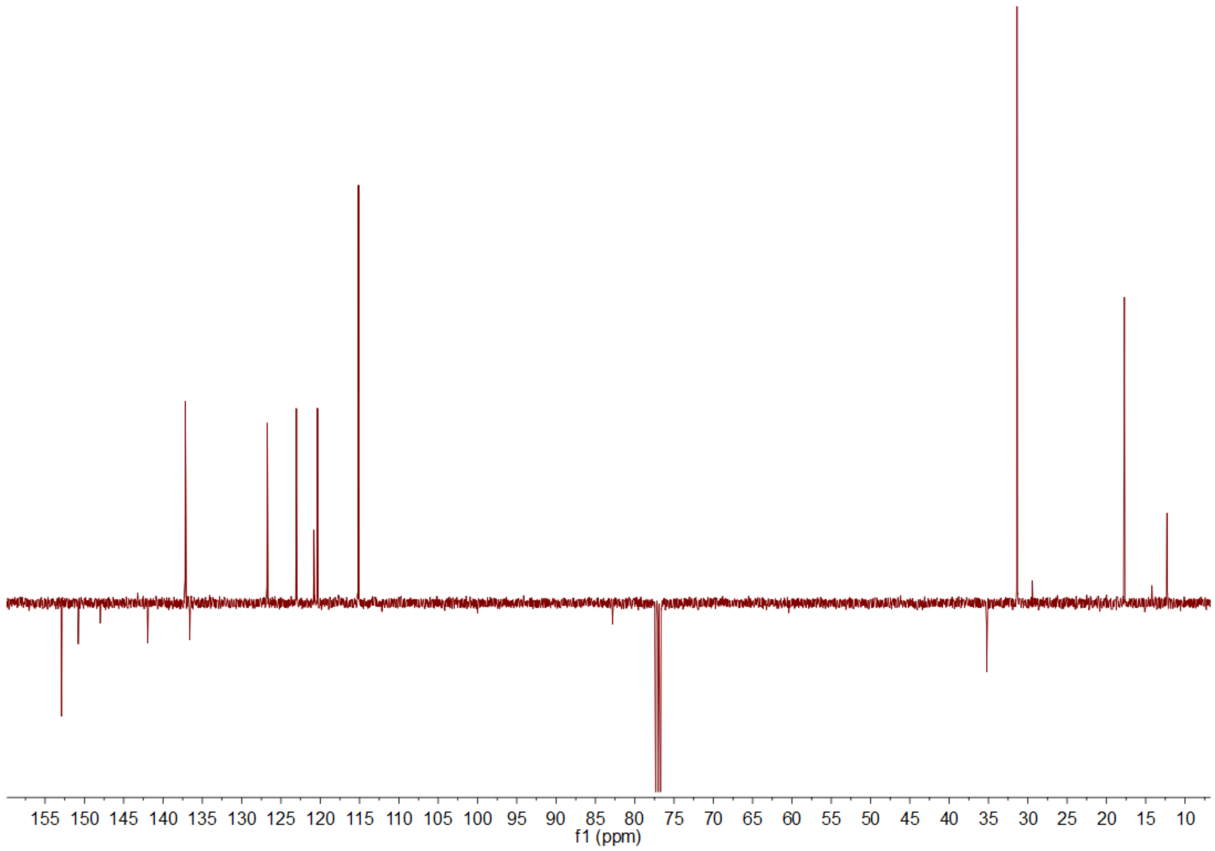

Figure S154 JMOD NMR ($CDCl_3$, 100 MHz) of 6.

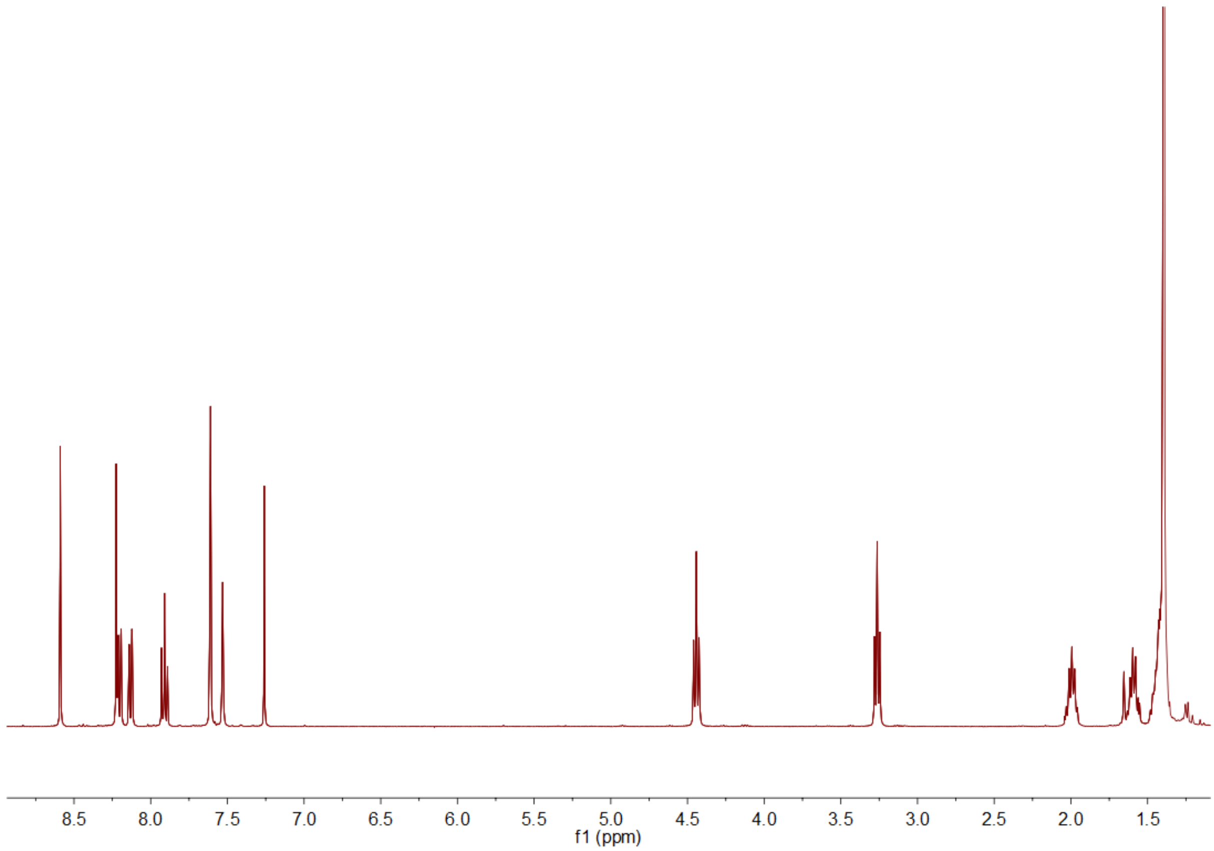

Figure S155 ^{1}H NMR ($CDCl_3$, 400 MHz) of 7.

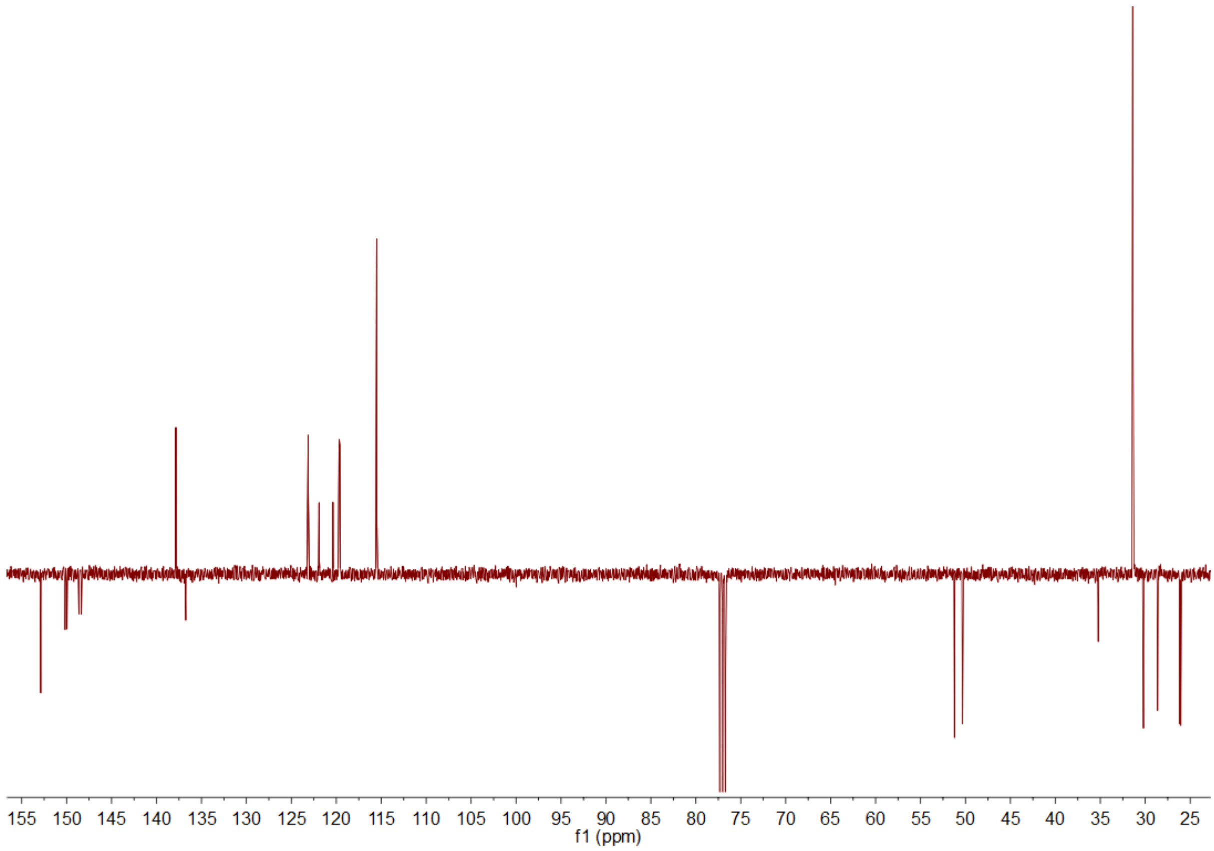

Figure S156 JMOD NMR ($CDCl_3$, 100 MHz) of 7.

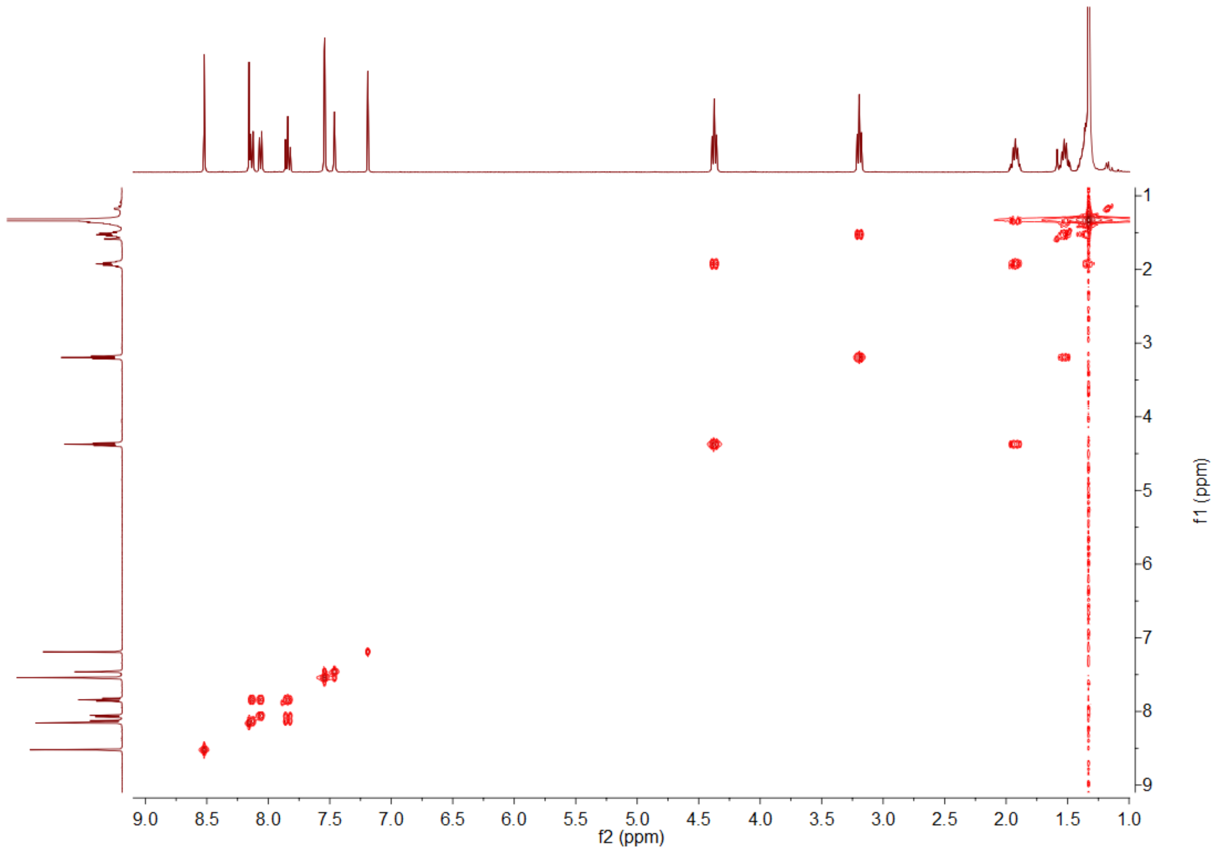

Figure S157 ^{1}H COSY NMR ($CDCl_3$, 400 MHz) of 7.

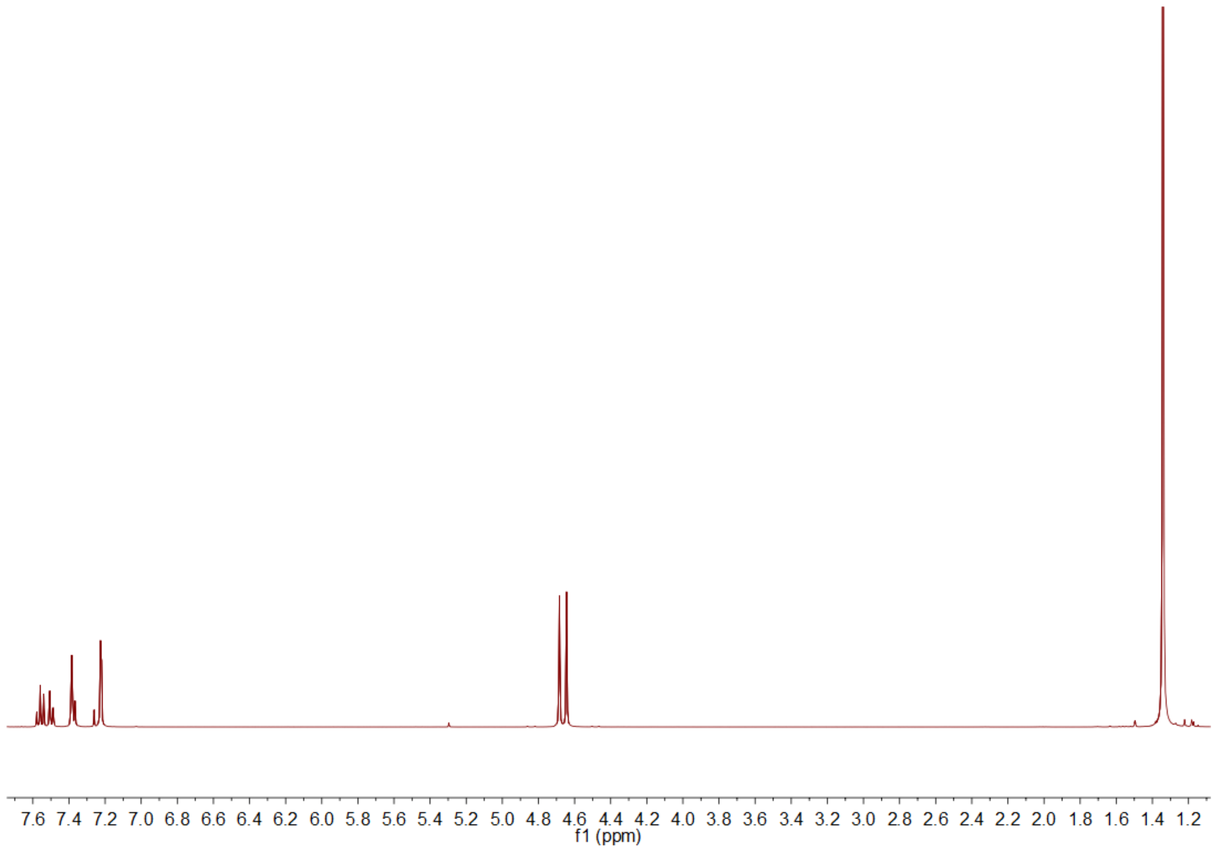

Figure S158 ^{1}H NMR ($CDCl_3$, 400 MHz) of S18.

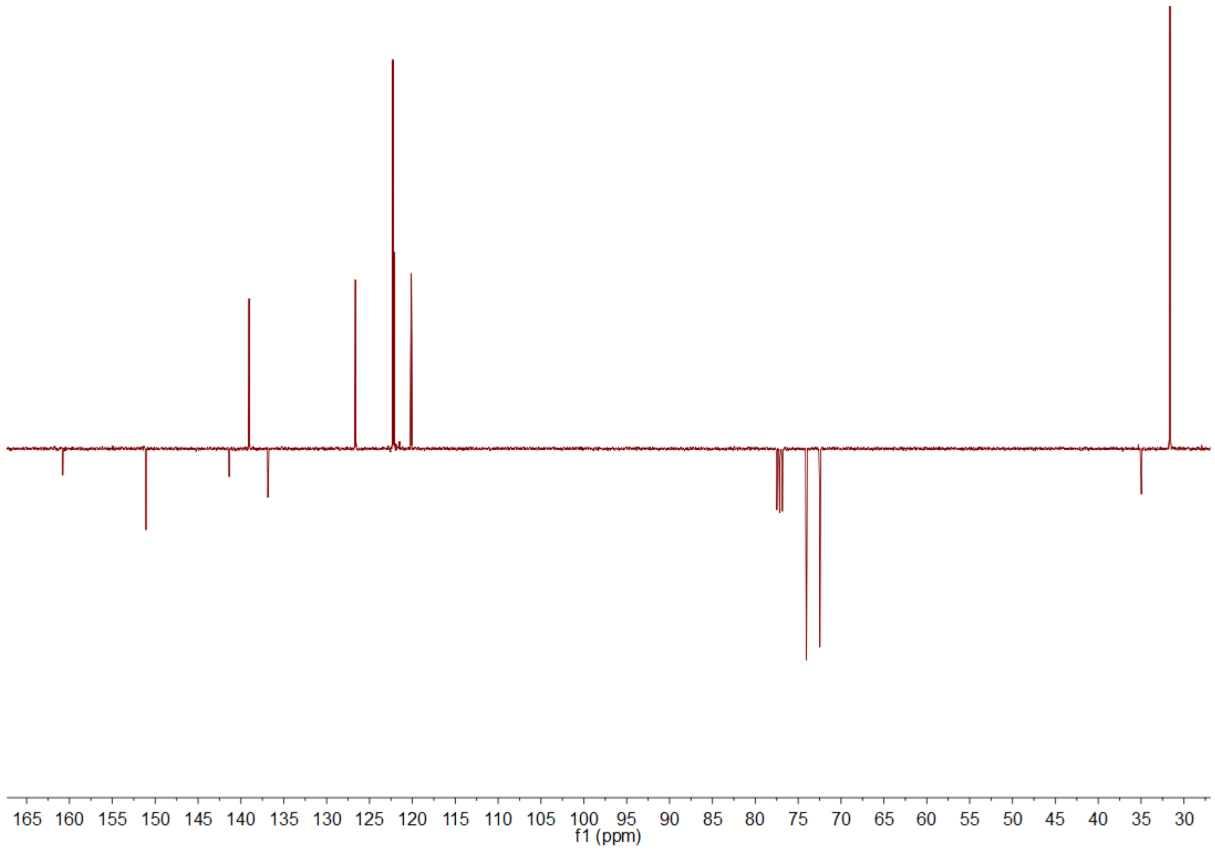

Figure S159 JMOD NMR ($CDCl_3$, 100 MHz) of S18.

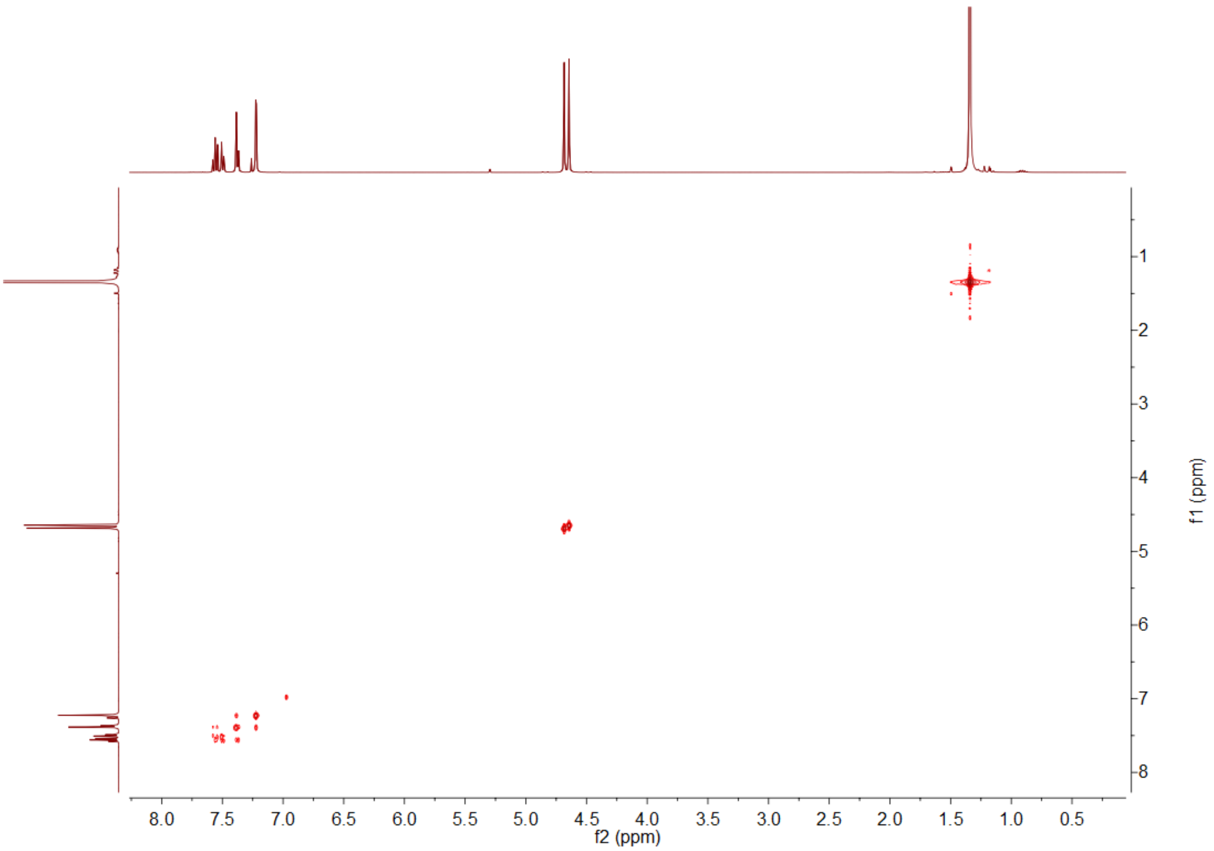

Figure S160 ¹H COSY NMR ($CDCl_3$, 400 MHz) of S18.

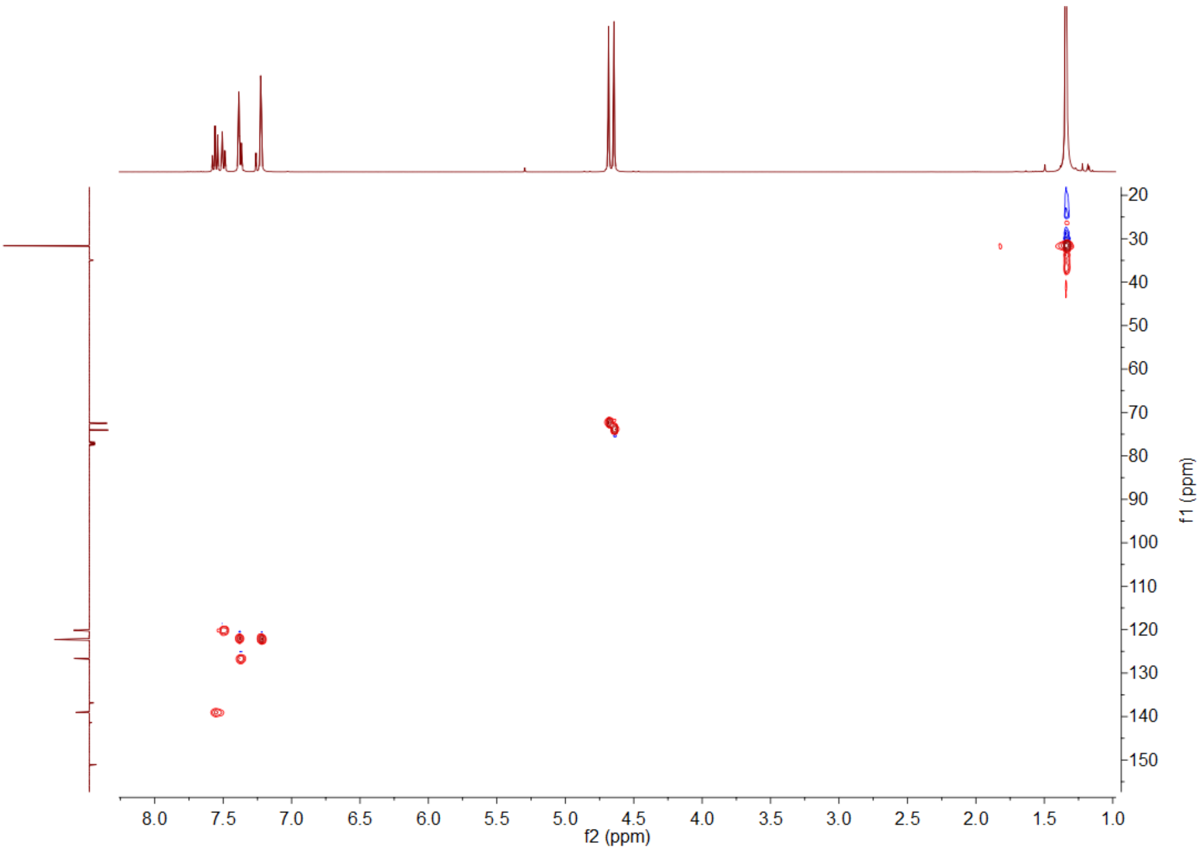

Figure S161 HSQC NMR ($CDCl_3$, 400 MHz) of S18.

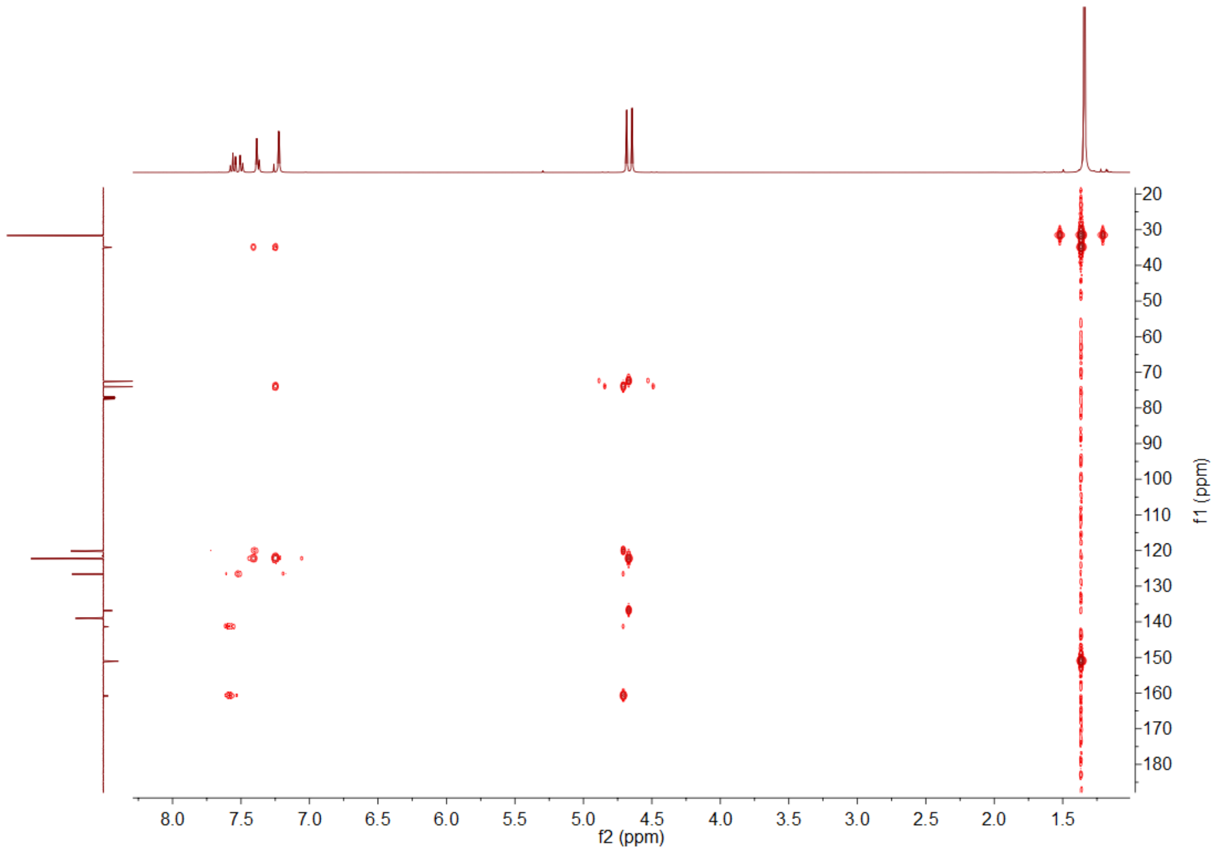

Figure S162 HMBC NMR ($CDCl_3$, 400 MHz) of S18.

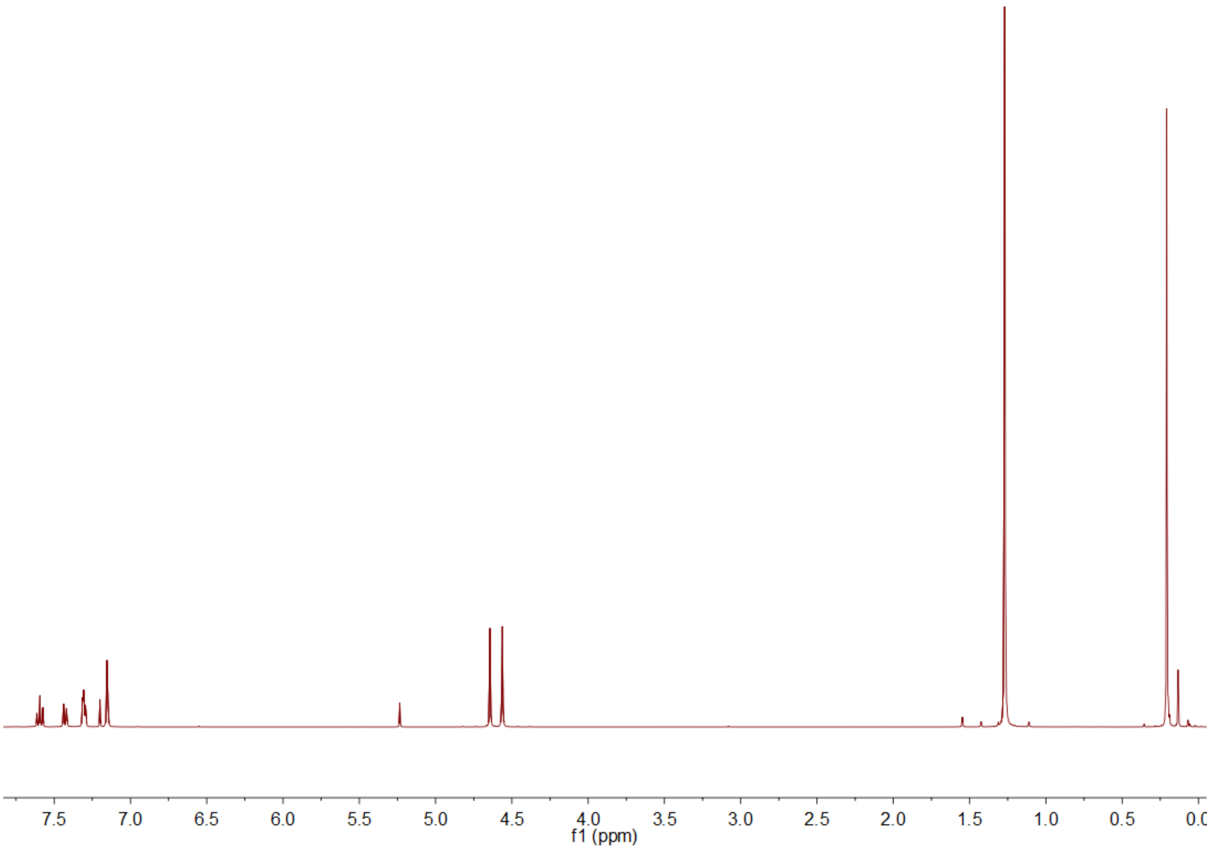

Figure S163 ^{1}H NMR ($CDCl_3$, 400 MHz) of S19.

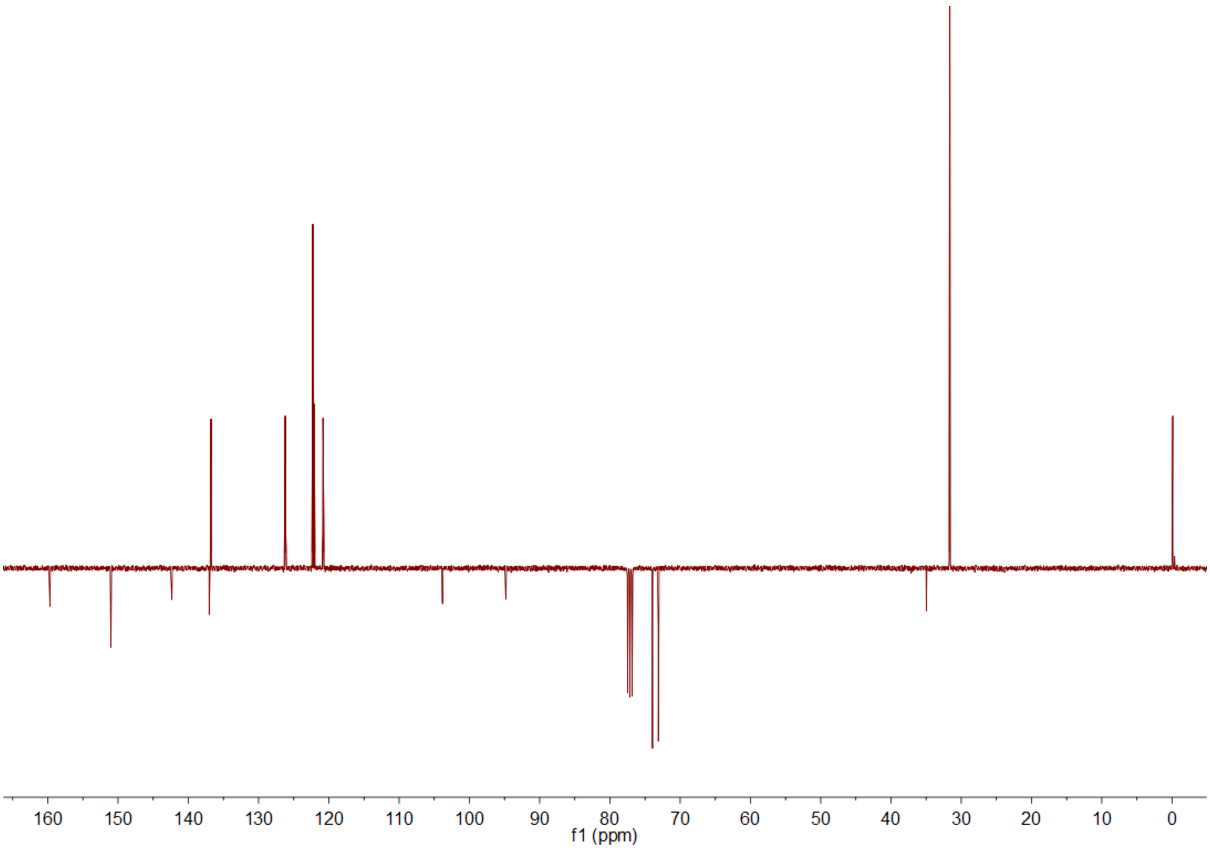

Figure S164 JMOD NMR ($CDCl_3$, 100 MHz) of S19.

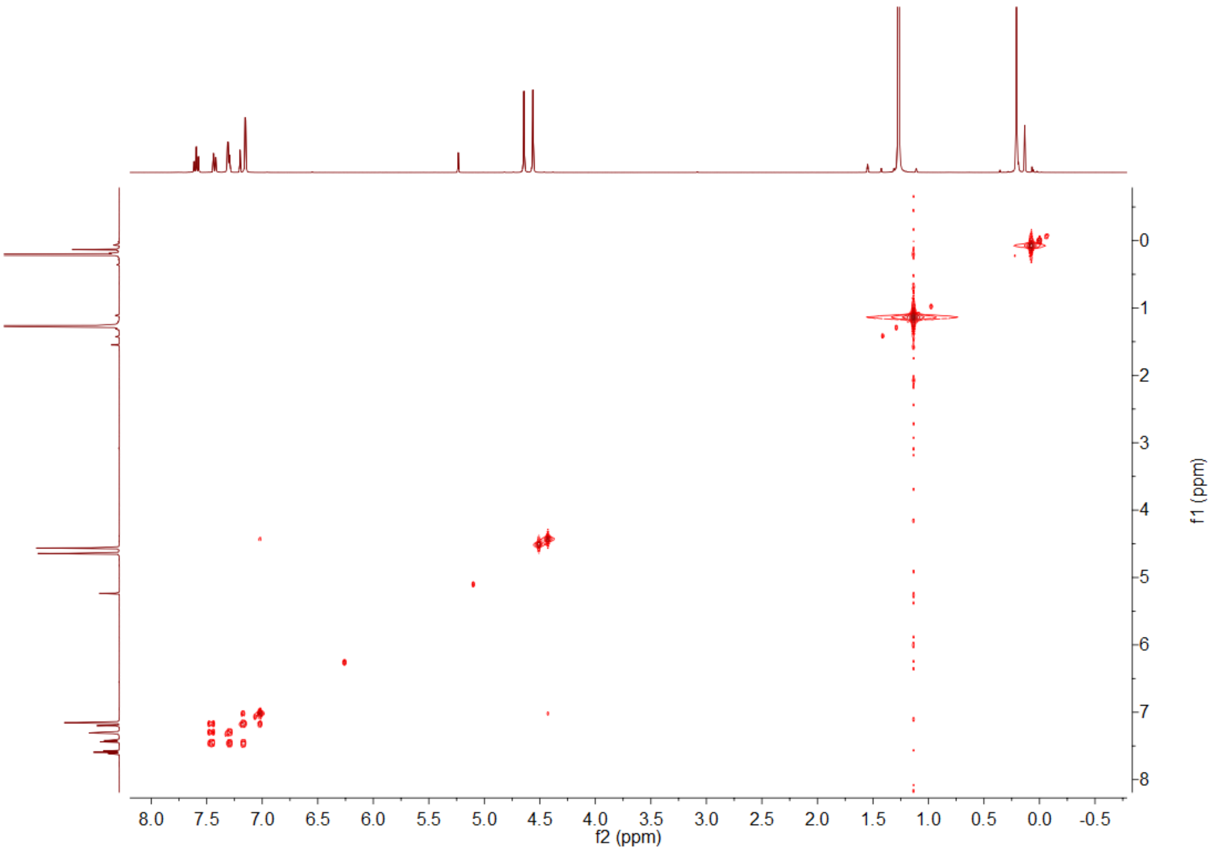

Figure S165 ^{1}H COSY NMR ($CDCl_3$, 400 MHz) of S19.

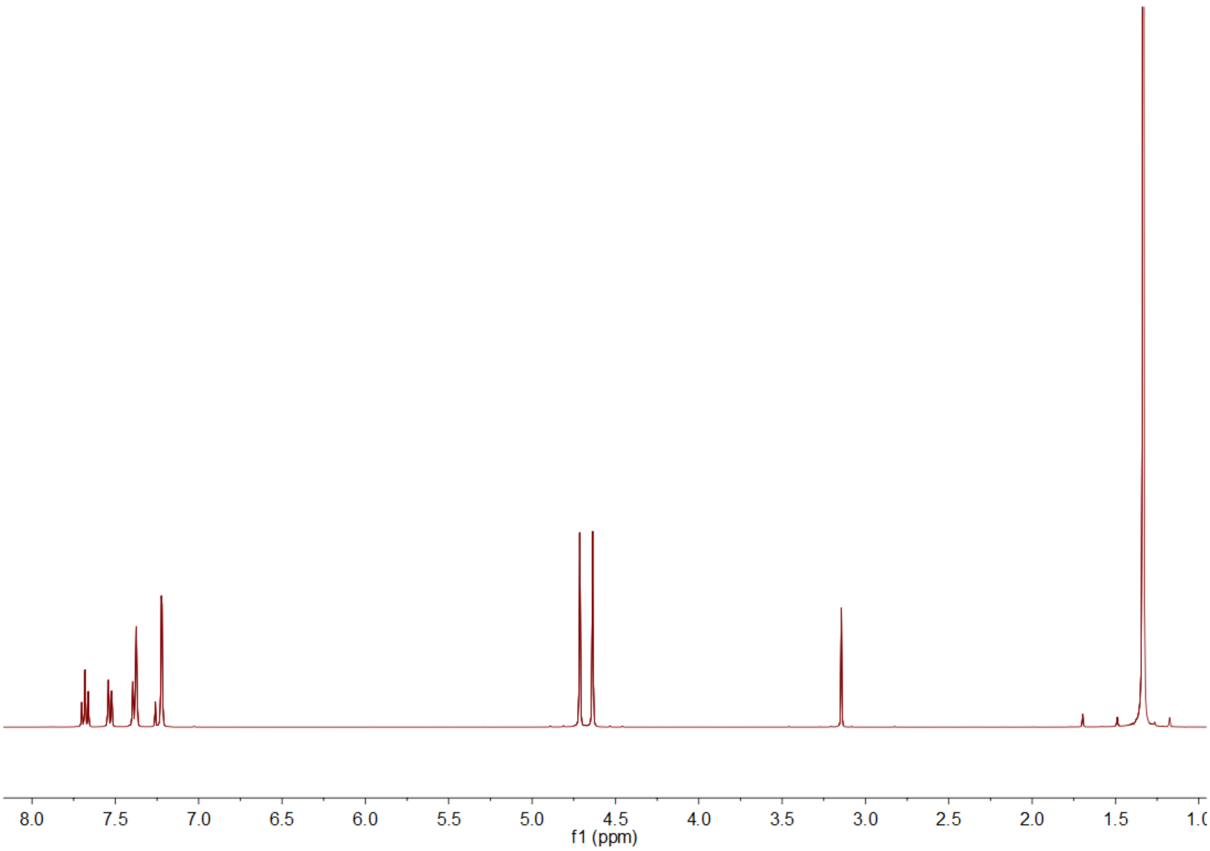

Figure S166 ^{1}H NMR ($CDCl_3$, 400 MHz) of 8.

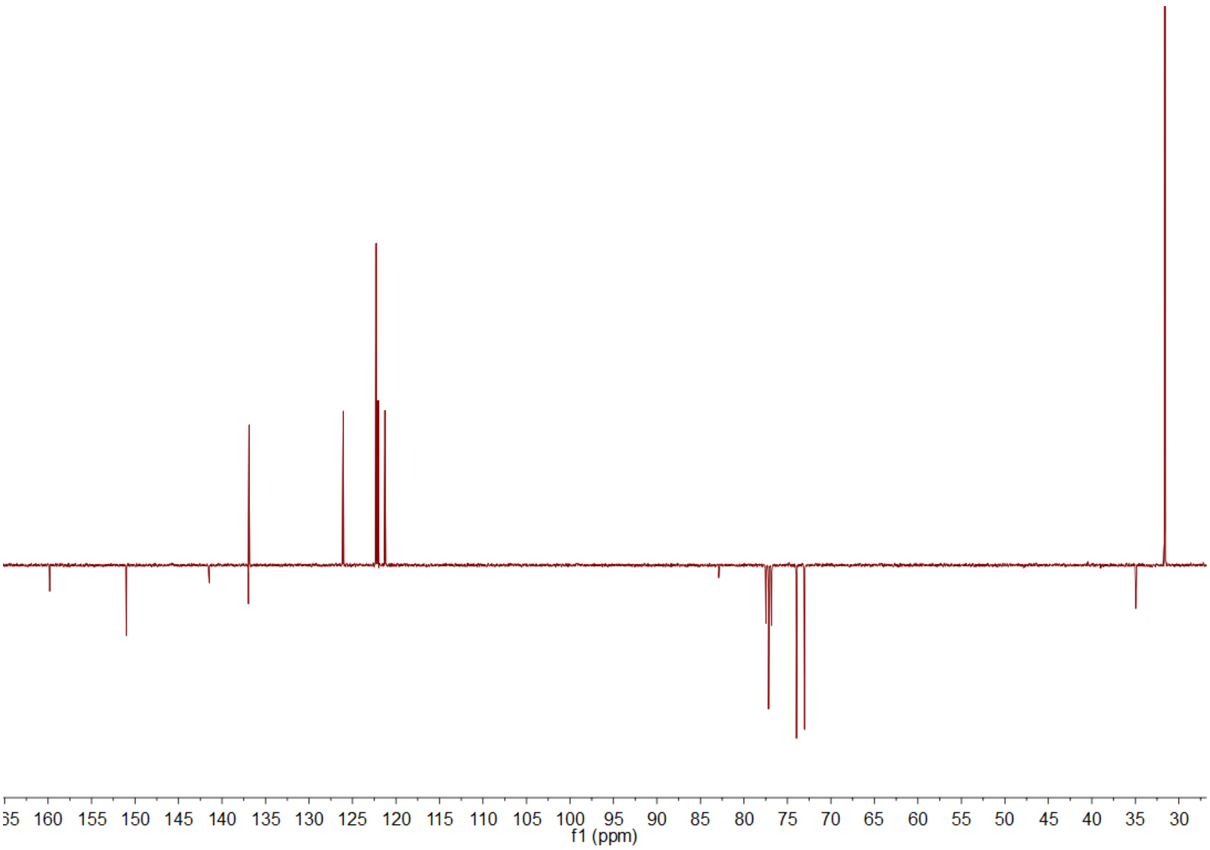

Figure S167 JMOD NMR ($CDCl_3$, 100 MHz) of 8.

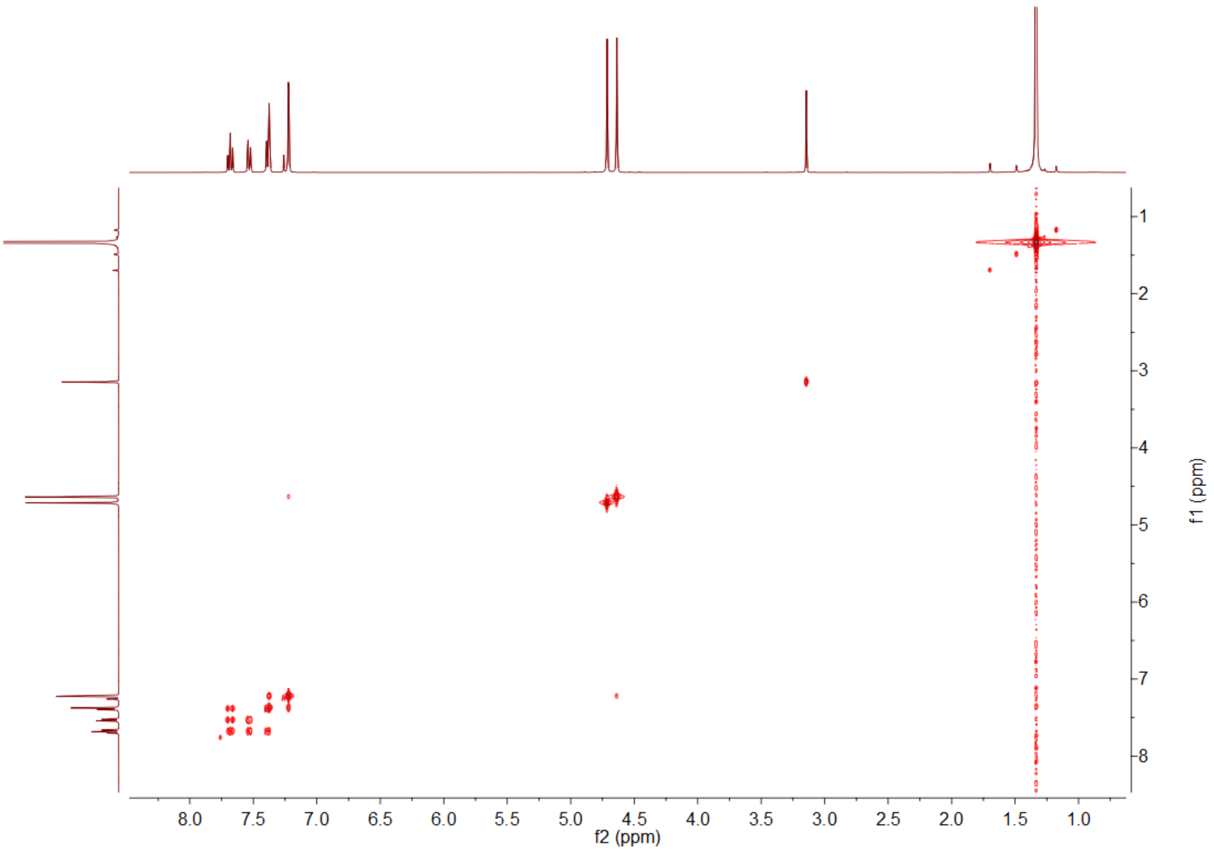

Figure S168 ^{1}H COSY NMR ($CDCl_3$, 400 MHz) of 8.

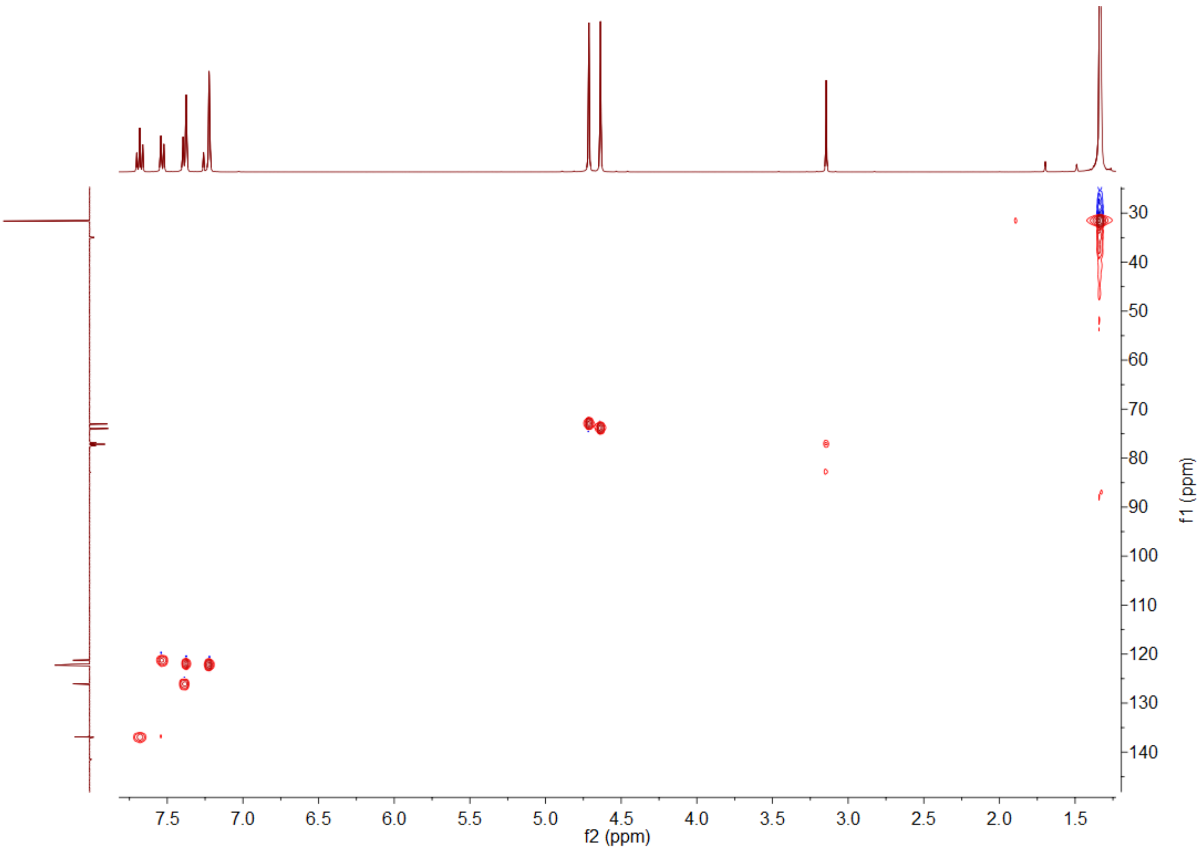

Figure S169 HSQC NMR ($CDCl_3$, 400 MHz) of 8.

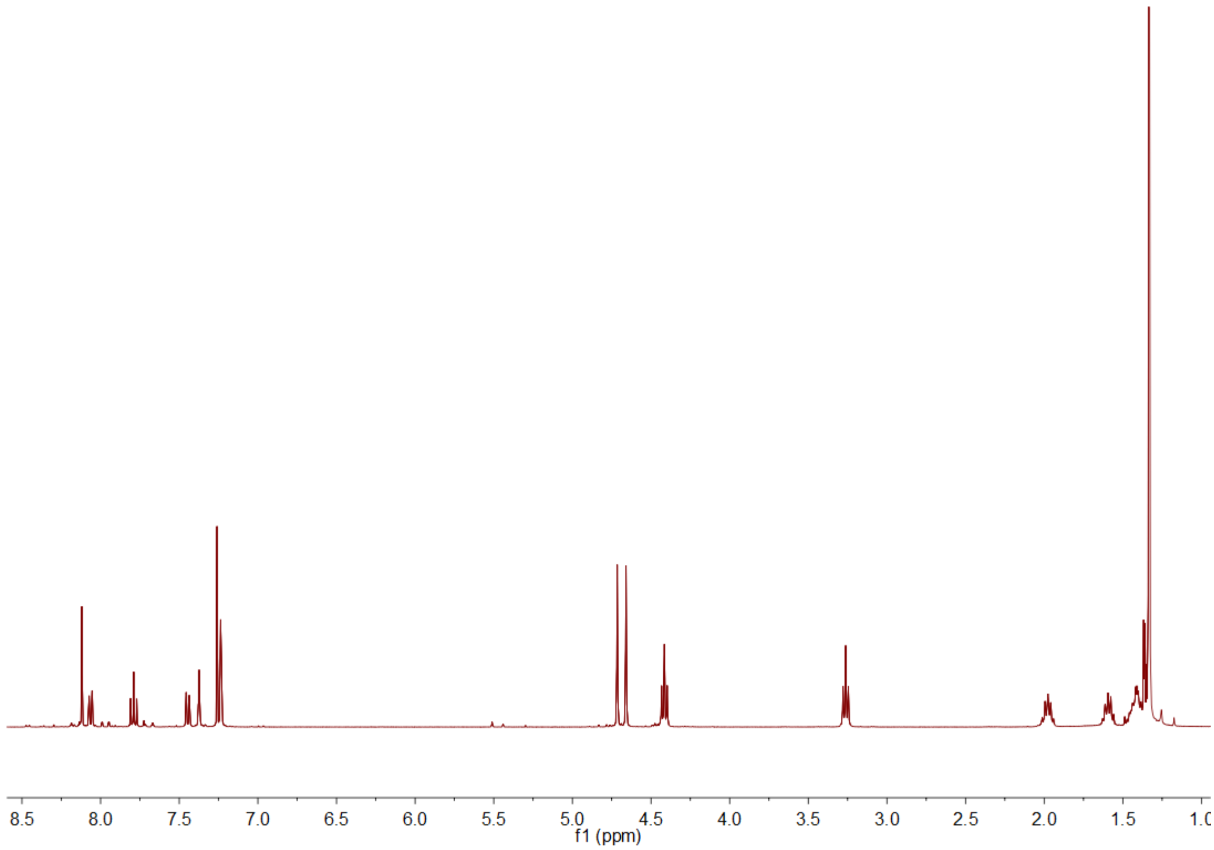

Figure S170 ^{1}H NMR ($CDCl_3$, 400 MHz) of S20.

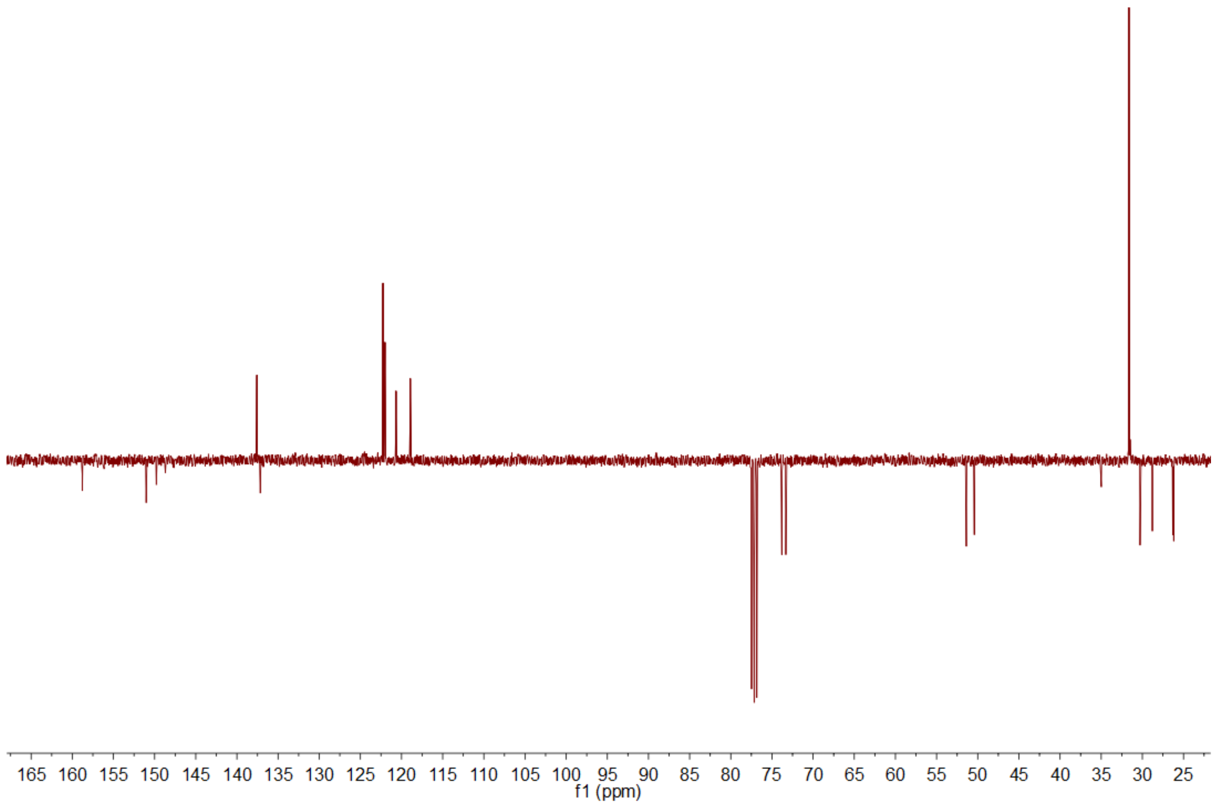

Figure S171 JMOD NMR ($CDCl_3$, 100 MHz) of S20.

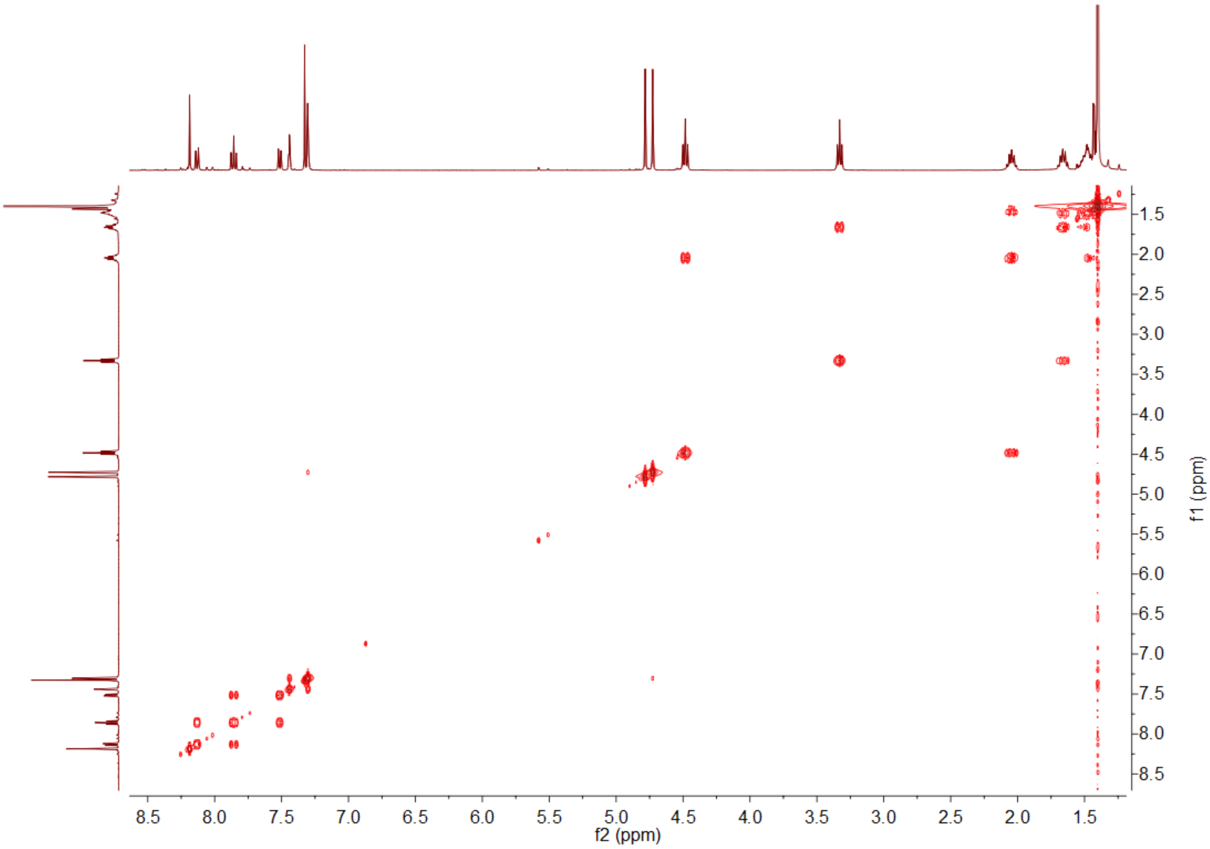

Figure S172 ^{1}H COSY NMR ($CDCl_3$, 400 MHz) of S20.

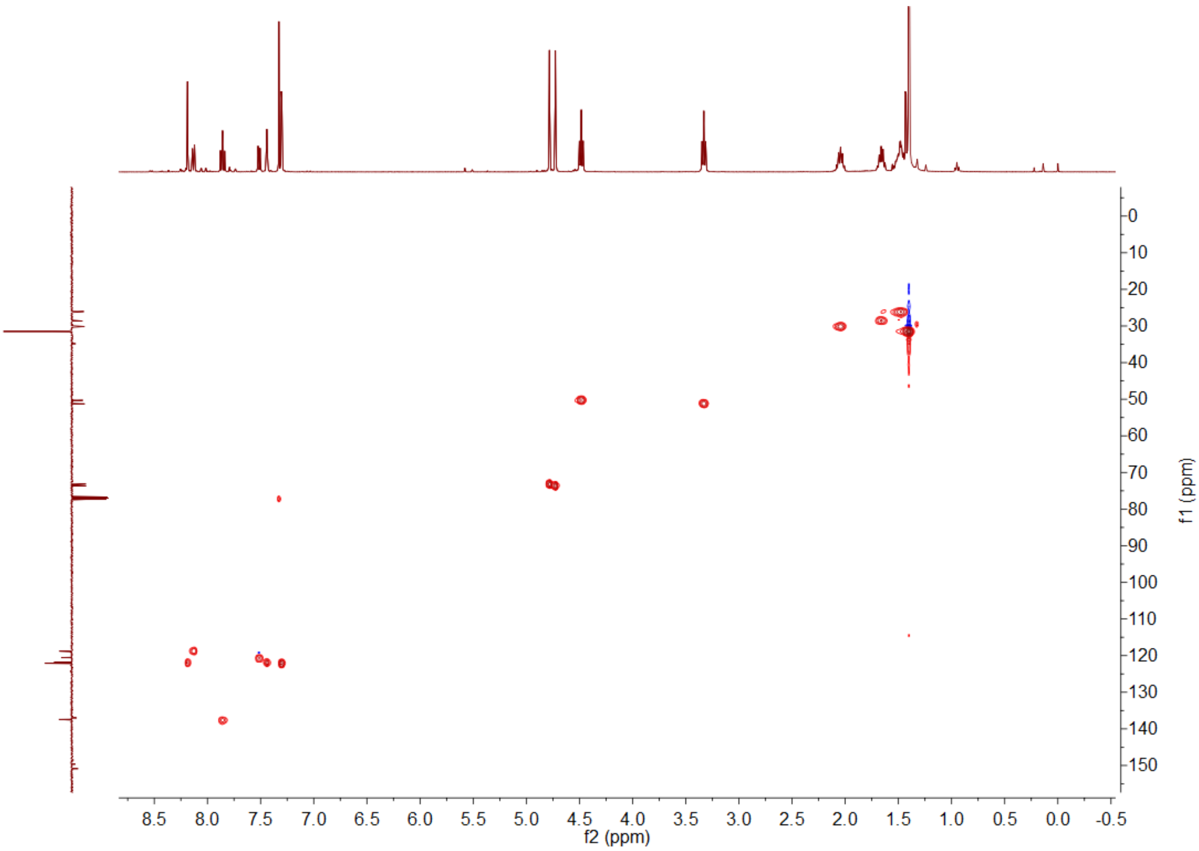

Figure S173 HSQC NMR ($CDCl_3$, 400 MHz) of S20.

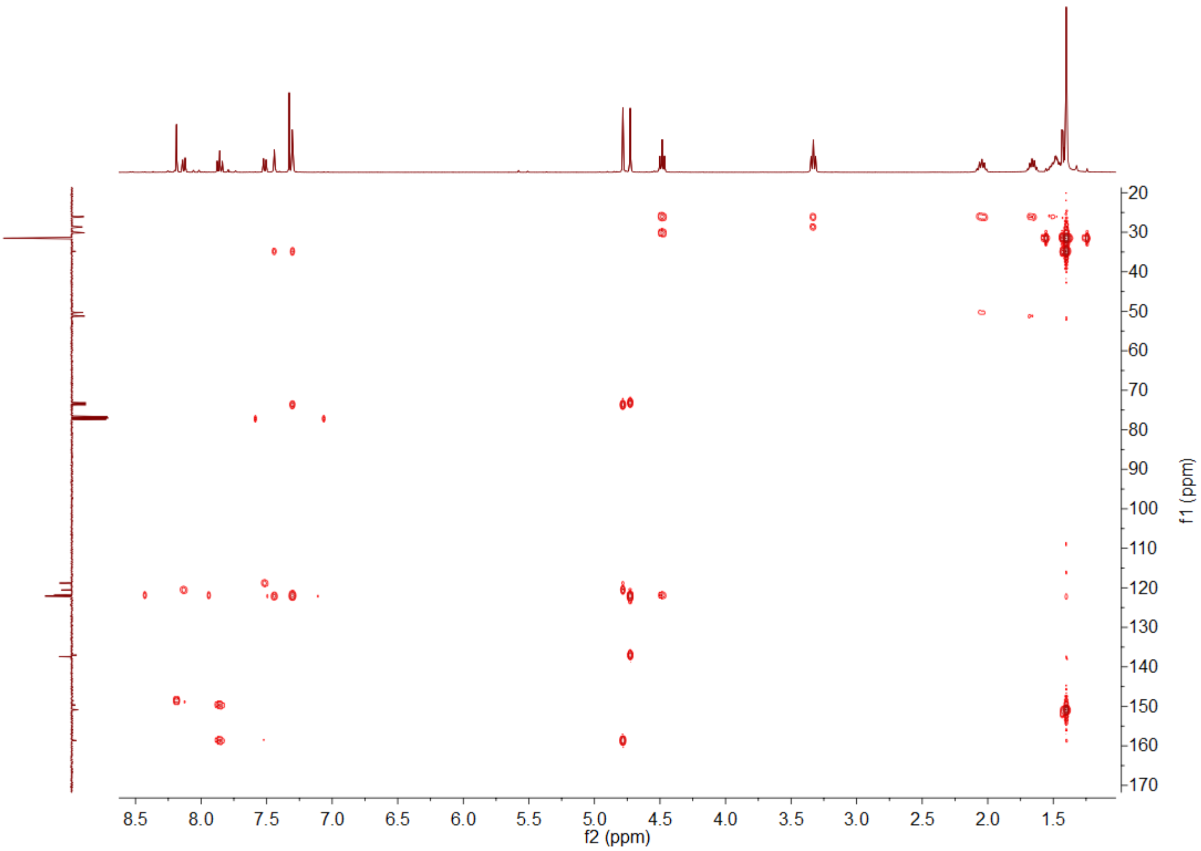

Figure S174 HMBC NMR ($CDCl_3$, 400 MHz) of S20.

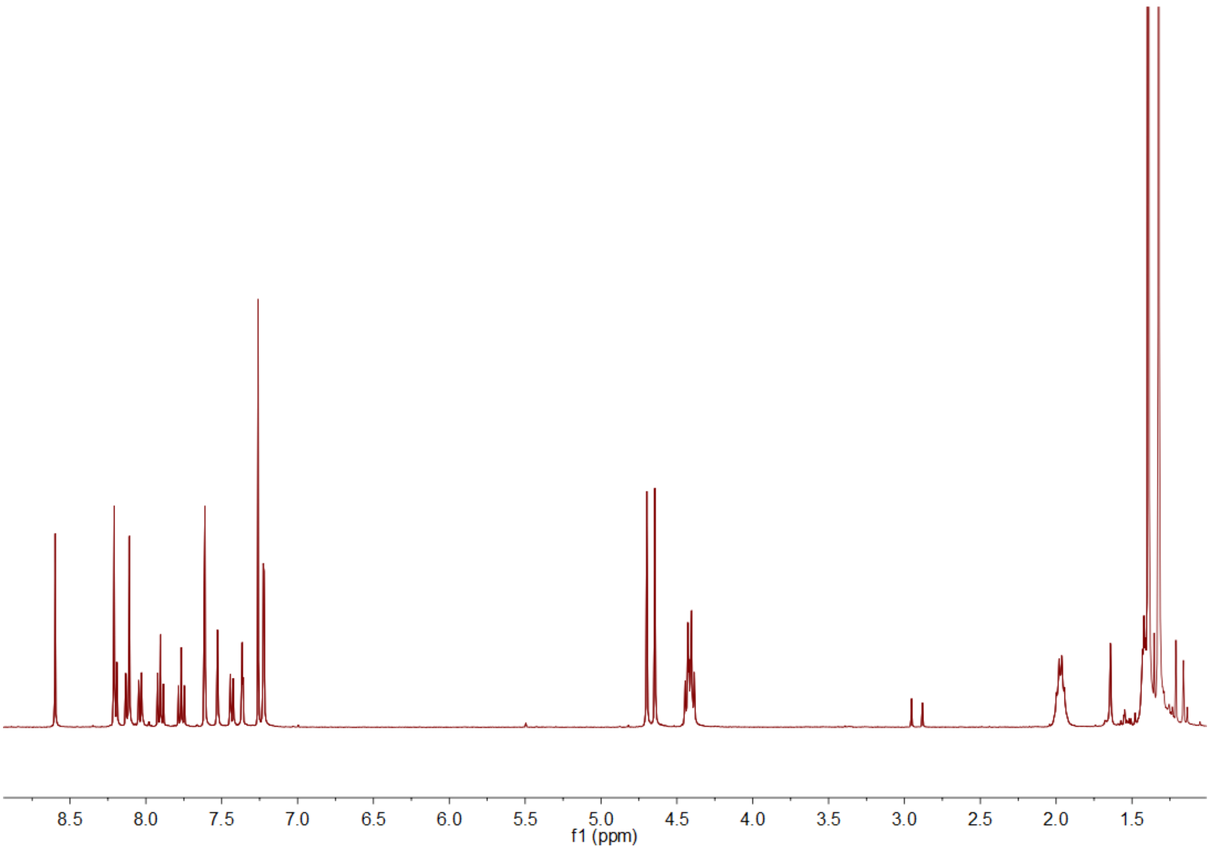

Figure S175 ^{1}H NMR ($CDCl_3$, 400 MHz) of S21.

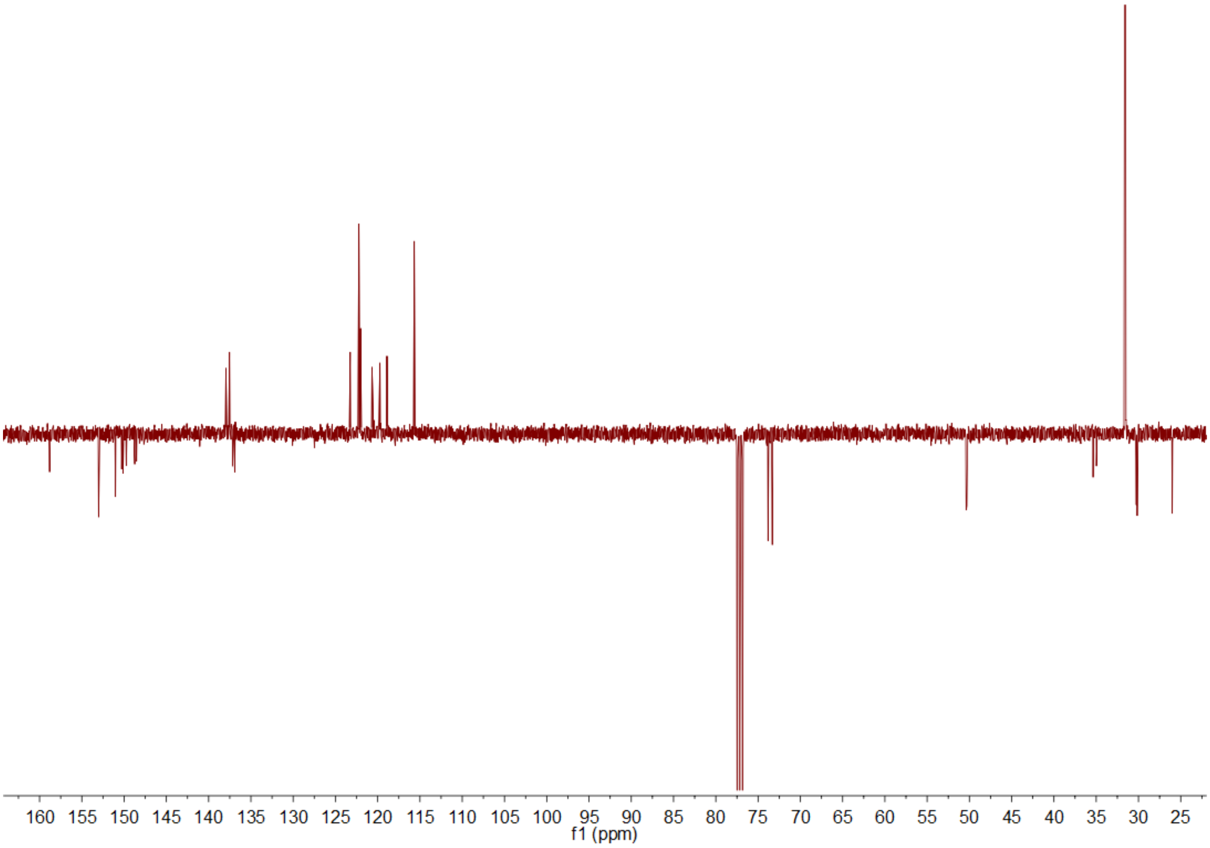

Figure S176 JMOD NMR ($CDCl_3$, 100 MHz) of S21.

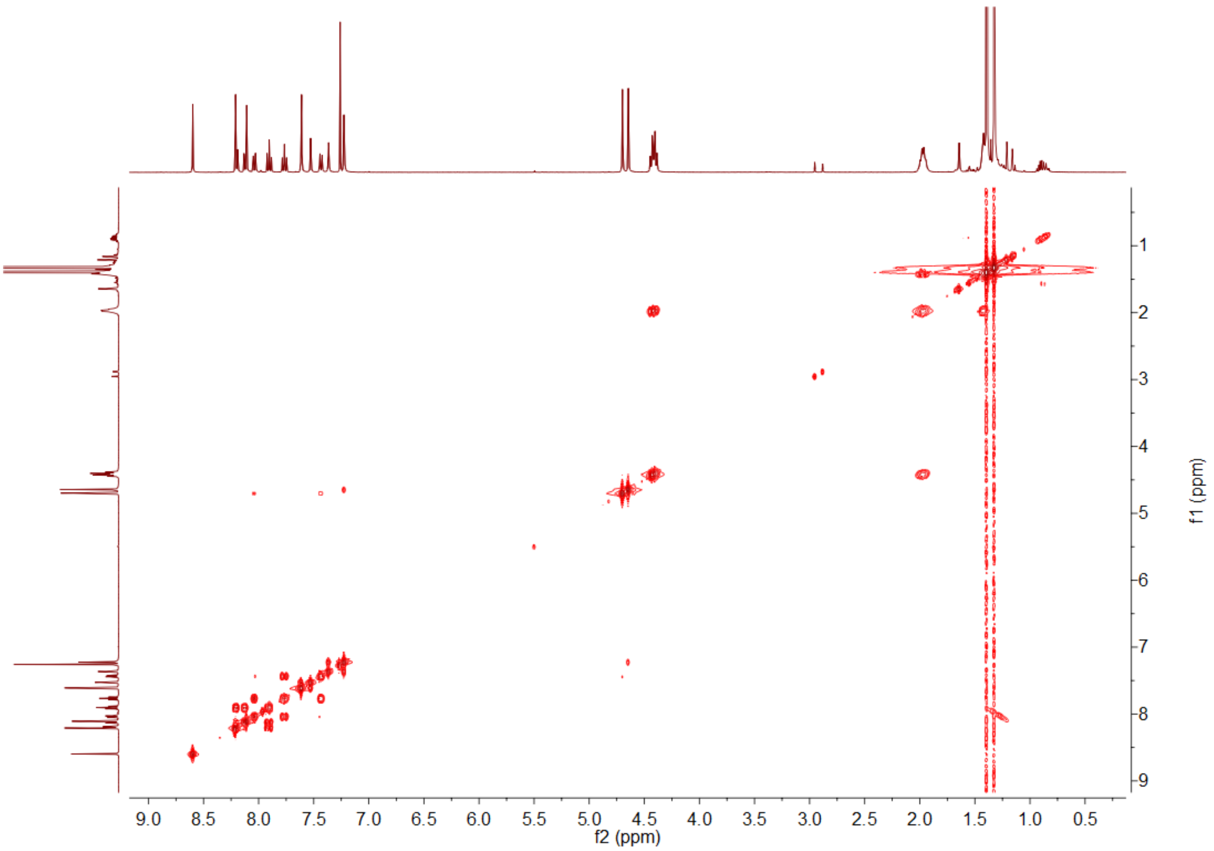

Figure S177 ^{1}H COSY NMR ($CDCl_3$, 400 MHz) of S21.

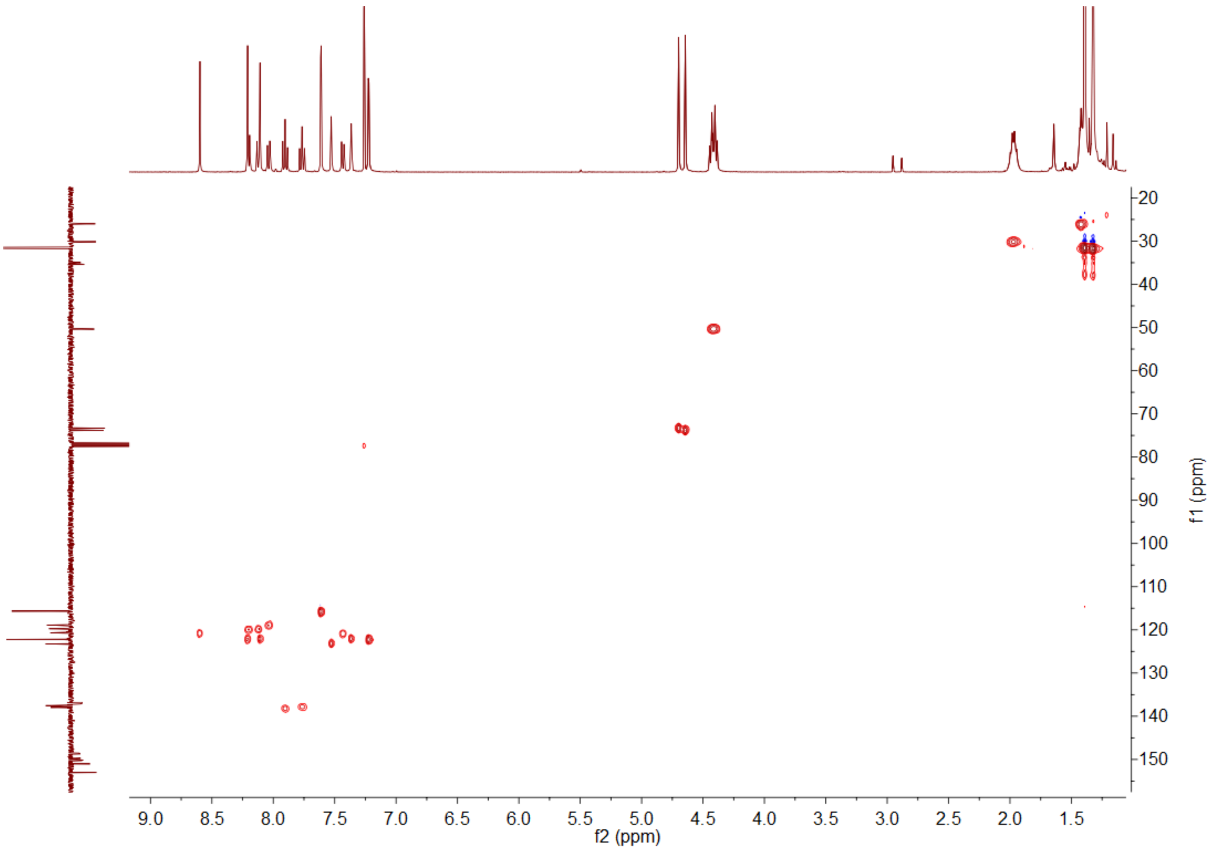

Figure S178 HSQC NMR ($CDCl_3$, 400 MHz) of S21.

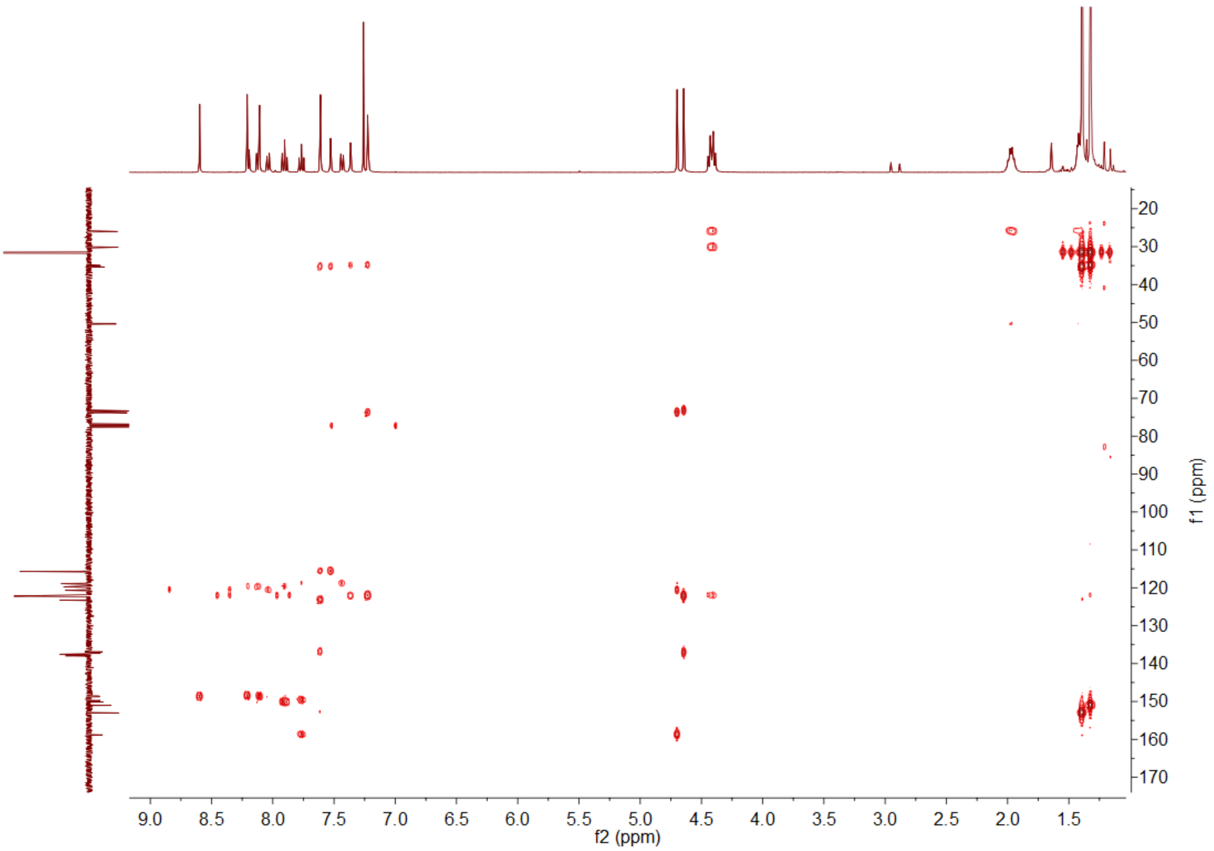

Figure S179 HMBC NMR ($CDCl_3$, 400 MHz) of S21.

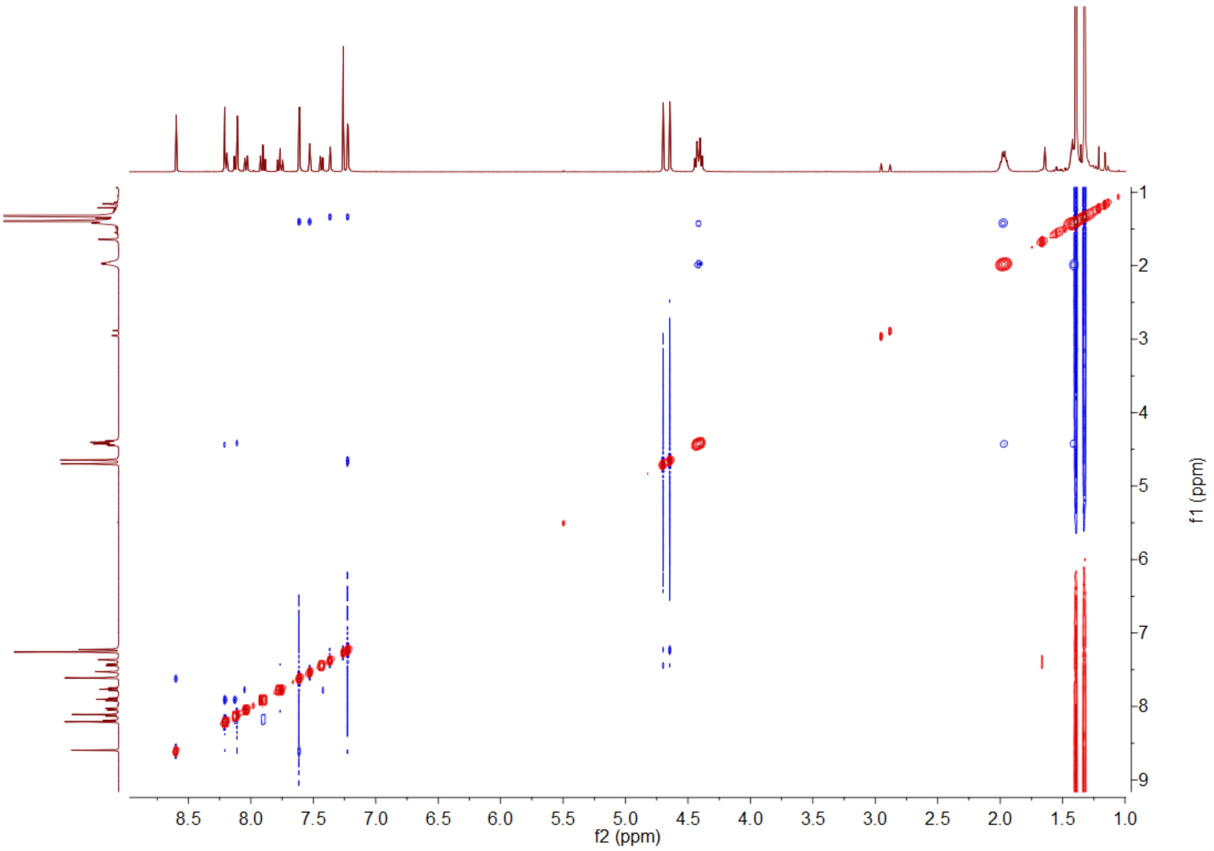

Figure S180 ^{1}H NOESY NMR ($CDCl_3$, 400 MHz) of S21.

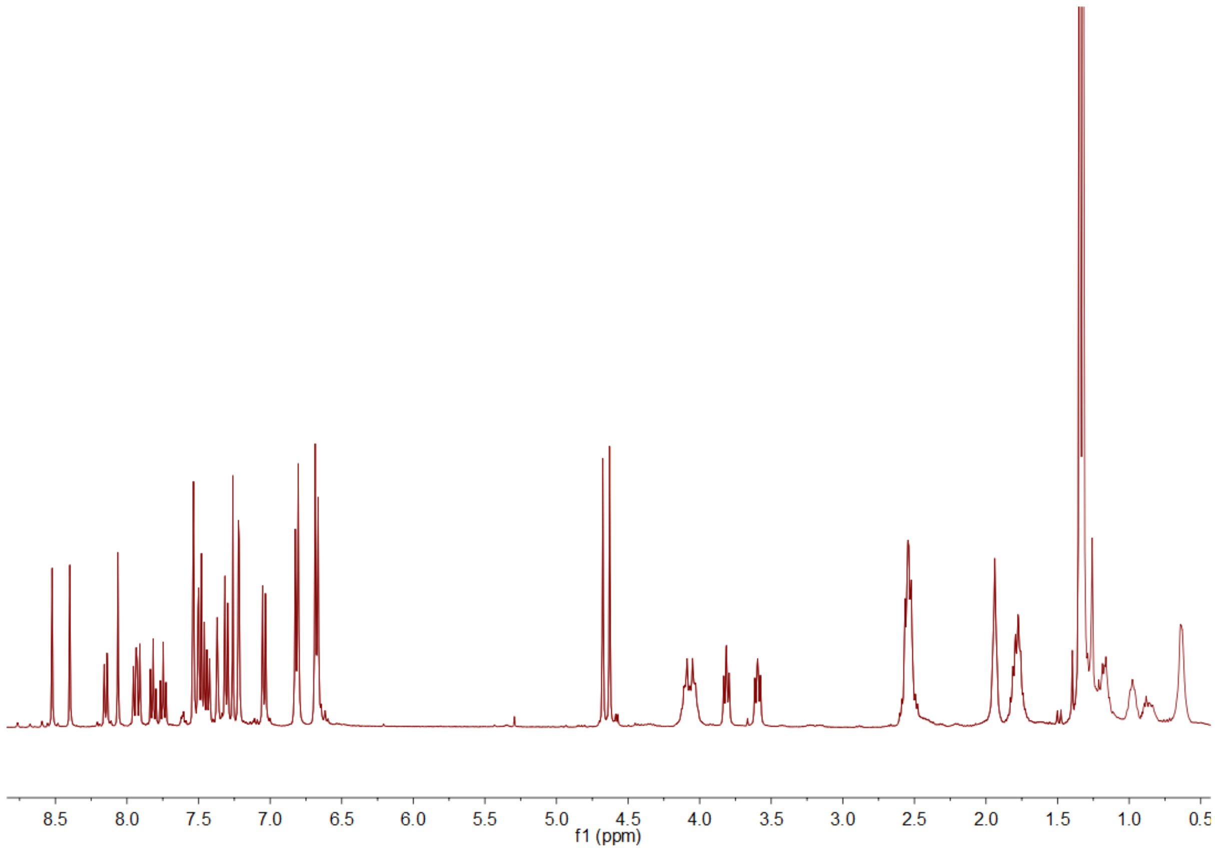

Figure S181 ^{1}H NMR ($CDCl_3$, 400 MHz) of 9a.

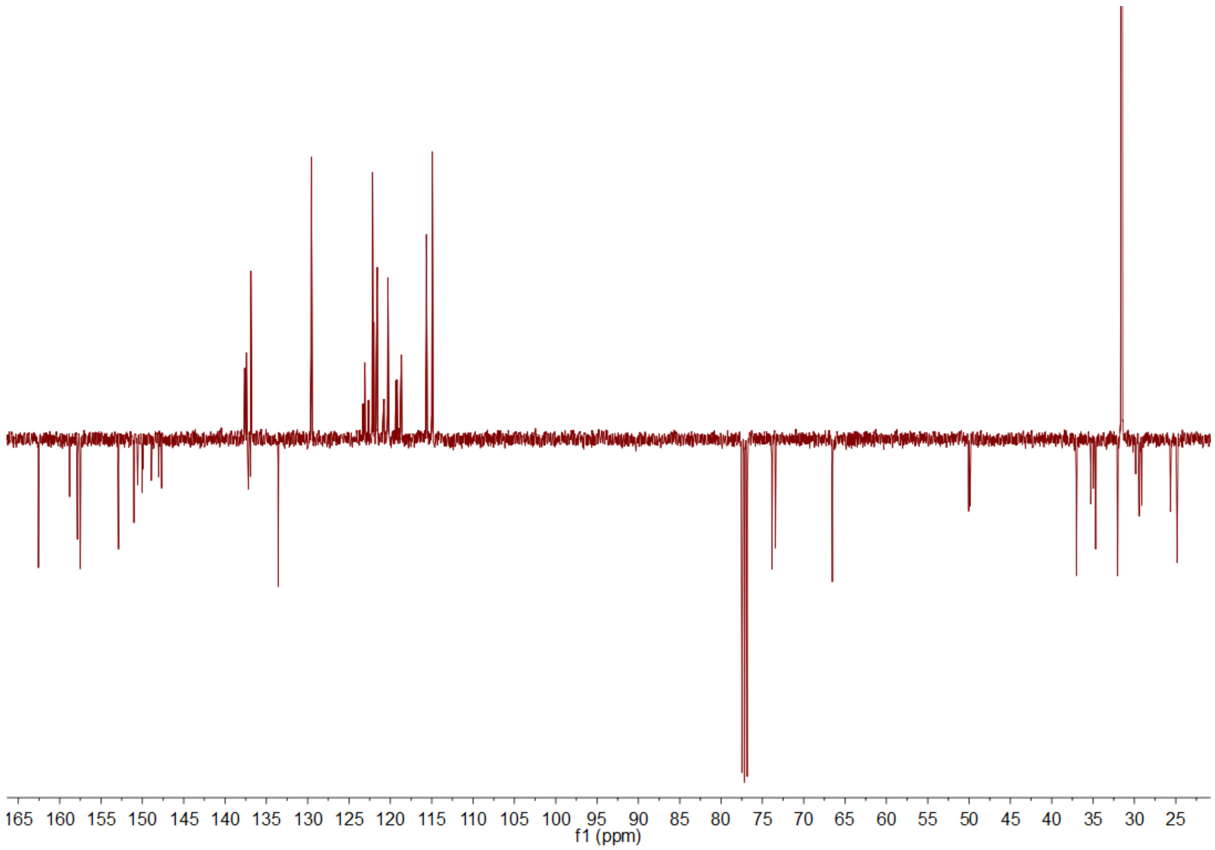

Figure S182 JMOD NMR ($CDCl_3$, 100 MHz) of 9a.

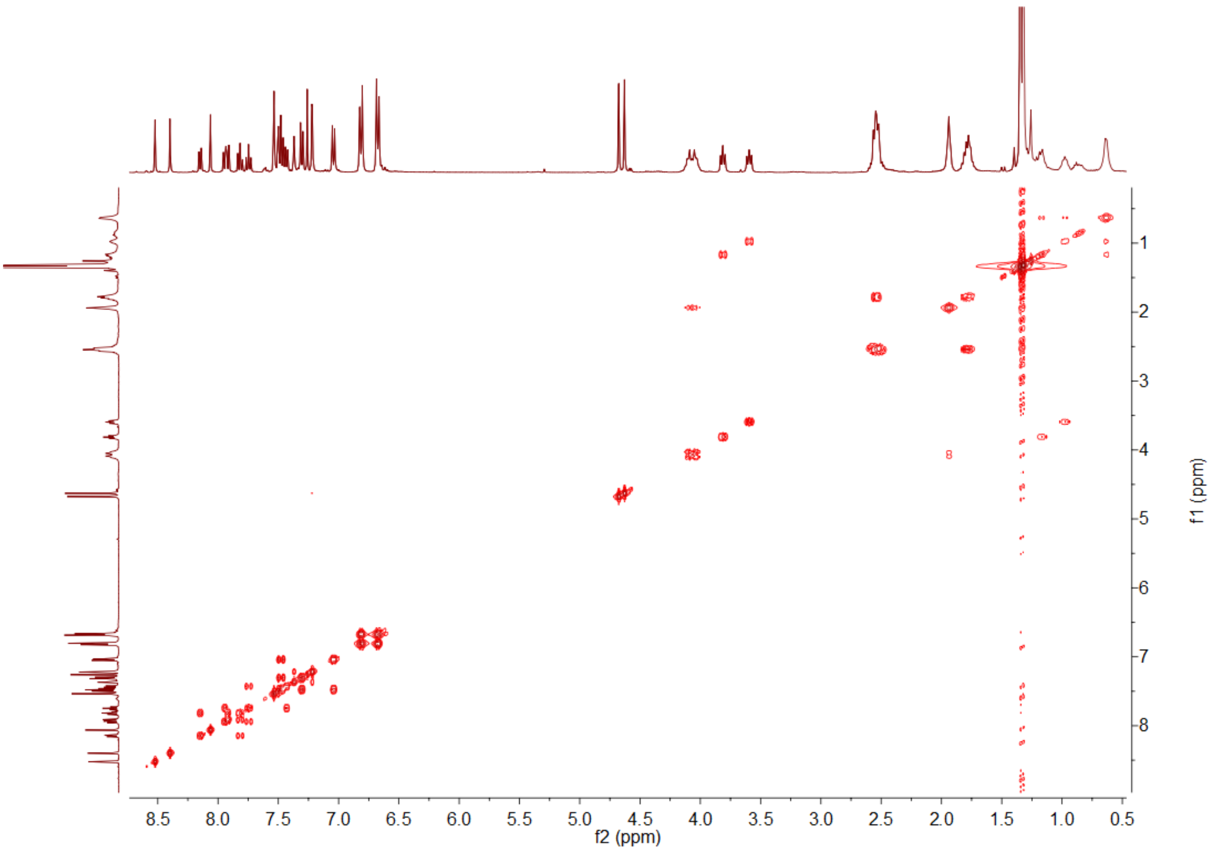

Figure S183 ^{1}H COSY NMR ($CDCl_3$, 400 MHz) of 9a.

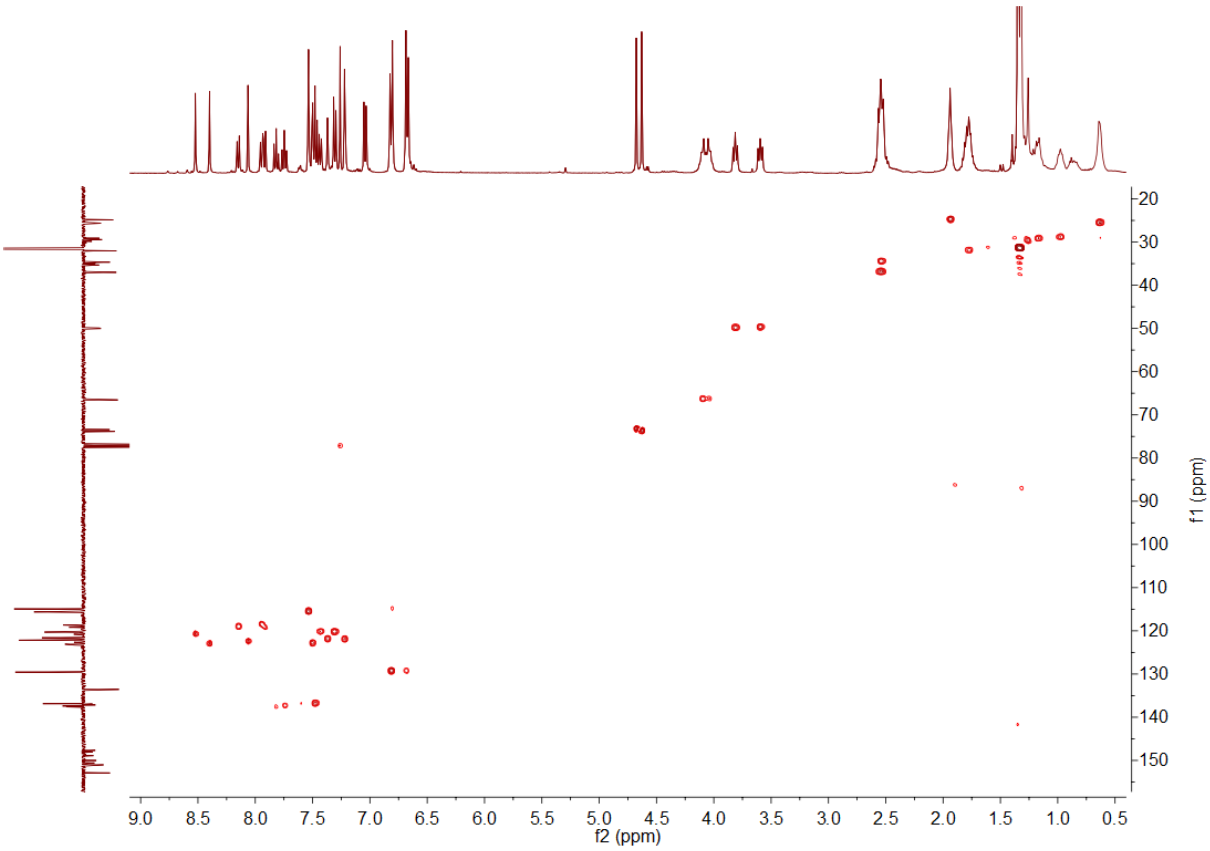

Figure S184 HSQC NMR ($CDCl_3$, 400 MHz) of 9a.

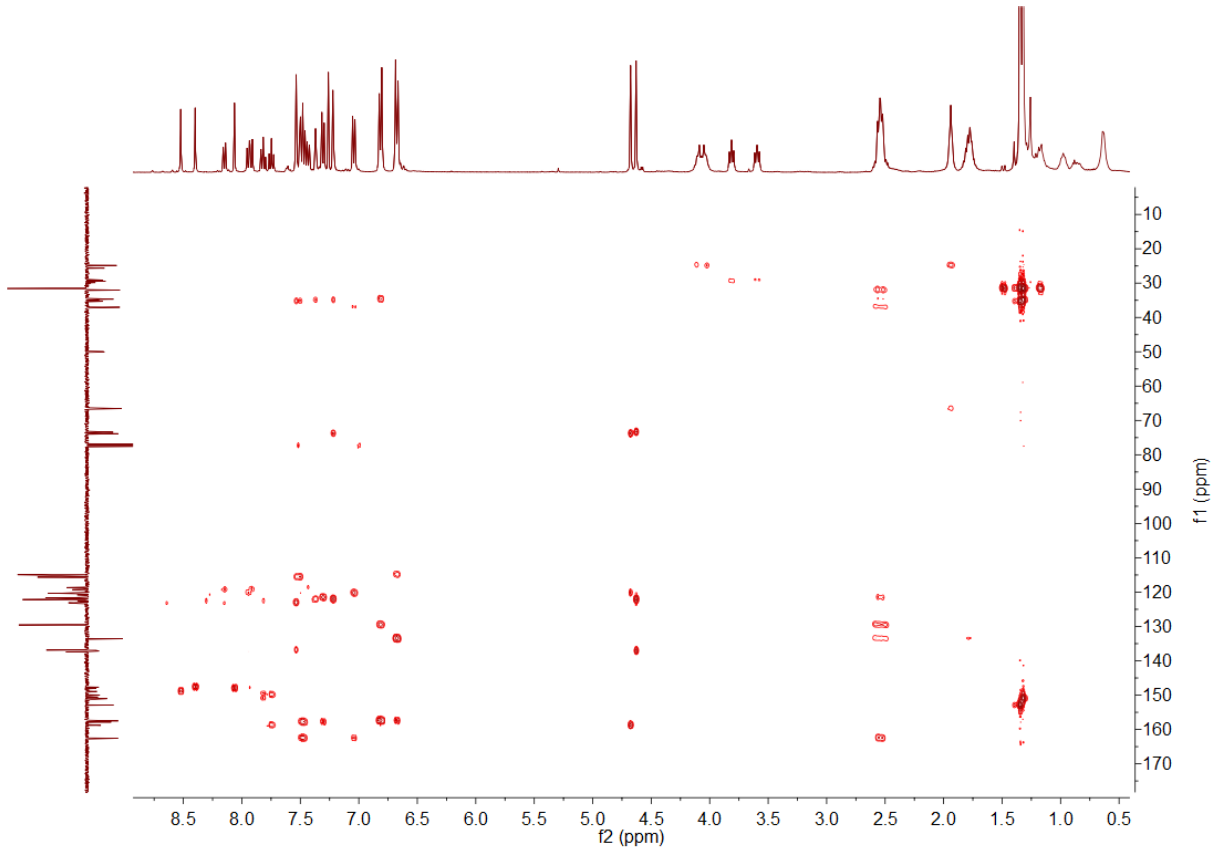

Figure S185 HMBC NMR ($CDCl_3$, 400 MHz) of 9a.

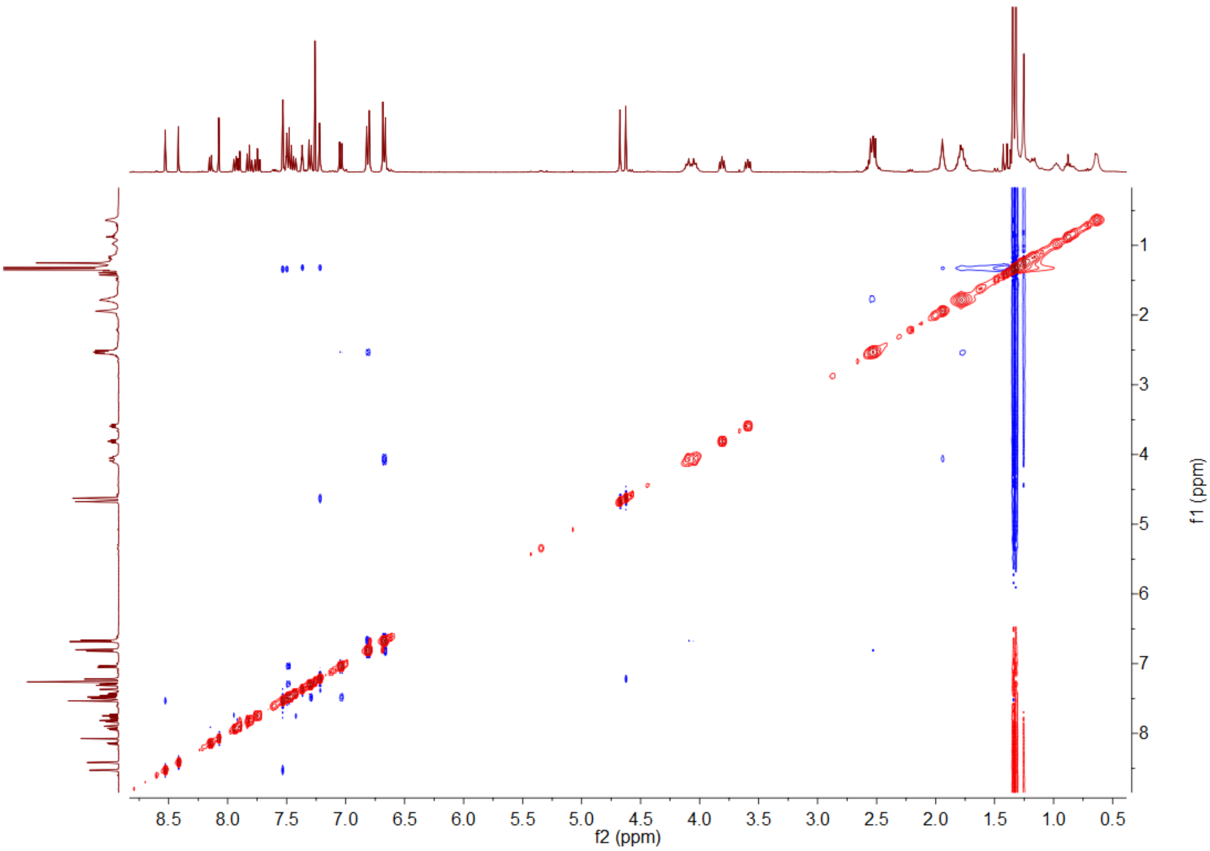

Figure S186 ^{1}H ROESY NMR ($CDCl_3$, 400 MHz) of 9a.

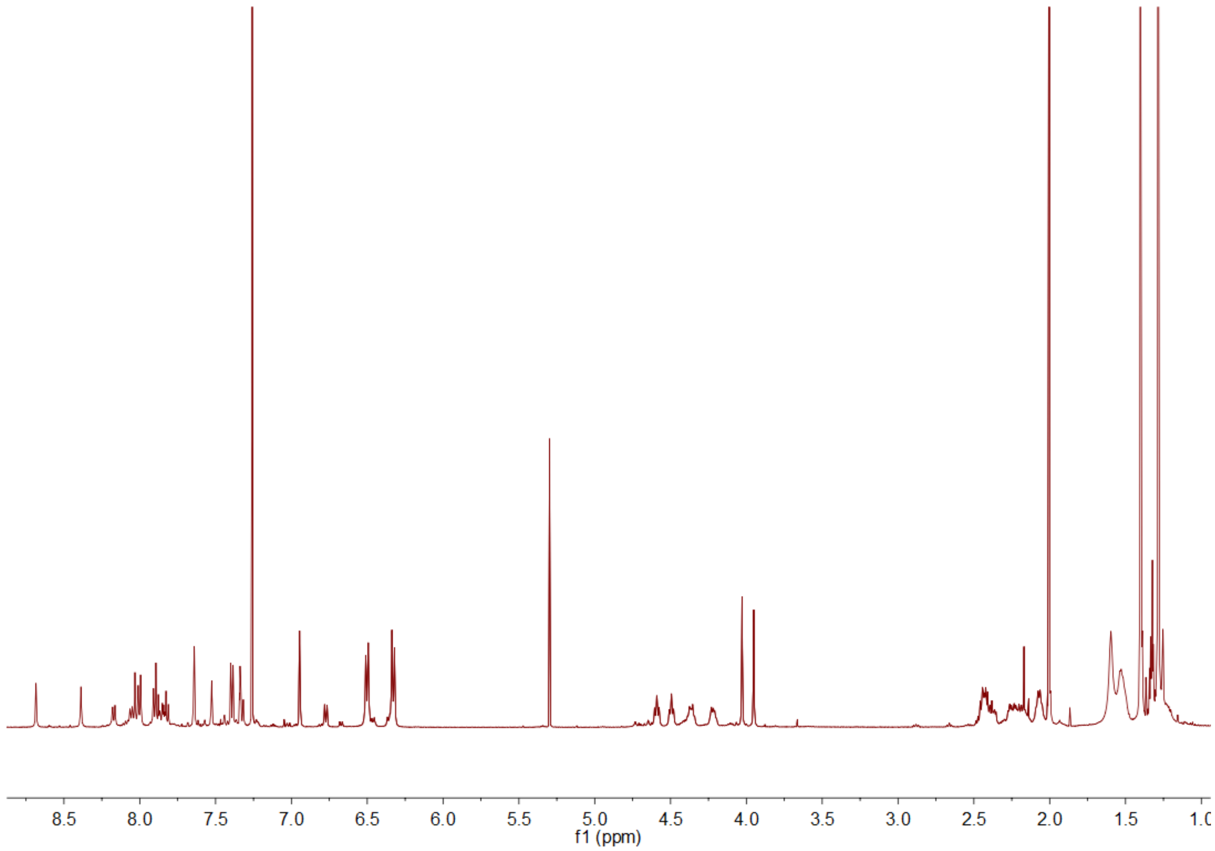

Figure S187 ^{1}H NMR ($CDCl_3$, 500 MHz) of [Cu(9a)](PF_6).

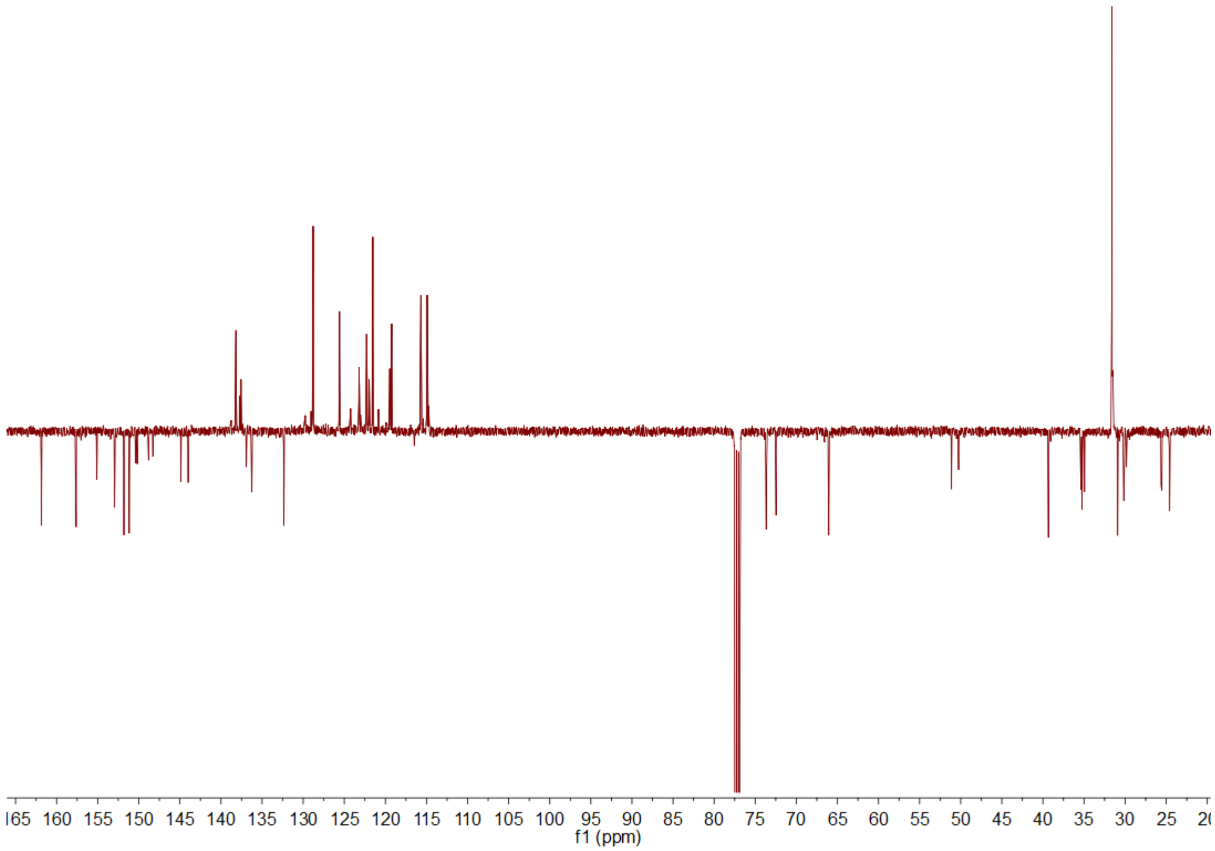

Figure S188 JMOD NMR ($CDCl_3$, 125 MHz) of [Cu(9a)](PF_6).

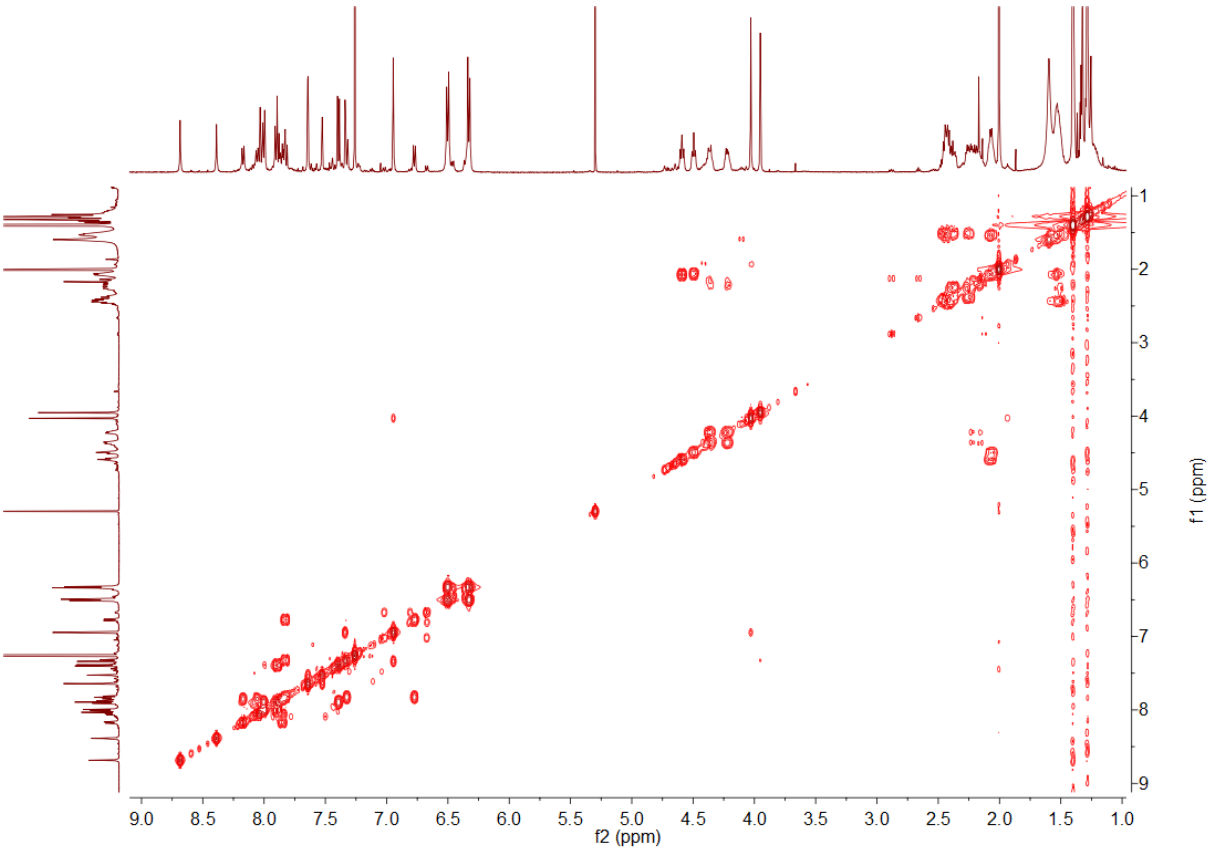

Figure S189 ^{1}H COSY NMR ($CDCl_3$, 500 MHz) of [Cu(9a)](PF_6).

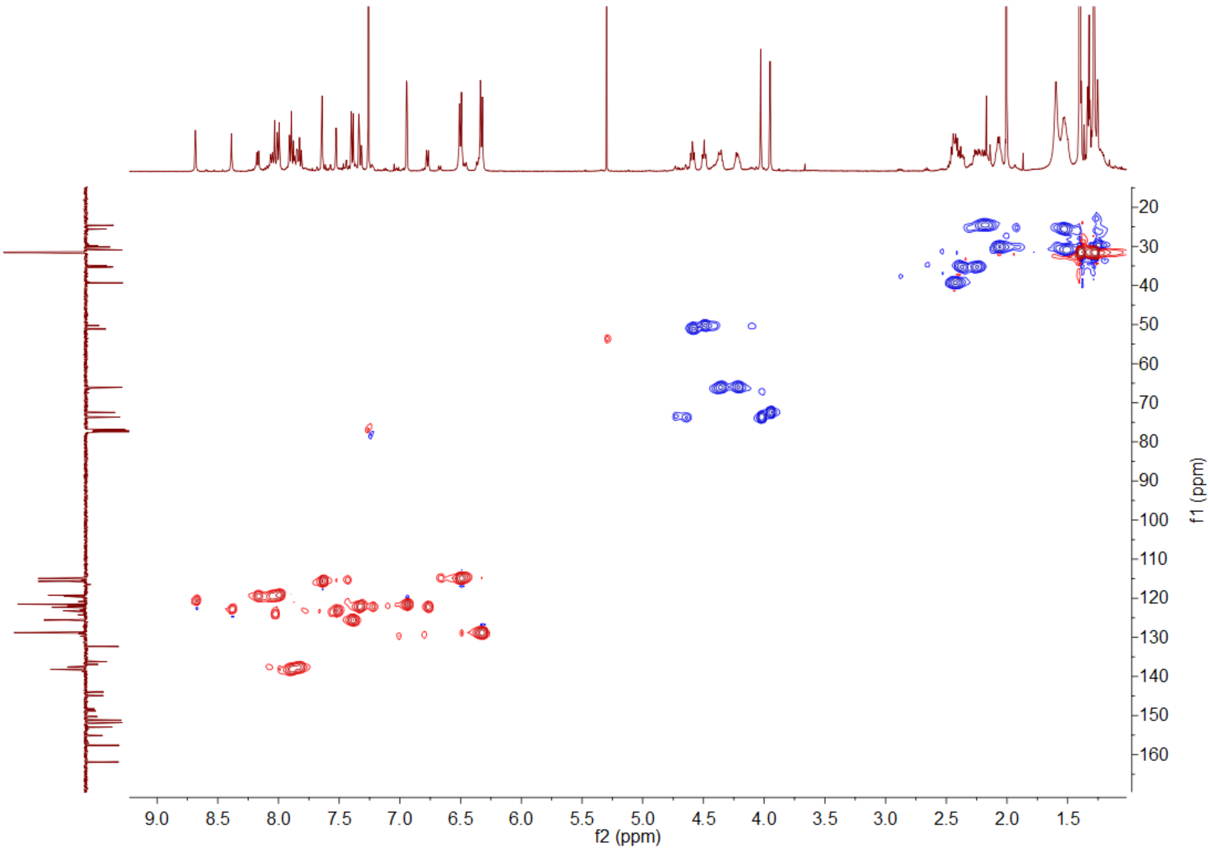

Figure S190 HSQC NMR ($CDCl_3$, 500 MHz) of [Cu(9a)](PF_6).

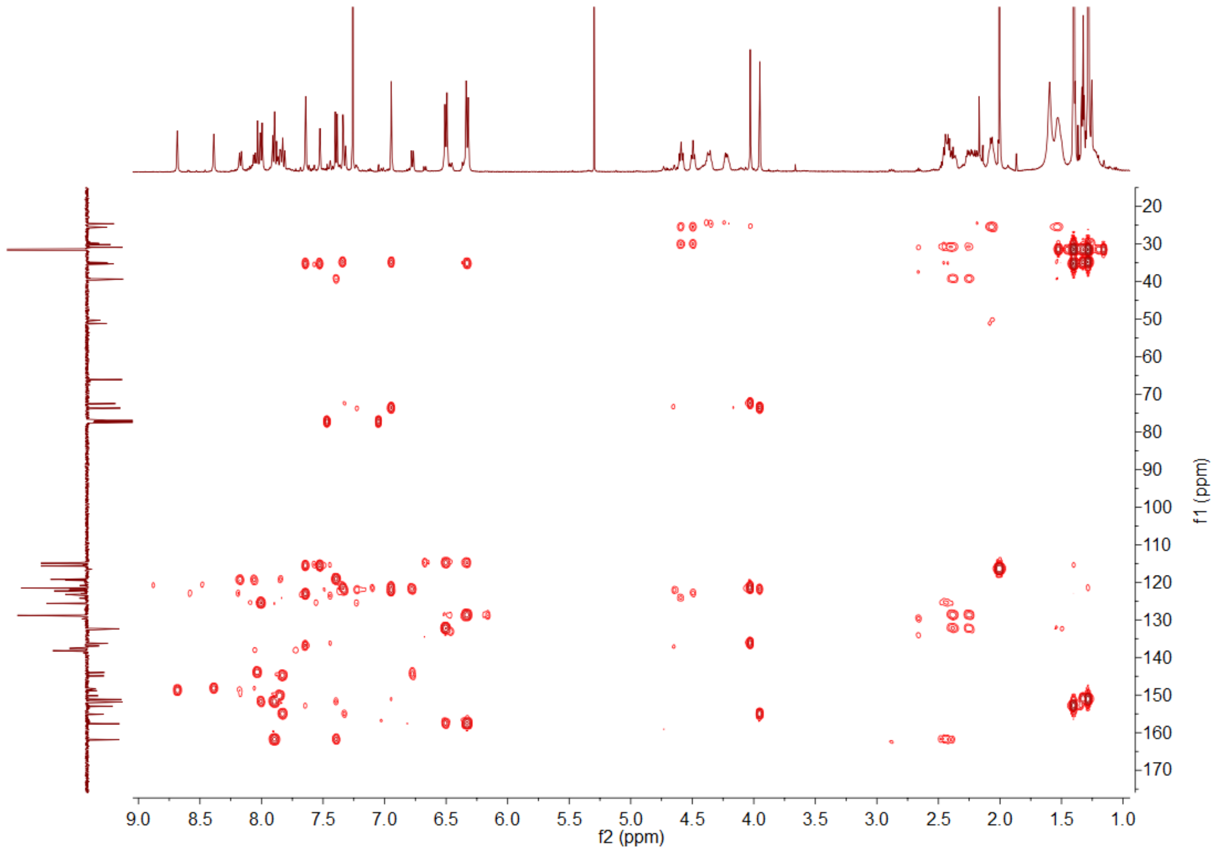

Figure S191 HMBC NMR ($CDCl_3$, 500 MHz) of [Cu(9a)](PF_6).

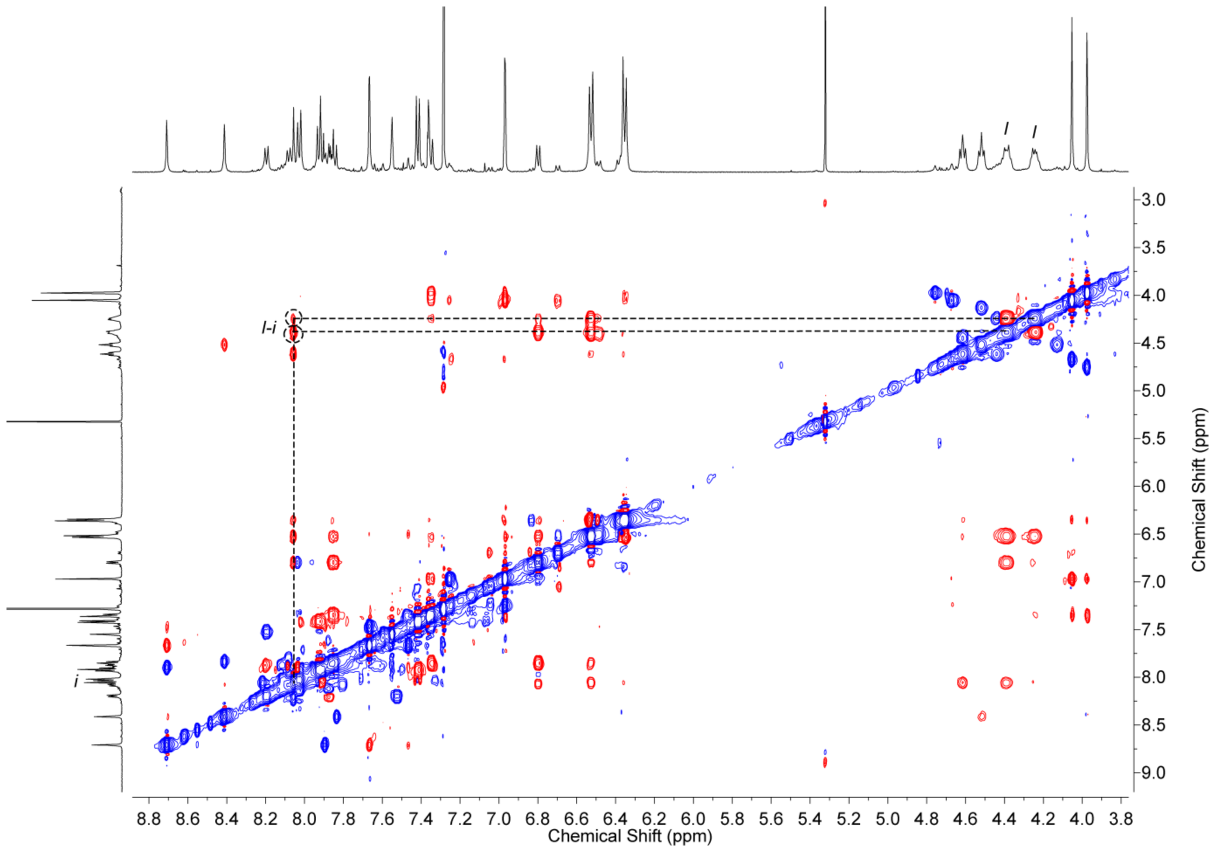

Figure S192 ^{1}H ROESY NMR ($CDCl_3$, 500 MHz) of [Cu(9a)](PF_6).

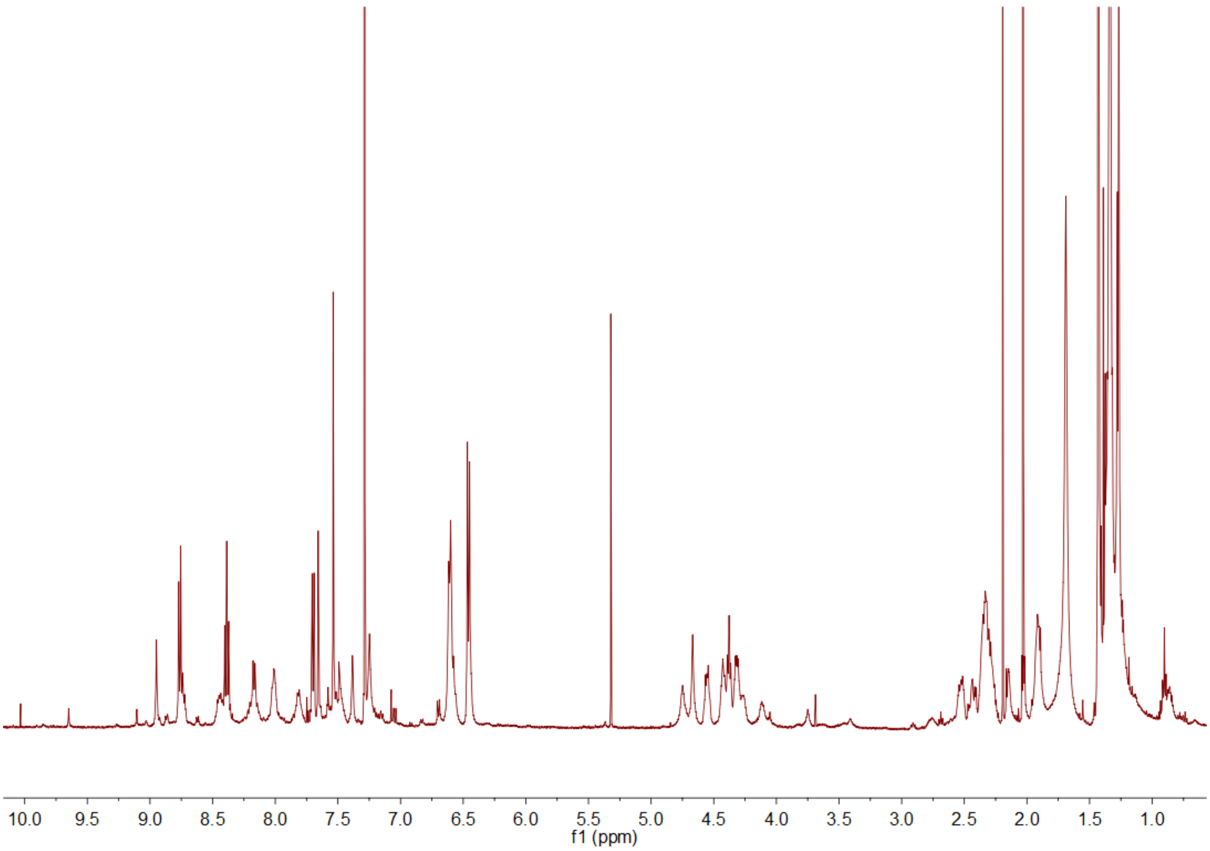

Figure S193 ^{1}H NMR ($CDCl_3$, 500 MHz) of [Zn(9a)]($OTf)_2$.

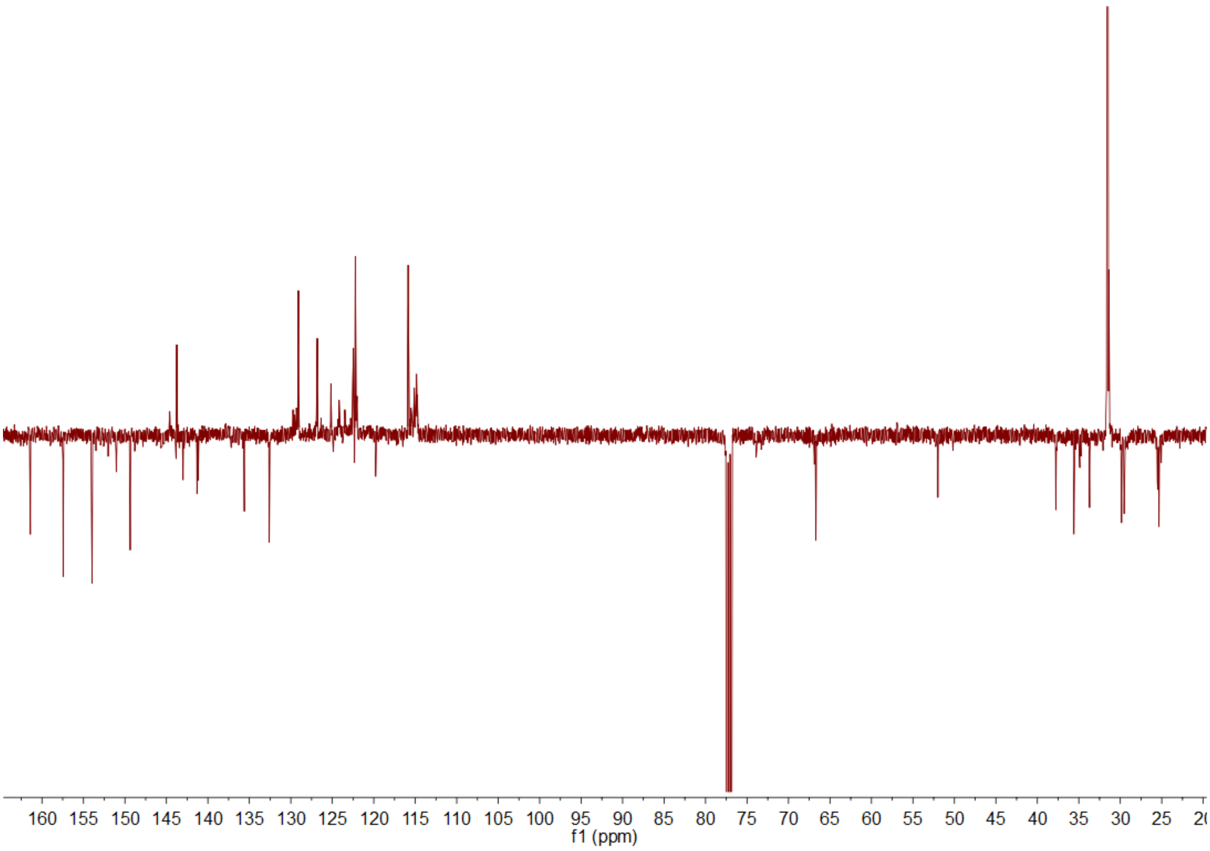

Figure S194 JMOD NMR ($CDCl_3$, 125 MHz) of [Zn(9a)]($OTf)_2$.

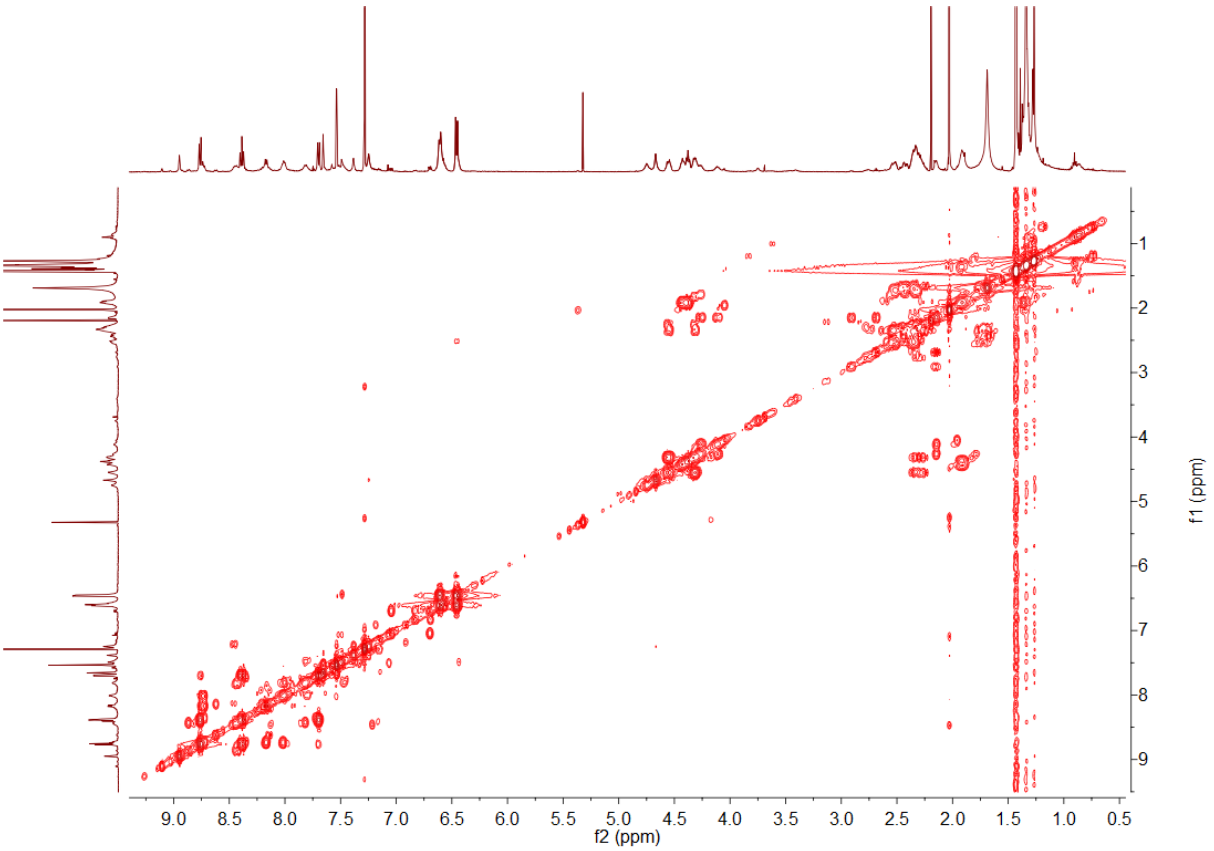

Figure S195 ¹H COSY NMR ($CDCl_3$, 500 MHz) of [Zn(9a)]($OTf)_2$.

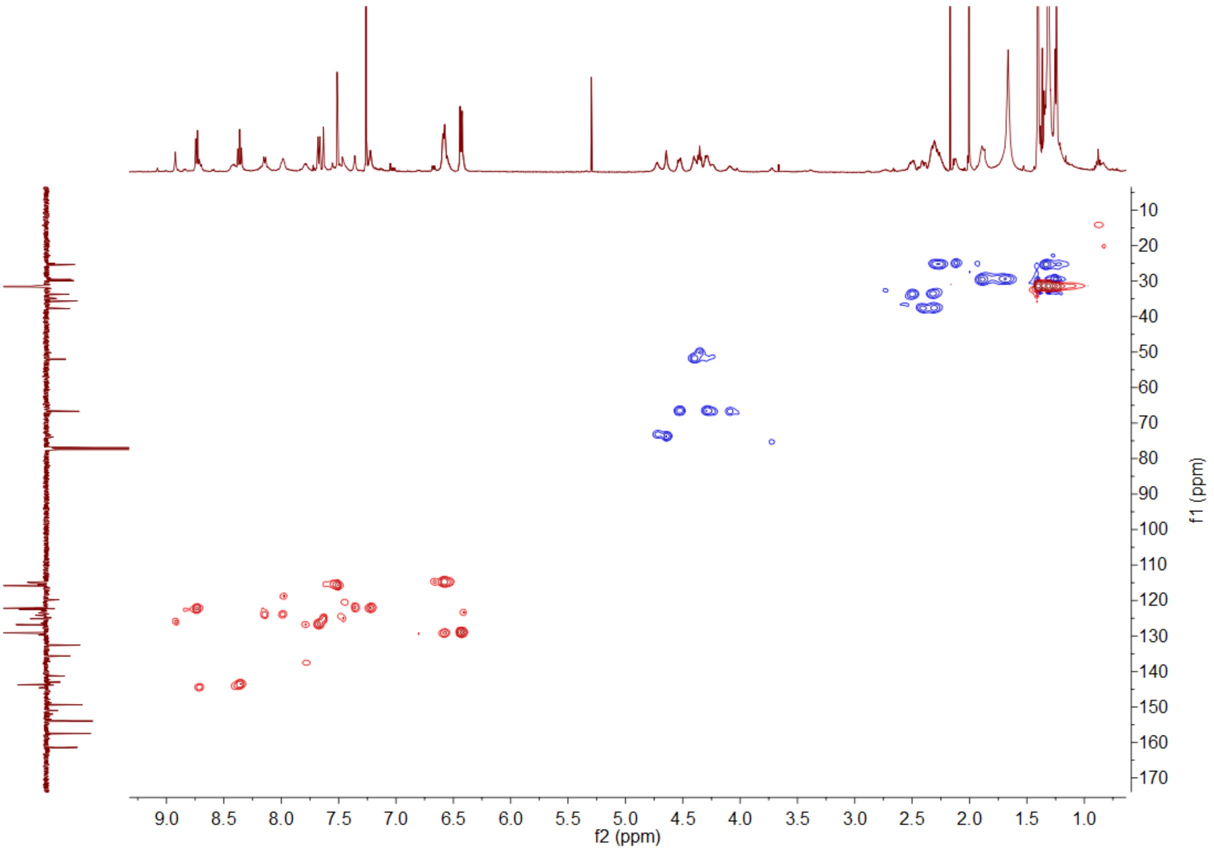

Figure S196 HSQC NMR ($CDCl_3$, 500 MHz) of [Zn(9a)]($OTf)_2$.

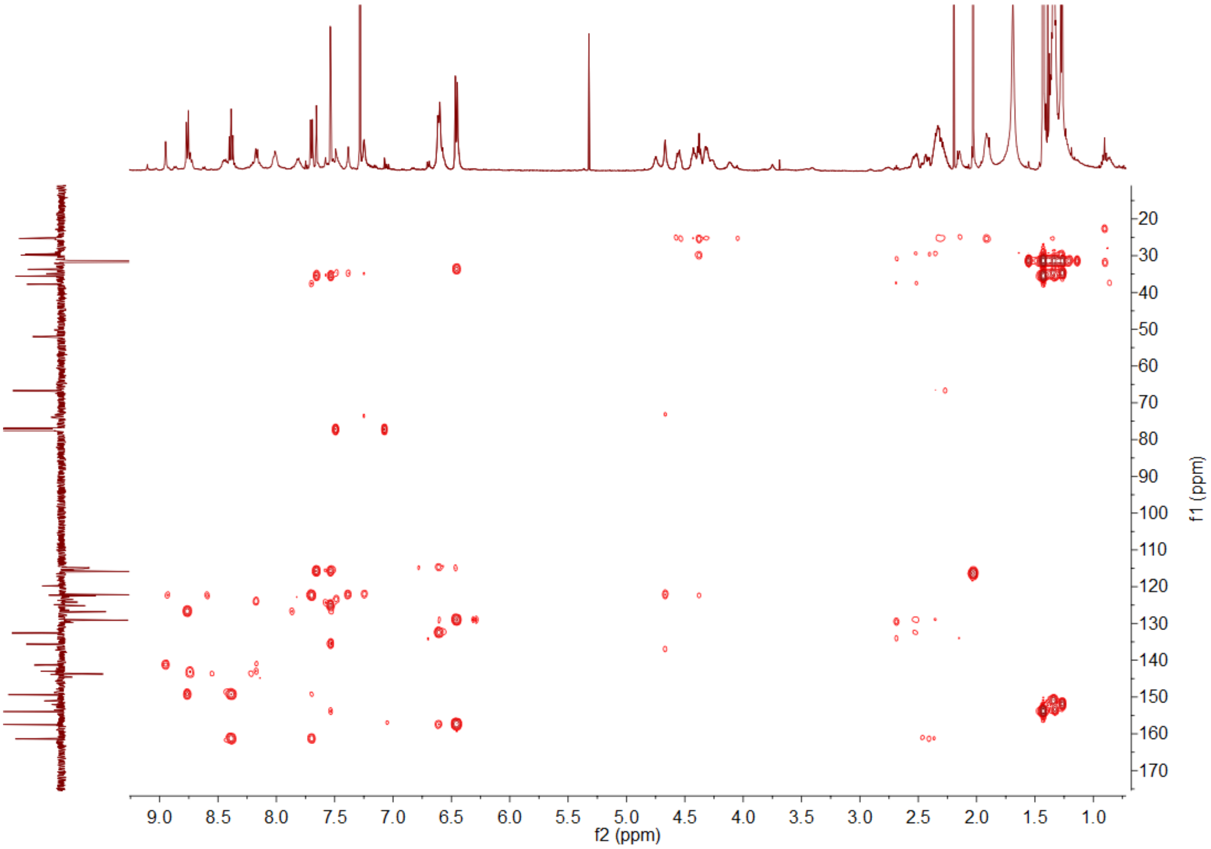

Figure S197 HSQC NMR ($CDCl_3$, 500 MHz) of [Zn(9a)]$(OTf)_2$.

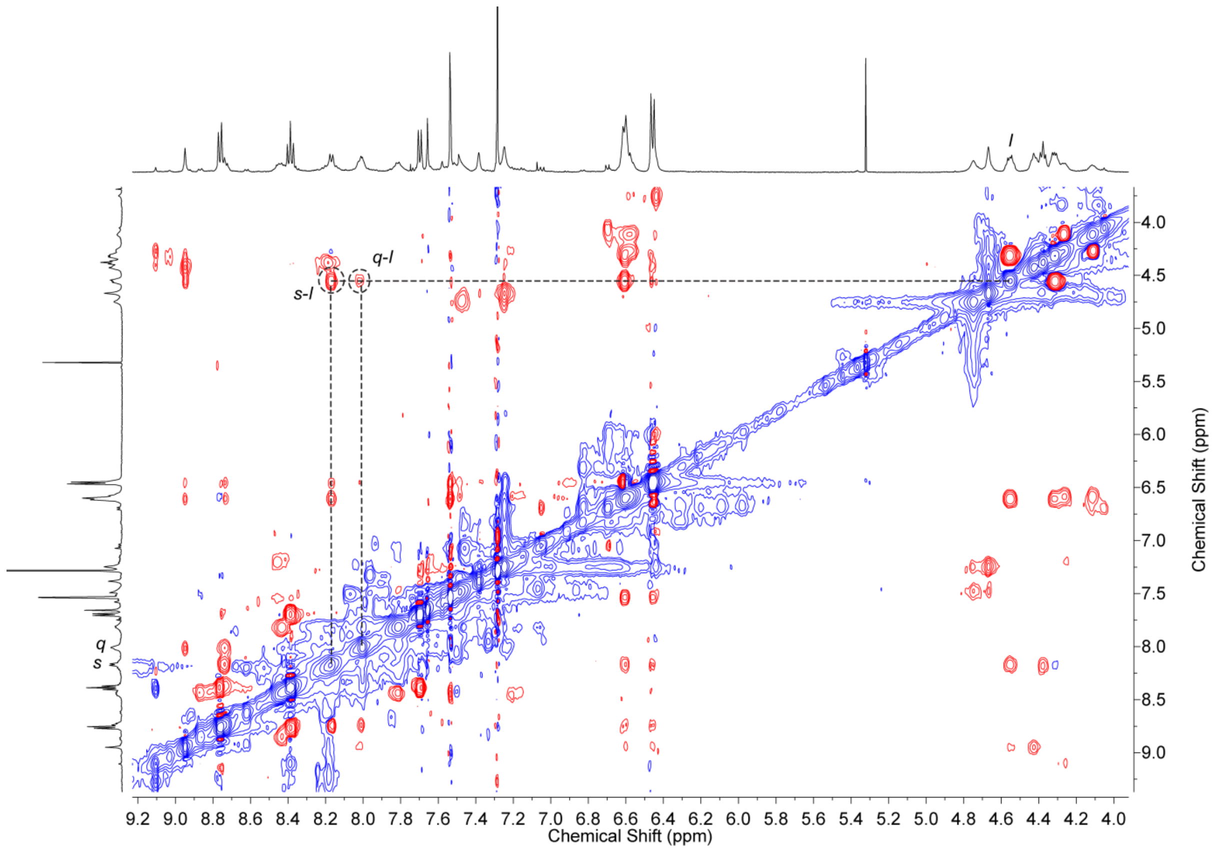

Figure S198 ^{1}H ROESY NMR ($CDCl_3$, 500 MHz) of [Zn(9a)]$(OTf)_2$.

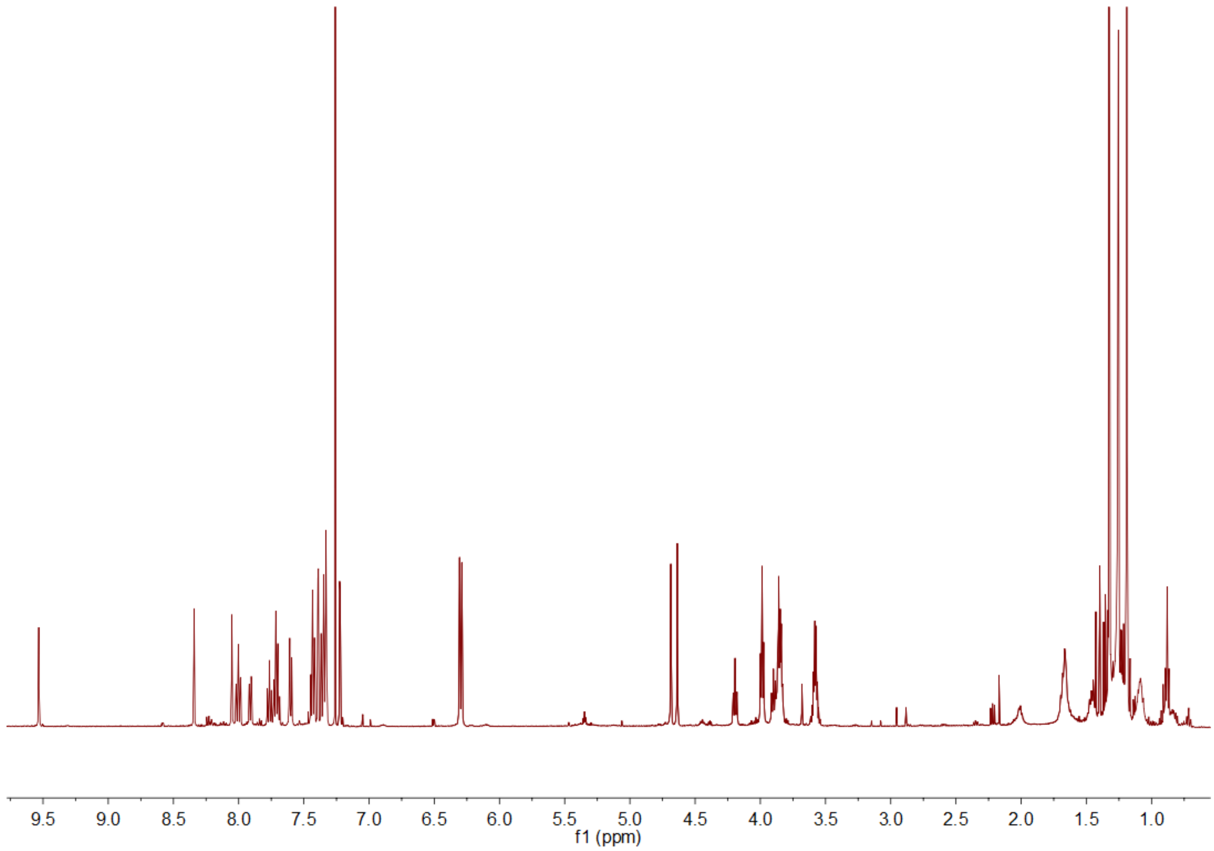

Figure S199 ^{1}H NMR ($CDCl_3$, 500 MHz) of 9e.

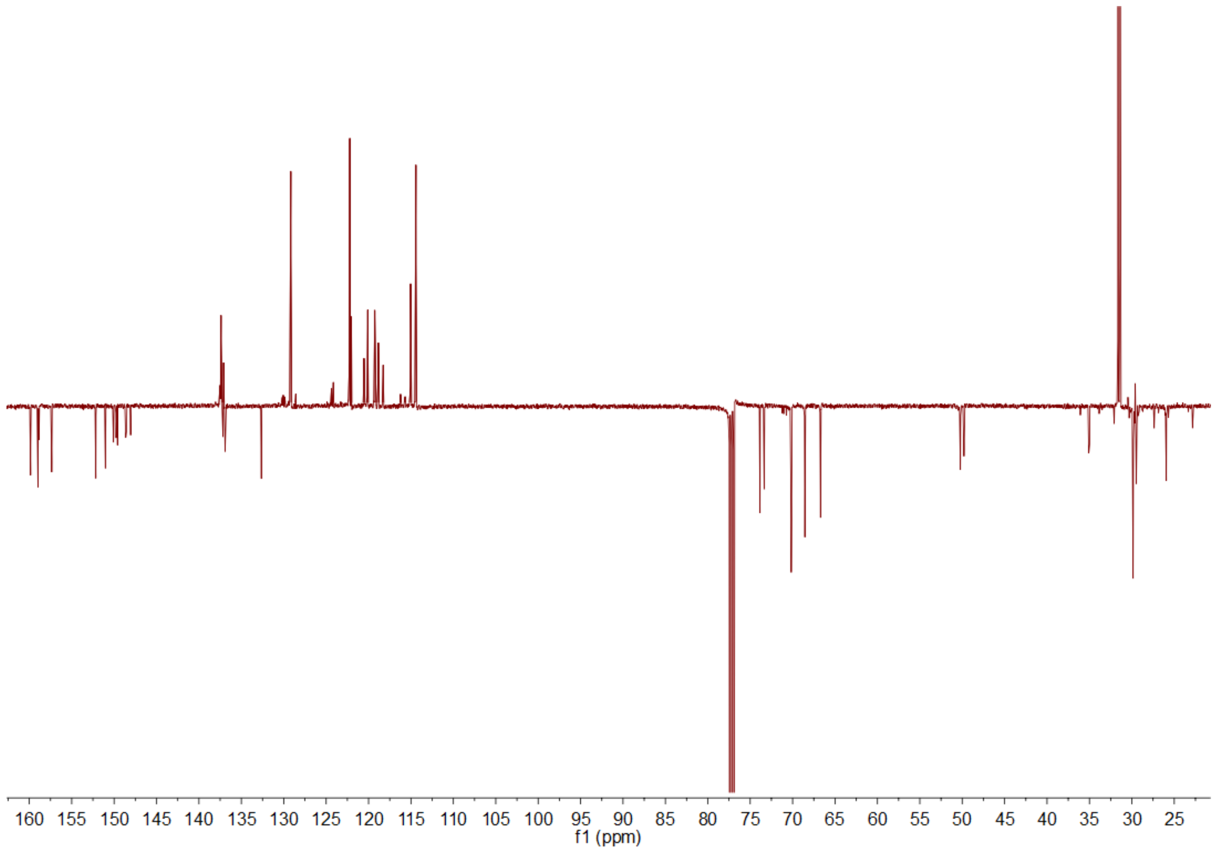

Figure S200 JMOD NMR ($CDCl_3$, 125 MHz) of 9e.

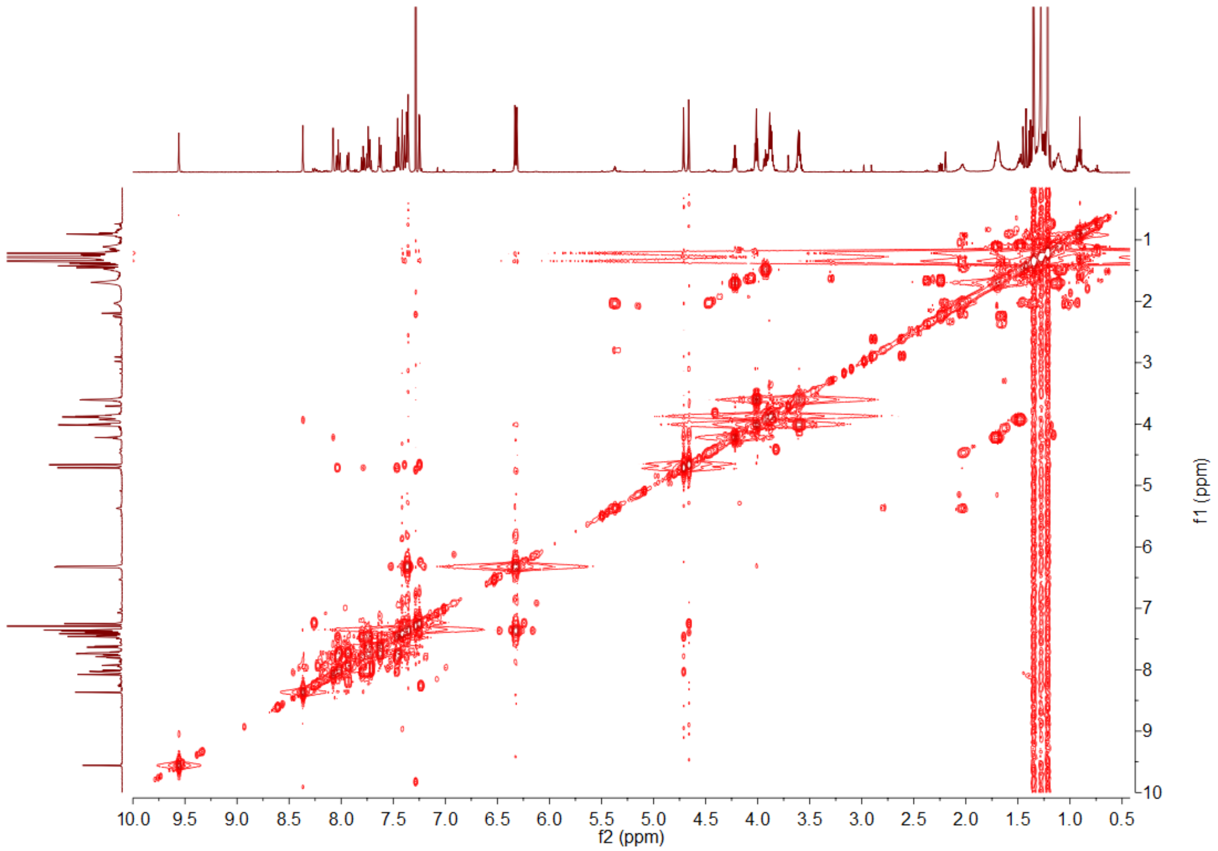

Figure S201 ^{1}H COSY NMR ($CDCl_3$, 500 MHz) of 9e.

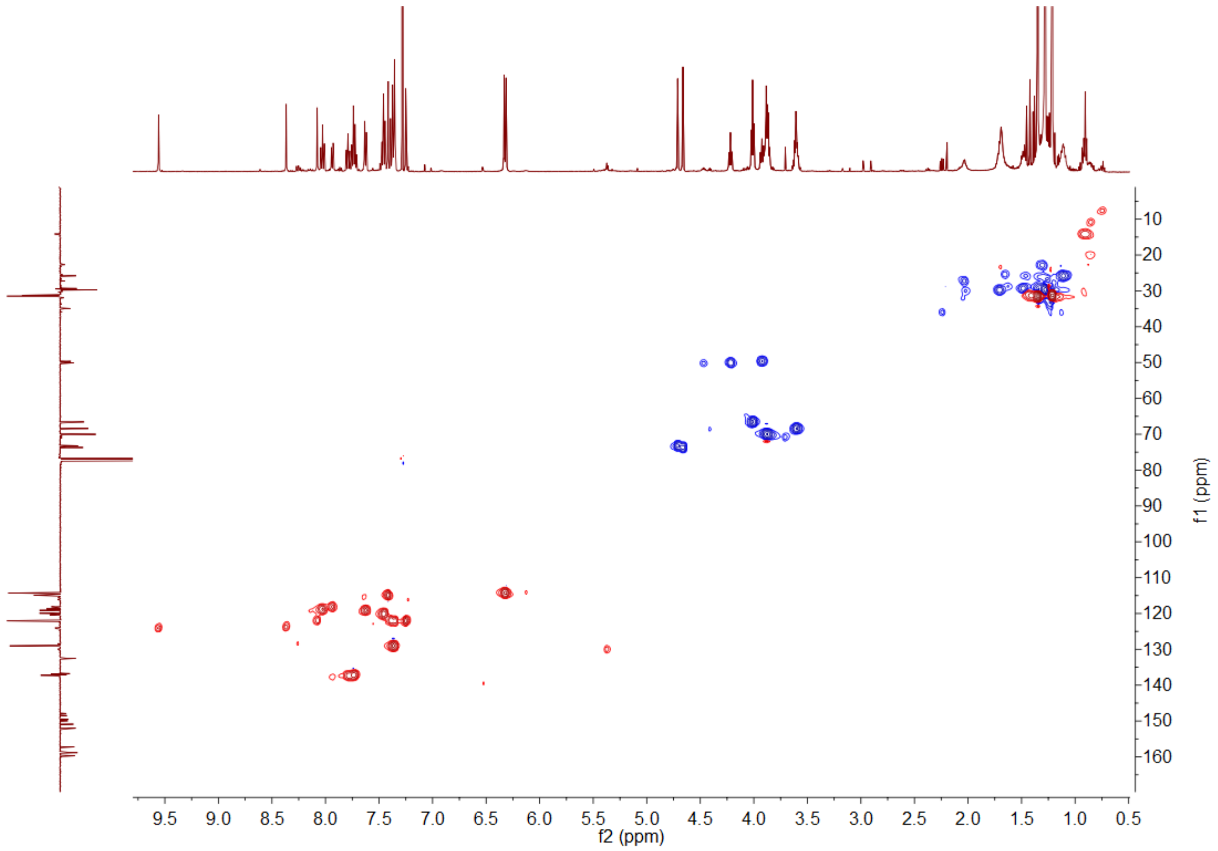

Figure S202 HSQC NMR ($CDCl_3$, 500 MHz) of 9e.

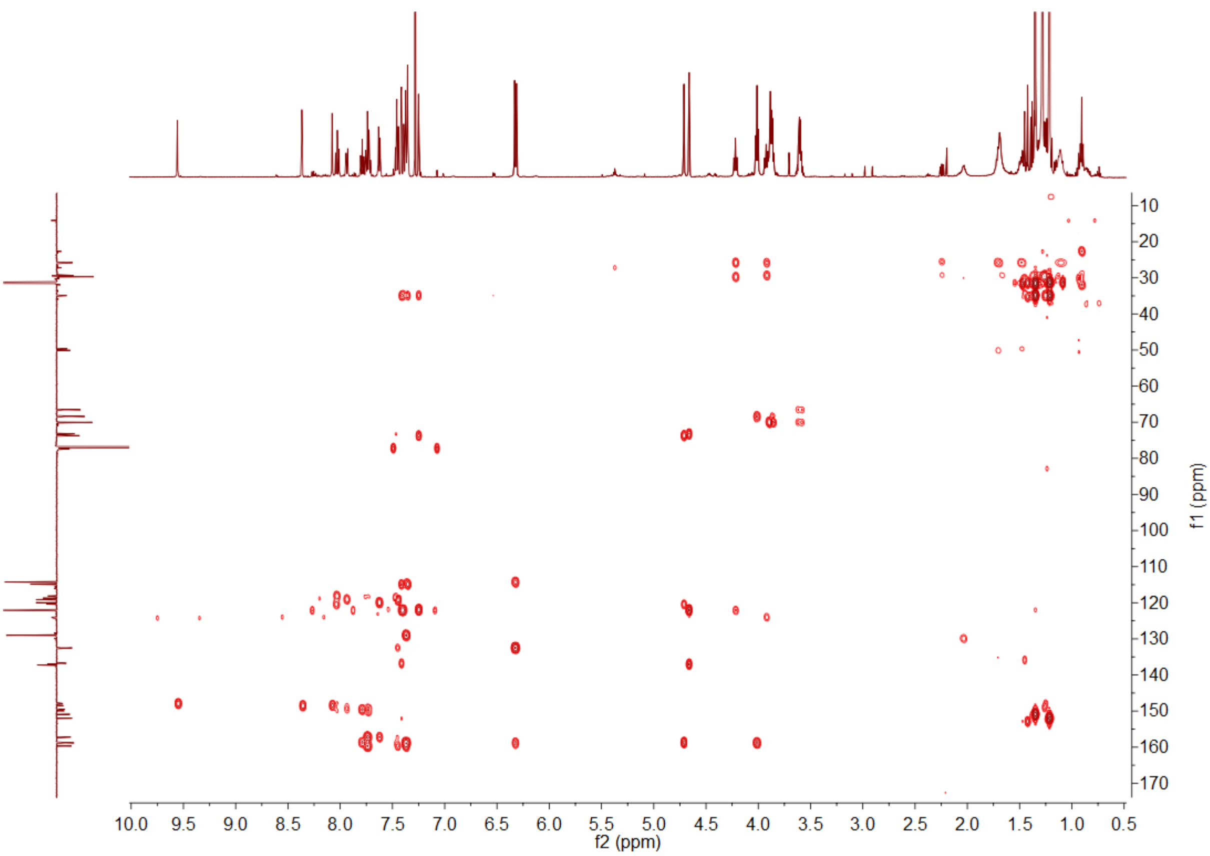

Figure S203 HMBC NMR ($CDCl_3$, 500 MHz) of 9e.

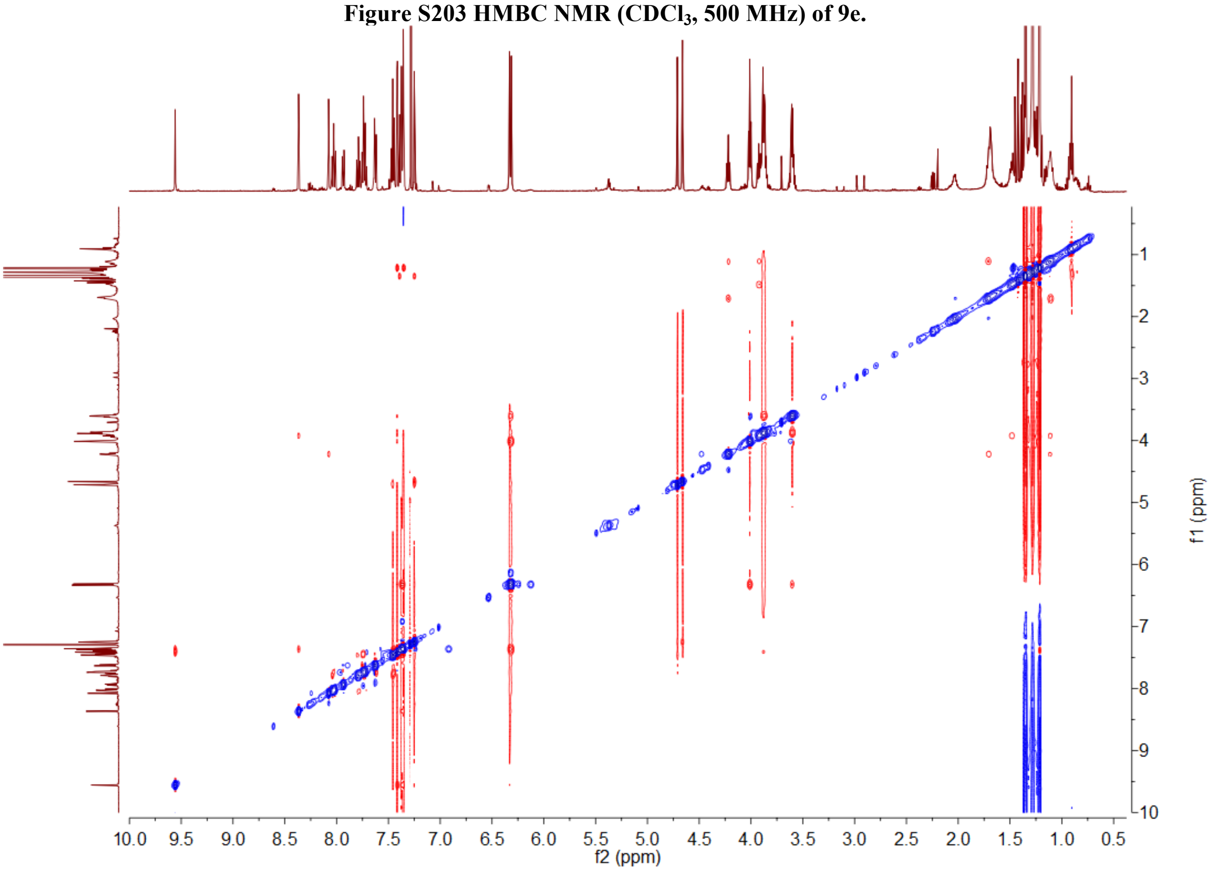

Figure S204 ^1H ROESY NMR ($CDCl_3$, 500 MHz) of 9e.

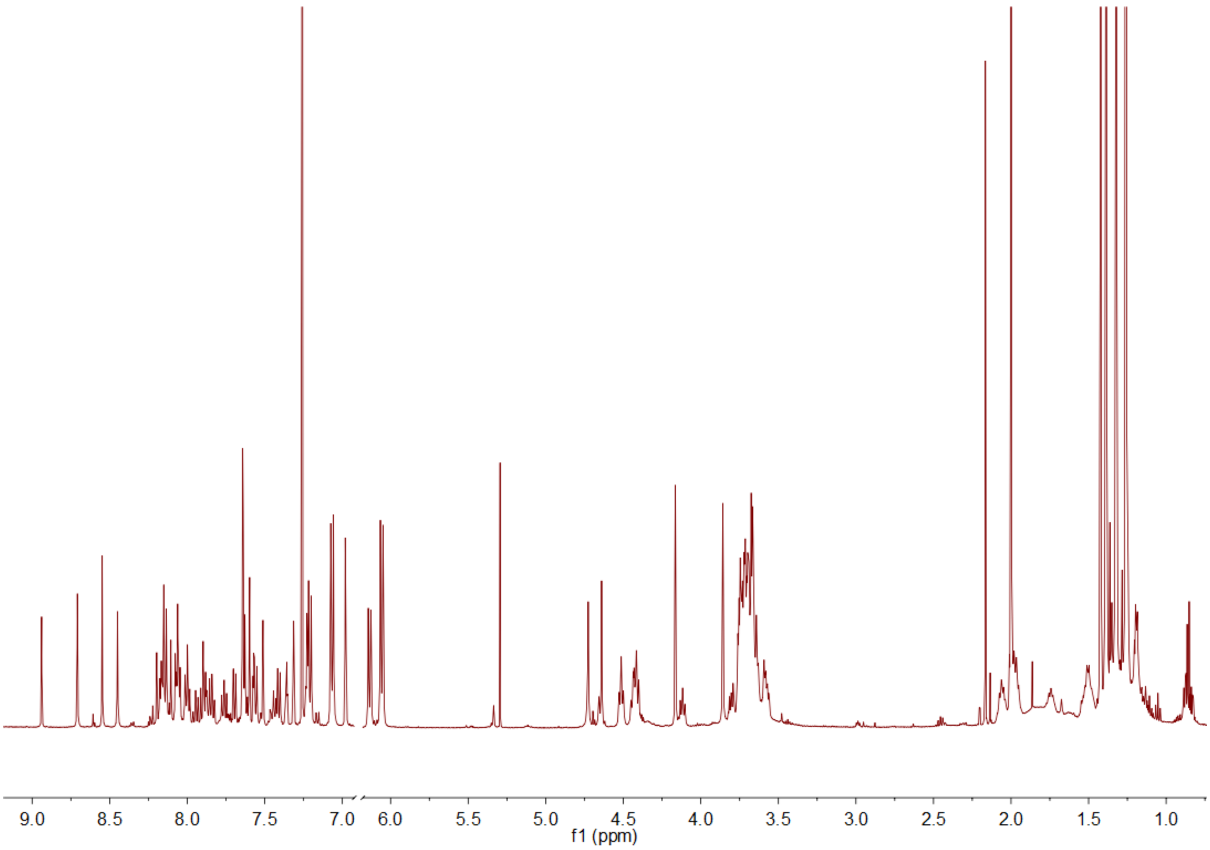

Figure S205 ^{1}H NMR ($CDCl_3$, 500 MHz) of [Cu(9e)](PF_6).

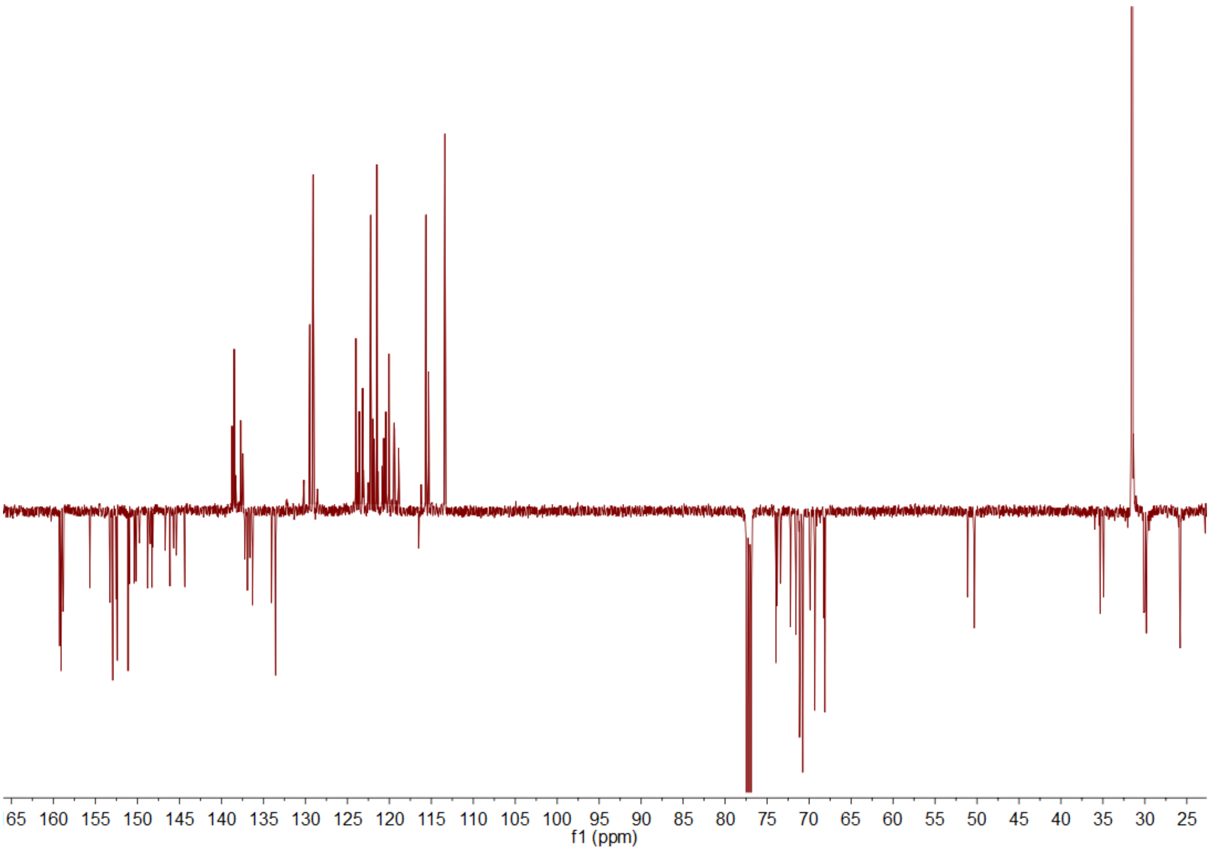

Figure S206 JMOD NMR ($CDCl_3$, 125 MHz) of [Cu(9e)](PF_6).

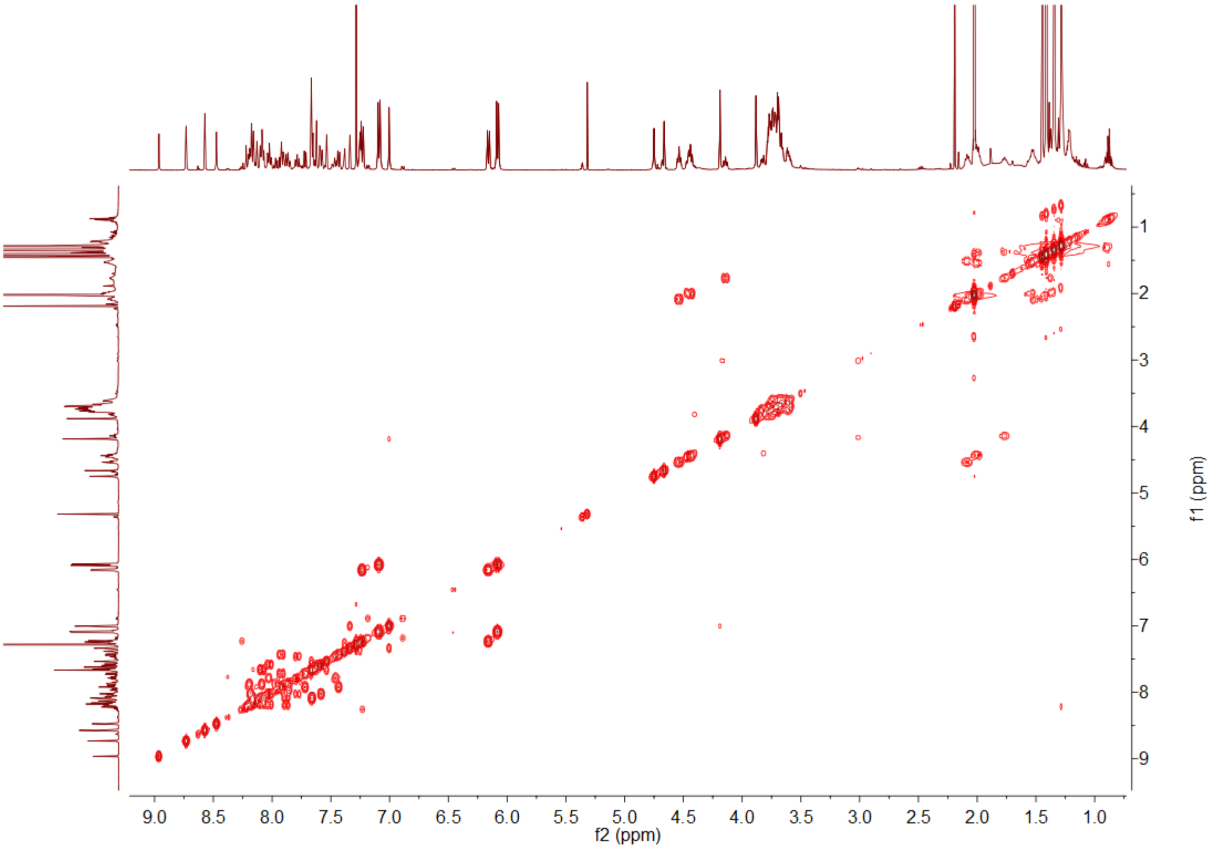

Figure S207 ^{1}H COSY NMR ($CDCl_3$, 500 MHz) of [Cu(9e)](PF_6).

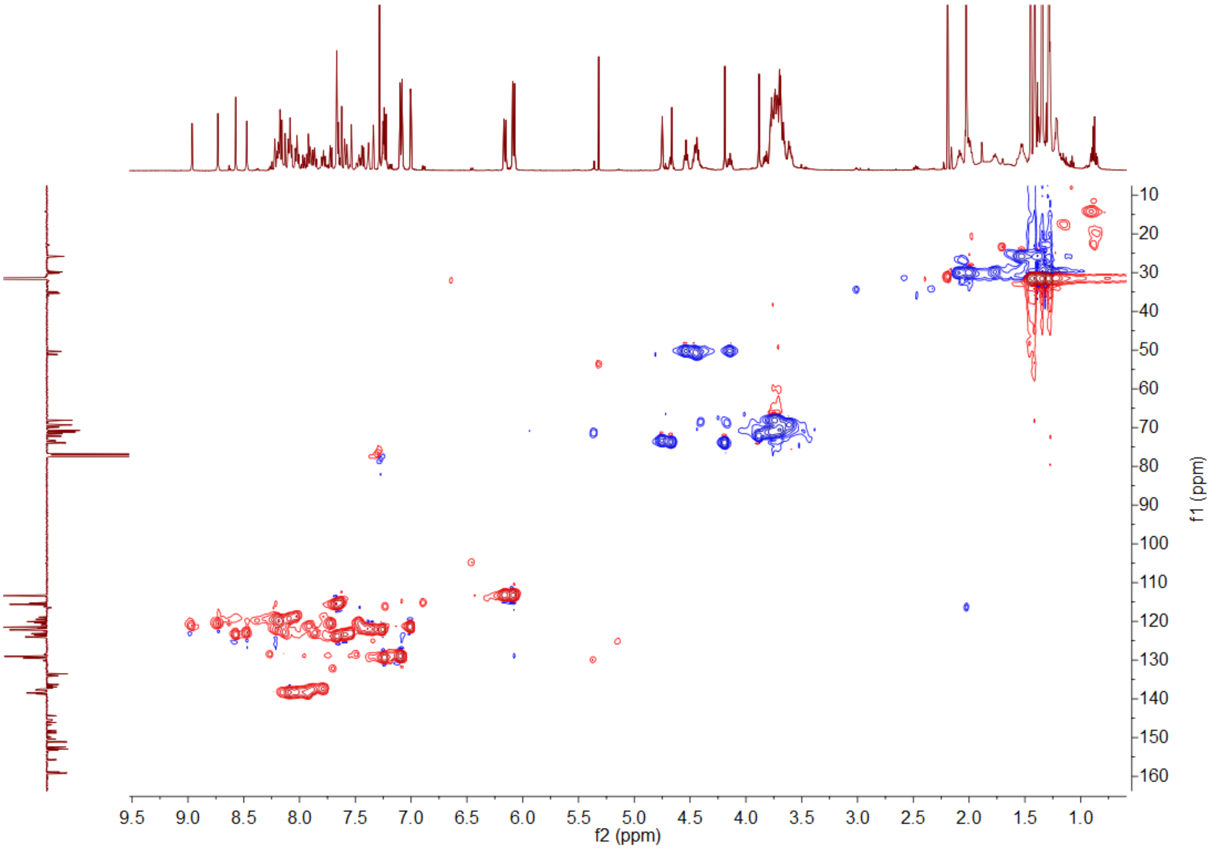

Figure S208 HSQC NMR ($CDCl_3$, 500 MHz) of [Cu(9e)](PF_6).

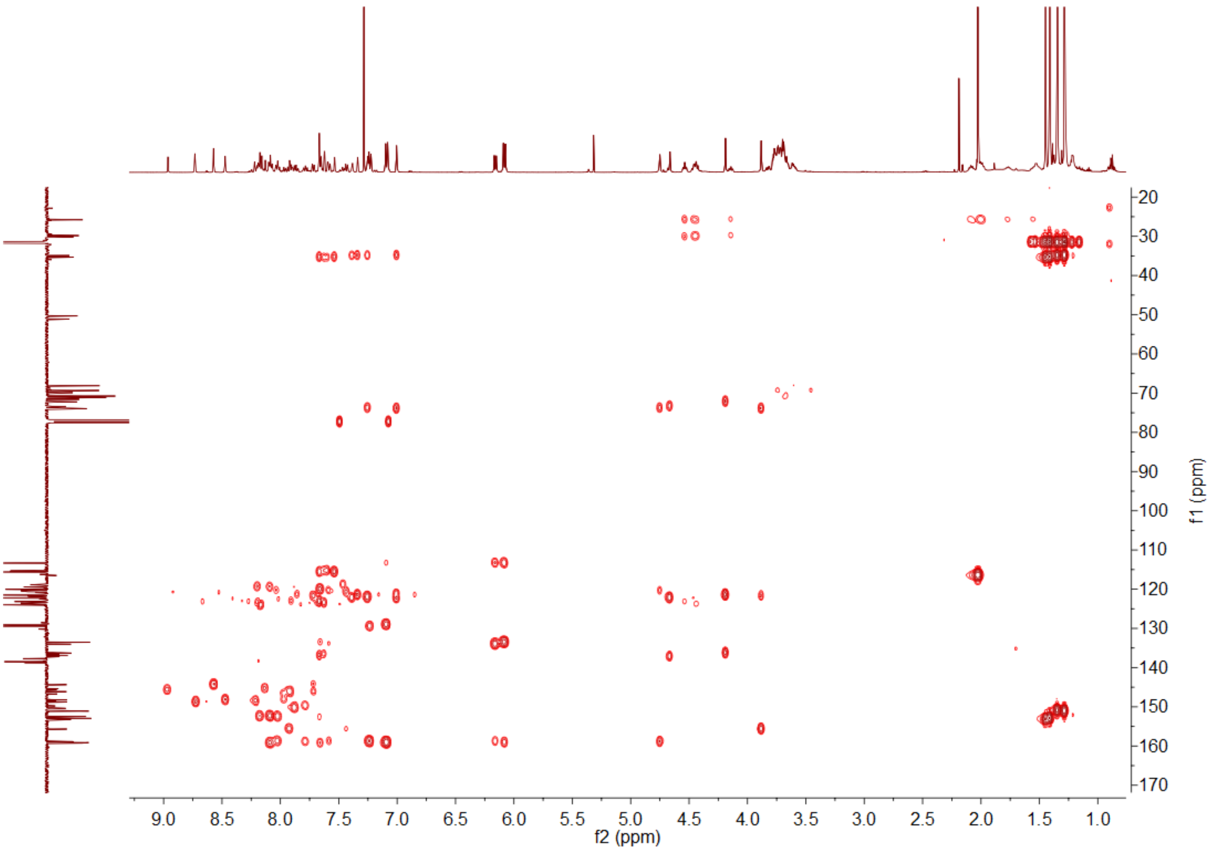

Figure S209 HMBC NMR ($CDCl_3$, 500 MHz) of [Cu(9e)](PF_6).

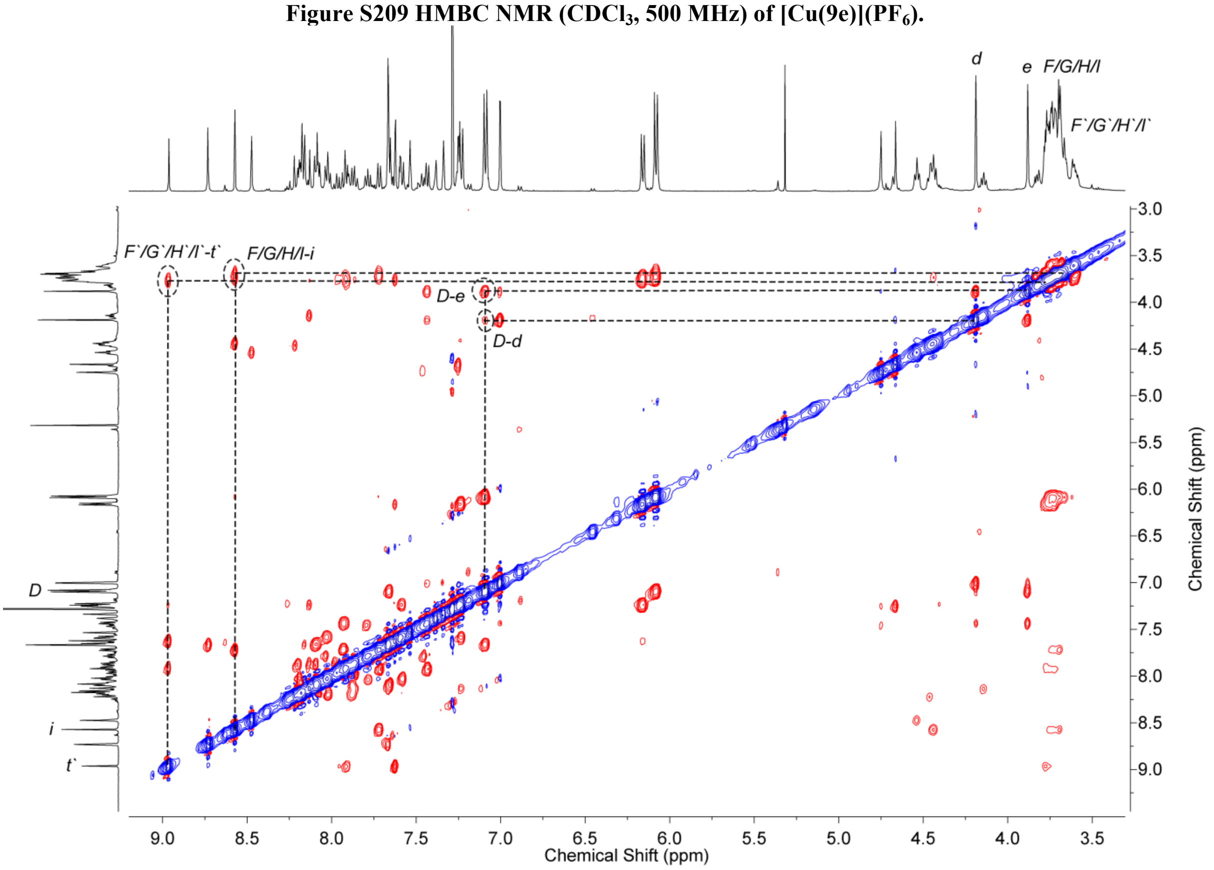

Figure S210 ^{1}H ROESY NMR ($CDCl_3$, 500 MHz) of [Cu(9e)](PF_6).

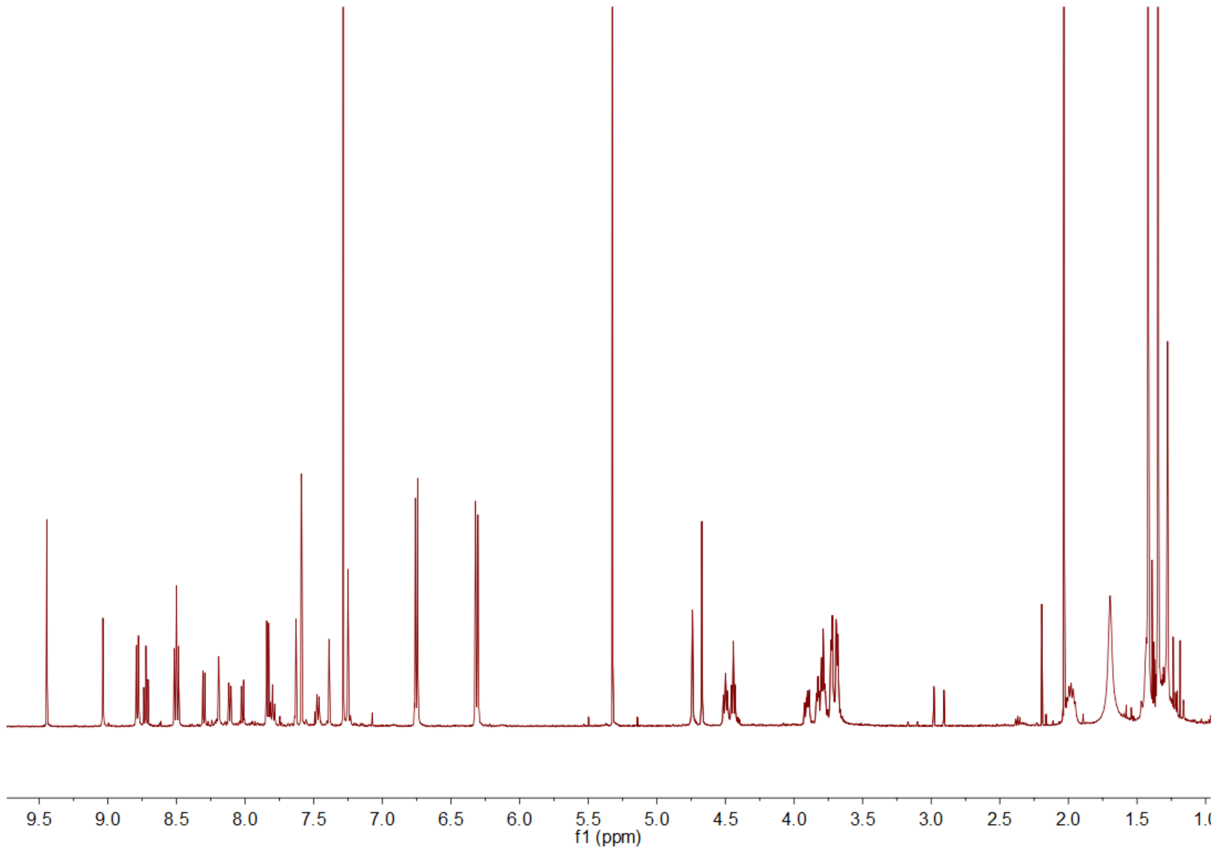

Figure S211 ^{1}H NMR ($CDCl_3$, 500 MHz) of [Zn(9e)]($OTf)_2$.

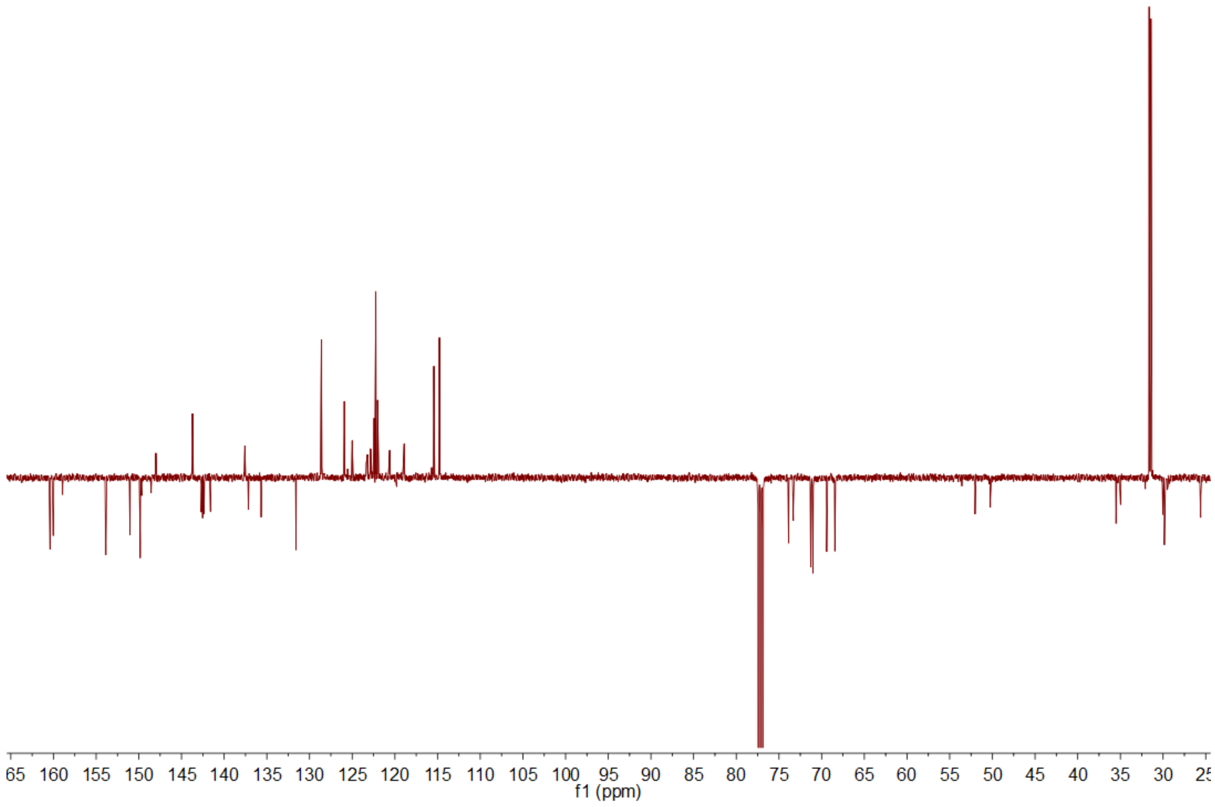

Figure S212 JMOD NMR ($CDCl_3$, 125 MHz) of [Zn(9e)]($OTf)_2$.

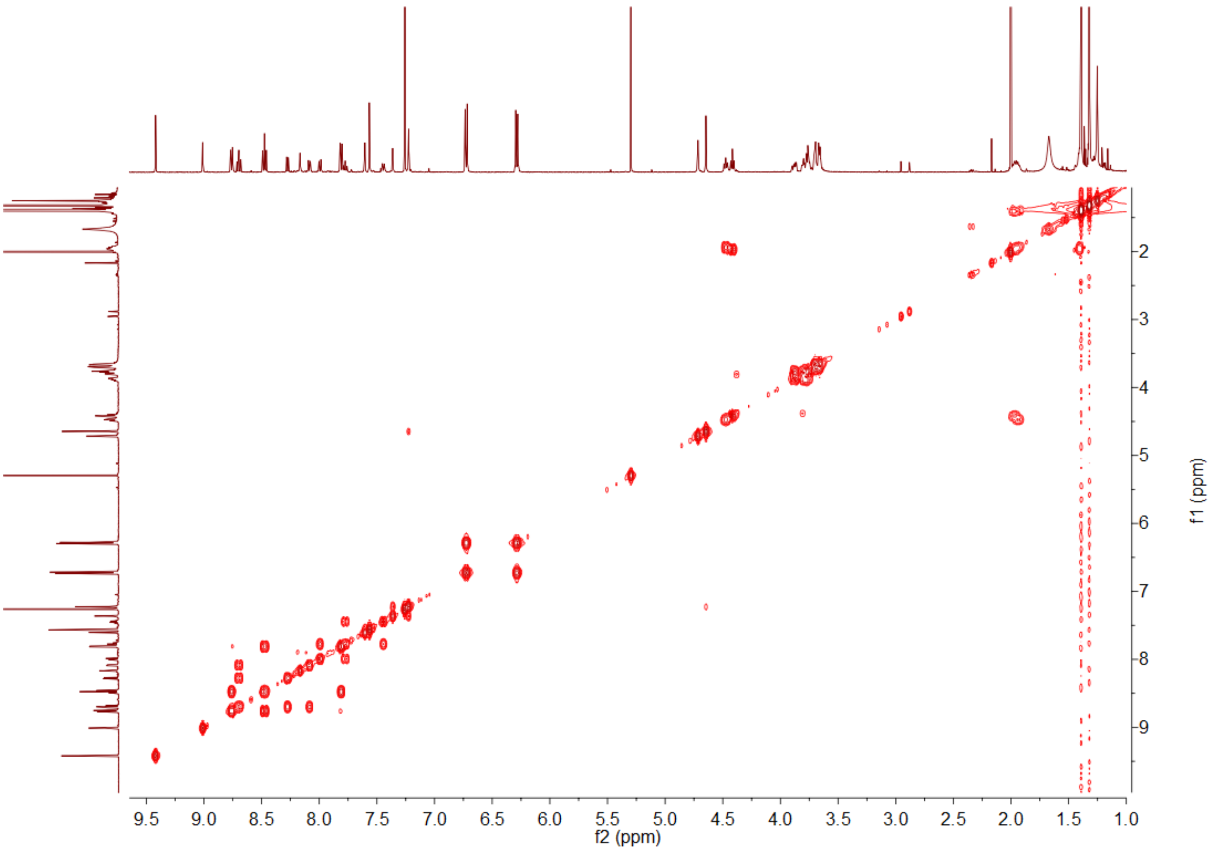

Figure S213 ¹H COSY NMR ($CDCl_3$, 500 MHz) of [Zn(9e)]($OTf)_2$.

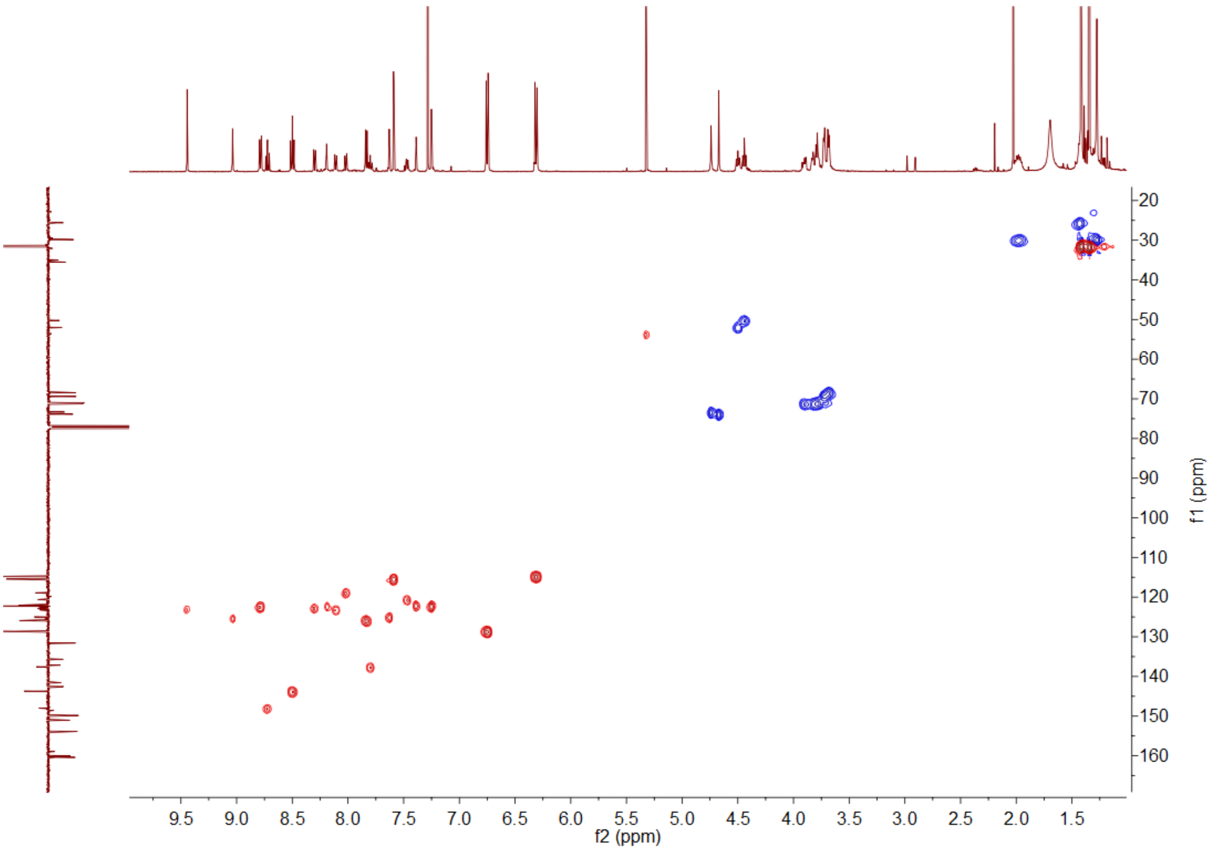

Figure S214 HSQC NMR ($CDCl_3$, 500 MHz) of [Zn(9e)]($OTf)_2$.

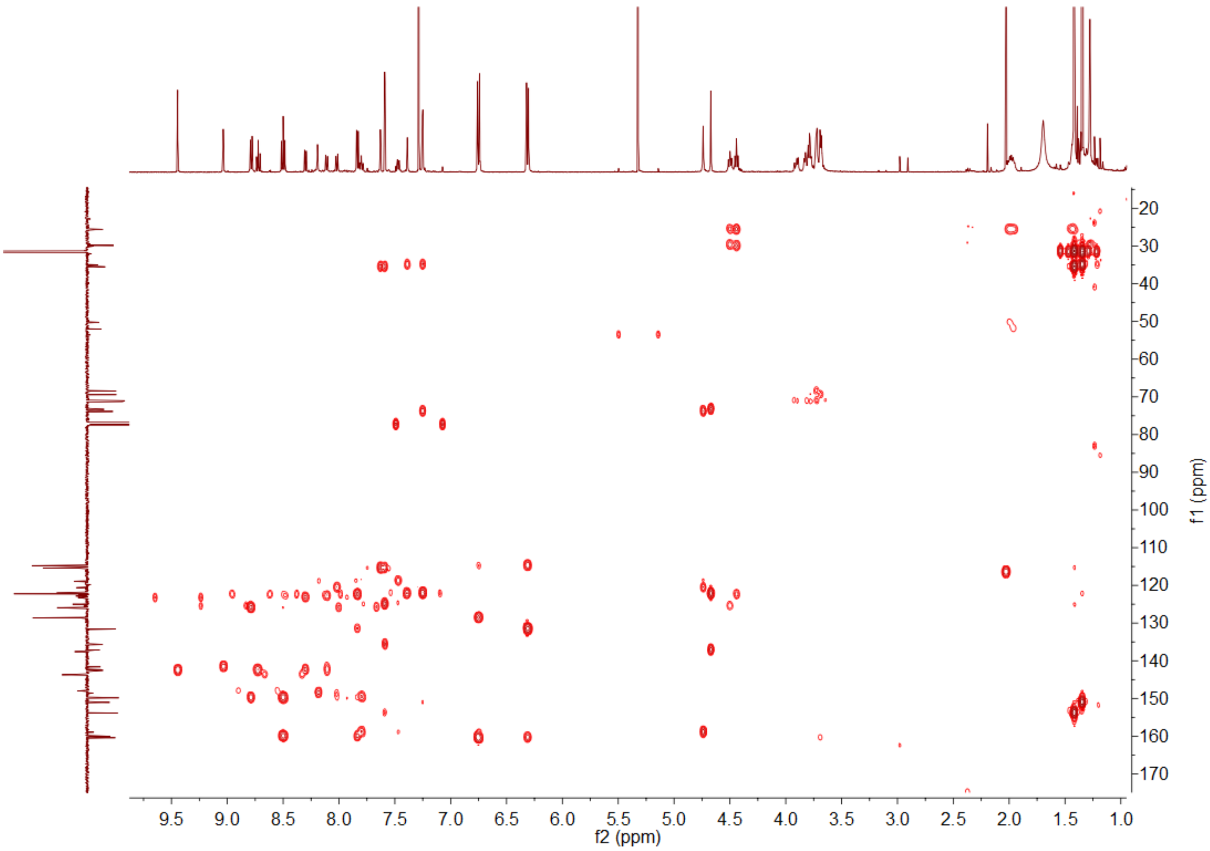

Figure S215 HMBC NMR ($CDCl_3$, 500 MHz) of [Zn(9e)]$(OTf)_2$.

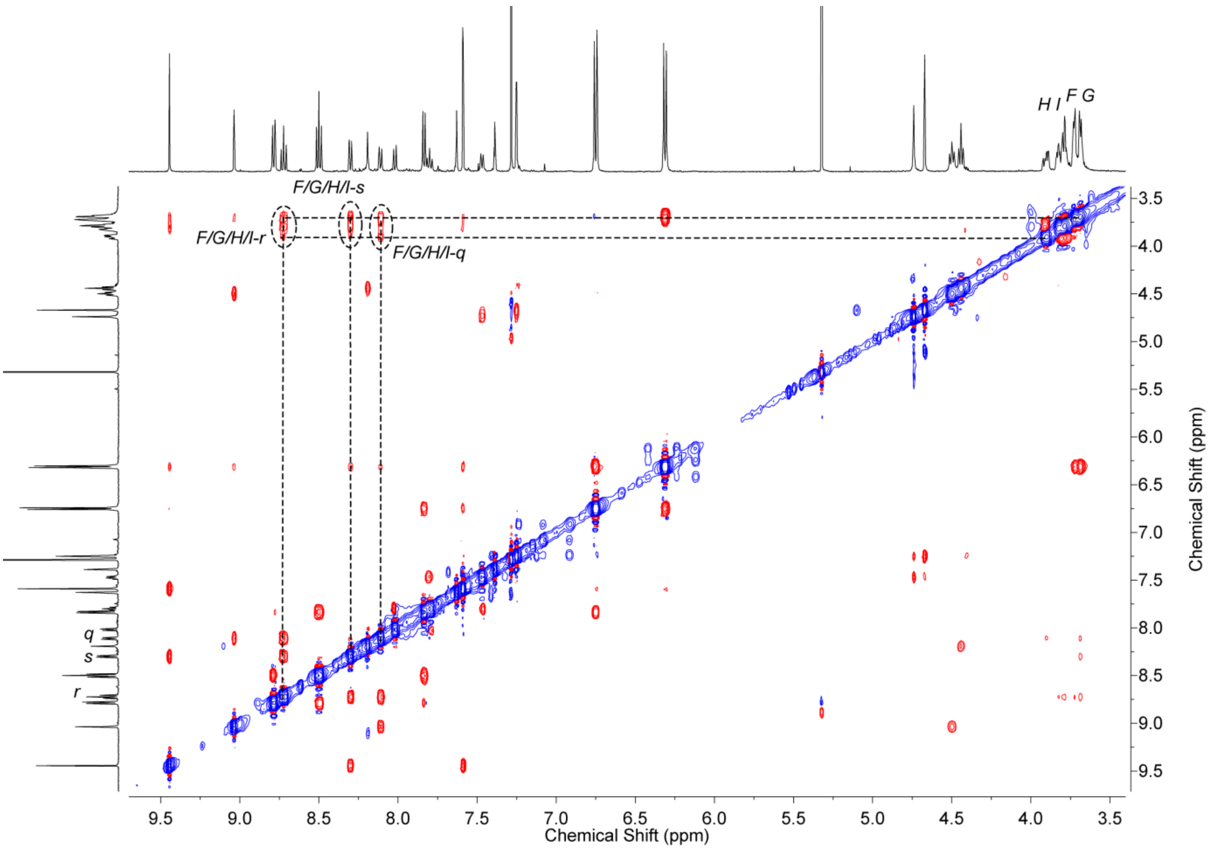

Figure S216 ^{1}H ROESY NMR ($CDCl_3$, 500 MHz) of [Zn(9e)]$(OTf)_2$.

6. Crystalographic Data

Data were collected using a Rigaku AFC12 goniometer equipped with an enhanced sensitivity (HG) Saturn724+ detector mounted at the window of an FR-E+ SuperBright molybdenum (λ = 0.71073 Å) rotating anode generator with VHF Varimax optics (70 µm focus) with the crystal held at 100 K (N_2 cryostream). Cell determination and data collection were done using CrystalClear-SM Expert 3.1; data reduction, cell refinement and absorption correction were performed with CrystalClear-SM Expert 2.1. All structures were solved using SUPERFLIP and refined against F_2 using anisotropic thermal displacement parameters for all non-hydrogen atoms, except where noted below, using WINGX and software packages within. Hydrogen atoms were placed in calculated positions and refined using a riding model.

X-ray Data for S12·CH_2Cl_2

CCDC Number	1442684	
Empirical formula	$C_{61}H_{75}Cl_2N_5O_5$	
Formula weight	1029.16	
Temperature	293(2) K	
Wavelength	0.71073 Å	
Crystal system	Monoclinic	
Space group	P 21/c	
Unit cell dimensions	a = 10.1938(16) Å	α= 90°
	b = 25.558(4) Å	β= 91.243(2)°
	c = 21.504(3) Å	γ = 90°
Volume	5601.2(15) Å^3	
Z	4	
Density (calculated)	1.220 Mg/m^3	
Absorption coefficient	0.169 mm^{-1}	
F(000)	2200	
Crystal size	0.100 × 0.070 × 0.030 mm^3	
Theta range for data collection	2.368 to 27.526°.	
Index ranges	-13<=h<=12, -33<=k<=33, -27<=l<=27	
Reflections collected	55328	
Independent reflections	12877 [R(int) = 0.0938]	
Completeness to theta = 25.242°	99.9 %	
Absorption correction	Semi-empirical from equivalents	
Max. and min. transmission	1.000 and 0.456	
Refinement method	Full-matrix least-squares on F^2	
Data / restraints / parameters	12877 / 0 / 727	
Goodness-of-fit on F^2	0.865	
Final R indices [I>2sigma(I)]	R1 = 0.0840, wR2 = 0.2327	
R indices (all data)	R1 = 0.1553, wR2 = 0.3066	
Extinction coefficient	n/a	
Largest diff. peak and hole	0.543 and -0.845 e.Å^{-3}	

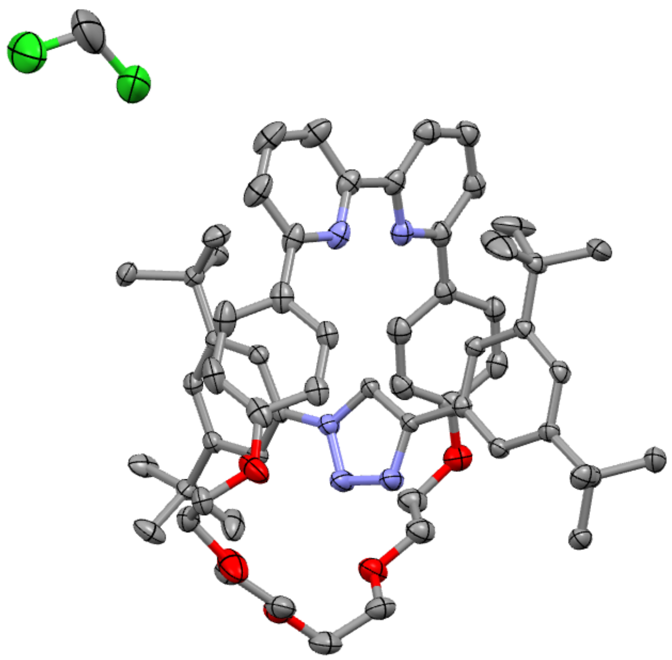

Figure S217 **Ellipsoid plot of the asymmetric unit of S12·CH_2Cl_2. Ellipsoids are shown at the 50% probability level. Hydrogen atoms and disordered atoms have been omitted for clarity.**

X-ray Data for {[Cu(2d)](PF_6)}$_2$·$(CH_3Cl)(CH_2Cl_2)$

CCDC Number	1442682	
Empirical formula	$C_{66}H_{69}Cl_5Cu_2F_{12}N_6O_8P_2$	
Formula weight	1668.54	
Temperature	293(2) K	
Wavelength	0.71073 Å	
Crystal system	Monoclinic	
Space group	P 21/c	
Unit cell dimensions	a = 14.590(3) Å	α= 90°
	b = 17.504(4) Å	β= 101.456(3)°
	c = 28.499(6) Å	γ = 90°
Volume	7133(3) $Å^3$	
Z	4	
Density (calculated)	1.554 Mg/m^3	
Absorption coefficient	0.918 mm^{-1}	
F(000)	3408	
Crystal size	0.069 × 0.054 × 0.024 mm^3	
Theta range for data collection	2.232 to 27.566°.	
Index ranges	-18<=h<=18, -22<=k<=22, -37<=l<=35	
Reflections collected	70413	
Independent reflections	16433 [R(int) = 0.0795]	
Completeness to theta = 25.242°	99.9 %	
Absorption correction	Semi-empirical from equivalents	
Max. and min. transmission	1.000 and 0.412	
Refinement method	Full-matrix least-squares on F^2	
Data / restraints / parameters	16433 / 3 / 957	
Goodness-of-fit on F^2	1.267	
Final R indices [I>2sigma(I)]	R1 = 0.1095, wR2 = 0.3244	
R indices (all data)	R1 = 0.1628, wR2 = 0.3630	
Extinction coefficient	n/a	
Largest diff. peak and hole	2.927 and -1.731 e.$Å^{-3}$	

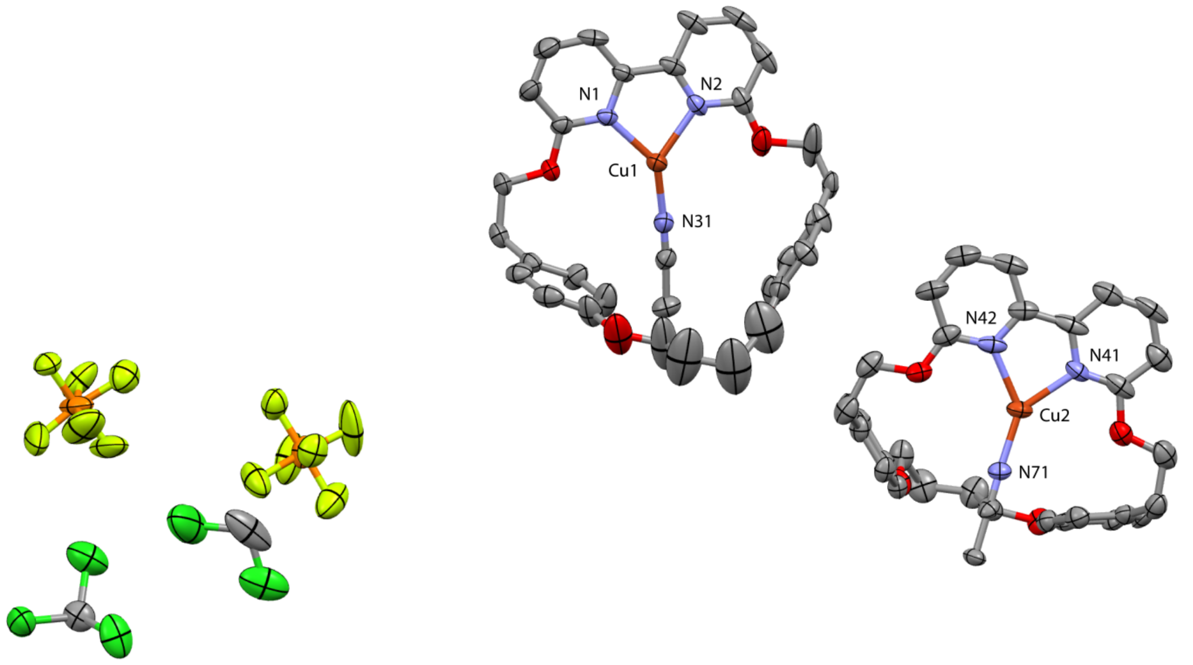

Figure S218 Ellipsoid plot of the asymmetric unit of {[Cu(2d)](PF_6)}$_2$·(CH_3Cl)(CH_2Cl_2). Ellipsoids are shown at the 50% probability level. Hydrogen atoms and disordered atoms have been omitted for clarity. Bond lengths (Å) and angles (°): N1-Cu1 2.033 (5), N2-Cu1 2.029(5), N31-Cu1 1.867(7), N1-Cu1-N2 81.2(2), N41-Cu2 2.007(6), N42-Cu2 2.050(6), N71-Cu2 1.858(5), N41-Cu2-N42 80.6(2).

X-ray Data for 2d Dimer

CCDC Number	1442683	
Empirical formula	$C_{60}H_{60}N_4O_8$	
Formula weight	965.12	
Temperature	293(2) K	
Wavelength	0.71073 Å	
Crystal system	Triclinic	
Space group	P -1	
Unit cell dimensions	a = 9.688(2) Å	α= 100.5350(10)°
	b = 11.374(2) Å	β= 110.1260(10)°
	c = 13.628(3) Å	γ = 109.435(2)°
Volume	1252.4(4) Å^3	
Z	1	
Density (calculated)	1.280 Mg/m^3	
Absorption coefficient	0.085 mm^{-1}	
F(000)	512	
Crystal size	0.161 × 0.131 × 0.042 mm^3	
Theta range for data collection	2.311 to 27.630°.	
Index ranges	-12<=h<=12, -14<=k<=14, -17<=l<=17	
Reflections collected	24806	
Independent reflections	5778 [R(int) = 0.0721]	
Completeness to theta = 25.242°	99.9 %	
Absorption correction	Semi-empirical from equivalents	
Max. and min. transmission	1.000 and 0.4852	
Refinement method	Full-matrix least-squares on F^2	
Data / restraints / parameters	5778 / 0 / 325	
Goodness-of-fit on F^2	1.034	
Final R indices [I>2sigma(I)]	R1 = 0.0515, wR2 = 0.1451	
R indices (all data)	R1 = 0.0667, wR2 = 0.1645	
Extinction coefficient	n/a	
Largest diff. peak and hole	0.342 and -0.235 e.Å^{-3}	

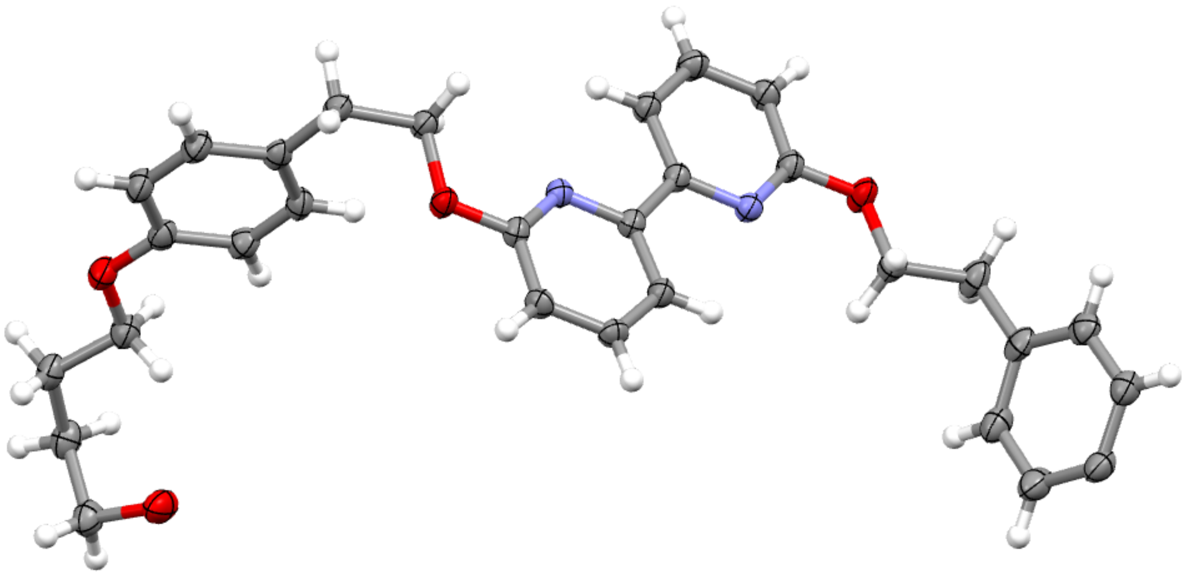

Figure S219 Ellipsoid plot of the asymmetric unit of the macrocycle 2d dimer. Ellipsoids are shown at the 50% probability level.

7. References

1) J. Winn, A. Pinczewska and S. M. Goldup, *J. Am. Chem. Soc.*, 2013, **135**, 13318.
2) R. J. Bordoli, S. M. Goldup, *J. Am. Chem. Soc.* **2014**, *136*, 4817.
3) R. S. Stoll, M. V. Peters, A. Kuhn, S. Heiles, R. Goddard, M. Bühl, C. M. Thiele, S. Hecht, *J. Am. Chem. Soc.,* 2009, **131**, 357.
4) E. M. Schuster, M. Botoshansky, M. Gandelman, *Angew. Chem. Int. Ed.,* 2008, **47**, 4555.
5) W. Zhu and D. Ma, *Chem. Commun.*, 2004, 888.
6) J. J. Gassensmith, L. Barr, J. M. Baumes, A. Paek, A. Nguyen and B. D. Smith, *Org. Lett.,* 2008, **10**, 3343.
7) H. Lahlali, K. Jobe, M. Watkinson, and S. M. Goldup, *Angew. Chem. Int. Ed.*, 2011, **50**, 4151.
8) V. Aucagne, J. Berna, J. D. Crowley, S. M. Goldup, K. D. Hänni, D. A. Leigh, P. J. Lusby, V. E. Ronaldson, A. M. Z. Slawin, A. Viterisi and D. B. Walker, *J. Am. Chem. Soc.,* 2007, **129**, 11950-11963.
9) M. Galli, J. E. M. Lewis and S. M. Goldup, *Angew. Chem. Int. Ed.,* 2015, **54**, 13545–13549.
